# Supplementary material for: Implementing the compassion intervention, a model for integrated care for people with advanced dementia towards the end of life in nursing homes: a naturalistic feasibility study
Source: BMJ Open. 2017 Jul 10;7(6):e015515. doi: 10.1136/bmjopen-2016-015515 (PMC5541605; doi:10.1136/bmjopen-2016-015515)
Supplement: Supplementary material 1 [file bmjopen-2016-015515supp001.pdf]

The Compassion Programme

(Care Of Memory Problems in Advanced Stages: Improving Our kNowledge)

Work-stream 3: Pilot study of enhanced integrated care for people with severe memory problems

---

Principal Investigator: Dr Louise Jones

Study Funders: Marie Curie Cancer Care

SYNOPSIS OF PROGRAMME 8
BACKGROUND 9
Epidemiological background 9
The clinical picture 9
Challenges 9
DEVELOPMENT OF THE COMPASSION MODEL OF ENHANCED CARE 10
Work stream 1 10
Work stream 2 10
Synthesis of findings and development of the enhanced model of care 11
AIMS AND OBJECTIVES OF PILOT STUDY 12
Objectives 12
RECRUITMENT AND CONSENT PROCEDURES 13
Location 13
Recruitment of care homes 13
Consent for implementing the enhanced model of care within the care home 13
Informing participants about the study 14
Recruitment of people with severe memory problems for evaluation of outcomes 14
Consent Procedures 16
Recruitment of family carers to give information for the evaluation 19
Recruitment of enhanced care team and care home staff to participate in qualitative interviews 20
Potential risks/strengths 21
PROJECT INTERVENTION 22
Preparing the Compassion Intervention manual for the enhanced model of care 22
Overview of the intervention 23
DATA COLLECTION 26

Enhanced care team process data 28

Care home level process data 28

Acceptability of the intervention to care home and enhanced care team staff 29

Outcomes for care home residents receiving the intervention 29

Outcomes for family carers 31

DATA ANALYSIS 34

Quantitative analysis 34

Qualitative analysis 34

Final analyses 35

Sample size 35

Health economics 35

PROJECT MANAGEMENT 35

Project Staffing 36

Core study team 36

Membership of our expert steering group 37

Patient and Public Involvement 38

STUDY OUTPUTS 38

REFERENCES 38

Appendix 1: Compassion intervention manual 42

Appendix 2. Bedford Alzheimer Nursing Severity (BANS) scale 42

Appendix 3: Waterlow scale 42

Appendix 4: Stirling Wound Assessment Scale 42

Appendix 5: Pain Assessment in Advanced Dementia (PAIND) 42

Appendix 6: Cohen Mansfield Agitation Inventory (CMAI) 42

Appendix 7: Neuropsychiatric Inventory (NPI) questionnaire 42

Appendix 8: Symptom Management at the End Of Life in Dementia (SM-EOLD) scale 42

Appendix 9: Quality of Life in late-stage Dementia (QUALID) 42

Appendix 10: Resource Utilisation in Dementia (RUD) - Lite.. 43

Appendix 11: The Comfort Assessment in Dying with Dementia scale (CAD-EOLD) 43

Appendix 12: The Zarit Burden Interview.. 43

Appendix 13: Hospital Anxiety and Depression Scale (HADS) 43

Appendix 14: The Satisfaction with Care at the End-of-Life in Dementia Questionnaire (SWC/CAD-EOLD).. 43

Appendix 15: EQ-5D-5L 43

Appendix 16: Health Care Professional Qualitative Interview Schedule 44

Appendix 17: Family Carer Qualitative Interview Schedule.. 46

Table 1: Abbreviations

BANS	Bedford Alzheimer Nursing Scale
BPSD	Behavioural and Psychological Symptoms of Dementia
CAD-EOLD	The Comfort Assessment in Dying with Dementia Scale
CCG	Clinical Commissioning Group
CCI	Charlson Co-morbidity Index
CMAI	Cohen Mansfield Agitation Inventory
CRF	Case Report Form
CRM	Cluster Representation Mechanism
DSM-IV	Diagnostic and Statistical Manual
FAST	Functional Assessment Staging
GP	General Practitioner
HADS	Hospital Anxiety Scale
HCPs	Health Care Professionals
ICL	Interdisciplinary Care Leader
MRC	Medical Research Council
NPI	The Neuropsychiatric Inventory
PAINAD	Pain Assessment in Advanced Dementia
QALY's	Quality-Adjusted Life Years
QUALID	The Quality of Life in Late Stage Dementia
RAM	Rand Appropriateness Method
RUD-Lite	Resource Utilisation in Dementia
SM-EOLD	Symptom Management at the End of Life in Dementia Scale
SWC/CAD-EOLD	The Satisfaction with Care/Care at dying at the End of Life in Dementia

A note on terminology:

Two groups of carers need to be considered in people with severe memory problems: family (unpaid, informal) carers and paid (formal) carers. Here we use "family carer" as: "someone of any age providing unpaid support to family or friends" (Carers UK). No term is ideal and not all unpaid care is provided by families; "informal carer" is seen to minimise the carer role; and "unpaid carer" suggests a form of voluntary work. Thus "family carer" indicates the family member, friend or other close person acting as the primary unpaid carer for, or key decision maker/supporter of the person with severe memory problems. In addition we refer to "paid carers" in care homes and the community.

Only a third of people with dementia ever receive a formal diagnosis. Therefore in the following protocol, in earlier work streams and information sheets for family and paid carers we have used the term "*severe memory problems*". This allows us to recruit a more representative sample of all those with severe memory problems caused by dementia- many of whom may not have received a previous diagnosis.

SYNOPSIS OF PROGRAMME

The numbers of people living and dying with severe memory problems are increasing. Currently, people with severe memory problems often receive poor quality end of life care. The aim of our research, funded by Marie Curie Cancer Care as a three year programme grant, is to develop and pilot a complex intervention that aims to improve end of life care for people with severe memory problems. In years 1 and 2 we developed the intervention (Compassion), an enhanced model of existing care. In year 3 we now plan to pilot the Compassion Intervention and assess how it operates in practice.

Our research programme has been divided into three consecutive work streams: In work stream one we defined, in detail, the final disease trajectory of people with severe memory problems. We gained an in-depth understanding of the:

- clinical symptom burden;
- health and social care needs of people with severe memory problems,
- current pathways of care as they reach the end of life;
- needs of their family carers

In work stream two we used mixed methods (focus groups and individual interviews with people with early dementia, family carers and health and social care staff) to develop a complex intervention (Compassion) to improve end of life care. We have defined the core components of the Compassion Intervention which aims to enhance current care, and the circumstances needed to operationalize these. This protocol describes the final work stream in which we shall pilot this enhanced model of care in order to learn and understand how it might operate in practice and to obtain data to inform a future definitive trial.

BACKGROUND

Epidemiological background

Approximately 600,000 people in the United Kingdom (UK) have dementia (10% of those over 65 years). By 2026 it is estimated that this will approach 840,000 rising to 1.2 million by 2050 (1). On*e third of people aged over 65 in the UK will die whilst suffering from dementia* (2). Systematic reviews suggest people with dementia have significantly increased mortality rates (3); even minor cognitive impairment is a strong independent predictor of mortality (4).

The clinical picture

People with severe memory problems can be identified using the Functional Assessment Staging Scale (FAST)(5). At level 6a and above the person will have difficulty putting clothing on properly without assistance, may have difficulty bathing properly, have urinary incontinence, be doubly incontinent or speak only a few words. A retrospective UK study of symptoms experienced in the last year of life by people with severe memory problems compared to cancer patients showed that the symptom burden and health care needs were comparable. In particular, 64% of those with severe memory problems experienced pain (compared to 59% with cancer), 46% breathing difficulties, 39% pressure sores and 86% difficulty with swallowing or loss of appetite (6;7). In people with severe memory problems acute physical illness may be an indicator of imminent death; 24% of those with moderate/severe dementia die after acute unplanned medical admissions compared to 7.5% of those without dementia (8).

Challenges

Essential components of good end of life care are often neglected in people with severe memory problems and referral to palliative care is rare (9) with fewer than 1% of hospice patients in Europe having a neurological diagnosis (10). In people with severe memory problems there are concerns about prognostic uncertainty and whether hospice staff can manage behavioural problems or communication difficulties (11;12);however, most symptoms experienced at the end of life such as pain or difficulties swallowing can be managed with good generalist care (13). Providing care in the usual place of residence is a major aim of the UK Government's End of Life Care Strategy; as well as benefitting patients and family carers this aims to save NHS costs by avoiding acute hospital admissions (13). A recent National Audit Office report indicated that about 50% of care home residents who died in hospital could have died within the care home setting (14). Evidence on how to improve care is limited. Based on available evidence, systematic reviews suggest the need for "care" tends to focus on specific interventions such as pain control, or the withdrawal of aspects of care e.g. *not* prescribing antibiotics (15;16). We suggest that good care requires a broader (but cost effective) palliative approach, tailored to meet the symptoms experienced by those with severe memory problems and also to meet the needs of family carers, particularly in the

terminal phase and in bereavement. Our work responds to UK government initiatives for care in dementia and at the end of life (13;17).

DEVELOPMENT OF THE COMPASSION MODEL OF ENHANCED CARE

We have used a realistic evaluation framework to develop the intervention, which incorporates information from a wide range of locations and sources. Improving end of life care is a complex undertaking. Our approach acknowledges the importance of context and social processes and allows us to find out about what mechanisms work, in what conditions, why, and how these produce particular outcomes. In brief, our findings so far have informed the enhanced model of care:

Work stream 1

In work stream 1 we conducted detailed research to define the symptom burden and needs of people with severe memory problems at the end of life, and their family carers. We have undertaken a longitudinal cohort study and have recruited 61 people with severe memory problems (FAST stage 7a and above, doubly incontinent and speaks only 5-6 words per day), 57 residing in care homes and four in their own homes. We have also recruited 26 of their family carers. Results from these studies showed how people with severe memory problems have multiple unmet needs, particularly with regards to management of pain and agitation. They are at high risk of pressure sores and have problems with eating and swallowing. There is lack of individual care planning and consideration of end of life care needs.

Work stream 2

Workshops with health and social care professionals

In work stream 2 in a first cycle of workshops we included a wide range of stakeholders and participants at all levels of responsibility. We conducted two workshops in London and one in each of Edinburgh, Solihull and Belfast. We used clinical vignettes describing people with severe memory problems and asked participants to consider how their care could be enhanced to provide solutions to the issues described.

In a second round of workshops we enhanced the content and face validity of our intervention, by using the RAND/UCLA approach (18). A key aspect of this approach is the Rand Appropriateness Method (RAM) which was used as a way to agree the key components of the intervention. To ensure that we took proper account of context further workshops were held across the four countries of the UK (sites in London, Edinburgh, Solihull, Belfast and Penarth). Before each workshop an online process managed by Survey Monkey, asked stakeholders to rank, for appropriateness, statements describing possible intervention components that were derived from the first round of workshops. Results were then analysed before each workshop and any points of disagreement were discussed further in the

workshop. Following this, participants were asked to rank statements describing components for necessity but independent of economic considerations. Data from all the workshops were pooled and a final bank of statements describing potential components of the enhanced model of care derived.

Interactive qualitative interviews with family carers and healthcare professionals

We conducted individual interactive interviews with 14 family carers and 14 health care professionals from a wide range of stakeholder sources including commissioners and health care assistants. Data analysis is on-going.

Workshops with family carers and people with early dementia

We conducted one workshop with five people with early dementia. We asked them to consider the type of care they would want in the future, especially towards the end of their lives. We also held a workshop with five family carers of people with severe memory problems. They were asked to suggest ways that care could be improved particularly considering end of life care planning and their own experiences of difficulties associated with the transfer of the person with severe memory problems to the acute hospital.

Policy documents

We undertook a detailed review of key documents currently operational in the four countries of the UK. We have focussed on documents that have been published since the National End of Life Care Strategy (2008) and Living Well with Dementia: a national dementia strategy (2009). Using a standardised template, we have summarised key statements arising and looked for similarities and differences in health and social care delivery across the four nations.

Synthesis of findings and development of the enhanced model of care

Findings from the cohort study workshop and interview data suggested a number of issues and ways that care could be improved, for example;

1. Importance of context: considerable regional variation in health and social care organisation and policy within the countries of the UK and Northern Ireland/ detailed repository of policy documents will be used to inform the reporting of our qualitative data and provide context for our recommendations.
2. Training for paid carers at the end of life, learning from hospice model
3. Training for paid carers on difficult conversations and care planning with family carers.
4. Improved staff skills and confidence/more trained nurses in ratio to health care assistants, a medical model like hospice care.
5. Need for enhanced bereavement support for paid and family carers including reflection on the death and care provided
6. Issues in care home culture/ prevent fear of deaths occurring

7. Lack of engagement of palliative care team/more education on dying with severe memory problems
8. Referrals and multi-disciplinary team working/ single point of contact, continuity of general practitioner (GP) care, rotating staff across environments to bring new learning, out of hours care from GP's who know patients.

The likelihood of successful implementation of our new enhanced care model requires that we understand the sociological theory underlying how our intervention would operate in practice (19). Following the RAM process, we scrutinised retained intervention components and mapped them to the theories described by Grol (19), categorising them according to which of the four operational levels identified by Ferlie and Shortell (20)and others such as Greenhalgh (21). We thought the components might operate on; 1) individual, 2) team, 3) group and 4) system levels. We explored both impact and process theories, operational and utilisation plans at the levels of the individual, social interaction, organisational context and economic/political context.

Details of the enhanced model of care for piloting are presented below (page 16 and Appendix 1).

AIMS AND OBJECTIVES OF PILOT STUDY

Our aim is to conduct a naturalistic pilot study to understand how the Compassion enhanced model of care operates in practice in two care homes in two different health and social care economies; one in the Camden Commissioning Group and one in the Barnet Commissioning Group.

Objectives

In the pilot study we will provide a coordinator with clinical skills- an "Interdisciplinary Care Leader (ICL)" who will coordinate and support the existing team of health and social care professionals working with participating care homes to enhance the management of people with severe memory problems. Our objectives will be met by collecting both quantitative data and qualitative data from the enhanced care team, care home staff and family caregivers

Specific objectives of the pilot study will be to:

1. Understand whether the enhanced model of care is feasible in the setting

2. Determine whether the enhanced model of care is acceptable to staff and family carers of people with severe memory problems in the care home

3. Understand facilitators and barriers to the implementation of the enhanced model of care by collecting qualitative data from paid and family carers on the experience of the intervention

4. Evaluate whether the enhanced model of care has an impact on a range of national key performance indicators and outcomes including those operating at a number of levels:
    a. Enhanced care team
    b. Care home environment and management
    c. Care home staff
    d. Family carers
    e. Residents with severe memory problems
5. Attempt to describe in detail the costs of delivering the intervention at our pilot sites and the costs of each of its sub-components to inform the commissioning process. These costs can be set against potential benefits and recommendations made

RECRUITMENT AND CONSENT PROCEDURES

Location

Through our previous cohort study we have worked with care homes in the Camden and Barnet Commissioning Group areas. We have chosen these as sites for our pilot intervention because we have previous experience of working with local clinicians including GPs and palliative care teams and they represent different location in terms of the socioeconomic and demographic composition of the area.

Recruitment of care homes

After gaining ethical consent for the study we will approach each care home manager by sending them a letter with brief study details. If the manager is interested, senior study staff will then visit the care home and provide further information regarding the project. We will, at the same time, also approach the proprietor or owner of the care home with similar information and seek their written consent for the home to participate in the enhanced care service and the collection of data from the home for the project outcomes.

Consent for implementing the enhanced model of care within the care home

We will be implementing our intervention of the enhanced care model at the level of the care home; our study can therefore be defined as a cluster pilot evaluation. The model of individual informed consent (or nominee assent) to receive the intervention may not be appropriate for a number of reasons. Firstly we are working with existing clinical services to offer an enhancement of usual care which is in line with the recent English Government Dementia and End of life care strategies. Secondly, we will be training and supporting the existing team to enhance and optimise practice, and thus may influence the care of all residents of the home.

We have consulted the UK Medical Research Council Guidance Document “Cluster randomised trials: methodological and ethical considerations.” Using this framework our intervention is designated as “type A”- interventions that are received (or not) by a whole cluster together so that there is only one decision to be made for the care home. Therefore we use the appropriate Cluster Representation Mechanism (CRM), in our case, the nursing home owners who will give their consent for the intervention to be implemented in their care home. We will also obtain the permission of an ethics committee to implement the enhanced model of care so that the project undergoes appropriate ethical scrutiny. Some evaluation data will be collected at the individual level from the care home and these data will be anonymised, and therefore managers will not be providing any individually identifiable participant data.

Where we will be collecting individual level data, i.e. the qualitative evaluation, resident quality of life and measures from nursing home staff and family carers, we will obtain individual informed consent to participate. We will document how many participants who are approached do consent to us collecting individual level data as this may inform the planning of our future work.

Informing participants about the study

After gaining ethical consent to implement the enhanced care model the research team will meet with care home staff to inform them of the study and to answer or discuss their queries or concerns regarding the study.

Recruitment of people with severe memory problems for evaluation of outcomes

To collect evaluation data we will aim to recruit as many eligible residents as possible from each participating care home. Our criteria have been developed from an existing NHS and Social Care enhanced model of care from South London which has been used by the King’s Fund as an example of UK best practice:

Resident Inclusion criteria

1. Aged over 65 years.
2. Severe memory problems indicating a clinical diagnosis of DSM-IV criteria for dementia (22).
3. Moderately severe or severe memory problems as classified on the Functional Assessment Staging Scale (FAST) grade 6a and above (5) see Table 2.

   Plus at least one of the following criteria:

- There are recurrent infections, significant weight loss and poor nutrition level, recurrent fevers, pains, falls, severe pressure ulcers that are not easily amenable to treatment, severe physical frailty.

- OR, the resident has severe, persistent distress (mental or physical) that is not easily amenable to treatment OR another condition (eg. co-morbid cancer) whose co-existence with dementia means that more intrusive treatments would be less appropriate.

Resident Exclusion criteria

- Residents who indicate either verbally or non-verbally that they do not wish to participate.

- Residents who are moribund, in a coma, or those where there are clinical concerns that may preclude them being approached.

Table 2: Functional Assessment Staging Scale (FAST)

STAGE	Description of functions lost
1	No difficulties, either subjectively or objectively
2	Complains of forgetting location of objects. Subjective word finding difficulties.
3	Decreased job functioning evident to co-workers; difficulty in traveling to new locations. Decreased organisational capacity.*
4	Decreased ability to perform complex tasks (e.g. planning dinner for guests, handling personal finances, difficulty marketing etc.)
5	Requires assistance in choosing proper clothing to wear for the day, season or occasion.
6a	Difficulty putting clothing on properly without assistance.
6b	Unable to bathe properly; e.g., difficulty adjusting bath water temperature) occasionally or more frequently over the past weeks.*
6c	Inability to handle mechanics of toileting (e.g., forgets to flush toilet, does not wipe properly or properly dispose of toilet tissue) occasionally or more frequently over the past weeks.*
6d	Urinary incontinence, occasional or more frequent.
6e	Faecal incontinence, (occasional or more frequently over the past week).
7a	Ability to speak limited to approximately a half dozen different words or fewer, in the course of an average day or in the course of an intensive interview.
7b	Speech ability limited to the use of a single intelligible word in an average day or in the course of an interview (the person may repeat the word over and over).
7c	Ambulatory ability lost (cannot walk without personal assistance).
7d	Ability to sit up without assistance lost (e.g., the individual will fall over if there are no lateral rests [arms] on the chair).
7e	Loss of the ability to smile.
7f	Loss of ability to hold up head independently.

*scored primarily on the basis of information obtained from a knowledgeable informant and/or caregiver.

Consent Procedures

Potential resident participants will have severe memory problems and may be physically frail. It is likely that they may not have the capacity to consent. Therefore our procedure has been developed to comply with capacity legislation governing England and Wales (Mental Capacity Act 2005, Sections 30-34) (see Figure 1).

Residents in care homes with severe memory problems

1. Although it is unlikely that any residents with severe memory problems will have capacity to give consent to participate in the study, The Mental Capacity act requires that we assume a person has this, unless shown otherwise. If the resident has capacity to consent to participate in the data collection, the care home manager will ask the resident if they are willing to see a member of the research team who will then consent them into the study. If capacity is not present the following steps will be taken.

2. On our behalf, the care home manager will attempt to identify their next of kin, family carer or someone close to the person (who does not receive remuneration for this role) who will act as a "personal consultee".

3. If the personal consultee is visiting the care home they will be approached by the care home manager and given verbal information and a written information sheet about the study. They will be encouraged to consider the person's prior wishes or thoughts regarding taking part in research. They will be asked to sign and return a reply slip indicating if they give consent for their contact details to be passed to the research team. If no reply slip is returned to the research team within 14 days, the research team will contact the care home to inform them of this. The care home will then contact the family carer only once and ask if they agree to the home giving the research team their contact details so the research team can contact them regarding the study. If the family carer does not give permission for the care home to give their details to the research team, no further contact will be made. If permission is granted a member of the research team will telephone the family carer. If the personal consultee agrees to the person taking part they will be sent an information sheet and a family carer assent form to sign or be invited to visit the care home and meet with the research team to do this in person. If no assent form is returned within 14 days then the research team will telephone the personal consultee on the maximum of two occasions to see whether they are still interested in participating.

4. If the personal consultee is not available in the care home (i.e. lives a distance from the home or is not able (or wishes) to visit) the care home manager will post the study information sheet to them. They will also be sent a reply slip to sign and return on whether they give permission for the care home to pass their contact details onto the research team. If no reply slip is returned to the research team within 14 days the research team will contact the care home to inform them of this. The care home will contact the family carer only once and ask if they agree to the home giving the research

team their contact details so the research team can contact them regarding the study. If the family carer does not give permission for the care home to give their details to the research team, no further contact will be made. If permission is granted a member of the research team will telephone the family carer. If the consultee agrees to the person taking part they will be sent a family carer assent form to sign or invited to visit the care home to meet with the research team to do this in person. If no assent form is returned within 14 days then the research team will telephone the personal consultee to see whether they are still interested in participating.

5. If a) no friend or next of kin that can act as a personal consultee is documented in the clinical notes, or, b) after three attempts at telephone contact over one week by the care home manager, they are unable to contact a personal consultee, then the research team will use a professional consultee. This will be defined as a senior experienced health or social care worker who is not directly involved in the research or care of the patient. Through the cohort study we have identified skilled professionals within each CCG who are not involved in the research project or in the patient's direct clinical care and are happy to act in this role. These "consultees" will be given information about the study and training on their responsibilities by the research team. They will follow a structured procedure to give assent for the person's participation in the study and sign their assent for this.

Figure 1. Summary of consent procedures for the collection of individual level outcome data

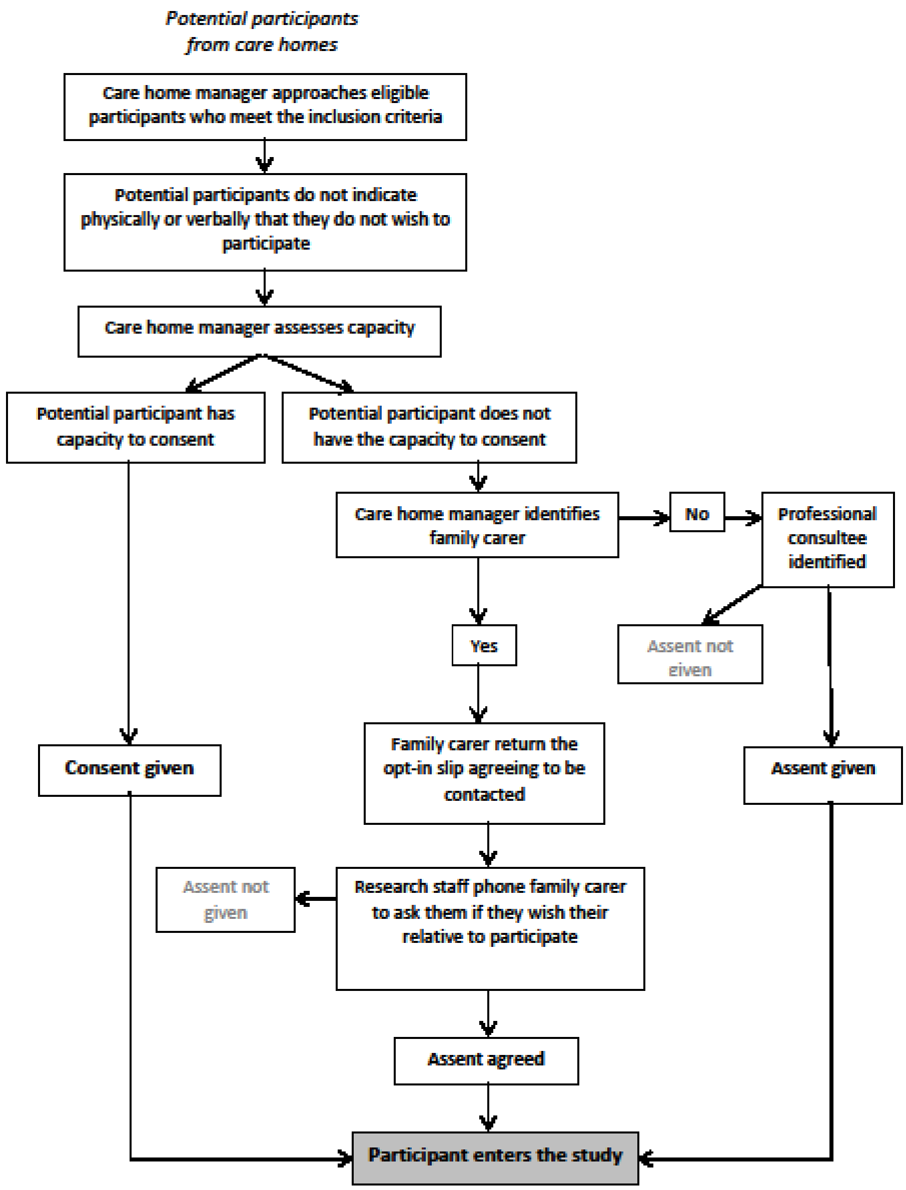

Recruitment of family carers to give information for the evaluation

We wish to evaluate the opinions of family carers of residents with severe memory problems who have received the enhanced care service. We will only recruit carers of people with severe memory problems who have already entered the study as the recruitment of dyads will enable us to link the experiences of people with severe memory problems and their family carers.

Family carer inclusion criteria

- If the resident with severe memory problems does not have capacity this will be the main family carer (e.g. family member or friend in regular contact and who is the next of kin or a 'key decision maker', identified by the care home manager). If the resident does have capacity we will ask them to nominate who they think is their family carer.
- English language sufficient to complete the study ratings.

Family carer exclusion criteria

- Family carers where there are clinical concerns that may preclude them being approached.
- Family carers aged 16 and under.
- If for any reason during the study the family carer becomes unavailable/unable to give consent we will withdraw the family carer from the study.

Consent procedure

Family carers of residents who do not have capacity to consent will be asked if they wish to participate when we recruit their relative/friend into the study. We will explain that we are interested in exploring their experiences of the enhanced care service now and, should the person die, their experiences of bereavement. They will be informed that they will have two weeks to decide whether they want to participate and can, if they wish, take time to discuss the study further, with other family members/friends, GP and/or research staff. They will be informed that if they decide not to take part that this will not adversely affect the care of their friend/relative or the support they receive as a family carer in any way. If the family carer agrees to participate then a consent form will be sent to them (or given to them when we see them face to face). If the consent form is not returned within 7 days we will contact them again to check whether they still wish to participate. There will be a maximum of two attempts to contact.

Where the resident does have capacity to consent for themselves we will need to recruit family carers independently. The care home manager will approach the family carer and given verbal information and a written information sheet about the study. They will be asked to sign and return a reply slip indicating if they give consent for their contact details to be

passed to the research team. If no reply slip is returned to the research team within 14 days, the research team will contact the care home to inform them of this. The care home will then contact the family carer only once and ask if they agree to the home giving the research team their contact details so the research team can contact the family carer regarding the study. If the family carer does not give permission for the care home to give their details to the research team, no further contact will be made. If permission is granted, a member of the research team will telephone the family carer. If they agree to participate they will be sent an information sheet and a consent form to sign or be invited to visit the care home and meet with the research team to do this in person. If no consent form is returned within 14 days then the research team will telephone the family carer on the maximum of two occasions to see whether they are still interested in participating.

If the family carer is not available in the care home (i.e. lives a distance from the home or is not able (or wishes) to visit) the care home manager will post the study information sheet to them. They will also be sent a reply slip to sign and return on whether they give permission for the care home to pass their contact details onto the research team. If no reply slip is returned to the research team within 14 days the research team will contact the care home to inform them of this. The care home will contact the family carer only once and ask if they agree to the home giving the research team their contact details so the research team can contact them regarding the study. If the family carer does not give permission for the care home to give their details to the research team, no further contact will be made. If permission is granted a member of the research team will telephone the family carer. If they agree to take part they will be sent a consent form to sign or invited to visit the care home to meet with the research team to do this in person. If no consent form is returned within 14 days then the research team will telephone the family carer to see whether they are still interested in participating.

Recruitment of enhanced care team and care home staff to participate in qualitative interviews

Recruitment and consent of heath care professionals and paid carers

The Interdisciplinary care leader (ICL)/care home manager will identify Healthcare Professionals (HCPs) and paid carers who have been involved in providing care and support to people with severe memory problems in the care home; this will include those from a variety of disciplines and organisations who go into the care home for example, care home staff, general practitioners, speech and language therapists, social workers etc. They will be asked whether they are interested in participating in the research and whether they are happy if the research staff can be given their contact number at work. The researcher will then contact them to discuss the study in detail.

The research team at the research site will ask if they are interested in participating, provide them with an information sheet and ask if they would be happy to participate. They will have

at least 48 hours to consider whether they wish to participate. They will be informed that their participation is voluntary and individuals or their organisation will not be identifiable in anyway and that all information will be anonymised and kept confidential. If the HCP/paid carer decides they do wish to take part in the study they will be asked to sign a consent form. We intend to conduct a maximum of 10 interviews per care home.

Potential risks/strengths

A strength of our approach is that we have developed our intervention using information gathered from a range of participants. These include health and social care staff, people with early dementia and their family and other unpaid carers. The intervention is also an enhancement of usual care which merely formalises recommendations made in exiting policy documents such as the English National Dementia and End of Life Care Strategies. It is being run in conjunction with established clinical services, adding to their capacity to manage and improve the care of people with severe memory problems who reside in a care home. It will not inhibit the “usual care” that they should receive and **clinical responsibility for the resident’s care will, as per usual practice, remain with their GP**. The measures we use to evaluate outcomes are mostly observational with no additional burden or discomfort to the patient and should be part of good routine end of life care (23) therefore the risk of any harm is minimal. If the person with severe memory problems does become upset or uncomfortable in any way with the assessment process, the researcher will stop the assessment immediately and report this to the care home staff and/or the resident’s family carer.

We do understand that this research may touch on some sensitive issues for family carers and paid staff, however, the Marie Curie Palliative Care Research Unit has extensive experience of conducting interviews with bereaved relatives of patients with malignant and non-malignant conditions, including end-stage renal disease and advanced dementia (24-27).

In the unlikely event that family carers do become upset in taking part in the study, the researcher will stop the assessment. They will with the family carer’s permission ask them if they want to have a break from the assessment, continue or to stop. It is natural that family carers may at times feel emotional when talking about their role or their relative/friend. If the family carer wishes to stop then the assessment will be brought to a close. If they become upset or if their scores on the Hospital Anxiety and Depression Scale (HADS) scale suggest clinical depression or anxiety they will be given information regarding support networks/agencies to contact should they wish, for example, the Admiral Nurse DIRECT or Alzheimer’s Society National Help lines, their General Practitioner or other relevant service if there is prior involvement.

The research staff collecting data will be given training and supervision on all of the study assessment tools and family carer interview schedule. The research team will review their recruitment procedures after one month. Any problems will be documented. If substantial

changes to the protocol are needed we will seek approval of proposed changes from the Research Ethics Committee.

If we discover issues of malpractice, maltreatment or serious neglect, to the degree that the relevant local authority's safeguarding procedures are triggered, we will in this circumstance be required to break patient confidentiality and inform the relevant authorities, following whichever standard local authority safeguarding procedures are in operation.

It is important that issues of sustainability are considered so that we do not leave the care home unsupported after the enhanced care pilot has finished. Evidence suggests that even after the research team have finished the pilot, benefits may persist and that local services maintain and further develop new interventions , so maintaining on going improvements in care; "dynamic sustainability" (28) . One aspect to sustain any benefits is that participating nursing homes will be provided with a structured training programme designed to meet any training/care needs identified during the cohort study.

PROJECT INTERVENTION

Preparing the Compassion Intervention manual for the enhanced model of care

We have produced a written document to describe Compassion in manual form as recommended by the Medical Research Council (MRC) guidance on the development of complex healthcare interventions 2008. This provides a framework by which the intervention can be sustained and becomes replicable at a number of sites. It describes for participating partners the core intervention components and the steps required to implement components. There are two core components:

1. Facilitation of integrated care for people with severe memory problems and their family carers.

2. Education, training and support for health and social care professionals at all levels and for family carers.

The manual in its development was reviewed by key stakeholders during a focus group (care home managers, representatives from palliative care, GPs and care of the elderly physicians). Necessary changes were made, and further amendments were made by the programme grant expert steering group.

The manual describes in detail processes which aim to improve end of life care for people with severe memory problems by:

- Enabling holistic individualised person centred care.

- Providing an interdisciplinary care leader (ICL) who will act as a central resource for health care professionals, care home staff and family carers involved in the care of people with severe memory problems.
- Developing links and joint working between all those involved in the care and management of people with severe memory problems to establish a model of integrated care.
- Improving the understanding of what is meant by an individualised personal care plan and how such a plan might be worked out and used in practice
- Providing support to front-line staff and managers in care homes to enable them to hold uncertainty and manage risk in people with severe memory problems to avoid unnecessary place of care transfers.
- Identifying, facilitating and supporting the training needs of care home staff in the care of those with severe memory problems.
- Recognising the needs of family carers, including being alert to possible anxiety and depression.
- Supporting the commissioning of effective and sustainable systems to deliver these objectives.

Overview of the intervention

The enhanced model of care delivered by the intervention will run for 6 months. For a detailed description of the intervention see Appendix 1. Facilitating effective clinical change in complex health and social care systems can be challenging. Compassion aims to set out a clear pathway of the actions that need to be taken, and by whom, for its effective implementation. This includes integrating change within existing systems to underpin current expertise and developing an understanding of what is needed for continued best practice. The key people involved in delivering Compassion for the pilot phase are listed below.

Interdisciplinary Care Leader (ICL)

The ICL will be a new post funded through the Compassion research project. The main responsibilities of the ICL will include:

- Developing an understanding of the health and social care professionals, pathways and services relevant to the care home residents with severe memory problems that are currently available.
- Working with the care home staff to identify and assess residents suitable for inclusion in the intervention.
- Establishing who the members of the core team involved in care will be, co-ordinating the weekly meetings and working within the core team to develop and implement personalised care plans for each resident included in the intervention.

- Establishing the wider clinical team, co-ordinating monthly meetings and maintaining effective communication to facilitate integrated co-ordination of care and the development of good working relationships between all health and social care professionals involved in the care of those with severe memory problems.
- Working with the care home staff to identify and support their educational and training needs, including fostering a culture of respect, dignity and quality of care for all residents and their family carers supporting someone with severe memory problems.
- Meeting with and supporting family carers to ensure their needs and wishes are understood.
- Collecting process data to support evaluation of the intervention.

The ICL will receive training in standard procedures with regard to clinical and information governance, safeguarding of vulnerable adults and the Mental Capacity Act prior to commencing in post. He/she will keep an anonymised reflective diary and will be supported by the research team at the Marie Curie Palliative Care Research Unit.

The Core Team

The core team comprises a range of existing staff who already regularly visit the homes and are responsible for overseeing the medical, nursing and social care needs of residents. During the intervention they will work with the ICL and are the key personnel required to deliver Compassion. The team will meet weekly and includes:

- Clinical Lead Professional (GP supporting the care home, Geriatrician or Old Age Psychiatrist)
- Member of care home staff (care home manager or floor/ unit manager)
- Interdisciplinary Care Leader

The Wider Team

The wider team includes local health and social care professionals and specialist services involved in the care of people with severe memory problems. The team includes staff from General Practice, Care of the Elderly, Old Age Psychiatrist, Palliative Care, Social Services and Community services such as District Nursing, Social Workers, Speech and Language Therapy, Dietetics, Tissue Viability, Physiotherapy and Occupational Therapy. However, the exact composition will depend on local working practices and the availability of key personnel. The wider care team will meet monthly with the core team; meetings may be face to face or via links such as conference calling. The organisation, communication, facilitation and recording of meetings will be the responsibility of the ICL but the team will be required to appoint a lead to chair the meetings.

The Compassion flow chart, shown below, outlines the steps of the intervention pathway, the roles and the responsibilities of those participating, and the work required within each step of the pathway.

Compassion **Intervention flow chart for pilot study**

Step 1: Engage local stakeholders and analyse local need, interest and readiness for change. Obtain necessary permissions (project team)

↓

Step 2: Map existing services (ICL and project team)

↓

Step 3: Identify Clinical Lead Professional and establish membership of the core and wider team. Hold initial orientation meeting (ICL and project team)

↓

Step 4: ICL works alongside care home staff to scope, identify and respond to training needs

↓

Step 5: ICL and care home staff identify residents who may benefit from the intervention

↓

Step 6: ICL and care home staff conduct initial assessment of care needs

↓

Step 7: ICL and care home staff discuss care needs and concerns with family carer and assess carer needs

↓

Step 8: Assessment of care needs discussed at weekly core team meeting with clinical lead and care plan agreed

↓

Step 9: ICL discusses and implements the care plan with the care home staff and family carer (if available)
ICL meets with night staff as appropriate to plan for possible out of hours events

↓

Step 10; ICL reviews residents with care home staff at regular intervals or after sentinel events
ICL reports back to core team and family carer, care plan reviewed and amended if necessary

↓

Step 11: Wider team meet monthly to discuss complex cases and review care plans
Wider team consider significant events, critical incident analysis (cyclical performance review)

↓

Step 12: After death family carer seen by ICL and signposted to additional support as required
Death is reviewed in wider team meeting

Ongoing training and facilitation model (running alongside Steps 3–12)

Education and Training considerations

Current education and training provision on end of life care in for people with severe memory problems within the CCG area will be scoped and mapped.

The ICL will work with the care home to help to establish and address the training and educational needs of their staff. This will be primarily by working alongside the staff but may also include one to one reflective discussions with key staff members. Learning and training needs will be addressed in a variety of ways but will include shared working and mentoring, use of online learning resources and formal topic based teaching sessions from local services

and experts if required. Training will be feasible within timing, staffing and financial constraints and will be agreed with the care home manager.

Education and training provided as part of the intervention will aim to enable care staff to recognise and respond effectively to the needs of people with severe memory problems and to support family carers with increased confidence and competence. Education and training will link to the core competencies outlined in the document "Developing end of life care practice: A guide to workforce development to support social care and health workers to apply the common core principles and competences for end of life care" (Skills for Care, Skills for Health, National End of Life Care Programme. 2012) and will include, communication skills, with residents suffering from severe memory problems and their family carers, assessment and care planning, advance care planning, symptom management to maintain comfort and wellbeing, knowledge and values.

DATA COLLECTION

Our enhanced care model may have an impact at a number of levels, for example on the individual resident and their family carer, on care home staff, at processes which occur at the level of care home management and on the intervention team itself. This is a feasibility study and thus we have to collect data on a range of outcome and process measures, to detect any impacts which the intervention may have on a complex care system and those who reside and work within it. Our measures map onto our key objectives which are to understand the barriers and facilitators to the implementation of the enhanced care model, to assess feasibility and acceptability of the model and to understand the impact of this model on individual residents and their family carers. Data collection is summarised in table 3 (below).

Process data: these will be collected by the ICL and the team delivering the enhanced service. It is evaluation data much of which is already routinely collected within this setting and is required for national NHS and social care end of life care targets and key commissioning performance indicators (marked with * in outcomes table). The data will give us information on the feasibility and acceptability of the intervention and barriers and facilitators to its implementation. This data will be anonymous at source and not collected at an individual level.

Data on individual outcomes: these data will be collected by the research team who will work independently of the enhanced service implementation team. We will collect data from residents with severe memory problems who receive the service, their family carers, individual care home staff and individual members of the intervention team. Thus to collect these data will require individual informed consent (or in the case of care home residents who may lack capacity, assent). For further information in our consent processes please see page 8.

Table 3: Process and outcomes measures

	Process data	Information on individual outcomes and perspectives
**Enhanced care team**	• Number of residents reviewed<br>• Contacts with family carers<br>• Attendance at team meetings<br>• Number of individual care plans made*<br>• Referral to other specialists	• Experience of participating in the enhanced care intervention<br>• Experience of participating in the enhanced care intervention<br>• ICL reflective practice diary<br>• Barriers and facilitators to the enhanced care intervention
**Care home level data**	• Use of pain tools *<br>• Number of residents with pain management plans*<br>• Recording of surrogate decision makers*<br>• Number of residents with resuscitation status recorded*<br>• Number of deaths within the care home in the last month*<br>• Recording of preferred place of death*<br>• Number of deaths in the usual/preferred place of care*<br>• Numbers of ambulance transfers to acute care*<br>• Visits by out of hours primary care*	
**Care home staff**	• Education and training needs of care home staff and how these were addressed	• Experience of participating in the enhanced care intervention
**Family carer**	• Numbers who have a needs assessment	• Satisfaction with the intervention<br>• Burden<br>• Anxiety and depression<br>• Satisfaction with general care<br>• Quality of life<br><br>If the resident dies:<br>• Satisfaction/quality of end of life care
**Care home resident**	• Number of baseline assessments<br>• Number of review assessments	• Severity of impairment<br>• Pressure sores risk and severity<br>• Pain<br>• Agitation<br>• Behavioural Symptoms<br>• Symptom management at end of

		life<br>• Quality of life<br>• Resource Utilisation<br>• Number of hospital admissions<br>• Sentinel events<br>• Use of parenteral feeding<br>• Use of personalised care plans<br>• Death in usual /preferred place of care<br><br>If the resident dies:<br>• Use of medication<br>• Burdensome interventions<br>• Adherence to individual care plan

*evaluation data which is already routinely collected within this setting and is required for national NHS and social care end of life care targets and key commissioning performance indicators

Enhanced care team process data

The ICL will record process data on a pro-forma to enable monitoring and evaluation of the enhanced service. These data will be collected on a monthly basis, it will be anonymised and not identifiable at the level of individual residents. These data give us information on the feasibility and acceptability of the intervention and will include:

- Number of residents reviewed by the enhanced care team
- Number of contacts with family carers (by phone and face to face)
- Attendance at team meetings
- Number of individual care plans made by the enhanced care team
- Referral to other specialists outside the care home, for example dietician, speech and language therapists, tissue viability nurses
- Education and training needs of care home staff and how these were addressed i.e., by individual training sessions, referral to online training resources

In addition the ICL will keep a reflective diary (carefully written to ensure anonymisation and confidentiality) recording their experiences of scoping for and implementing the intervention, including notes on care home dynamics, their interactions with the core and wider teams, the care being delivered by staff and any changes being observed that may not be captured by the outcome measures.

Care home level process data

This data will be collected on a monthly basis by the care home manager (to comply with the UK Data Protection Act 1998) in collaboration with the ICL. It will be anonymised and not identifiable at the level of individual residents. Much of this data should already be routinely

collected and is required by governance organisations and local health and social care commissioners in their assessment of whether services are meeting statutory key performance indicators. The ICL will document:

- Whether pain tools are being routinely used in the care home
- The number of residents with pain management plans
- The recording of surrogate decision makers in the care home records
- Number of residents with resuscitation status recorded
- Number of deaths within the care home in the last month
- Recording of preferred place of death
- Number of deaths in the usual/preferred place of care
- Numbers of ambulance transfers to acute care
- Visits by out of hours primary care

Acceptability of the intervention to care home and enhanced care team staff

We will explore the experience of participating in the intervention with members of the enhanced care team and care home staff. We will conduct qualitative interviews with a purposively sampled selection of staff at the end of the project. These interviews will occur at the end of the intervention period. We will explore the staff experience of the enhanced care team using a structured topic guide which maps onto key areas of current UK end of life and social care policy, for example, how they found working with the ICL, whether the ICL enhanced the way they performed their role, whether the enhanced care model changed how they recognised symptoms such as pain and how these were managed (for interview guide see Appendix 2). Interviews will be audio taped and transcribed verbatim (anonymised). They will last no longer than one hour and participants will be offered the opportunity to review transcripts to ensure accuracy.

Outcomes for care home residents receiving the intervention

These data will be collected independently by the research team only on those residents who have given informed consent to participate or whose relatives have given signed assent for their participation

Demographic information (age, marital status, previous employment) will be collected at the beginning of the evaluation. Severity of dementia will be measured using the FAST scale. At study entry information from GP notes will be obtained by the research team, including: medical co-morbidity (the Charlson Co-morbidity Index (CCI): which includes 19 diseases weighted on the basis of their association with mortality). This allows for the documentation of painful co-morbidities (29).We will document medications from GP prescriptions (e.g. antibiotics, analgesia and antipsychotics). We will document the presence of advance directives, care plans and specific requests regarding hospitalization and resuscitation.

Clinical assessment

Researchers will assess participants and document their symptom burden with the proforma used in our cohort study (25). It consists of a typical, detailed generalist approach to palliative care.

Functional Assessment Staging Scale (FAST): This observational scale describes a continuum of seven successive stages of functional impairment, from normality to the most severe dementia (5). (See Table 2; ***Recruitment of people with severe memory problems for evaluation of outcomes***)

Bedford Alzheimer Nursing Scale (BANS): This brief 8-item scale is used to stage the level of severe memory impairment in terms of factors such as eye-contact and speech (30). (See Appendix 2).

Pressure sores risk and severity: The Waterlow Scale will be used for the assessment of risk for developing pressure sores (See Appendix 3). It has high inter-rater reliability and sensitivity (31). The Stirling Scale measures the extent of damage from a scale of 1, Non-blanching erythema of intact skin to 4, full-thickness wound, which involving subcutaneous tissue and the deep fascia (32). (See Appendix 4).

Observational scales completed with care home staff or family carers

Pain Assessment in Advanced Dementia (PAINAD): This measures pain during care tasks and at rest. A comprehensive systematic review has identified this tool as having sensitivity and clinical utility (33). (See Appendix 5).

Cohen Mansfield Agitation Inventory (CMAI): This observational scale rates a range of behaviours many of which are relevant and challenging in dementia, for example wandering, grabbing on to people and pushing. It enables measurements over short timescales and is completed with a carer or staff member (34). (See Appendix 6).

The Neuropsychiatric Inventory (NPI): is a brief caregiver questionnaire that is used to assess behavioural and psychological symptoms commonly observed in residents with severe memory problems (BPSD) i.e. psychosis, mood disturbances, agitation, personality changes, pacing, wandering, and appetite disturbances. Its use in primary care is recommended, as it not only assesses the severity of the symptom for the patient but also the distress that the symptom causes the caregiver (35). (See Appendix 7).

Symptom Management at the End of Life in Dementia Scale (SM-EOLD): Is a tool used to assess comfort and pain during the prior 30 days (36). (See Appendix 8).

The Quality of Life in Late Stage Dementia Scale (QUALID): is a validated scale that assesses quality of life over the prior week (37). (See Appendix 9).

Resource Utilisation in Dementia (RUD)-lite: Resource Utilisation in Dementia (RUD)-lite: Is a short version of the RUD structured interview to assess costs of care including patient accommodation, informal care, community care and hospitalizations (38). (See Appendix 10).

Monthly follow up assessments

Participating residents will be reviewed every four weeks in the care home by the research team, for a maximum of six months, or until death. We will repeat measures: the generalist clinical assessment; Waterlow, Sterling, CMAI, NPI, BANS, PAINAD, SM-EOLD, QUALID, and the RUD-lite. We shall also record prospectively the number of acute hospital admissions, the reasons for these, "burdensome interventions" e.g. enteral feeding tubes (27) and "sentinel events", defined as "new medical conditions that have the potential to lead to a significant change in health status and a shift in the goals of care" e.g. pneumonia, hip fracture (6). Prescription medications and use will also be collected.

Data collection post death

The Comfort Assessment in Dying with Dementia Scale (CAD-EOLD) (36) (See Appendix 11) will be completed with care home staff within 14 days of the resident's death to assess their level of comfort and pain in the seven days prior to their death. Through a review of care home notes we shall record use of medication at the end of life (i.e. "just in case" prescribing, opiates, syringe drivers and artificial hydration or nutrition), sentinel events and burdensome interventions. We will examine adherence to any individual care plans which were made.

Outcomes for family carers

Data will be collected independently by the research team during face to face interviews at study entry within 14 days of the initial resident assessment and then every month, by post or over the telephone (family carers' preference). If family carers are un-contactable for more than 2 months or withdraw from the study we will document the reason and aim to continue to include the person with severe memory problems in the study, unless the carer specifically withdraws their assent.

At project start

We will collect demographic data to include age, sex, ethnicity, education, employment and occupation (present or previous), marital status, relationship to the care home resident, the number of years spent caring and any other caring responsibilities e.g. children under 18 years of age.

At project start and each monthly follow-up

As with the participating residents, measures (listed below) will be repeated at monthly intervals. We shall inquire about contact with the ICL and whether end of life issues have been mentioned.

Zarit Burden Interview: a 22-item self-report questionnaire, the most consistently used measure of carer burden in dementia. The questionnaire asks the carer to reflect on how they feel when they are caring for the person (39). (See Appendix 12).

Hospital Anxiety and Depression Scale (HADS): a self-report instrument for clinically significant anxiety and depression (40). (See Appendix 13).

The Satisfaction with Care at the End of Life Scale in Dementia Scale **(SWC/CAD-EOLD):** a validated tool that quantifies overall satisfaction with care in advanced dementia. This brief 10-item self-administered questionnaire assesses the caregiver's level of satisfaction with decision-making, medical and nursing care, and their understanding of the condition of the person with dementia (See Appendix 14). The CAD version is used to assess care received around the time of death (36) (see data collection in bereavement - below). (See Appendix 11).

EQ-5D-5L: this instrument is an index-based utility set for the calculation of quality-adjusted life years (QALYs) used to inform health economic evaluations of healthcare interventions (41). (See Appendix 15).

Qualitative interviews

To gain a deeper understanding of how they experience the enhanced care model and working with the ICL we shall offer qualitative interviews with the research team and to all participating family carers in a place of their choice. These will occur at the end of the feasibility study for the enhanced model of care or in bereavement if the resident dies (for interview schedule see Appendices 16 and 17).

Data collection in bereavement

To gain a deeper understanding of the circumstances surrounding the death and the views of the carer on which aspects of care were or were not satisfactory, where possible we shall ask additional questions all bereaved family carers. In this case we will ensure these interviews take place two months after bereavement, this has been found to be the optimal time for such work whereby the carer feels ready to think about their loss but still has sufficient recall of events (42;43). We found in our cohort study that these interviews are acceptable (we have completed ten so far) and family carers are keen to reflect on their experiences (25). The SWC-EOLD scale will be completed to assess family carer's level of satisfaction with care

and the CAD-EOLD to assess the resident's level of comfort and pain in the 7 days prior to their death from the carer's perspective.

We will items from a topic guide similar to that used successfully in our cohort study which was acceptable to family carers (44). Interviews will be audio taped and transcribed verbatim (anonymised). They will last no longer than one hour and carers will be offered the opportunity to review transcripts to ensure accuracy.

Table 4: Summary of data collection

	Project start	During project (monthly for 6 months)	After death/ in bereavement	After project ends
Enhanced care team process data	**x**	**x**		
Care home level data	**x**	**x**		
Paid carers/ enhanced care team staff qualitative interviews				**x**
**Residents**				
Demographic information	**x**			
FAST scale	**x**			
Charlson Co-morbidity Index	**x**			
Medications	**x**	**x**		
Prior advance care plans and wishes documented	**x**			
Symptom burden/generalist clinical assessment	**x**	**x**		
Bedford Alzheimer Nursing Scale	**x**	**x**		
Pressure sore risk and severity	**x**	**x**		
Pain Assessment in Advanced Dementia	**x**	**x**		
Cohen Mansfield Agitation Inventory	**x**	**x**		
Neuropsychiatric inventory	**x**	**x**		
Symptom Management at the End of Life in Dementia Scale	**x**	**x**		
Quality of Life in Late dementia Scale	**x**	**x**		
Resource Utilization in Dementia Scale	**x**	**x**		
Burdensome interventions		**x**		
Sentinel events		**x**		
Comfort Assessment in Dying Scale			**x**	
**Family carers**				
Demographic data	**x**			
Zarit Burden Interview	**x**	**x**		

Hospital Anxiety and Depression Scale	**x**	**x**	**x**	
Satisfaction with Care at the End of Life in Dementia Scale	**x**	**x**	**x**	
Comfort Assessment in Dying Scale			**x**	
EQ-5D-5L	**x**	**x**	**x**	
Qualitative interviews			**x**	**x**

DATA ANALYSIS

Data will be collected at the start of the intervention, and at monthly time points until a resident dies or until the end of the intervention period (6 months). This will ensure a detailed understanding and, because of the mortality rates expected, minimize attrition. Data will be entered into a password protected anonymised database by the research team.

Quantitative analysis

We will use simple descriptive statistics to summarise process data and the outcomes collected by the ICL at the care home level (i.e. number of deaths in the last month etc.) We will describe the demographic and clinical characteristics of residents and family carers who participate in the data collection, as well as symptoms experienced, interventions received and any sentinel events. We will describe the symptom burden and quality of care received using SWEOLCD, QUALID. We will compare the scores to the results of our previous study in order to gain inferences on whether the enhanced care project makes a difference. The results will be summarised using mean and standard deviation or alternatives in case of non-normally distrusted data. Appropriate plots will also be produced.

Qualitative analysis

The interviews will be audio-taped, transcribed verbatim and entered onto a qualitative software programme (Atlas-ti) for the coding, management and retrieval of data. Transcripts will be analysed and coded using Thematic Analysis. The data analysis process will follow the guidelines provided by Braun and Clarke (45) to develop meaningful themes and a rigorous approach to data analysis will be adopted by working to the quality framework recommended by Spencer (46). Throughout the analytic process, the researchers will engage in ongoing reflection with the use of memoing and reflective diaries to engage with the data further and refine emergent themes. Data triangulation will be achieved by interviewing both family carers and care home staff from a variety of work roles (i.e., care home manager, health care assistant, nurse) to explore the facilitators and barriers to the implementation of the enhanced model of care from different perspectives.

Final analyses

After full data collection ends, we will undertake definitive analyses to detail the demographic features of the cohort and assess the symptom management and their health care needs (using Stirling, Waterlow, NPI and sentinel events), taking into account repeated measures on individual subjects. We shall describe the level and nature of unmet needs and examine descriptively (using mean and standard deviations or suitable alternatives in case of non-normally distributed data and graphs) how comfort and quality of life change over time (using PAINAD, SM-EOLD, SW-EOLD and QUALID). We will describe the trajectory of carer wellbeing (HADS and Zarit Buden Inteview) during their friend/relative's final stages of life with severe memory problems and how this may change if the resident dies, using plots of the of wellbeing over time.

Sample size

This is a pilot study and as such a formal power calculation is not appropriate. Numbers are chosen on pragmatic grounds as sufficient to demonstrate feasibility in terms of recruitment and acceptance of the intervention. We will aim to recruit 30 residents with severe memory problems from two care homes from which to collect individual outcome data.

Health economics

Health economic evaluation will consider resource allocation in caring for patients with severe problems and, where relevant, in their last 6 months of life, as well as the quality of life of their family carers and associated economic impact on these family carers in this period.

Data on resource and service use for people with severe memory problems (RUD-Lite) and economic burden on family carers (Zarit Burden Interview) will be collected both at baseline and monthly after the enhanced care project has been implemented. These data will be collated with unit costs data from *Unit Costs of Health and Social Care (2012)* (47;48) to obtain costs per patient from NHS (such as averted hospital admission, costs for a typical episode), costs from personal social services (such as training and education for care home staff) and costs from societal perspectives (such as local commissioners' decisions on scarce resource allocation, additional costs to public purse where caring responsibilities had been met by the state instead of family carers).

Economic evaluation of the quality-adjusted life years (QALYs) for family carers will utilize EQ-5D-5L instrument to assess if enhanced care project has resulted in greater utility attained for this group and associated cost-effectiveness.

PROJECT MANAGEMENT

Registration, sponsorship and indemnity

The project will be registered with the research departments at the participating CCG. University College London will be the project sponsor and provide insurance. The research team will obtain honorary clinical contracts for each participating CCG, adhering to the Marie Curie Palliative Care Research Unit's Lone Worker Policy (2012).

Data protection

Case Report Forms (CRF) for the study will be stored in accordance with the Declaration of Helsinki. Electronic data will be anonymised and stored on a password protected database. At the end of the study anonymised files will be stored securely in a secure UCL archiving facility.

Research network support

The programme has been adopted by the DeNDRoN (Dementias and Neurodegenerative Diseases Network)

Project Staffing

The person appointed to the ICL post will have extensive experience in the care of older people and their family carers in care home settings and with expertise in severe memory problems and social care. They will deliver the intervention with the core team. They will be supervised by the PI (Dr Louise Jones) and, given the nature of the work, offered supportive clinical supervision by Dr E Sampson. The ICL will receive training to acclimatise them to the care homes in which they will be working and familiarise them with the intervention manual. They will have a monthly meeting with the project team to check adherence to the principles of the manual and to make any necessary adaptations to this. Two clinical researchers, from the Marie Curie Palliative Care Research Unit, who have extensive clinical and research experience with both palliative care and people with severe memory problems and family carers will collect the individual data for the evaluation of the enhanced care intervention. The researchers have particular skills in interviewing bereaved family carers and relatives.

Core study team

Dr Louise Jones, Head of Unit, is PI and guarantor for the programme. She leads the Marie Curie palliative care research team at UCL. She is a palliative care physician and expert in qualitative and quantitative research in end of life care in a range of long term conditions. She has a long history of collaboration with other members of the team.

Dr Elizabeth Sampson is an international expert in end of life care research in dementia. She has expertise in epidemiology and old age psychiatry and leads the dementia research

group within the Marie Curie research team at UCL where she is deputy Head of Unit. She will lead this research programme and manage the research team.

Professor Michael King the director of the Division of Psychiatry at UCL, in which the Marie Curie Unit resides. He is co-director of PRIMENT Clinical Trials Unit which specialises in trials in mental health and primary care. He is expert in epidemiology, development and evaluation of complex health care interventions and clinical trials. He will provide expertise in particular for the development and testing of our intervention.

Professor Irwin Nazareth is professor of Primary Care and head of department of Primary Care and Population Health at UCL. He is co-director of PRIMENT Clinical Trials Unit. He is expert in epidemiology, development and testing of complex healthcare interventions.

Professor Stephen Morris is professor of Health Economics UCL. He is expert in economic evaluations of complex healthcare interventions and NHS databases and will provide expertise on health economics for all workstreams.

Professor Rumana Omar is professor in Biostatistics UCL and expert in analysing complex datasets where, because of the nature of the cohort under study, data may be missing.

Professor Gerard Leavey is a social scientist who is expert in qualitative research particularly in complex mental health conditions. He leads the Northern Ireland centre for mental health research and policy (NIAMH) and is academic lead for the Ulster hub of the All Ireland Institute for Palliative Care Research.

Membership of our expert steering group

We have convened an expert steering group that has met every six months throughout the programme. The core members of our research team bring expertise in end of life care, care of the elderly, old age psychiatry, health services research, epidemiology, primary care, social science, health economics and statistics. To complement this skill mix we have included a further range of expertise through the external membership of our expert steering group:

Experts in dementia care research- in secondary care - Professor Gill Livingston (UCL), and in primary care-Professor Louise Robinson (Newcastle)

Experts in end of life care: Min Stacpoole (Senior Nurse, St Christopher's Hospice), Claire Henry (Lead NHS National End of Life Programme), Karen Harrison-Dening (Consultant Admiral nurse, Dementia UK and dementia policy adviser to Marie Curie Cancer Care)

Experts in social care: Sharon Blackburn, Chief Executive, English Care Homes association (ECCA), Graham Stokes, BUPA, to represent the private sector

Expert by experience: Mr John Sprange

Patient and Public Involvement

Mr John Sprange will participate in our steering group. His input will be essential and we will encourage and facilitate him in this work through our local Camden Services User Research Forum (SURF).

STUDY OUTPUTS

Dissemination

We shall prepare documents for dissemination by end of life and dementia care organisations such as Marie Curie Cancer Care, BUPA, Dementia UK, The Alzheimer's Society, National End of Life Care programme and the government special advisor for dementia including detailed reports, scientific presentations and papers for peer reviewed journals, and publicise our findings on the Marie Curie website. A summary will be provided to all participants who would like to receive this.

REFERENCES

(1) Knapp M, Privette A. Dementia UK. London: Alzheimer's Society; 2007.

(2) Brayne C, Gao L, Dewey M, Matthews FE. Dementia before death in ageing societies--the promise of prevention and the reality. PLoS Med 2006 October;3(10):e397.

(3) Dewey ME, Saz P. Dementia, cognitive impairment and mortality in persons aged 65 and over living in the community: a systematic review of the literature. Int J Geriatr Psychiatry 2001 August;16(8):751-61.

(4) Sampson EL, Bulpitt CJ, Fletcher AE. Survival of community-dwelling older people: the effect of cognitive impairment and social engagement. J Am Geriatr Soc 2009 June;57(6):985-91.

(5) Reisberg B. Functional assessment staging (FAST). Psychopharmacol Bull 1988;24(4):653-9.

(6) Mitchell SL, Teno JM, Kiely DK, Shaffer ML, Jones RN, Prigerson HG et al. The clinical course of advanced dementia. N Engl J Med 2009 October 15;361(16):1529-38.

(7) McCarthy M, Addington-Hall J, Altmann D. The experience of dying with dementia: a retrospective study. International Journal of Geriatric Psychiatry 1997 March;12(3):404-9.

(8) Sampson EL, Blanchard MR, Jones L, Tookman A, King M. Dementia in the acute hospital: prospective cohort study of prevalence and mortality. Br J Psychiatry 2009 July;195(1):61-6.

(9) Sampson EL, Gould V, Lee D, Blanchard MR. Differences in care received by patients with and without dementia who died during acute hospital admission: a retrospective case note study. Age Ageing 2006 March;35(2):187-9.

(10) Davies, E. and Higginson, I. J. Better palliative care for older people. World Health Organisation Europe; 2004.

(11) Sanders S, Swails P. Caring for individuals with end-stage dementia at the end of life. Dementia 2009;8:117-38.

(12) McCarty CE, Volicer L. Hospice access for individuals with dementia. Am J Alzheimers Dis Other Demen 2009 December 20;24(6):476-85.

(13) Department of Health. End of life care strategy: Promoting high quality care for all adults at the end of life. London: Department of Health; 2008.

(14) Bourne, J. Improving services and support for people with dementia. National Audit Office; 2007.

(15) Sampson EL, Ritchie CW, Lai R, Raven PW, Blanchard MR. A systematic review of the scientific evidence for the efficacy of a palliative care approach in advanced dementia. Int Psychogeriatr 2005 March;17(1):31-40.

(16) Goodman C, Evans C, Wilcock J, Froggatt K, Drennan V, Sampson E et al. End of life care for community dwelling older people with dementia: an integrated review. Int J Geriatr Psychiatry 2009 August 17.

(17) The coalition: our programme for government. London: Cabinet Office; 2010.

(18) Fitch, K., Bernstein, S. J., Aguilar, M., Burnand, B., LaCalle, J., Lazaro, P., van het Loo, M., McDonnell, J., Vader, J. P., and Kahan, J. P. The Rand/UCLA appropriateness method user's manual. Santa Monica: RAND; 2001.

(19) Grol RP, Bosch MC, Hulscher ME, Eccles MP, Wensing M. Planning and studying improvement in patient care: the use of theoretical perspectives. Milbank Q 2007;85(1):93-138.

(20) Ferlie EB, Shortell SM. Improving the quality of health care in the United Kingdom and the United States: a framework for change. Milbank Q 2001;79(2):281-315.

(21) Greenhalgh T, Robert G, Macfarlane F, Bate P, Kyriakidou O. Diffusion of innovations in service organizations: systematic review and recommendations. Milbank Q 2004;82(4):581-629.

(22) American Psychiatric Association. Diagnostic and Statistical Manual of Mental Disorders. 4th Edition ed. Washington DC: American Psychiatric Association; 1994.

(23) Gold Standards Framework. The Gold Standards Framework. NHS End of Life Care Programme 2008;Available from: URL: http://www.goldstandardsframework.nhs.uk/

(24) Thuné-Boyle ICV, Sampson EL, Jones L, King M, Lee D, Blanchard MR. Challenges to improving end of life care of people with advanced dementia in the UK. Dementia 2010;9:285-98.

(25) Sampson EL, Jones L, Thune-Boyle IC, Kukkastenvehmas R, King M, Leurent B et al. Palliative assessment and advance care planning in severe dementia: an exploratory randomized controlled trial of a complex intervention. Palliat Med 2011 April;25(3):197-209.

(26) Walsh K, Jones L, Tookman A, Mason C, McLoughlin J, Blizard R et al. Reducing emotional distress in people caring for patients receiving specialist palliative care. Randomised trial. Br J Psychiatry 2007 February;190:142-7.

(27) Low J, Perry R, Wilkinson S. A qualitative evaluation of the impact of palliative care day services: the experiences of patients, informal carers, day unit managers and volunteer staff. Palliat Med 2005 January;19(1):65-70.

(28) Chambers DA, Glasgow RE, Stange KC. The dynamic sustainability framework: addressing the paradox of sustainment amid ongoing change. Implement Sci 2013;8:117.

(29) Charlson ME. Studies of prognosis: progress and pitfalls. J Gen Intern Med 1987 September;2(5):359-61.

(30) Volicer L, Hurley AC, Lathi DC, Kowall NW. Measurement of severity in advanced Alzheimer's disease. J Gerontol 1994 September;49(5):M223-M226.

(31) Waterlow J. Pressure sores: a risk assessment card. Nursing Times 1985;81(48):49-55.

(32) Reid J, Morison M. Classification of pressure sore severity. Nurs Times 1994 May 18;90(20):46-50.

(33) Zwakhalen SM, Hamers JP, bu-Saad HH, Berger MP. Pain in elderly people with severe dementia: a systematic review of behavioural pain assessment tools. BMC Geriatr 2006;6:3.

(34) Cohen-Mansfield J, Marx MS, Rosenthal AS. A description of agitation in a nursing home. J Gerontol 1989 May;44(3):M77-M84.

(35) Cummings JL, Mega M, Gray K, Rosenberg-Thompson S, Carusi DA, Gornbein J. The Neuropsychiatric Inventory: comprehensive assessment of psychopathology in dementia. Neurology 1994 December;44(12):2308-14.

(36) Kiely DK, Volicer L, Teno J, Jones RN, Prigerson HG, Mitchell SL. The validity and reliability of scales for the evaluation of end-of-life care in advanced dementia. Alzheimer Dis Assoc Disord 2006 July;20(3):176-81.

(37) Weiner MF, Martin-Cook K, Svetlik DA, Saine K, Foster B, Fontaine CS. The quality of life in late-stage dementia (QUALID) scale. J Am Med Dir Assoc 2000 May;1(3):114-6.

(38) Wimo A, Winblad B. Resource utilisation in dementia: RUD Lite. Brain Aging 2003;3:48-59.

(39) Zarit SH, Reever KE, Bach-Peterson J. Relatives of the impaired elderly: correlates of feelings of burden. Gerontologist 1980 December;20(6):649-55.

(40) Zigmond AS, Snaith RP. The hospital anxiety and depression scale. Acta Psychiatr Scand 1983 June;67(6):361-70.

(41) Herdman M, Gudex C, Lloyd A, Janssen M, Kind P, Parkin D et al. Development and preliminary testing of the new five-level version of EQ-5D (EQ-5D-5L). Qual Life Res 2011 December;20(10):1727-36.

(42) Engel SE, Kiely DK, Mitchell SL. Satisfaction with end-of-life care for nursing home residents with advanced dementia. J Am Geriatr Soc 2006 October;54(10):1567-72.

(43) Mitchell SL, Kiely DK, Jones RN, Prigerson H, Volicer L, Teno JM. Advanced dementia research in the nursing home: the CASCADE study. Alzheimer Dis Assoc Disord 2006 July;20(3):166-75.

(44) Sampson EL, Harrison-Dening K, Greenish W, Mandal U, Holman A, Jones L. End of Life Care for People with Dementia. Marie Curie Cancer Care 2009;Available from: URL: http://www.mariecurie.org.uk/forhealthcareprofessionals/end-of-life-dementia.htm

(45) Braun V, Clarke V. Using thematic analysis in psychology . Qual Res Psychol 2006;3(2):77-101.

(46) Spencer, L., Ritchie, J., Lewis, J., and Dillon, L. Quality in qualitative evaluation: A framework for assessing research evidence. London: The Cabinet Office; 2003.

(47) Publications and statistics. Department of Health 2010;Available from: URL: www.dh.gov.uk/en/Publicationsandstatistics/Publications/PublicationsPolicyAndGuidance

(48) Curtis L. PSSRU 2009;Available from: URL: http://www.pssru.ac.uk/uc/uc2009contents.htm

APPENDICES

Appendix 1: Compassion intervention manual

The Final Compassion Intervention Manual will be published with free access on the Marie Curie website (www.mariecurie.org.uk).

Appendix 2. Bedford Alzheimer Nursing Severity (BANS) scale

Please refer to: Volicer L, Hurley AC, Lathi DC, Kowall NW. Measurement of severity in advanced Alzheimer's disease. J Gerontol 1994 September;49(5):M223-M226.

Appendix 3: Waterlow scale

Please refer to: Waterlow J. Pressure sores: a risk assessment card. Nursing Times 1985;81(48):49-55.

Appendix 4: Stirling Wound Assessment Scale

Please refer to Reid J, Morison M. Classification of pressure sore severity. Nurs Times 1994 May 18;90(20):46-50.

Appendix 5: Pain Assessment in Advanced Dementia (PAIND)

Please refer to: Zwakhalen SM, Hamers JP, bu-Saad HH, Berger MP. Pain in elderly people with severe dementia: a systematic review of behavioural pain assessment tools. BMC Geriatr 2006;6:3.

Appendix 6: Cohen Mansfield Agitation Inventory (CMAI)

Please refer to: Cohen-Mansfield J, Marx MS, Rosenthal AS. A description of agitation in a nursing home. J Gerontol 1989 May;44(3):M77-M84.

Appendix 7: Neuropsychiatric Inventory (NPI) questionnaire

Please refer to: Cummings JL, Mega M, Gray K, Rosenberg-Thompson S, Carusi DA, Gornbein J. The Neuropsychiatric Inventory: comprehensive assessment of psychopathology in dementia. Neurology 1994 December;44(12):2308-14.

Appendix 8: Symptom Management at the End Of Life in Dementia (SM-EOLD) scale

Please refer to: Kiely DK, Volicer L, Teno J, Jones RN, Prigerson HG, Mitchell SL. The validity and reliability of scales for the evaluation of end-of-life care in advanced dementia. Alzheimer Dis Assoc Disord 2006 July;20(3):176-81.

Appendix 9: Quality of Life in late-stage Dementia (QUALID)

Please refer to: Weiner MF, Martin-Cook K, Svetlik DA, Saine K, Foster B, Fontaine CS. The quality of life in late-stage dementia (QUALID) scale. J Am Med Dir Assoc 2000 May;1(3):114-6.

Appendix 10: Resource Utilisation in Dementia (RUD) - Lite

Please refer to: Wimo A, Winblad B. Resource utilisation in dementia: RUD Lite. Brain Aging 2003;3:48-59.

Appendix 11: The Comfort Assessment in Dying with Dementia scale (CAD-EOLD)

Please refer to: Kiely DK, Volicer L, Teno J, Jones RN, Prigerson HG, Mitchell SL. The validity and reliability of scales for the evaluation of end-of-life care in advanced dementia. Alzheimer Dis Assoc Disord 2006 July;20(3):176-81.

Appendix 12: The Zarit Burden Interview

Please refer to: Zarit SH, Reever KE, Bach-Peterson J. Relatives of the impaired elderly: correlates of feelings of burden. Gerontologist 1980 December;20(6):649-55.

Appendix 13: Hospital Anxiety and Depression Scale (HADS)

Please refer to: Zigmond AS, Snaith RP. The hospital anxiety and depression scale. Acta Psychiatr Scand 1983 June;67(6):361-70.

Appendix 14: The Satisfaction with Care at the End-of-Life in Dementia Questionnaire (SWC/CAD-EOLD)

Please refer to: Kiely DK, Volicer L, Teno J, Jones RN, Prigerson HG, Mitchell SL. The validity and reliability of scales for the evaluation of end-of-life care in advanced dementia. Alzheimer Dis Assoc Disord 2006 July;20(3):176-81.

Appendix 15: EQ-5D-5L

Please refer to: Herdman M, Gudex C, Lloyd A, Janssen M, Kind P, Parkin D et al. Development and preliminary testing of the new five-level version of EQ-5D (EQ-5D-5L). Qual Life Res 2011 December;20(10):1727-36.

Appendix 16: Health Care Professional Qualitative Interview Schedule

HCP interview schedule Compassion Study (HCP interview schedule for intervention V1 09.01.2014)

Preamble

Thank you for agreeing to this interview. As you know we have introduced an Interdisciplinary Care Leader into the care home in which you work. The reason why we have invited you today for this discussion is to understand what your thoughts are on this service and if there was anything about this service that you think can be improved. Also, please be assured that the topics that we discuss today are strictly confidential and will remain completely anonymous.

Interview

Firstly, just for the purposes of the recording can you:

1. Describe your current role here

2. The type and amount of contact you have on a day to day basis with residents with severe memory problems (how severe these are, their roles and responsibilities)

Now I would like to talk about the role of the ICL and how it may have influenced the way you perform your job:

3. Tell me about how you found working with the ICL

4. Did the ICL influence the way you performed your role? If so, how? Can you provide some examples of how the ICL did this?

5. Do you think the ICL changed the care you provided to residents?

6. Did you find that it influenced any of the following:
    a. Your knowledge of dementia
    b. How you assess patients with severe memory problems
    c. How you recognise symptoms such as pain and how you manage these symptoms?
    d. Were you given any support and guidance on initiating and implementing advance care plans? If so, can you give us an example of when this happened?
    e. The way you communicate/interact with patients who are no longer able to communicate
    f. How comfortable you are about communicating with family members, including discussions about palliative care and death/dying
    g. How you communicate with other HCPs

7. Tell me about your needs. Did the ICL influence the support that you receive in your role?
    a. E.g., such as support following patient death

For care home manager: Did you notice any changes in the way your staff provided care to patients? How do you feel the ICL was received by your staff?

8. Is there anything about this service that can be improved? Is there anything that you would do differently if you were implementing this service?

Appendix 17: Family Carer Qualitative Interview Schedule

Family carer interview schedule (Compassion Study - Carer interview schedule for intervention V1 09.01.2014)

Preamble

Thank you for agreeing to this interview. The reason that we have invited you along for this discussion is to get an idea of the care and support that you and your relative have received over the last few months. If you feel that you need to stop or leave the room at any time please tell me. Whatever you tell me will be made anonymous for the purposes of the study.

Interview

I'd like to begin by asking you a little bit about X memory problems and your understanding of his/her illness

1. Tell me about X's illness and symptoms over the last few months
    a. Both physiological and psychological needs

2. Tell me about the types of support or services has X received over the last few months
    a. Formal or informal (Religion/spirituality)
    b. Satisfaction

Now I'd like to ask you some questions about your needs as a carer:

3. How have you found dealing with X's illness over the last few months? What have you found particularly difficult?
    a) Both physiological and psychological needs
    b) Own mental health

4. Tell me about the support that you needed including emotional, psychological and social needs religious/spiritual needs. Were your needs assessed?

a. If so, tell me about the services that you were offered to meet these needs (If yes, determine who this was offered by and when this took place)

5. Did you have any discussions with HCP's (GP, Consultant, nursing home staff etc) about (if yes, determine when these took place):
    a. Course of illness
    b. Additional information
    c. Treatments – decision making – past and future
    d. Inclusion of other family members

6. Has anyone discussed your thoughts if X's condition were to deteriorate? If so, who discussed these with you and when?

    a) POA

    b) DNAR

    c) Place of death

    d) ACP – Feasibility of carrying out another person's wishes

7. Has anyone discussed what the future holds for X?
    a. i.e., religious beliefs/spirituality – Any recognition in the home?

8. We would also like to find out if the ICL has influenced the care and support that you and your relative have received over the last few months.
    a. Tell me about any changes to the care and support that both you and X have received over the last few months
    b. Tell me if these changes had a positive or a negative impact on you and X
    c. Ways in which we can improve this service? How else can the ICL help you and your relative?

Additional question if patient has passed away: Can you tell me a little about what happened when X passed away?

a) Did it all go smoothly?
b) Were their end of life wishes met? (such as religious/spiritual wishes)
c) Did you receive immediate and ongoing bereavement, emotional and spiritual support?
