# Supplementary material for: Implementing the compassion intervention, a model for integrated care for people with advanced dementia towards the end of life in nursing homes: a naturalistic feasibility study
Source: BMJ Open. 2017 Jul 10;7(6):e015515. doi: 10.1136/bmjopen-2016-015515 (PMC5541605; doi:10.1136/bmjopen-2016-015515)
Supplement: Supplementary material 2 [file bmjopen-2016-015515supp002.pdf]

Evaluation of the implementation of the COMPASSION intervention to improve care towards the end of life for people with advanced dementia residing in two care homes in north London: assessment of long term effects, maintenance and sustainability.

The COMPASSION programme research team:

L Jones, E L Sampson, K Moore, M Elliott , N Kupeli, S Davis, J Harrington, B Candy, V Vickerstaff, A Gola, M King, G Leavey,S Morris, I Nazareth, R Z Omar

Background

The COMPASSION intervention (available from the authors) was developed through a 3 year NIHR portfolio research programme funded by Marie Curie Cancer Care (Jones et al 2012) and it aims to improve end of life care for people with advanced dementia. In the final year of the programme, COMPASSION was implemented, in two care homes in two different clinical commissioning groups, in north London in an exploratory study (ref Elliott 2014).

COMPASSION consists of two key components enabled by an interdisciplinary care leader (ICL) working with the multidisciplinary team within the care home and with associated primary and secondary care providers. These components are: (i) facilitation of integrated care (ii) provision of training and support for care home staff and family carers. We anticipate that there will be ripple and diffusion effects that will influence a third component which is the wider political, economic and commissioning environment within each clinical commissioning group.

The two study sites differed in their level of readiness for receipt of the intervention: service provision for care at the end of life for people with advanced dementia was thought to be more developed at the Camden care home. The exploratory study commenced between May and June 2014 and lasted for 6 months at each site.

An important part of understanding the effects of complex healthcare interventions is collecting evidence on their long term effects, both positive and negative, checking for evidence of potential harms, and what factors are affecting maintenance of any change exerted by the intervention (MRC 2008). Much thought has been given to how maintenance and sustainability might be assessed. In a recent paper, Chambers et al 2013 suggest that when an innovation team leaves a test site, it becomes difficult for the routine service providers to adhere to the new model as closely and ‘programme drift’ and ‘voltage drop’ (reduced adherence to protocols) are natural and inevitable processes. However, they argue that each site may adapt what they have learned from the innovation and continue to behave in newly adapted ways that are sympathetic to their own particular context. Thus those components of an intervention that are effective and workable will vary between sites. It is likely that, given this flexibility, such mechanisms are most likely to lead towards the aims and objectives of the intervention or innovative model of care.

Aims

We aim to assess the longer term effects of implementation of COMPASSION at two care home sites by understanding the impact of the intervention on members of the multidisciplinary team involved in the care of residents with advanced dementia.

Design

We shall collect qualitative data from a purposive sample of health and social care professionals in the care home and in associated primary and secondary care services. We shall seek to understand any alterations in how services are organized and resources allocated (such as changes in staffing levels, engagement of the multi-disciplinary team across primary and secondary care) that have occurred since the COMPASSION exploratory intervention team exited the site. We shall use a realist approach to analyzing the data to enable an understanding of the contexts and mechanisms that are operating that are likely to affect outcomes in the care of people with advanced dementia (Pawson and Tilley 1997). We shall consider the mechanisms at the 4 levels recommended in the study of organizational change: individual, group, organizational, and wider economic and political context (Ferlie and Shortell 2001; Grol 2007)

Study setting

2 care homes in North London, UK. BLINDED TO MEET ETHICAL REQUIREMENTS STATED BELOW.

Sample

A maximum of 10 health and social care providers at each site. We shall attempt to approach professionals who have previously been interviewed as part the piloting of our intervention in an exploratory study (Elliott 2014). Where there has been staff turnover, we shall attempt to interview the newly hired personnel. We expect our sample to include health care assistants, trained nursing staff, allied health professionals, social care professionals, care home managers, general practitioners, and members of specialist services such as community palliative care, geriatricians and mental health providers.

Procedures

Participants will be given an information sheet and at least 48 hours to consider whether they wish to take part. Those who agree will be asked to give informed consent to two in-depth qualitative interviews that will be audio-taped and transcribed verbatim. The first interview will take place 4 months after the COMPASSION exploratory team left the site; the second after a further 4 months. Interviews will last between 15-60 minutes. We shall work to a topic guide and our focus will be on understanding the experience of the intervention, whether and how it has affected practice, whether and how it has affected behaviours of individuals and teams, whether and how it has been thought to influence care. We shall explore with care home managers whether there have been changes in resource allocation, service organization and personnel, and whether there have been any effects on the behaviours of the care home owners. In speaking with any newly hired personnel we shall attempt to understand whether any of the effects of COMPASSION are thought to have diffused into their training and practice. In this way we hope to gain an understanding of whether components of COMPASSION have started to become embedded in the culture of each care home.

Data will be collected by a member of the research team who was not involved in the implementation of the COMPASSION intervention.

Analysis

Transcribed interviews will be read and coded for emergent themes using framework analysis (Ritchie and Spencer 1993). Coding and themes will be checked by a second member of the research team. We shall then hold meetings of the wider research team, including those involved in implementation in the exploratory study, to discuss what themes are emerging and categorise them to understand the contexts, mechanisms and outcomes that are operating. We shall use these data to develop a realist programme theory for sustainability of COMPASSION.

We shall consider data collected at four months to develop a provisional programme sustainability theory, and this will be refined in an iterative process using data collected at eight months. See Figure 1 below.

Figure 1. Realist analysis of data and development of programme theories

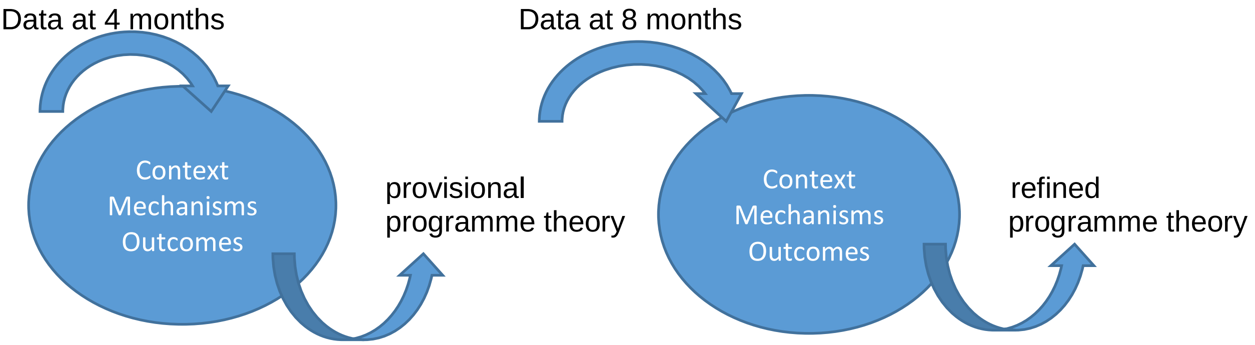

We shall merge these data with qualitative data collected from similar health and social care professionals during the exploratory study to refine an overall programme theory of how COMPASSION has operated throughout its implementation and beyond.

We shall attempt to understand which components of COMPASSION are key to its implementation and which sections of the intervention manual are followed most closely. We shall attempt to describe and understand the reasons for programme drift and voltage drop described by Chambers 2013. We shall consider how our data inform further amendments to the structure and content of COMPASSION and the role of the ICL who was the key implementation person working at each site during the exploratory study. This will allow us to adapt and tailor the intervention manual accordingly.

Economic considerations

We shall not collect any economic data directly. However, we shall use the understanding gained from the qualitative data and work with the health economist within our wider research team to explore how COMPASSION components 1 and 2 have influenced attitudes to commissioning and the wider economic and political context within each participating clinical commissioning group. We shall use refinements we make to the COMPASSION manual to consider the costs of the core components that we retain and whether resource allocation has altered since the intervention ceased. This will inform recommendations for further roll out of the intervention at other sites and for consideration by service planners and providers in the

clinical commissioning groups, the care home provision system and providers of end of life care and care of people with dementia in the NHS and the voluntary sector.

Ethical issues

Data collection in this work will involve health and social care professionals only who will be given information sheets in advance of giving written informed consent for participation in audio-taped interviews. All data will be anonymized and no individual or research site will be identifiable in reports or publications arising from the work.

Data will be kept in locked cabinets using usual procedures within the research department and all procedures will conform to the Data Protection Act.

Plans for dissemination

Findings from this work will be prepared for publication at national and international conferences, in scientific journals and as part of policy documents prepared by organisations involved in dementia and end of life care such as the Alzheimer's Society and Marie Curie.

Findings will be merged with other data arising from the COMPASSION programme. Learning from the programme will be used within the MARQUE programme, funded by ESRC and NIHR in workstreams led by our research team. MARQUE is one of the tranches of work arising from the UK Prime Minister's Dementia Challenge 2013.

References

Chambers DA, Glasgow RE, Stange KC. The dynamic sustainability framework: addressing the paradox of sustainment amid ongoing change. Implement Sci. 2013 Oct 2;8:117. doi: 10.1186/1748-5908-8-117.

Craig P, Dieppe P, Macintyre S, et al. Developing and evaluating complex interventions: the new Medical Research Council guidance. BMJ 2008;337:979–83

Elliot, M., Harrington, J., Moore, K., Davis, S., Kupeli, N., Vickerstaff, V. H., Gola, A., Candy, B., Sampson, E. L., Jones, L. (2014). A protocol for an exploratory phase i mixed-methods study of enhanced integrated care for care home residents with advanced dementia: The compassion intervention. *BMJ Open* doi:10.1136/bmjopen-2014-005661

Grol RPTM, Bosch MC, Hulscher MEJL, Eccles MP, Wensing M. Planning and stuying improvement in patient care: the use of theoretical perspectives. Millbank quarterly Vol 85 No1 2007 (pp93-138)

Jones L, Harrington J, Scott S, et al. CoMPASs: IOn programme (Care of Memory Problems in Advanced Stages of dementia: Improving Our Knowledge): protocol for a mixed methods study. BMJ Open 2012;2:e002265. doi:10.1136/bmjopen-2012-002265

R Pawson and N Tilley (1997) xvii + 235p *REALISTIC EVALUATION*, London: Sage.

Spencer L, Ritchie J, Lewis J, *et al. Quality in qualitative evaluation: A framework for assessing research evidence.* London: The Cabinet Office; 2003. http://www.civilservice.gov.uk/wp-content/uploads/2011/09/a_quality_framework_tcm6-38740.pdf (accessed May 2014)
