# Supplementary material for: Implementing the compassion intervention, a model for integrated care for people with advanced dementia towards the end of life in nursing homes: a naturalistic feasibility study
Source: BMJ Open. 2017 Jul 10;7(6):e015515. doi: 10.1136/bmjopen-2016-015515 (PMC5541605; doi:10.1136/bmjopen-2016-015515)
Supplement: Supplementary material 3 [file bmjopen-2016-015515supp003.pdf]

Supplementary File 3: Context of each NH

**Level**	**Both NHs**	
**Political and economic context (health and social care system in the UK)**	• Both NHs located in north London<br>• Despite policy attempts to integrate services (e.g. Better Care Fund), funding and management of social services are separate from the National Health Service (NHS)<br>• NHs operate within the social service system<br>• The majority of NHs are privately run entities operating for profit<br>• Residents are assessed for eligibility for continuing care funding from their local authority or pay privately for social care<br>• Clinical Commissioning Groups (CCGs) manage priorities for funding of healthcare services and operate locally. The two NHs were located within different CCGs. UK residents are entitled to services through the NHS<br>• Other specialist and allied health services should be available in all NHs, however, access and availability can be uneven[1]<br>• NHs do not require a nurse to be employed, unless beds are allocated as nursing home beds<br>• Some NH beds are also allocated as dementia specific, requiring the NH to have staff with expertise in dementia care	
**Organisational context**	Both NHs privately run by larger companies operating multiple NHs.	
**CCG context**	**Camden CCG**	**Barnet CCG**
Index of Multiple Deprivation#[2]	• 3rd decile of relative deprivation	• 6th decile of relative deprivation
Number of NHs in CCG*	• 13 NHs and care homes (not 24 hour nursing support)	• 91 NHs and care homes (not 24 hour nursing support)
Relevant CCG priorities	• 'Frail and elderly' programme<br>• 'Long term conditions and Cancer' programme[3]	• 'End of life' priority[4]
**Individual NH context**	**NH1**	**NH2**
Beds and levels of care	99 nursing home beds across five units including one dementia specific unit and one younger people with disabilities (not engaged in Intervention)	77 beds with three units: residential care and two nursing care units, one was dementia specific.
Management	Manager and deputy manager. Deputy manager retired half way through implementation.	Manager and Deputy manager. Deputy manager resigned in the weeks prior to implementation.
Nursing and healthcare assistants	Each unit managed by a nurse 24 hours a day with up to five healthcare assistants. Staff involved in direct care work 12hr shifts from 8:00-20:00 or 20:00-8:00	Both nursing units managed by a nurse with up to five healthcare assistants.
Activity co-ordinator	3 part-time staff (approx. 2 full time equivalent)	1 full time
External healthcare professionals		

Level	Both NHs	
GP	All residents registered with one GP clinic. Regular GP visits for 2X3hr sessions per week.	Residents registered with one GP clinic. Regular GP visits for 1X3hr session per week.
Actively involved at NH	Dietetics/nutrition, Geriatrics, Nursing (palliative care; tissue viability; Mental Health), Occupational Therapy, Physiotherapy (although long waiting lists are a deterrent), Podiatry, Social Work, Speech and Language Therapy, Hospital programme facilitating safe discharge from emergency department for complex and frail older patients.	Speech and Language Therapy, Old Age Psychiatry, district nursing (for non-nursing unit)
Available if required	Old Age Psychiatry, psychology	Nursing (palliative care and mental health)
Not available	Care of the Elderly	Geriatrics
Care planning	Care plans are monitored on a monthly basis by the nurse. They are kept as paper based records in the relevant nurse's office. There are templates for different areas of care. Examples of assessments used include: Abbey Pain scale; Doloplus 2, Cornell Depression Scale and Geriatric Depression Scale, Malnutrition Universal Screening Tool, Waterlow Pressure Ulcer Risk, Bradford Dementia Group Wellbeing Profile. Residents typically have 14-20 different care plans. Sentinel events or a significant change in condition will lead to a review and potentially instigating a new care plan as indicated.	Care plans are monitored on a monthly basis by the nurse. They are kept as paper based records in the relevant nurse's office. The template includes 25 different care needs.
Communication processes	• Documentation is manually recorded. Only the manager enters data for generating report back to the NH company.<br>• Verbal handover occurs twice daily during change of shift.<br>• Offer meetings for family members; recent poor attendance was leading the manager to query continued value.<br>• Nurses communicate with other nurses on the same floor working on different shifts using a communication book.<br>• Care plans include communication pages to report when healthcare professionals or family members have had discussions/appointments with NH staff.	• Documentation is manually recorded.<br>• No central place for recording deaths, hospitalisations or other adverse events.<br>• Nurses report in resident care plan on a daily basis and review care plans on a monthly basis. Nurses keep dairies to record resident medical appointments etc.<br>• Handover occurs at staff changeover.<br>• Regular family meetings are held.
Training and professional development	• 40 care staff have National Vocational Qualifications; 20 enrolled in health and social care training.<br>• Electronic matrix shows when each staff member completed compulsory and non-compulsory training flagging those who are due. There are 11 mandated competencies reviewed regularly.<br>• Training sessions rune on a regular basis – staff are informed via flyers in each unit. Sessions are scheduled at a set hour that is the quietest in the afternoon. Expectation that up to half of the staff currently working are given the opportunity to attend.	• No formalised structure for running regular training programmes. Training no longer offered via local palliative care service.<br>• A multi-day dementia training programme was run on an annual for a small number of staff to complete.

Level	Both NHs	
	• Access to training is not available to staff who have been in the country for less than three years. This can be a barrier for upskilling staff.	
Dementia and palliative care	• An advance care plan is developed on admission.<br>• Specialist Palliative Care Nurse from the local hospital's community palliative care nursing service visits the NH regularly and manages complex symptoms at EOL and provides staff training in palliative care. Links commenced 6-7 years earlier when palliative care felt that the NH's referrals were low or inappropriate.<br>• Use local electronic register to inform emergency and out-of-hour services about residents at the EOL and documented care wishes such as 'Do Not Attempt Resuscitation'<br>• 60 nursing and care staff were enrolled (prior to Intervention) in a distant education course about dementia.<br>• The manager attends local dementia strategy meetings.<br>• Manager frustrated by lack of consensus on best care in dementia. Manager felt staff needed more understanding of biological processes in dementia to help understand why a resident is acting the way they are.<br>• Annual memorial function with religious service; family of deceased residents invited.<br>• Two nurses (prior to the Intervention) were attending Gold Standards Accreditation training. Accreditation not achieved during implementation.<br>• During implementation it became evident that there were a range of staff development needs to build skills in dementia and palliative care	• They use the Gold Standards Framework to assess whether residents are nearing EOL. Specialist palliative care specifies that they are only to be called for 'signs and symptoms' (primarily pain management).<br>• Prior to the Intervention they had introduced forms regarding Power of Attorney, the Mental Capacity Act and deprivation of liberty.<br>• Staff have completed syringe driver training; no team available to provide IV antibiotics.<br>• One nurse (prior to the Intervention) attending Gold Standards Accreditation training with the goal of achieving accreditation. Not achieved during implementation.<br>• During implementation it became evident that there were a range of staff development needs to build skills in dementia and palliative care

1st decile = most deprived
* Source: http://www.carehome.co.uk/care_search.cfm (accessed 20th October 2016)

References

1. Seymour JE, Kumar A, Froggatt K. Do nursing homes for older people have the support they need to provide end-of-life care? A mixed methods enquiry in England. *Palliative medicine* 2011;25(2):125-38 doi: doi: 10.1177/0269216310387964published Online First: Epub Date]|.
2. Department for Communities and Local Government. English Indices of Deprivation 2015. Open Government License 2015.
3. NHS Camden Clinical Commissioning Group. 2014/15 Final Annual Report and Outcomes: Working with the people of Camden to achieve the best health for all, 2015.
4. NHS Barnet Clinical Commissioning Group. Annual Report and Accounts 2014/15: Working with local people to develop seamless, accessible care for a healthier Barnet, 2015.
