# Supplementary material for: Implementing the compassion intervention, a model for integrated care for people with advanced dementia towards the end of life in nursing homes: a naturalistic feasibility study
Source: BMJ Open. 2017 Jul 10;7(6):e015515. doi: 10.1136/bmjopen-2016-015515 (PMC5541605; doi:10.1136/bmjopen-2016-015515)
Supplement: Supplementary material 4 [file bmjopen-2016-015515supp004.pdf]

Supplementary File 4: ICL time spent by activity by hours

Activity	NH1 hours (%)	NH2 hours (%)	Hours not attributable to a NH (%)	Total hours (%)	Total Costs*
Assessing needs					
Assessing needs**	122.75 (44)	87.75 (40)	NA	210.5 (32)	£6,241
Meeting family	9.75 (3)	14 (6)	NA	23.75 (4)	£665
Meeting staff	21.75 (8)	16.25 (7)	NA	38 (6)	£1,064
Emails/phone calls^	24 (9)	14.25 (6)	5.75 (4)	44 (7)	£869#
Core meetings	10.25 (4)	5.75 (3)	NA	16 (3)	£448
Wider Meetings	7.5 (3)	NA	NA	7.5 (1)	£210
Staff training					
Preparing training	19 (7)	34.25 (16)	26.75 (17)	80 (12)	£1,753
Providing training	14.25 (5)	19.25 (9)	NA	33.5 (5)	£1,019
Other					
Travel	47.25 (17)	29.75 (13)	30 (19)	107 (16)	£4,053***
ICL professional development	NA	NA	67 (42)	67 (10)	£1,468
ICL clinical supervision	NA	NA	28.75 (18)	28.75 (4)	£463
Total	276.5 (100)	221.25 (100)	158.25 (100)	656 (100)	£18,255

*Source for hourly rate: Department of Health and Health Education England, includes on-costs
**Includes unproductive time in the NH such as waiting to speak to staff, trying to locate staff or records etc.
***Includes cost of train fare
#excludes cost of telephone calls
^includes time speaking with or sending emails to family members
NA = not applicable
