# Supplementary material for: Implementing the compassion intervention, a model for integrated care for people with advanced dementia towards the end of life in nursing homes: a naturalistic feasibility study
Source: BMJ Open. 2017 Jul 10;7(6):e015515. doi: 10.1136/bmjopen-2016-015515 (PMC5541605; doi:10.1136/bmjopen-2016-015515)
Supplement: Author's manuscript [file bmjopen-2016-015515.draft_revisions.pdf]

BMJ Open

Implementing the Compassion Intervention, a model for integrated care for people with advanced dementia towards the end of life in nursing homes: A naturalistic feasibility study

Journal:	*BMJ Open*
Manuscript ID	bmjopen-2016-015515
Article Type:	Research
Date Submitted by the Author:	09-Dec-2016

Complete List of Authors:	Moore, Kirsten; University College London, Marie Curie Palliative Care Research Department<br>Candy, Bridget; University College London, Marie Curie Palliative Care Research Department<br>Davis, Sarah; University College London, Marie Curie Palliative Care Research Department<br>Gola, Anna; Marie Curie Palliative Care Research Unit, UCL Division of Psychiatry<br>Harrington, Jane; University College London, Marie Curie Palliative Care Research Department<br>Kupeli, Nuriye; Marie Curie Palliative Care Research Unit, UCL Division of Psychiatry<br>Vickerstaff, Victoria; University College London, Marie Curie Palliative Care Research Department<br>King, Michael; University College Medical School, Division of Psychiatry<br>Leavey, Gerard; University of Ulster, Psychology<br>Nazareth, Irwin; UCL, Primary Care and Population Health<br>Omar, Rumana; University College London,<br>Jones, Louise; University College London, Marie Curie Palliative Care Research Department<br>Sampson, Elizabeth; University College London, Marie Curie Palliative Care Research Department

**Primary Subject Heading**:	Palliative care
Secondary Subject Heading:	Neurology
Keywords:	Dementia < NEUROLOGY, Adult palliative care < PALLIATIVE CARE, Change management < HEALTH SERVICES ADMINISTRATION & MANAGEMENT, MEDICAL EDUCATION & TRAINING

SCHOLARONE™
Manuscripts

IMPLEMENTING THE COMPASSION INTERVENTION, A MODEL FOR INTEGRATED CARE FOR PEOPLE WITH ADVANCED DEMENTIA TOWARDS THE END OF LIFE IN NURSING HOMES: A NATURALISTIC FEASIBILITY STUDY

Kirsten J Moore[1], Bridget Candy[1], Sarah Davis[1], Anna Gola[1], Jane Harrington[1], Nuriye Kupeli[1], Victoria Vickerstaff[1], Michael King[2], Gerard Leavey[3], Irwin Nazareth[4], Rumana Z Omar[5], Louise Jones[1], Elizabeth L Sampson[1]

[1]Marie Curie Palliative Care Research Department, Division of Psychiatry, University College London, London, United Kingdom;

[2]Division of Psychiatry, University College London, London, United Kingdom;

[3]Bamford Centre for Mental Health & Wellbeing, University of Ulster, Derry Londonderry, United Kingdom;

[4]Department of Primary Care and Population Health, University College London, United Kingdom;

[5]Department of Statistical Science, University College London, London, United Kingdom;

Corresponding author: Kirsten Moore kirsten.moore@ucl.ac.uk

6th Floor, Maple House, 149 Tottenham Court Road, London, United Kingdom, W1T 7NF

P: +44 (0)20 7679 9488

Word count (excluding title page, abstract, references, figures and tables): 5,103

For peer review only

ABSTRACT

Background: Many people with dementia die in nursing homes, but quality of care may be sub-optimal. We developed the theory-driven 'Compassion Intervention' to enhance end-of-life care in advanced dementia.

Objectives: To (i) understand how the Intervention operated in nursing homes in different health economies; (ii) collect preliminary outcome data and costs of an Interdisciplinary Care Leader to facilitate the Intervention; (iii) check the Intervention caused no harm.

Design: A naturalistic feasibility study of Intervention implementation for 6 months

Settings: Two nursing homes in northern London, United Kingdom.

Participants: Thirty residents with advanced dementia were assessed of whom nine were recruited for data collection; four of these residents' family members were interviewed. Twenty-eight nursing home and external healthcare professionals participated in interviews at seven (n=19), 11 (n=19) and 15 months (n=10).

Intervention: An Interdisciplinary Care Leader led two core Intervention components: 1) integrated, interdisciplinary assessment and care; 2) education and support for paid and family carers.

Data collected: Process and outcome data were collected. Symptoms were recorded monthly for recruited residents. Semi-structured interviews were conducted at seven, eleven and 15 months with nursing home staff and external healthcare professionals and at seven months with family carers. Interdisciplinary Care Leader hours were costed using Department of Health and Health Education England tariffs.

Results: Contextual differences were identified between sites: Nursing Home 2 had lower involvement with external healthcare services. Core components were implemented at both sites but multidisciplinary meetings were only established in Nursing Home 1. The Intervention prompted improvements in advance care planning, pain management and person-centred care; we observed no harm. Six-month Interdisciplinary Care Leader costs were £18,255.

For peer review only - http://bmjopen.bmj.com/site/about/guidelines.xhtml

Conclusions: Implementation was feasible to differing degrees across sites, dependent on context. Our data inform future testing to identify the Intervention's effectiveness in improving end-of-life care in advanced dementia.

Trial registration: ClinicalTrials.gov:NCT02840318

STRENGTHS AND LIMITATIONS OF THIS STUDY

- This feasibility study informs future testing of the Compassion Intervention to identify its effectiveness in improving end of life care for residents with advanced dementia and their families.
- We followed principles of dynamic sustainability, recognising that implementing protocols in real-life settings requires adaptations, and that rigid adherence to guidelines tested in controlled settings may not be suitable or effective in broader contexts.
- We structured our approach using the five phases of implementation described in the literature on whole systems change in healthcare including orientation, insight, acceptance, change and maintenance.
- Recognising the importance of context on implementation, we report on four levels of nursing home context: political and economic; organisational; social; and individual professionals
- As an exploratory study the sample size was small and we did not aim to detect differences or calculate a sample size for future studies.

INTRODUCTION

Dementia is the fourth commonest cause of death in high income countries[1] where most people with dementia die in long-term care institutions including nursing homes (NHs)[2-4]. The European Association for Palliative Care defines good care for people with dementia approaching death as person-centred, involving shared decision-making with the person with

For peer review only - http://bmjopen.bmj.com/site/about/guidelines.xhtml

dementia and family members[5]. This may require an integrated approach [6] and a central care coordinator[5]. UK policy states that care is integrated when “people benefit from care that is person-centred and co-ordinated within healthcare settings, across mental and physical health and across health and social care. For care to be integrated, organisations and care professionals need to bring together all of the different elements of care that a person needs.”[7]

Currently, barriers to integrated care remain[8]. Many NH residents experience burdensome interventions and distressing symptoms during the last months of life[9]. Recent data show higher emergency admissions amongst older people residing in NHs[10], indicating persistent gaps in healthcare planning.

Providing good EOL dementia care is complex, prognosis is unpredictable[11] and managing symptoms is difficult when communication is compromised. The need for a complex intervention is reflected in the European Association for Palliative Care’s 57 recommendations for optimal EOL dementia care[5]. However, interventional research on providing EOL care in dementia is scant[12] and lacks a theoretical basis[13].

Establishing a complex intervention begins with development based on the available evidence and theories, testing its acceptability and feasibility in practice, evaluation via larger trials through to wider dissemination into practice[14]. Practice change theories highlight the challenge of incorporating interventions into practice and the need to consider the effect of context at societal, organisational and individual levels[15].

The Compassion Intervention

Within a three-year research programme funded by Marie Curie Care (National Institute for Health Research, Primary Care Research Network Refs. 12621; 12623)[16], we used the

For peer review only - http://bmjopen.bmj.com/site/about/guidelines.xhtml

RAND/UCLA Appropriateness Method[17] to achieve national consensus on the components of Compassion ('the Intervention'), a complex model of EOL care for people with advanced dementia. The development of the Intervention has been reported[6], is based in theories of multi-level and whole systems change[15 18], and is described in detail in a manual (available on the Marie Curie website).

There are two core components: facilitation of an integrated, multi-disciplinary approach to assessment, treatment and care; and education, training and support for formal and informal carers. The Intervention is aimed at people aged 65 years and over who have advanced dementia using criteria based on an existing model of UK best practice[19]:

a) memory problems indicating a diagnosis of dementia according to the fourth Diagnostic and Statistical Manual of Mental Disorders;
b) Functional Assessment Staging scale grade 6a (difficulty putting on clothing) through to 7f (unable to hold head up)[20];
c) comorbidities or unmanaged symptoms such as agitation, recurrent infections, pain and pressure ulcers.

The Intervention is coordinated by an Interdisciplinary Care Leader (ICL) who scopes local practice and identifies key personnel to support EOL care. Scoping ensures the Intervention complements, rather than duplicates, existing local processes. The ICL establishes and co-ordinates key activities to address the two core components of the Intervention (Table 1). Activities to facilitate component 1 include: (i) person-centred assessment of residents, focussing on their physical, psychological, emotional and social needs, (ii) meetings of the core care team and the wider multidisciplinary care teams. Activities to facilitate component 2 include: (iii) staff training sessions, education and support for NH staff and family carers. The ICL role requires clinical experience in care of frail older people and those with dementia, particularly towards EOL. Skills may be drawn from the fields of nursing, social work or a profession allied to medicine.

For peer review only

For peer review only - http://bmjopen.bmj.com/site/about/guidelines.xhtml

Table 1: Key activities of the Compassion Intervention

Component and activity	Purpose	Who is involved	Content
1: facilitation of an integrated, multi-disciplinary approach to assessment, treatment and care:<br>a) Individual holistic resident assessment	To identify symptoms, areas of current unmet need, anticipated future needs and corresponding actions and goals.	The ICL assesses eligible residents in conjunction with NH nurses and healthcare assistants. The process involves liaison with the resident and family about their perceived needs, issues and expectations regarding EOL care.	Assessment template:<br>• Dementia diagnosis and progression (Functional Assessment Staging scale)<br>• Significant other medical conditions<br>• Life history, interests<br>• Important goals for care & wellbeing<br>• Needs or restrictions related to faith and/or culture<br>• EOL wishes<br>• Current medication (and recent changes)<br>• Level of meaningful communication & understanding<br>• Presence of pain or discomfort (Pain Assessment in Advanced Dementia)<br>• Behavioural symptoms and sleep disturbance<br>• Psychological wellbeing, mood, anxiety or depression (Cornell Scale for Depression in Dementia)<br>• Mobility, falls risk, sitting balance and posture, contractures/tone<br>• Skin conditions, pressure sore risk (Waterlow score)<br>• Continence, constipation/bowel problems, UTIs<br>• Eating and swallowing, oral care, weight loss, nutritional status<br>• Other problems – chest infections, breathlessness, fits, blackouts<br>• Recent change in condition<br>• Summary of unmet needs and anticipated/ future needs<br>• Action plan and goals
1: facilitation of an integrated, multi-disciplinary approach to assessment, treatment and care:<br>b) Weekly core meetings	To review, agree on and enact (including referrals), the individual holistic resident assessments.	The core team includes those responsible for medical, nursing and social needs of resident and may include: the clinician responsible for resident's medical needs (GP, geriatrician or Old Age Psychiatrist), NH staff responsible for resident's social care and nursing needs, and the ICL	Review of individual assessments including developing an action plan to address areas of unmet need, discussion of anticipated needs, an escalation plan for the most likely 'what ifs', review of medications and prescribing 'just in case' medications if appropriate and review of EOL wishes and resuscitation status to ensure these are clearly documented. A review date and whether the resident's needs require discussion with the wider team will be decided.
1: facilitation of an integrated, multi-	To discuss (in person or via teleconference), complex cases	The wider team will consist of the core team plus any local health and social care professionals and specialist	The core team will present for discussion residents who have complex needs requiring specialist advice or those where actions agreed by the core

For peer review only

Component and activity	Purpose	Who is involved	Content
disciplinary approach to assessment, treatment and care: c) Monthly wider team meetings	and review care plans, consider significant events, critical incident analysis.	services involved in the care of people with advanced dementia. This is likely to include General Practice, Care of the Elderly, Old Age Psychiatry, Palliative Care, Social Services and Community services such as District Nursing, Speech and Language Therapy, Dietetics, Tissue Viability, Physiotherapy and Occupational Therapy. Composition will depend on local working practices and the availability of key personnel.	team have not been successful at alleviating symptoms. The wider team will also consider learning or training needs that may become evident as a consequence of this shared working. The meetings will include discussion of critical incidents, deaths, hospital admissions, complaints or compliments, and significant events relating to the care of residents so that learning points can be identified.
2: Education, training and support for formal and informal carers	To establish and address the educational needs of staff members so that they can recognise and respond effectively to the needs of people with advanced dementia and to support family carers with increased confidence	ICL will work with the NH and wider team to identify and address education needs and will obtain agreement from NH manager to run formal training sessions. The ICL will be supported by the wider team to undertake training and education. The target of training could include staff and family carers.	EOL care for people with advanced dementia linking to core competencies outlined in[21] including:<br>• Communication skills with residents with advanced dementia and family carers<br>• Assessment and care planning<br>• Symptom management to maintain comfort and wellbeing<br>• Advance care planning<br>• Knowledge and values, to understand advanced dementia and EOL care and when to refer to specialist services. To be sensitive to the needs of family carers and to foster respect, dignity and quality care.

Aim

We aimed to (i) understand how the Intervention operated in two nursing homes (NHs) in different health and social care settings; (ii) collect preliminary outcome data and estimate the cost of employing an ICL to inform further evaluative studies; (iii) check that the Intervention caused no physical or psychological harm to residents or their family carers.

METHOD

A naturalistic feasibility study of the Compassion Intervention. We followed the principles of dynamic sustainability, recognising that implementing protocols in real-life settings requires

For peer review only - http://bmjopen.bmj.com/site/about/guidelines.xhtml

adaptations, and that rigid adherence to guidelines tested in controlled settings may not be suitable or effective in broader contexts[22]. We structured our approach using the five phases of implementation described by Grol[18]:

a) Orientation (awareness of the need for a revised model of care; interest and involvement in the work)
b) Insight (understanding of the revised model of care; insight into existing routines of care)
c) Acceptance (positive attitudes to the possibilities of developing practice; a decision to explore change)
d) Change (actual adoption of a new care model; try-out and confirmation of value)
e) Maintenance (new practice integrated into routines; new practice embedded in the organisation).

Recognising the importance of context on implementation, we report on four levels of NH context: political and economic; organisational; social; and individual professionals[18].

We employed a full-time ICL (KM) with a social care background and experience of working with people with dementia in NHs. The ICL received supervision from clinicians with palliative and dementia expertise. Two NHs were invited to participate; both were involved earlier in our research programme and provided data for a longitudinal (9 months) cohort study to understand the clinical context of people with advanced dementia and their family carers[16]. NH managers identified eligible residents. We aimed to assess two residents in each NH per week.

Implementation occurred over 6 months at each site (see published protocol[23], Supplementary file 1 and Supplementary file 2). In month 1, the ICL met with NH managers and key external healthcare professionals, introduced herself to staff and displayed study posters. The Intervention was launched in Nursing Home 1 (NH1) in May 2014 and Nursing

For peer review only - http://bmjopen.bmj.com/site/about/guidelines.xhtml

Home 2 (NH2) in June 2014. Table 1 shows the activities led by the ICL and after six months the ICL ceased active engagement. To assess maintenance of activities, interviews with relevant stakeholders were conducted after the ICL withdrew at months 7, 11 and 15. Participants were recruited from May 2014 to August 2015. The nature of the intervention prevented masking but independent researchers collected individual level resident data and conducted qualitative interviews.

Data collection

Scoping of existing context

The ICL interviewed each NH manager prior to launching the Intervention. Topics included: resident characteristics, staffing levels, care planning and communication processes, access to external healthcare professionals, training opportunities, dementia and palliative care and expectations about the Intervention. This was supplemented through meetings with deputy managers and other external healthcare professionals.

Qualitative and quantitative process data recorded by ICL

The ICL kept a (i) reflective diary recording observations of practice, liaison with staff, family and residents, examples of improvements in care and personal responses to the role[24]; (ii) a daily log of time spent on tasks related to implementation to enable estimation of costs. We assumed that staff time spent in meetings and training was consistent with usual working practice and so was not considered an additional cost; any opportunity costs incurred would have been offset by the training skills acquired. Over six months at each site, the ICL collected monthly NH-wide data on the number of residents with: documented resuscitation status; a pain management plan; preferred place of death recorded; hospital admissions. Data on emergency phone calls and location of deaths were collected. Resident assessments undertaken by the ICL were part of routine care and were maintained within the NH as clinical information according to their governance polices. Findings from

For peer review only - http://bmjopen.bmj.com/site/about/guidelines.xhtml

assessments could be reflected on in the anonymised ICL diary and used to inform other Intervention activities such as training. Formal training sessions with staff and family were formally evaluated by participants.

NH resident data

Monthly individual outcome data from participant residents who had been assessed by the ICL and their family carers were collected by researchers (NK, SD). Residents were recruited during the first four months of implementation to enable at least three months of outcome data. We used measures from our earlier cohort study for simple comparisons and to check for potential harm[16]. To describe the sample at baseline we used the Functional Assessment Staging scale[20], the Charlson Comorbidity Index[25] and Bedford Alzheimer Nursing Scale[26]. To assess resident outcomes we used the Waterlow Scale (pressure ulcer risk)[27], Neuropsychiatric Inventory[28], Cohen Mansfield Agitation Inventory[29], Pain Assessment in Advanced Dementia[30], Symptom Management at EOL in Dementia[31] and Quality of Life in Late Stage Dementia Scale[32]. For carer outcomes we used the 22-item Zarit Burden Interview [33], the Hospital Anxiety and Depression Scale[34], Satisfaction with Care at EOL in Dementia[31] and the Resource Utilization in Dementia Questionnaire[35].

Qualitative interview data from staff and family carers

We conducted semi-structured interviews with a purposive representative sample of NH staff and attending professionals at three time-points (months 7, 11 and 15) after the ICL left the site. Family carers who had agreed for a resident to have monthly individual data collected were invited for interview at month 7. Interviews were audio-recorded and transcribed verbatim. We aimed to: assess participants' views of the strengths and weaknesses of the Intervention; identify whether any changes in practice were implemented due to the Intervention; and explore whether these changes were maintained after the ICL left.

For peer review only - http://bmjopen.bmj.com/site/about/guidelines.xhtml

Analysis

Qualitative analysis

Transcripts were checked against the audio-recording. One researcher involved in interviewing and transcribing (NK) re-read and coded all transcripts using QSR International Pty Ltd NVivo V10 software (2012). Framework analysis was used[36], based on the five phases of implementation[18]. Small chunks of text were extracted and coded, summarising their content. NK categorised each piece of coded text under each of the five phases. After all coded text was categorised, codes were grouped into a smaller number of themes within each phase of implementation. Additional details about each category reported by Grol et al[18] were also used to inform the categorisation process. The revised structure was reviewed by GL to check for agreement with interpretation. This led to an additional theme being incorporated into the context section of the results. Themes were evident in both NHs, unless identified otherwise.

Quantitative analysis

Process data are reported as total number of activities undertaken (Table 1) and total ICL hours spent on different activities. ICL hours spent on activities associated with the implementation were costed using the Department of Health and Health Education England tariffs to estimate the cost of engaging the ICL. Training evaluations and outcomes (facility wide and individual) are reported using descriptive statistics using statistical package IBM SPSS Version 22 (2013). Outcome data were used for monitoring potential harm and to examine the feasibility of collecting measures in future trials, hence a sample size calculation was not performed. For individual assessments we present outcome measures from the last available assessment using descriptive statistics. We also compare these measures with data from our earlier cohort study but did not make statistical comparisons due to an anticipated small sample size.

For peer review only - http://bmjopen.bmj.com/site/about/guidelines.xhtml

Ethics approval and consent to participate

Ethical approval for roll out of Compassion and data collection was granted by the National Research Ethics Service, London—Camden and Islington Research Ethics Committee (Reference 14/LO/0370) and for assessment of maintenance and sustainability by UCL Research Ethics Committee (ID 3618/001). NH managers gave written consent for their site to participate, and permission for the ICL to carry out clinical assessments of eligible residents and have access to their files. None of the residents had capacity to make an informed decision for research participation so NH managers invited their next of kin/primary contact to give agreement. If next of kin were not available, a professional consultee provided agreement according to the Mental Capacity Act (2005). Staff and family gave written informed consent prior to each interview.

RESULTS

We begin by describing the nursing home (NH) context based on the experiences of the ICL, data collected during set-up and qualitative interviews. We describe how the Intervention operated in practice from experiences of the ICL and qualitative interviews. We report the extent to which the core Intervention activities (Table 1) were possible. We present findings from the qualitative interviews to understand the five phases of implementation: orientation, insight, acceptance, change and maintenance [18]. Finally we present individual and NH wide outcomes and cost data to inform future testing or commissioning of a similar intervention. Figure 1 provides a flowchart of all participants. In total 48 interviews were conducted (NH1=30; NH2=18) with 28 NH and external healthcare professionals at seven (n=19), 11 (n=19) and 15 months (n=10). Four family carers all from NH2 were interviewed at seven months.

Figure 1 here

For peer review only - http://bmjopen.bmj.com/site/about/guidelines.xhtml

Context

Supplementary file 3 describes both NHs according to contextual levels; political and economic, organisational, social, and individual professionals[18]. While both NHs were located within the same broader political and economic contexts, they also operated within different local funding systems for health and social care services (Clinical Commissioning Groups; CCGs). NH1 was located in a more socio-economically deprived area[37]. Both NHs were located in CCGs with priorities around EOL, but only the NH1 CCG also had a priority relating to care for the 'frail and elderly'[38 39]. NH1 was located in a CCG with fewer NHs than NH2. Both NHs were part of larger private companies. Key functional differences between NH1 and NH2 related to access and involvement with external healthcare services, level of detail in care planning processes, and procedures for training for staff, all indicating greater support and development of processes in NH1. While NH1 only contained nursing beds (99 beds with 85 for older people), NH2 had three units with only two of these providing nursing care (52 beds). The third unit (25 beds) was a residential unit with visiting nurses only; residents from here were not assessed during the Intervention.

During implementation and through in-depth qualitative interviews, we found that the context of both NHs was characterised by poor knowledge in dementia and EOL care. Training needs were identified in: pain management, clinical observation and needs assessment, communication with family and residents, advance care planning, person-centred care, psychological aspects of dementia and transition planning. For example, concerns were raised by NH nurses and external healthcare professionals about the confidence of NH nurses having EOL conversations with family:

> *"…often these conversations are quite difficult to conduct and it needs time and it needs some background knowledge and I... No disrespect to the nurses here, I just*

For peer review only - http://bmjopen.bmj.com/site/about/guidelines.xhtml

don't think many of them would have the depth of knowledge and skills to actually do that" (NH1 Geriatrician, Month 11)

Staff worried about the pressures of time and the need to complete tasks which sometimes meant basic care tasks were overlooked, lengthy discussions about EOL care were impossible and social engagement with residents was minimal.

Even the patient care, she [ICL] was able to get in and say this one their nails need to be cut, this one has been refusing to get out of bed but their hair needs to be washed, maybe we have applied some approaches but they did not work… [ICL] had all the time, she was able to … give recommendations so actually GP will do this and us [nurses], we'll do this. (NH1 Deputy Manager, Month 7)

Activities undertaken

Assessments, core meetings and training were undertaken in both NHs (Table 2). Weekly core meetings were scheduled, but many were cancelled due to staff leave or immediate resident care needs. At NH2, the GP experienced significant time constraints and attended only the first two meetings. The group agreed to weekly meetings with the ICL, manager and nurse with specific medical issues referred to the GP. Core meetings provided an opportunity to discuss individual assessments. These involved the ICL reviewing the resident's file, observing and talking to them and their family and seeking clarification from NH staff. NH staff had limited time and may have viewed this as duplicating existing assessments. Discussions with families sought views about current care and concerns about EOL care. The ICL intended to involve NH staff in these discussions but competing staff demands usually prevented this. Common issues identified included swallowing and eating difficulties, pain, pressure area care and lack of social engagement. Advance care plan documentation was more routinely discussed in core meetings at NH1 than NH2.

For peer review only - http://bmjopen.bmj.com/site/about/guidelines.xhtml

Table 2: Process Measures

Over 6 month period	NH1	NH2
ICL visits to NH prior to implementation	8	2
ICL visits to NH during implementation	64	53
ICL visits to external HCPs prior to implementation	2 - palliative care nurse and GP	0
ICL visits to external HCPs during implementation	1 – palliative care nurse	1 - palliative care Lead Clinical Nurse Specialist
Core meetings	10 core meetings with GP, deputy manager and nurse from relevant floor (GP missed one meeting)	8 core meetings with manager and a nurse. GP attended first two meetings.
Comprehensive individualised assessments completed	15	15
Individualised assessments discussed at core meeting	15	13
Individual reviews completed	15	0*
Referrals made to external HCPs	6 (2 X Community Mental Health Team; 2 X Speech and Language Therapist; 2 X Occupational Therapist)	4 (3 X Old Age Psychiatrist; 1 X Manual Handling Trainer)
Wider meetings	6 meetings; usually with Geriatrician, GP, palliative care nurse, Triage and Rapidly Elderly Assessment Team, NH nursing staff and deputy manager (and/or manager)	Wider meetings not established. The ICL was able to arrange one meeting with the palliative care nurse, NH manager and deputy manager.
Number of residents assessed by ICL discussed at wider meeting	11	Not applicable
Number of discussions with family members (not number of family members)	15	24
Number of training sessions (total number of attendees)	9 (84)	5 (21)

*No formal reviews involving reassessment were completed at NH2, although there was subsequent discussion of many of the residents at subsequent meetings.

For peer review only - http://bmjopen.bmj.com/site/about/guidelines.xhtml

For peer review only

During core meetings, staff training needs were discussed and sessions planned, including managing distress during hoist transfers (NH1), and understanding pain and behavioural symptoms (both NHs). At NH1 the manager requested a general information session on dementia and EOL care, while at NH2 the manager requested a half-day session for nurses on pain management and discussing EOL care with family. Fewer training sessions were held at NH2 and staff attendance was sub-optimal. Training was positively evaluated (Table 3).

Table 3: Staff training evaluation

	Reducing distress during personal care	Behaviour and pain management		EOL care in dementia	
NH	NH1 (n= 23)	NH1 (n=36)	NH2 (n=12)	NH1 (n=25)	NH2 (n=9*)
Duration in hours	1	1	1	1	4
Sessions	2Xday; 1Xnight	2Xday; 1Xnight	2Xday; 1X night & day	2Xday; 1Xnight	2 X nursing staff
**Evaluation: Median (IQR)**					
Was this training relevant to your day to day work? #	4 (3-4)	4 (4-4)	4 (3-4)	4 (3-4)	4 (3.25-4)
Did you learn anything new from the training? #	3 (3-4)	4 (3.25-4)	4 (3-4)	3 (3-4)	3.5 (3-4)
Do you think this training will influence your work? #	4 (3-4)	4 (4-4)	4 (3-4)	3 (3-4)	4 (3-4)
Was the training level:~	1 (0-1)	1 (1-1)	1 (1-1)	1 (1-1)	1 (1-1)
Did the training provide a useful refresher? #	3 (3-3)	4 (3-4)	3 (3-3.75)	Not asked	Not asked
Has this training improved your confidence in talking to family about EOL care? ^	Not asked	Not asked	Not asked	4 (4-4)	4 (4-4)

*evaluation sheet missing from one attendee

For peer review only - http://bmjopen.bmj.com/site/about/guidelines.xhtml

measured on a 5 point likert scale from 0=Strongly Disagree – 4=Strongly Agree
~ measured on a 3 point likert scale: 0=too basic; 1=about right; 2= too complex
^ measured on a 5 point likert scale from 0=Not at all – 4=Yes, a lot; higher median better

Both managers requested the ICL to run information sessions for family members on issues regarding dementia, EOL symptoms and advance care planning. Twelve family members attended at NH1 with the NH manager. At NH2 the session (6 families) generated much discussion, overran the allotted time and led to a follow-up session (3 families). Evaluations indicated that the sessions were relevant, helpful, contained new information and that the timing was appropriate.

The lower involvement with external healthcare professionals at NH2 prevented establishing wider meetings. At NH1, six months prior to implementation, wider monthly meetings had been initiated. These meetings were supported by the ICL and involved both review of residents requiring palliative care and reflecting on whether EOL care processes could have been better for deceased residents.

Implementation phases

The staff and family interviews give information on the five implementation phases [18].

Phase 1: Orientation

NH managers highlighted their role in promoting the Intervention; "*Within two or three weeks I had gone in and prepared the staff that she [ICL] was going to be here and that she had full access to the records and the staff*" (NH1 Manager, Month 7). Staff and family engagement was attributed to the importance of the Intervention topic. "*I am happy that something like this is going on, that someone is interested and is trying to help people with dementia and end of life*" (NH1 Nurse, Month 7); and "*I think it was right for the programme to suggest and talk about end of life palliative care*" (NH2 Family Carer, Month 7). Characteristics of the ICL were attributed to engaging staff with the Intervention; *"[ICL] was very helpful… I would say*

For peer review only - http://bmjopen.bmj.com/site/about/guidelines.xhtml

she's a very good listener… she's got plenty of time, which I think is lovely" (NH2 Deputy Manager, Month 7).

Phase 2: Insight

As reported under context, NH staff had only basic knowledge regarding dementia EOL care and it was important that they gained insight into the need for practice improvements. Many staff were receptive to receiving information. Training from the ICL improved knowledge and promoted a person-centred view of dementia care. The Intervention provided insights into existing routines critical for driving practice improvements, often highlighting existing deficits in the care being provided:

> *"… through these 6 months I realised... the paperwork was being reviewed, reviewed, reviewed but actually the patient was not being reviewed it was just being carried forward." (NH1 GP, Month 7)*

Whilst wider meetings at NH1 had started before implementation, the ICL also provided an alternative view during these meetings:

> *"…her [ICL] input was useful… during the MDM [wider multidisciplinary meeting]…her feedback and some of her suggestions actually helped us to see things a little bit differently" (NH1 Geriatrician, Month 7)*

Phase 3: Acceptance

Staff were energised by the Intervention as it provided an opportunity to develop new ideas and skills, and, ultimately, improve dementia care:

For peer review only - http://bmjopen.bmj.com/site/about/guidelines.xhtml

"… anybody new coming [in] will come up with new ideas, new experiences from other places, it's building up. You cannot say I am that clever when I am not. I am open to new ideas all the time." (NH1 Nurse, Month 7)

However, initially, the NH staff were wary of change and the ICL experienced some early difficulties engaging:

"I don't know that the staff really understood for quite a while why she [ICL] was there and what she was doing. I don't think it was her problem; I think it was more what the project was all about." (NH1 Palliative Care Nurse, Month 7).

Phase 4: Change

Participants identified practices that had become part of NH protocols and routines as a result of the Intervention. Participants confirmed the value of the ICL's EOL discussions with family carers. At NH1 a modified template to support advance care planning was introduced to replace three existing care plans relating to EOL wishes, and to provide greater guidance to NH staff about how to manage possible EOL symptoms. At NH2 modifiable wall-mounted care charts (Care Charts UK ©) in residents' rooms were introduced to communicate residents' needs and preferences. Greater focus on pain assessment for residents who were unable to communicate led to introducing the Pain Assessment in Advanced Dementia assessment[30] and pain management plans at NH2.

"[ICL] gave me this wonderful sheet about pain control, really and how to… so we've implemented some of the things that she has given to us." (NH2 Deputy Manager, Month 7)

For peer review only - http://bmjopen.bmj.com/site/about/guidelines.xhtml

However, time demands also prevented NH staff and GPs attending Intervention meetings and training:

> *"It was really good what she was saying but I haven't got the time to do it. So she would sit and discuss them and it would take them half an hour forty minutes to talk about two or three patients and if I've got to see fourteen in the morning - I just can't do it." (NH2 GP, Month 7)*

Phase 5: Maintenance

Staff described the new Advance Care Plan at NH1 and pain management plans and the wall mounted care charts at NH2 as being maintained at Months 11 and 15 and becoming embedded into routine care:

> *"The care [nursing] home are actually using her template, developed a new advanced care plan which has incorporated the points that she [the ICL] raised and so that's what we are using now, for all new patients that come in… existing patients, we are transferring gradually. (NH1 GP, Month 11)*

> *Do you know who loves them [care charts] best? Can I tell you, the relatives… they will tell you the detail about their loved one… So the minute somebody comes in I tell them about the work that the ICL did and then I tell them about the 'this is me' life profile… when we had our Care Quality Commission inspection they really liked the 'this is me' profiles (NH2 Manager, Month 15)*

It was apparent that the need for staff development and a shift from task-driven to compassionate care would require a longer duration and further training and support from

For peer review only - http://bmjopen.bmj.com/site/about/guidelines.xhtml

the ICL. Continuing support and training from the ICL could build on this work, further enhancing staff confidence.

> *"I think that if she'd been there for a whole lot longer then what would have happened is there would be an evolving of her role in a sense that the issues that were raised would have become identified by the nurses as routine" (NH1 GP, Month 7)*

Cost of Implementation

Supplementary file 4 presents the time the ICL spent on various activities and this was used to calculate the costs of Implementation. Of the total 656 hours, 42% were spent on NH1 activities, 34% on NH2 activities and 24% on activities not attributable to one particular NH. Engagement of the ICL to implement the Intervention in two NHs for six months was costed at £18,255 including on-costs and travel fares (and excluding time the ICL spent on non-Intervention activities).

Individual resident outcomes

We recruited 9/28 residents assessed by the ICL for monthly data collection (Figure 1). Recruitment was hampered by difficulties engaging with family members who had limited day-to-day involvement with their relative and did not respond to letters and phone calls. Four residents died or moved NH before agreement was obtained. One daughter declined participation due to her family's request that their relative should not be involved in research.

At NH1 the three residents had a median age of 81 years (Interquartile Range [IQR]: 76-93) and two were female. At NH2 the median age of the six residents was 80 years (IQR: 76-85) and all were female. Data were descriptively compared to those from the larger cohort (Table 4). As none of the nine participants died during the data collection period, we compared their outcomes with the 52 participants involved in the cohort study who survived

For peer review only - http://bmjopen.bmj.com/site/about/guidelines.xhtml

the nine month data collection period. Findings in Table 4 suggest that the Intervention did not cause harm to residents, but the effects on carers at NH2 may need further consideration.

Table 4: Resident evaluation data compared with larger cohort

Baseline Assessment	***Cohort study (n=52)****	***NH1 (n=3)***	***NH2 (n=6)***
**Functional Assessment Staging scale**			
6b-6d (Unable to bathe independently – urinary incontinence)	0	0	1
6e-7b (doubly incontinent- loss of ability to speak > 6 words)	21	1	4
7c-7e (ambulatory ability lost-can't hold up head independently)	31	2	1
**Charlson comorbidity index** median (IQR)	6 (6-7)	6 (4-7)	5 (4-6)
**Bedford Alzheimer Nursing Scale** median (IQR)	22 (18-23)	22 (21-24)	22 (20-23)
Final Visit	***Cohort study (n=52)***	***NH1 (n=3)***	***NH2 (n=6)***
**Waterlow Scale (Pressure ulcer risk)**			
High risk (15-19)	14 (27)	1 (33)	1 (17)
Very high risk (≥20)	36 (69)	2 (67)	4 (67)
**Neuropsychiatric inventory -** Number of symptoms, median (IQR)	4 (1.5-6)	2 (2-5)	4 (2-6)
**Cohen Mansfield Agitation Inventory:** behavioural disturbances (≥39)	29 (56)	1 (33)	3 (50)
**Pain Assessment in Advanced Dementia:** (n, %)			
Rest (≥2)	10 (19)	0 (0)	2 (33)
Movement (≥2)	29 (60)	2 (67)	1 (17)
**Symptom Management at EOL in Dementia Scale** median (IQR)	26 (20-35)	30 (26-32)	33 (31-37)
**Quality of Life in Late Stage Dementia Scale** median (IQR)	24.5 (20-28.5)	23 (23-31)	25 (20-28)
Carer measures:	(n= 23)	(n=0)	(n= 4)
**Zarit Burden Interview** median (IQR)	11 (6-18)		23 (15-28)
**Hospital Anxiety and Depression Scale ≥8** n (%)			

For peer review only - http://bmjopen.bmj.com/site/about/guidelines.xhtml

Anxiety	8 (35)		2 (50)
Depression	5 (21)		2 (50)
**Satisfaction with Care at EOL in Dementia Scale** median (IQR)	30 (29-33)		34 (28-39)
**Resource Utilization in Dementia Questionnaire** median (IQR)			
Visits from doctor, physiotherapist, psychologist, other HCP in previous month	1 (1-3)	0 (0-2)	1 (1-2)
All general hospital admissions in previous month	0.5 (0-1)	0 (0-0)	0 (0-0)

*The cohort study involved 85 residents in total but this table only includes the 52 participants who survived the nine month data collection period.
Charlson Comorbidity Index (19 diseases)[25]
Bedford Alzheimer Nursing Scale: range 7-28, higher scores indicate severity[26]
Waterlow Scale: range 2-46, higher score higher pressure ulcer risk[27]
Neuropsychiatric inventory: total symptoms, maximum 12[28]
Cohen Mansfield Agitation Inventory: range 29-203, scores ≥39 indicates clinically significant agitation[29]
Pain Assessment in Advanced Dementia: range 0-10; scores ≥2 indicates pain[30]
Symptom Management at EOL in Dementia: range 0–45; higher scores indicate better symptom control[31]
Quality of Life in Late Stage Dementia Scale: range 11-55, lower scores indicate better quality of life[32]
Zarit Burden Interview: range 0-88, higher scores indicate greater burden[33]
Hospital Anxiety and Depression Scale: Anxiety and depression subscales range 0-21, scores ≥8 indicates clinically significant depression or anxiety[34]
Satisfaction with Care at EOL in Dementia: range 10–40; higher scores indicate more satisfaction with EOL care[31]
Resource Utilization in Dementia Questionnaire[35]

For peer review only

NH wide outcomes

NH wide outcomes were not easily obtained and therefore we reduced collection frequency to three time points (months 1, 4 and 7). Manual searches of daily logs and individual care plans were required. At NH1 resuscitation status was not documented consistently and at NH2 obtaining these data required reading of individual care plans. What data were collected showed few of out-of-hours GP calls and visits, ambulance calls and unplanned hospitalisations. At NH1 pain management plan frequency increased slightly during implementation from 71% to 85% of residents. Preferred place of death was reported for 30% of residents at month 1 and 85% at month 4 (month 7 data were unavailable). These measures could only be collected at month 1 in NH2 where we found one resident (not

For peer review only - http://bmjopen.bmj.com/site/about/guidelines.xhtml

cognitively impaired) had a pain management plan in place, 21% had their preferred place of death recorded and 30% had a documented 'Do not attempt resuscitation' form.

Over the seven month data collection period, 17 NH1 residents died, ten in their usual NH. For the seven hospital deaths, one was the preferred place of death reported by family and another did not have a documented preference. For two residents with the NH documented as the preferred place, families requested their relative be admitted to hospital. At NH2 for the three months in which resident deaths were reported, twelve residents died and seven who had a documented preference, died in their preferred place.

DISCUSSION

Principal findings

We report on how the Compassion Intervention operated in two UK NHs in different healthcare funding systems and the feasibility of implementation. Our data inform evaluative studies to address gaps in EOL care for residents with advanced dementia. We found that implementation was dependent on several aspects of the local NH context. These included the state of readiness for accepting the intervention, in particular local funding priorities within the healthcare system and relations between multidisciplinary care providers across specialist and generalist services; organisational structures within the nursing home including staffing levels, confidence, knowledge and skills of staff, and existing assessment procedures for residents. The period of implementation was short but there was evidence that the Intervention achieved acceptance within both NHs. We noted changes in care processes such as advance care planning, pain management and the introduction of wall-mounted care charts; these were maintained nine months later. Despite limited NH staff availability, three of the four key activities were implemented in both NHs. No wider meetings and fewer training sessions were implemented at NH2 than NH1. The NH context may explain these differences. We were unable to assess whether changes led to better

For peer review only - http://bmjopen.bmj.com/site/about/guidelines.xhtml

outcomes for residents or family, but there were no indications of harm to residents. Of concern was that the small number of carers recruited appeared to have poorer mental health when compared with the wider cohort, despite reporting benefits of participation and higher satisfaction with end of life care.

Strengths and weaknesses

This was an exploratory study. Whilst the sample size was small, we did not aim to detect differences or calculate a sample size for future studies. Our work is strengthened by the theory and evidence underpinning the Intervention described in earlier publications[6 16 23]. We took note of contextual factors affecting the five phases of implementation described in the literature on whole systems change in healthcare[18]. Our Intervention provides a framework to optimise EOL care in accordance with European Association for Palliative Care recommendations[5].

Recruitment of only four informal carers limits our understanding of the impact of the Intervention on families and this needs exploration in future work. There is evidence from other research[40] that carers do benefit from attempts to improve care for relatives with dementia who are dying. We are aware that involvement of the ICL in both roll-out and monitoring of the Intervention (KM) creates potential for bias. This may be counter-balanced by the depth of understanding achieved which was of importance at this stage of evaluation. We engaged independent researchers in the analysis of interviews (NK, GL) and quantitative data (AG, VV, RO, ES) and all co-authors critically reviewed the findings. We have not incorporated an analysis of the ICL diary here, but auto-ethnographic findings have been published elsewhere [24].

For peer review only - http://bmjopen.bmj.com/site/about/guidelines.xhtml

Implications and future research

Consistent with previous studies[41], collecting NH level data proved challenging and further evaluations should allocate resources for collecting reliable data. The low frequency of deaths, unplanned hospitalisations and out-of-hours calls implies a large number of NHs would be required to give sufficient power to investigate NH wide outcomes. Individual measures show more promise as meaningful outcomes for individual residents; the Symptom Management at EOL in Dementia[31] and the Satisfaction with Care at EOL in Dementia[31] Scales can assess multiple EOL symptoms and family satisfaction with care.

Few other interventions have been specifically developed to improve EOL care in advanced dementia. In the US, an interdisciplinary approach towards individualised care plans for residents with advanced dementia achieved this by creating new hospice units within the long term care setting rather than attempting to change NH practice[42]. In the UK, the Gold Standards Framework for Care Homes and the ABC EOL Education Programme promote a palliative approach within care homes (including NHs), although not specifically for residents with dementia[43 44]. A pilot study found that 42% of external facilitators expressed concerns about lack of time to enable adequate support[45]. The level of facilitation in the Compassion Intervention was higher than the ‘high facilitation’ reported in the Gold Standards programme, and training on its own is unlikely to change resistant norms and practices[46].

Our work did not lead to substantial changes to the Compassion intervention manual. However, we added an alternative checklist to prompt nursing homes to review existing assessment domains rather than implementing a new template. Prior to working with this Intervention, NHs should consider the feasibility of weekly core meetings and how to incorporate assessments into existing processes. The role of the ICL appeared crucial in this

For peer review only - http://bmjopen.bmj.com/site/about/guidelines.xhtml

exploratory study; further research is needed to consider how this might be accommodated in routine practice.

Figure 1: Flowchart of participants

Acknowledgements

We thank other members of the Compassion research team, past and present, for their support in completing this research, particularly Sharon Scott and Steve Morris for their earlier contributions in developing the Compassion programme, and Margaret Elliott for her work on developing the Compassion Intervention manual, providing clinical support to the ICL, and undertaking qualitative interviews. We would also like to thank Ritchard Ledgerd and Dr Karen Harrison-Dening for providing clinical support to the ICL outside the research team. Thank you to Professor Martin Marshall for advice on implementation theory. Thank you to Marie Curie for funding and supporting the programme, the two care homes who completed the Intervention and the residents and carers who participated in this study.

Competing interests

All authors have completed the ICMJE uniform disclosure form at www.icmje.org/coi_disclosure.pdf and declare: authors had financial support from Marie Curie for the submitted work; no financial relationships with any organisations that might have an interest in the submitted work in the previous three years; no other relationships or activities that could appear to have influenced the submitted work.

Funding

This work was supported by Marie Curie Cancer Care (now Marie Curie), [grant number: MCCC-FPR-11-U] through a process administered in partnership with Cancer Research UK.

For peer review only - http://bmjopen.bmj.com/site/about/guidelines.xhtml

Authors' contributions

The Intervention conception and the process used to develop core components of the Compassion manual design was undertaken by LJ, ES, MK, GL, IN, RO, BC and JH. ES and LJ managed the study from conception to completion. VV and AG managed all quantitative data analysis and cleaning with input from ES and RO. SD, JH and NK were involved in undertaking qualitative interviews, transcribing and analysing the qualitative data. KM undertook the role as the ICL working in the two care homes and involved in collecting facility wide data, process data and maintaining a reflective dairy. KM prepared the manuscript which was critically reviewed and the final version approved by all authors.

REFERENCES

1. World Health Organization. The top 10 causes of death. Fact sheet N°310, 2014.

2. Houttekier D, Cohen J, Bilsen J, et al. Place of Death of Older Persons with Dementia. A Study in Five European Countries. *Journal of the American Geriatrics Society* 2010;58(4):751-56 doi: 10.1111/j.1532-5415.2010.02771.x.

3. Reyniers T, Deliens L, Pasman HR, et al. International Variation in Place of Death of Older People Who Died From Dementia in 14 European and non-European Countries. *J Am Med Dir Assoc* 2015;16(2):165-71 doi: 10.1016/j.jamda.2014.11.003.

4. Sleeman KE, Ho YK, Verne J, et al. Reversal of English trend towards hospital death in dementia: a population-based study of place of death and associated individual and regional factors, 2001-2010. *BMC neurology* 2014;14:59 doi: 10.1186/1471-2377-14-59.

5. van der Steen JT, Radbruch L, Hertogh CM, et al. White paper defining optimal palliative care in older people with dementia: a Delphi study and recommendations from the

For peer review only - http://bmjopen.bmj.com/site/about/guidelines.xhtml

European Association for Palliative Care. *Palliative medicine* 2014;28(3):197-209 doi: 10.1177/0269216313493685.

6. Jones L, Candy B, Davis S, et al. Development of a model for integrated care at the end of life in advanced dementia: A whole systems UK-wide approach. *Palliative medicine* 2016;30(3):279-95 doi: 10.1177/0269216315605447.
7. Monitor. Guidance: Delivering better integrated care. Secondary Guidance: Delivering better integrated care 2014 (updated 2015). https://www.gov.uk/guidance/enabling-integrated-care-in-the-nhs.
8. Kupeli N, Leavey G, Harrington J, et al. What are the barriers to care integration for those at the advanced stages of dementia living in care homes in the UK? Health care professional perspective. *Dementia (London, England)* 2016 doi: 10.1177/1471301216636302.
9. Mitchell SL, Teno JM, Kiely DK, et al. The Clinical Course of Advanced Dementia. *New England Journal of Medicine* 2009;361(16):1529-38 doi: 10.1056/NEJMoa0902234.
10. Smith P, Sherlaw-Johnson C, Ariti C, et al. Focus on: Hospital Admissions from Care Homes: Nuffield Trust and Health Foundation, 2015.
11. Mitchell SL, Miller SC, Teno JM, et al. Prediction of 6-month survival of nursing home residents with advanced dementia using ADEPT vs hospice eligibility guidelines. *Jama* 2010;304(17):1929-35 doi: 10.1001/jama.2010.1572.
12. Goodman C, Evans C, Wilcock J, et al. End of life care for community dwelling older people with dementia: an integrated review. *International Journal of Geriatric Psychiatry* 2010;25(4):329-37 doi: 10.1002/gps.2343.
13. van der Steen JT, Goodman C. What research we no longer need in neurodegenerative disease at the end of life: the case of research in dementia. *Palliative medicine* 2015;29(3):189-92 doi: 10.1177/0269216315569998.
14. Craig P, Dieppe P, Macintyre S, et al. Developing and evaluating complex interventions: New guidance. Secondary Developing and evaluating complex interventions: New

For peer review only - http://bmjopen.bmj.com/site/about/guidelines.xhtml

guidance 2008. https://www.mrc.ac.uk/documents/pdf/complex-interventions-guidance/.

15. Ferlie EB, Shortell SM. Improving the quality of health care in the United Kingdom and the United States: a framework for change. *The Milbank quarterly* 2001;79(2):281-315 doi: 10.1111/1468-0009.00206.

16. Jones L, Harrington J, Scott S, et al. CoMPASs: IOn programme (Care Of Memory Problems in Advanced Stages of dementia: Improving Our Knowledge): protocol for a mixed methods study. *BMJ open* 2012;2(6) doi: 0.1136/bmjopen-2012-002265.

17. Fitch K, Bernstein S, Aguilar M, et al. The RAND/UCLA Appropriateness Method User's Manual Santa Monica: RAND, 2001.

18. Grol RP, Bosch MC, Hulscher ME, et al. Planning and studying improvement in patient care: the use of theoretical perspectives. *The Milbank quarterly* 2007;85(1):93-138 doi: 10.1111/j.1468-0009.2007.00478.xpublished Online First: Epub Date]|.

19. Sonola L, Thiel V, Goodwin N, et al. Oxleas advanced dementia service. Supporting carers and building resilience: The Kings Fund, 2013.

20. Reisberg B. Functional assessment staging (FAST). *Psychopharmacology bulletin* 1988;24(4):653-9

21. Skills for Care, Skills for Health, National End of Life Care Programme. Developing end of life care practice: a guide to workforce development to support social care and health workers to apply the common core principles and competencies for end of life care. Secondary Developing end of life care practice: a guide to workforce development to support social care and health workers to apply the common core principles and competencies for end of life care 2012. http://www.skillsforcare.org.uk/Documents/Topics/End-of-life-care/A-guide-to-workforce-development-for-end-of-life-care.pdf.

22. Chambers DA, Glasgow RE, Stange KC. The dynamic sustainability framework: addressing the paradox of sustainment amid ongoing change. *Implementation science : IS* 2013;8:117 doi: 10.1186/1748-5908-8-117.

For peer review only - http://bmjopen.bmj.com/site/about/guidelines.xhtml

23. Elliott M, Harrington J, Moore K, et al. A protocol for an exploratory phase I mixed-methods study of enhanced integrated care for care home residents with advanced dementia: the Compassion Intervention. *BMJ open* 2014;4(6):e005661 doi: 10.1136/bmjopen-2014-005661.

24. Saini G, Sampson EL, Davis S, et al. An ethnographic study of strategies to support discussions with family members on end-of-life care for people with advanced dementia in nursing homes. *BMC palliative care* 2016;15(1):55 doi: 10.1186/s12904-016-0127-2.

25. Charlson ME, Pompei P, Ales KL, et al. A new method of classifying prognostic comorbidity in longitudinal studies: development and validation. *Journal of chronic diseases* 1987;40(5):373-83

26. Volicer L, Hurley AC, Lathi DC, et al. Measurement of severity in advanced Alzheimer's disease. *J Gerontol* 1994;49(5):M223-6 doi: 10.1093/geronj/49.5.M223.

27. Waterlow J. Pressure sores: a risk assessment card. *Nurs Times* 1985;81(48):49-55

28. Cummings JL, Mega M, Gray K, et al. The Neuropsychiatric Inventory - Comprehensive assessment of psychopathology in dementia. *Neurology* 1994;44(12):2308-14

29. Cohen-Mansfield J, Marx MS, Rosenthal AS. A description of agitation in a nursing-home. *Journals of Gerontology* 1989;44(3):M77-M84 doi: 10.1093/geronj/44.3.M77.

30. Warden V, Hurley AC, Volicer L. Development and psychometric evaluation of the Pain Assessment in Advanced Dementia (PAINAD) scale. *J Am Med Dir Assoc* 2003;4(1):9-15 doi: 10.1097/01.jam.0000043422.31640.f7.

31. Kiely DK, Volicer L, Teno J, et al. The validity and reliability of scales for the evaluation of end-of-life care in advanced dementia. *Alzheimer Dis Assoc Disord* 2006;20(3):176-81 doi: 10.1097/00002093-200607000-00009published Online First: Epub Date]|.

32. Weiner MF, Martin-Cook K, Svetlik DA, et al. The quality of life in late-stage dementia (QUALID) scale. *J Am Med Dir Assoc* 2000;1(3):114-6

33. Zarit SH, Reever KE, Bach-Peterson J. Relatives of the impaired elderly: correlates of feelings of burden. *Gerontologist* 1980;20(6):649-55 doi: 10.1093/geront/20.6.649.

For peer review only - http://bmjopen.bmj.com/site/about/guidelines.xhtml

34. Zigmond AS, Snaith RP. The hospital anxiety and depression scale. *Acta Psychiatr Scand* 1983;67(6):361-70 doi: 10.1111/j.1600-0447.1983.tb09716.x.

35. Wimo A, Winblad B. Resource Utilization in Dementia: RUD Lite. *Brain Aging.* 2003;3:48-59

36. Gale NK, Heath G, Cameron E, et al. Using the framework method for the analysis of qualitative data in multi-disciplinary health research. *BMC medical research methodology* 2013;13:117-17 doi: 10.1186/1471-2288-13-117.

37. Department for Communities and Local Government. English Indices of Deprivation 2015. Open Government License 2015.

38. NHS Barnet Clinical Commissioning Group. Annual Report and Accounts 2014/15: Working with local people to develop seamless, accessible care for a healthier Barnet, 2015.

39. NHS Camden Clinical Commissioning Group. 2014/15 Final Annual Report and Outcomes: Working with the people of Camden to achieve the best health for all, 2015.

40. Engel SE, Kiely DK, Mitchell SL. Satisfaction with end-of-life care for nursing home residents with advanced dementia. *J Am Geriatr Soc* 2006;54(10):1567-72 doi: 10.1111/j.1532-5415.2006.00900.x.

41. Schnelle JF, Bates-Jensen BM, Chu L, et al. Accuracy of nursing home medical record information about care-process delivery: implications for staff management and improvement. *J Am Geriatr Soc* 2004;52(8):1378-83 doi: 10.1111/j.1532-5415.2004.52372.x.

42. Kovach C, Kovach SA, Wilson PE. The effects of hospice interventions on behaviors, discomfort, and physical complications of end stage dementia nursing home residents. *American journal of Alzheimer's disease and other dementias* 1996;11(4):7-15 doi: 10.1177/153331759601100402.

43. Kinley J, Stone L, Dewey M, et al. The effect of using high facilitation when implementing the Gold Standards Framework in Care Homes programme: a cluster randomised

For peer review only

For peer review only - http://bmjopen.bmj.com/site/about/guidelines.xhtml

controlled trial. *Palliative medicine* 2014;28(9):1099-109 doi: 10.1177/0269216314539785.

44. Mayrhofer A, Goodman C, Smeeton N, et al. The feasibility of a train-the-trainer approach to end of life care training in care homes: an evaluation. *BMC palliative care* 2016;15(1):11 doi: 10.1186/s12904-016-0081-z.

45. Clifford C, Badger F, Plumridge G, et al. Using the Gold Standards Framework in Care Homes: An evaluation of the Phase 2 programme. Secondary Using the Gold Standards Framework in Care Homes: An evaluation of the Phase 2 programme 2007. http://www.endoflifecare-intelligence.org.uk/view?rid=96.

46. Johnson M, Attree M, Jones I, et al. Diagnosis, prognosis and awareness of dying in nursing homes: towards the Gold Standard? *International journal of older people nursing* 2014;9(2):95-105 doi: 10.1111/opn.12024.

For peer review only

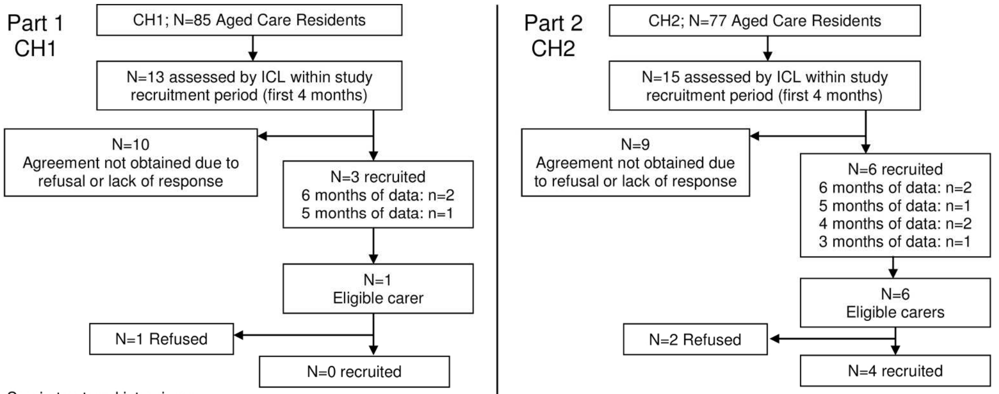

Semi-structured interviews

Role	Month 7	Month 11	Month 15
CH Manager	1	1	1
CH Deputy Manager	2	1	1
CH Nurse	2	3	2
CH Healthcare assistant	4	1	0
CH Activity Co-ordinator	1	1	0
General Practitioner	1	1	1
Geriatrician	1	1	1
Palliative Care Nurse	1	1	0
Mental Health Care Nurse	0	1	0
Family Carers	0		
Total	13	11	6

Role	Month 7	Month 11	Month 15
CH Manager	1	1	1
CH Deputy Manager	1	1	1
CH Nurse	0	2	2
CH Healthcare assistant	2	2	0
CH Activity Co-ordinator	1	1	0
General Practitioner	1	0	0
Geriatrician	NA	NA	NA
Palliative Care Nurse	0	1	0
Mental Health Care Nurse	NA	NA	NA
Family Carers	4		
Total	10	8	4

Figure 1: Flowchart of participants

190x120mm (300 x 300 DPI)

The Compassion Programme

(Care Of Memory Problems in Advanced Stages: Improving Our kNowledge)

Work-stream 3: Pilot study of enhanced integrated care for people with severe memory problems

Principal Investigator: Dr Louise Jones

Study Funders: Marie Curie Cancer Care

For peer review only

The Compassion Programme: WS3- pilot study Version 1, 24th January 2014

SYNOPSIS OF PROGRAMME 8

BACKGROUND 9

Epidemiological background 9

The clinical picture 9

Challenges 9

DEVELOPMENT OF THE COMPASSION MODEL OF ENHANCED CARE 10

Work stream 1 10

Work stream 2 10

Synthesis of findings and development of the enhanced model of care 11

AIMS AND OBJECTIVES OF PILOT STUDY 12

Objectives 12

RECRUITMENT AND CONSENT PROCEDURES 13

Location 13

Recruitment of care homes 13

Consent for implementing the enhanced model of care within the care home 13

Informing participants about the study 14

Recruitment of people with severe memory problems for evaluation of outcomes 14

Consent Procedures 16

Recruitment of family carers to give information for the evaluation 19

Recruitment of enhanced care team and care home staff to participate in qualitative interviews 20

Potential risks/strengths 21

PROJECT INTERVENTION 22

Preparing the Compassion Intervention manual for the enhanced model of care 22

Overview of the intervention 23

DATA COLLECTION 26

For peer review only - http://bmjopen.bmj.com/site/about/guidelines.xhtml

Enhanced care team process data 28

Care home level process data 28

Acceptability of the intervention to care home and enhanced care team staff 29

Outcomes for care home residents receiving the intervention 29

Outcomes for family carers 31

DATA ANALYSIS 34

Quantitative analysis 34

Qualitative analysis 34

Final analyses 35

Sample size 35

Health economics 35

PROJECT MANAGEMENT 35

Project Staffing 36

Core study team 36

Membership of our expert steering group 37

Patient and Public Involvement 38

STUDY OUTPUTS 38

REFERENCES 38

Appendix 1: Compassion intervention manual 42

Appendix 2. Bedford Alzheimer Nursing Severity (BANS) scale 42

Appendix 3: Waterlow scale 42

Appendix 4: Stirling Wound Assessment Scale 42

Appendix 5: Pain Assessment in Advanced Dementia (PAIND) 42

Appendix 6: Cohen Mansfield Agitation Inventory (CMAI) 42

Appendix 7: Neuropsychiatric Inventory (NPI) questionnaire 42

For peer review only - http://bmjopen.bmj.com/site/about/guidelines.xhtml

Appendix 8: Symptom Management at the End Of Life in Dementia (SM-EOLD) scale 42

Appendix 9: Quality of Life in late-stage Dementia (QUALID) 42

Appendix 10: Resource Utilisation in Dementia (RUD) - Lite.. 43

Appendix 11: The Comfort Assessment in Dying with Dementia scale (CAD-EOLD) 43

Appendix 12: The Zarit Burden Interview... 43

Appendix 13: Hospital Anxiety and Depression Scale (HADS) 43

Appendix 14: The Satisfaction with Care at the End-of-Life in Dementia Questionnaire (SWC/CAD-EOLD).. 43

Appendix 15: EQ-5D-5L 43

Appendix 16: Health Care Professional Qualitative Interview Schedule 44

Appendix 17: Family Carer Qualitative Interview Schedule.. 46

For peer review only

The Compassion Programme: WS3- pilot study Version 1, 24th January 2014

Table 1: Abbreviations

BANS	Bedford Alzheimer Nursing Scale
BPSD	Behavioural and Psychological Symptoms of Dementia
CAD-EOLD	The Comfort Assessment in Dying with Dementia Scale
CCG	Clinical Commissioning Group
CCI	Charlson Co-morbidity Index
CMAI	Cohen Mansfield Agitation Inventory
CRF	Case Report Form
CRM	Cluster Representation Mechanism
DSM-IV	Diagnostic and Statistical Manual
FAST	Functional Assessment Staging
GP	General Practitioner
HADS	Hospital Anxiety Scale
HCPs	Health Care Professionals
ICL	Interdisciplinary Care Leader
MRC	Medical Research Council
NPI	The Neuropsychiatric Inventory
PAINAD	Pain Assessment in Advanced Dementia
QALY's	Quality-Adjusted Life Years
QUALID	The Quality of Life in Late Stage Dementia
RAM	Rand Appropriateness Method
RUD-Lite	Resource Utilisation in Dementia
SM-EOLD	Symptom Management at the End of Life in Dementia Scale
SWC/CAD-EOLD	The Satisfaction with Care/Care at dying at the End of Life in Dementia

A note on terminology:

Two groups of carers need to be considered in people with severe memory problems: family (unpaid, informal) carers and paid (formal) carers. Here we use "family carer" as: "someone of any age providing unpaid support to family or friends" (Carers UK). No term is ideal and not all unpaid care is provided by families; "informal carer" is seen to minimise the carer role; and "unpaid carer" suggests a form of voluntary work. Thus "family carer" indicates the family member, friend or other close person acting as the primary unpaid carer for, or key decision maker/supporter of the person with severe memory problems. In addition we refer to "paid carers" in care homes and the community.

Only a third of people with dementia ever receive a formal diagnosis. Therefore in the following protocol, in earlier work streams and information sheets for family and paid carers we have used the term "*severe memory problems*". This allows us to recruit a more representative sample of all those with severe memory problems caused by dementia- many of whom may not have received a previous diagnosis.

For peer review only - http://bmjopen.bmj.com/site/about/guidelines.xhtml

SYNOPSIS OF PROGRAMME

The numbers of people living and dying with severe memory problems are increasing. Currently, people with severe memory problems often receive poor quality end of life care. The aim of our research, funded by Marie Curie Cancer Care as a three year programme grant, is to develop and pilot a complex intervention that aims to improve end of life care for people with severe memory problems. In years 1 and 2 we developed the intervention (Compassion), an enhanced model of existing care. In year 3 we now plan to pilot the Compassion Intervention and assess how it operates in practice.

Our research programme has been divided into three consecutive work streams: In work stream one we defined, in detail, the final disease trajectory of people with severe memory problems. We gained an in-depth understanding of the:

- clinical symptom burden;
- health and social care needs of people with severe memory problems,
- current pathways of care as they reach the end of life;
- needs of their family carers

In work stream two we used mixed methods (focus groups and individual interviews with people with early dementia, family carers and health and social care staff) to develop a complex intervention (Compassion) to improve end of life care. We have defined the core components of the Compassion Intervention which aims to enhance current care, and the circumstances needed to operationalize these. This protocol describes the final work stream in which we shall pilot this enhanced model of care in order to learn and understand how it might operate in practice and to obtain data to inform a future definitive trial.

For peer review only - http://bmjopen.bmj.com/site/about/guidelines.xhtml

BACKGROUND

Epidemiological background

Approximately 600,000 people in the United Kingdom (UK) have dementia (10% of those over 65 years). By 2026 it is estimated that this will approach 840,000 rising to 1.2 million by 2050 (1). One *third of people aged over 65 in the UK will die whilst suffering from dementia* (2). Systematic reviews suggest people with dementia have significantly increased mortality rates (3); even minor cognitive impairment is a strong independent predictor of mortality (4).

The clinical picture

People with severe memory problems can be identified using the Functional Assessment Staging Scale (FAST)(5). At level 6a and above the person will have difficulty putting clothing on properly without assistance, may have difficulty bathing properly, have urinary incontinence, be doubly incontinent or speak only a few words. A retrospective UK study of symptoms experienced in the last year of life by people with severe memory problems compared to cancer patients showed that the symptom burden and health care needs were comparable. In particular, 64% of those with severe memory problems experienced pain (compared to 59% with cancer), 46% breathing difficulties, 39% pressure sores and 86% difficulty with swallowing or loss of appetite (6;7). In people with severe memory problems acute physical illness may be an indicator of imminent death; 24% of those with moderate/severe dementia die after acute unplanned medical admissions compared to 7.5% of those without dementia (8).

Challenges

Essential components of good end of life care are often neglected in people with severe memory problems and referral to palliative care is rare (9) with fewer than 1% of hospice patients in Europe having a neurological diagnosis (10). In people with severe memory problems there are concerns about prognostic uncertainty and whether hospice staff can manage behavioural problems or communication difficulties (11;12);however, most symptoms experienced at the end of life such as pain or difficulties swallowing can be managed with good generalist care (13). Providing care in the usual place of residence is a major aim of the UK Government's End of Life Care Strategy; as well as benefitting patients and family carers this aims to save NHS costs by avoiding acute hospital admissions (13). A recent National Audit Office report indicated that about 50% of care home residents who died in hospital could have died within the care home setting (14). Evidence on how to improve care is limited. Based on available evidence, systematic reviews suggest the need for "care" tends to focus on specific interventions such as pain control, or the withdrawal of aspects of care e.g. *not* prescribing antibiotics (15;16). We suggest that good care requires a broader (but cost effective) palliative approach, tailored to meet the symptoms experienced by those with severe memory problems and also to meet the needs of family carers, particularly in the

For peer review only - http://bmjopen.bmj.com/site/about/guidelines.xhtml

terminal phase and in bereavement. Our work responds to UK government initiatives for care in dementia and at the end of life (13;17).

DEVELOPMENT OF THE COMPASSION MODEL OF ENHANCED CARE

We have used a realistic evaluation framework to develop the intervention, which incorporates information from a wide range of locations and sources. Improving end of life care is a complex undertaking. Our approach acknowledges the importance of context and social processes and allows us to find out about what mechanisms work, in what conditions, why, and how these produce particular outcomes. In brief, our findings so far have informed the enhanced model of care:

Work stream 1

In work stream 1 we conducted detailed research to define the symptom burden and needs of people with severe memory problems at the end of life, and their family carers. We have undertaken a longitudinal cohort study and have recruited 61 people with severe memory problems (FAST stage 7a and above, doubly incontinent and speaks only 5-6 words per day), 57 residing in care homes and four in their own homes. We have also recruited 26 of their family carers. Results from these studies showed how people with severe memory problems have multiple unmet needs, particularly with regards to management of pain and agitation. They are at high risk of pressure sores and have problems with eating and swallowing. There is lack of individual care planning and consideration of end of life care needs.

Work stream 2

Workshops with health and social care professionals

In work stream 2 in a first cycle of workshops we included a wide range of stakeholders and participants at all levels of responsibility. We conducted two workshops in London and one in each of Edinburgh, Solihull and Belfast. We used clinical vignettes describing people with severe memory problems and asked participants to consider how their care could be enhanced to provide solutions to the issues described.

In a second round of workshops we enhanced the content and face validity of our intervention, by using the RAND/UCLA approach (18). A key aspect of this approach is the Rand Appropriateness Method (RAM) which was used as a way to agree the key components of the intervention. To ensure that we took proper account of context further workshops were held across the four countries of the UK (sites in London, Edinburgh, Solihull, Belfast and Penarth). Before each workshop an online process managed by Survey Monkey, asked stakeholders to rank, for appropriateness, statements describing possible intervention components that were derived from the first round of workshops. Results were then analysed before each workshop and any points of disagreement were discussed further in the

For peer review only - http://bmjopen.bmj.com/site/about/guidelines.xhtml

workshop. Following this, participants were asked to rank statements describing components for necessity but independent of economic considerations. Data from all the workshops were pooled and a final bank of statements describing potential components of the enhanced model of care derived.

Interactive qualitative interviews with family carers and healthcare professionals

We conducted individual interactive interviews with 14 family carers and 14 health care professionals from a wide range of stakeholder sources including commissioners and health care assistants. Data analysis is on-going.

Workshops with family carers and people with early dementia

We conducted one workshop with five people with early dementia. We asked them to consider the type of care they would want in the future, especially towards the end of their lives. We also held a workshop with five family carers of people with severe memory problems. They were asked to suggest ways that care could be improved particularly considering end of life care planning and their own experiences of difficulties associated with the transfer of the person with severe memory problems to the acute hospital.

Policy documents

We undertook a detailed review of key documents currently operational in the four countries of the UK. We have focussed on documents that have been published since the National End of Life Care Strategy (2008) and Living Well with Dementia: a national dementia strategy (2009). Using a standardised template, we have summarised key statements arising and looked for similarities and differences in health and social care delivery across the four nations.

Synthesis of findings and development of the enhanced model of care

Findings from the cohort study workshop and interview data suggested a number of issues and ways that care could be improved, for example;

1. Importance of context: considerable regional variation in health and social care organisation and policy within the countries of the UK and Northern Ireland/ detailed repository of policy documents will be used to inform the reporting of our qualitative data and provide context for our recommendations.
2. Training for paid carers at the end of life, learning from hospice model
3. Training for paid carers on difficult conversations and care planning with family carers.
4. Improved staff skills and confidence/more trained nurses in ratio to health care assistants, a medical model like hospice care.
5. Need for enhanced bereavement support for paid and family carers including reflection on the death and care provided
6. Issues in care home culture/ prevent fear of deaths occurring

For peer review only - http://bmjopen.bmj.com/site/about/guidelines.xhtml

7. Lack of engagement of palliative care team/more education on dying with severe memory problems
8. Referrals and multi-disciplinary team working/ single point of contact, continuity of general practitioner (GP) care, rotating staff across environments to bring new learning, out of hours care from GP's who know patients.

The likelihood of successful implementation of our new enhanced care model requires that we understand the sociological theory underlying how our intervention would operate in practice (19). Following the RAM process, we scrutinised retained intervention components and mapped them to the theories described by Grol (19), categorising them according to which of the four operational levels identified by Ferlie and Shortell (20)and others such as Greenhalgh (21). We thought the components might operate on; 1) individual, 2) team, 3) group and 4) system levels. We explored both impact and process theories, operational and utilisation plans at the levels of the individual, social interaction, organisational context and economic/political context.

Details of the enhanced model of care for piloting are presented below (page 16 and Appendix 1).

AIMS AND OBJECTIVES OF PILOT STUDY

Our aim is to conduct a naturalistic pilot study to understand how the Compassion enhanced model of care operates in practice in two care homes in two different health and social care economies; one in the Camden Commissioning Group and one in the Barnet Commissioning Group.

Objectives

In the pilot study we will provide a coordinator with clinical skills- an "Interdisciplinary Care Leader (ICL)" who will coordinate and support the existing team of health and social care professionals working with participating care homes to enhance the management of people with severe memory problems. Our objectives will be met by collecting both quantitative data and qualitative data from the enhanced care team, care home staff and family caregivers

Specific objectives of the pilot study will be to:

1. Understand whether the enhanced model of care is feasible in the setting

2. Determine whether the enhanced model of care is acceptable to staff and family carers of people with severe memory problems in the care home

3. Understand facilitators and barriers to the implementation of the enhanced model of care by collecting qualitative data from paid and family carers on the experience of the intervention

For peer review only - http://bmjopen.bmj.com/site/about/guidelines.xhtml

4. Evaluate whether the enhanced model of care has an impact on a range of national key performance indicators and outcomes including those operating at a number of levels:
    a. Enhanced care team
    b. Care home environment and management
    c. Care home staff
    d. Family carers
    e. Residents with severe memory problems
5. Attempt to describe in detail the costs of delivering the intervention at our pilot sites and the costs of each of its sub-components to inform the commissioning process. These costs can be set against potential benefits and recommendations made

RECRUITMENT AND CONSENT PROCEDURES

Location

Through our previous cohort study we have worked with care homes in the Camden and Barnet Commissioning Group areas. We have chosen these as sites for our pilot intervention because we have previous experience of working with local clinicians including GPs and palliative care teams and they represent different location in terms of the socioeconomic and demographic composition of the area.

Recruitment of care homes

After gaining ethical consent for the study we will approach each care home manager by sending them a letter with brief study details. If the manager is interested, senior study staff will then visit the care home and provide further information regarding the project. We will, at the same time, also approach the proprietor or owner of the care home with similar information and seek their written consent for the home to participate in the enhanced care service and the collection of data from the home for the project outcomes.

Consent for implementing the enhanced model of care within the care home

We will be implementing our intervention of the enhanced care model at the level of the care home; our study can therefore be defined as a cluster pilot evaluation. The model of individual informed consent (or nominee assent) to receive the intervention may not be appropriate for a number of reasons. Firstly we are working with existing clinical services to offer an enhancement of usual care which is in line with the recent English Government Dementia and End of life care strategies. Secondly, we will be training and supporting the existing team to enhance and optimise practice, and thus may influence the care of all residents of the home.

For peer review only - http://bmjopen.bmj.com/site/about/guidelines.xhtml

We have consulted the UK Medical Research Council Guidance Document "Cluster randomised trials: methodological and ethical considerations." Using this framework our intervention is designated as "type A"- interventions that are received (or not) by a whole cluster together so that there is only one decision to be made for the care home. Therefore we use the appropriate Cluster Representation Mechanism (CRM), in our case, the nursing home owners who will give their consent for the intervention to be implemented in their care home. We will also obtain the permission of an ethics committee to implement the enhanced model of care so that the project undergoes appropriate ethical scrutiny. Some evaluation data will be collected at the individual level from the care home and these data will be anonymised, and therefore managers will not be providing any individually identifiable participant data.

Where we will be collecting individual level data, i.e. the qualitative evaluation, resident quality of life and measures from nursing home staff and family carers, we will obtain individual informed consent to participate. We will document how many participants who are approached do consent to us collecting individual level data as this may inform the planning of our future work.

Informing participants about the study

After gaining ethical consent to implement the enhanced care model the research team will meet with care home staff to inform them of the study and to answer or discuss their queries or concerns regarding the study.

Recruitment of people with severe memory problems for evaluation of outcomes

To collect evaluation data we will aim to recruit as many eligible residents as possible from each participating care home. Our criteria have been developed from an existing NHS and Social Care enhanced model of care from South London which has been used by the King's Fund as an example of UK best practice:

Resident Inclusion criteria

1. Aged over 65 years.
2. Severe memory problems indicating a clinical diagnosis of DSM-IV criteria for dementia (22).
3. Moderately severe or severe memory problems as classified on the Functional Assessment Staging Scale (FAST) grade 6a and above (5) see Table 2.

Plus at least one of the following criteria:

- There are recurrent infections, significant weight loss and poor nutrition level, recurrent fevers, pains, falls, severe pressure ulcers that are not easily amenable to treatment, severe physical frailty.

For peer review only - http://bmjopen.bmj.com/site/about/guidelines.xhtml

- OR, the resident has severe, persistent distress (mental or physical) that is not easily amenable to treatment OR another condition (eg. co-morbid cancer) whose co-existence with dementia means that more intrusive treatments would be less appropriate.

Resident Exclusion criteria

- Residents who indicate either verbally or non-verbally that they do not wish to participate.

- Residents who are moribund, in a coma, or those where there are clinical concerns that may preclude them being approached.

Table 2: Functional Assessment Staging Scale (FAST)

STAGE	Description of functions lost
1	No difficulties, either subjectively or objectively
2	Complains of forgetting location of objects. Subjective word finding difficulties.
3	Decreased job functioning evident to co-workers; difficulty in traveling to new locations. Decreased organisational capacity.*
4	Decreased ability to perform complex tasks (e.g. planning dinner for guests, handling personal finances, difficulty marketing etc.)
5	Requires assistance in choosing proper clothing to wear for the day, season or occasion.
6a	Difficulty putting clothing on properly without assistance.
6b	Unable to bathe properly; e.g., difficulty adjusting bath water temperature) occasionally or more frequently over the past weeks.*
6c	Inability to handle mechanics of toileting (e.g., forgets to flush toilet, does not wipe properly or properly dispose of toilet tissue) occasionally or more frequently over the past weeks.*
6d	Urinary incontinence, occasional or more frequent.
6e	Faecal incontinence, (occasional or more frequently over the past week).
7a	Ability to speak limited to approximately a half dozen different words or fewer, in the course of an average day or in the course of an intensive interview.
7b	Speech ability limited to the use of a single intelligible word in an average day or in the course of an interview (the person may repeat the word over and over).
7c	Ambulatory ability lost (cannot walk without personal assistance).
7d	Ability to sit up without assistance lost (e.g., the individual will fall over if there are no lateral rests [arms] on the chair).
7e	Loss of the ability to smile.
7f	Loss of ability to hold up head independently.

*scored primarily on the basis of information obtained from a knowledgeable informant and/or caregiver.

For peer review only - http://bmjopen.bmj.com/site/about/guidelines.xhtml

Consent Procedures

Potential resident participants will have severe memory problems and may be physically frail. It is likely that they may not have the capacity to consent. Therefore our procedure has been developed to comply with capacity legislation governing England and Wales (Mental Capacity Act 2005, Sections 30-34) (see Figure 1).

Residents in care homes with severe memory problems

1. Although it is unlikely that any residents with severe memory problems will have capacity to give consent to participate in the study, The Mental Capacity act requires that we assume a person has this, unless shown otherwise. If the resident has capacity to consent to participate in the data collection, the care home manager will ask the resident if they are willing to see a member of the research team who will then consent them into the study. If capacity is not present the following steps will be taken.

2. On our behalf, the care home manager will attempt to identify their next of kin, family carer or someone close to the person (who does not receive remuneration for this role) who will act as a "personal consultee".

3. If the personal consultee is visiting the care home they will be approached by the care home manager and given verbal information and a written information sheet about the study. They will be encouraged to consider the person's prior wishes or thoughts regarding taking part in research. They will be asked to sign and return a reply slip indicating if they give consent for their contact details to be passed to the research team. If no reply slip is returned to the research team within 14 days, the research team will contact the care home to inform them of this. The care home will then contact the family carer only once and ask if they agree to the home giving the research team their contact details so the research team can contact them regarding the study. If the family carer does not give permission for the care home to give their details to the research team, no further contact will be made. If permission is granted a member of the research team will telephone the family carer. If the personal consultee agrees to the person taking part they will be sent an information sheet and a family carer assent form to sign or be invited to visit the care home and meet with the research team to do this in person. If no assent form is returned within 14 days then the research team will telephone the personal consultee on the maximum of two occasions to see whether they are still interested in participating.

4. If the personal consultee is not available in the care home (i.e. lives a distance from the home or is not able (or wishes) to visit) the care home manager will post the study information sheet to them. They will also be sent a reply slip to sign and return on whether they give permission for the care home to pass their contact details onto the research team. If no reply slip is returned to the research team within 14 days the research team will contact the care home to inform them of this. The care home will contact the family carer only once and ask if they agree to the home giving the research

For peer review only - http://bmjopen.bmj.com/site/about/guidelines.xhtml

team their contact details so the research team can contact them regarding the study. If the family carer does not give permission for the care home to give their details to the research team, no further contact will be made. If permission is granted a member of the research team will telephone the family carer. If the consultee agrees to the person taking part they will be sent a family carer assent form to sign or invited to visit the care home to meet with the research team to do this in person. If no assent form is returned within 14 days then the research team will telephone the personal consultee to see whether they are still interested in participating.

5. If a) no friend or next of kin that can act as a personal consultee is documented in the clinical notes, or, b) after three attempts at telephone contact over one week by the care home manager, they are unable to contact a personal consultee, then the research team will use a professional consultee. This will be defined as a senior experienced health or social care worker who is not directly involved in the research or care of the patient. Through the cohort study we have identified skilled professionals within each CCG who are not involved in the research project or in the patient's direct clinical care and are happy to act in this role. These "consultees" will be given information about the study and training on their responsibilities by the research team. They will follow a structured procedure to give assent for the person's participation in the study and sign their assent for this.

For peer review only - http://bmjopen.bmj.com/site/about/guidelines.xhtml

The Compassion Programme: WS3- pilot study Version 1, 24th January 2014

Figure 1. Summary of consent procedures for the collection of individual level outcome data

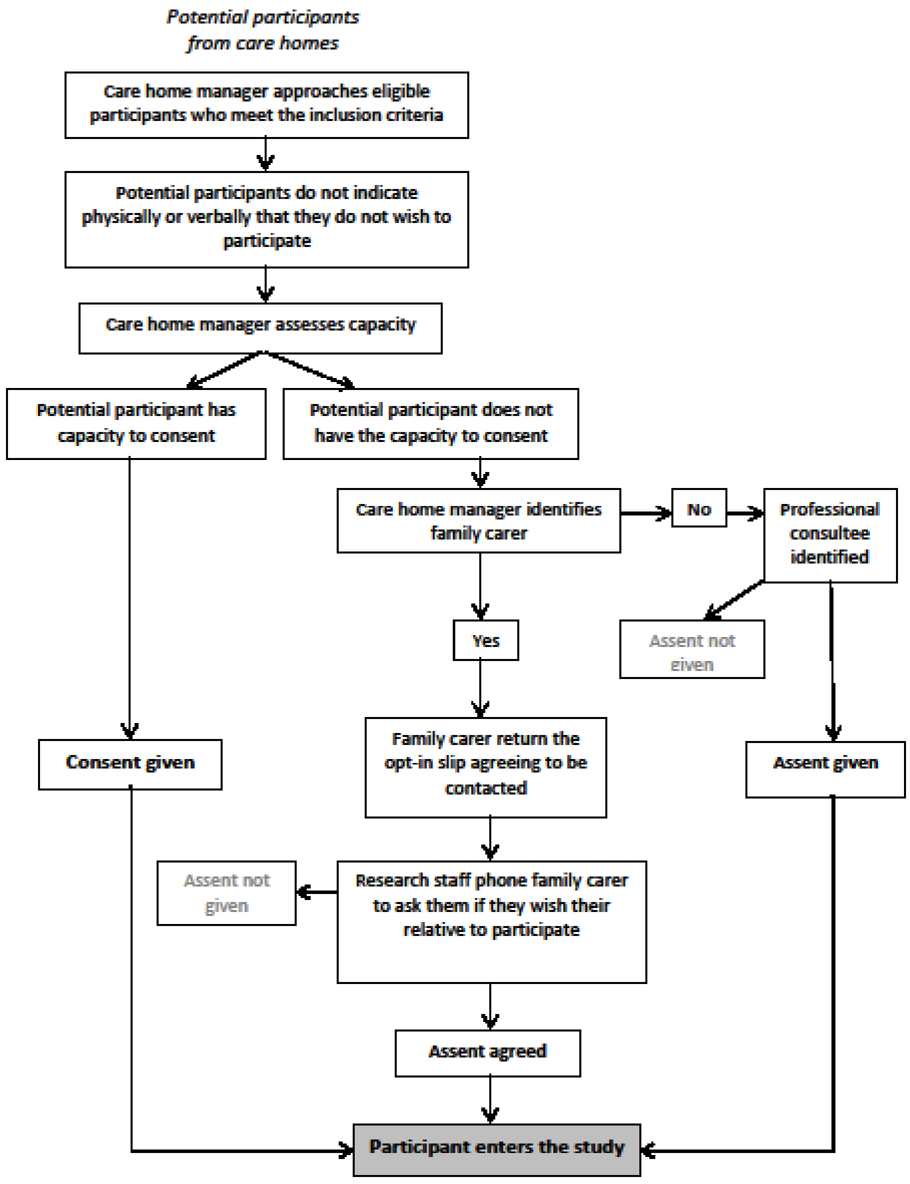

For peer review only - http://bmjopen.bmj.com/site/about/guidelines.xhtml

Recruitment of family carers to give information for the evaluation

We wish to evaluate the opinions of family carers of residents with severe memory problems who have received the enhanced care service. We will only recruit carers of people with severe memory problems who have already entered the study as the recruitment of dyads will enable us to link the experiences of people with severe memory problems and their family carers.

Family carer inclusion criteria

- If the resident with severe memory problems does not have capacity this will be the main family carer (e.g. family member or friend in regular contact and who is the next of kin or a 'key decision maker', identified by the care home manager). If the resident does have capacity we will ask them to nominate who they think is their family carer.
- English language sufficient to complete the study ratings.

Family carer exclusion criteria

- Family carers where there are clinical concerns that may preclude them being approached.
- Family carers aged 16 and under.
- If for any reason during the study the family carer becomes unavailable/unable to give consent we will withdraw the family carer from the study.

Consent procedure

Family carers of residents who do not have capacity to consent will be asked if they wish to participate when we recruit their relative/friend into the study. We will explain that we are interested in exploring their experiences of the enhanced care service now and, should the person die, their experiences of bereavement. They will be informed that they will have two weeks to decide whether they want to participate and can, if they wish, take time to discuss the study further, with other family members/friends, GP and/or research staff. They will be informed that if they decide not to take part that this will not adversely affect the care of their friend/relative or the support they receive as a family carer in any way. If the family carer agrees to participate then a consent form will be sent to them (or given to them when we see them face to face). If the consent form is not returned within 7 days we will contact them again to check whether they still wish to participate. There will be a maximum of two attempts to contact.

Where the resident does have capacity to consent for themselves we will need to recruit family carers independently. The care home manager will approach the family carer and given verbal information and a written information sheet about the study. They will be asked to sign and return a reply slip indicating if they give consent for their contact details to be

For peer review only - http://bmjopen.bmj.com/site/about/guidelines.xhtml

passed to the research team. If no reply slip is returned to the research team within 14 days, the research team will contact the care home to inform them of this. The care home will then contact the family carer only once and ask if they agree to the home giving the research team their contact details so the research team can contact the family carer regarding the study. If the family carer does not give permission for the care home to give their details to the research team, no further contact will be made. If permission is granted, a member of the research team will telephone the family carer. If they agree to participate they will be sent an information sheet and a consent form to sign or be invited to visit the care home and meet with the research team to do this in person. If no consent form is returned within 14 days then the research team will telephone the family carer on the maximum of two occasions to see whether they are still interested in participating.

If the family carer is not available in the care home (i.e. lives a distance from the home or is not able (or wishes) to visit) the care home manager will post the study information sheet to them. They will also be sent a reply slip to sign and return on whether they give permission for the care home to pass their contact details onto the research team. If no reply slip is returned to the research team within 14 days the research team will contact the care home to inform them of this. The care home will contact the family carer only once and ask if they agree to the home giving the research team their contact details so the research team can contact them regarding the study. If the family carer does not give permission for the care home to give their details to the research team, no further contact will be made. If permission is granted a member of the research team will telephone the family carer. If they agree to take part they will be sent a consent form to sign or invited to visit the care home to meet with the research team to do this in person. If no consent form is returned within 14 days then the research team will telephone the family carer to see whether they are still interested in participating.

Recruitment of enhanced care team and care home staff to participate in qualitative interviews

Recruitment and consent of heath care professionals and paid carers

The Interdisciplinary care leader (ICL)/care home manager will identify Healthcare Professionals (HCPs) and paid carers who have been involved in providing care and support to people with severe memory problems in the care home; this will include those from a variety of disciplines and organisations who go into the care home for example, care home staff, general practitioners, speech and language therapists, social workers etc. They will be asked whether they are interested in participating in the research and whether they are happy if the research staff can be given their contact number at work. The researcher will then contact them to discuss the study in detail.

The research team at the research site will ask if they are interested in participating, provide them with an information sheet and ask if they would be happy to participate. They will have

For peer review only - http://bmjopen.bmj.com/site/about/guidelines.xhtml

at least 48 hours to consider whether they wish to participate. They will be informed that their participation is voluntary and individuals or their organisation will not be identifiable in anyway and that all information will be anonymised and kept confidential. If the HCP/paid carer decides they do wish to take part in the study they will be asked to sign a consent form. We intend to conduct a maximum of 10 interviews per care home.

Potential risks/strengths

A strength of our approach is that we have developed our intervention using information gathered from a range of participants. These include health and social care staff, people with early dementia and their family and other unpaid carers. The intervention is also an enhancement of usual care which merely formalises recommendations made in exiting policy documents such as the English National Dementia and End of Life Care Strategies. It is being run in conjunction with established clinical services, adding to their capacity to manage and improve the care of people with severe memory problems who reside in a care home. It will not inhibit the "usual care" that they should receive and **clinical responsibility for the resident's care will, as per usual practice, remain with their GP**. The measures we use to evaluate outcomes are mostly observational with no additional burden or discomfort to the patient and should be part of good routine end of life care (23) therefore the risk of any harm is minimal. If the person with severe memory problems does become upset or uncomfortable in any way with the assessment process, the researcher will stop the assessment immediately and report this to the care home staff and/or the resident's family carer.

We do understand that this research may touch on some sensitive issues for family carers and paid staff, however, the Marie Curie Palliative Care Research Unit has extensive experience of conducting interviews with bereaved relatives of patients with malignant and non-malignant conditions, including end-stage renal disease and advanced dementia (24-27).

In the unlikely event that family carers do become upset in taking part in the study, the researcher will stop the assessment. They will with the family carer's permission ask them if they want to have a break from the assessment, continue or to stop. It is natural that family carers may at times feel emotional when talking about their role or their relative/friend. If the family carer wishes to stop then the assessment will be brought to a close. If they become upset or if their scores on the Hospital Anxiety and Depression Scale (HADS) scale suggest clinical depression or anxiety they will be given information regarding support networks/agencies to contact should they wish, for example, the Admiral Nurse DIRECT or Alzheimer's Society National Help lines, their General Practitioner or other relevant service if there is prior involvement.

The research staff collecting data will be given training and supervision on all of the study assessment tools and family carer interview schedule. The research team will review their recruitment procedures after one month. Any problems will be documented. If substantial

For peer review only - http://bmjopen.bmj.com/site/about/guidelines.xhtml

changes to the protocol are needed we will seek approval of proposed changes from the Research Ethics Committee.

If we discover issues of malpractice, maltreatment or serious neglect, to the degree that the relevant local authority's safeguarding procedures are triggered, we will in this circumstance be required to break patient confidentiality and inform the relevant authorities, following whichever standard local authority safeguarding procedures are in operation.

It is important that issues of sustainability are considered so that we do not leave the care home unsupported after the enhanced care pilot has finished. Evidence suggests that even after the research team have finished the pilot, benefits may persist and that local services maintain and further develop new interventions , so maintaining on going improvements in care; "dynamic sustainability" (28) . One aspect to sustain any benefits is that participating nursing homes will be provided with a structured training programme designed to meet any training/care needs identified during the cohort study.

PROJECT INTERVENTION

Preparing the Compassion Intervention manual for the enhanced model of care

We have produced a written document to describe Compassion in manual form as recommended by the Medical Research Council (MRC) guidance on the development of complex healthcare interventions 2008. This provides a framework by which the intervention can be sustained and becomes replicable at a number of sites. It describes for participating partners the core intervention components and the steps required to implement components. There are two core components:

1. Facilitation of integrated care for people with severe memory problems and their family carers.

2. Education, training and support for health and social care professionals at all levels and for family carers.

The manual in its development was reviewed by key stakeholders during a focus group (care home managers, representatives from palliative care, GPs and care of the elderly physicians). Necessary changes were made, and further amendments were made by the programme grant expert steering group.

The manual describes in detail processes which aim to improve end of life care for people with severe memory problems by:

- Enabling holistic individualised person centred care.

For peer review only - http://bmjopen.bmj.com/site/about/guidelines.xhtml

- Providing an interdisciplinary care leader (ICL) who will act as a central resource for health care professionals, care home staff and family carers involved in the care of people with severe memory problems.
- Developing links and joint working between all those involved in the care and management of people with severe memory problems to establish a model of integrated care.
- Improving the understanding of what is meant by an individualised personal care plan and how such a plan might be worked out and used in practice
- Providing support to front-line staff and managers in care homes to enable them to hold uncertainty and manage risk in people with severe memory problems to avoid unnecessary place of care transfers.
- Identifying, facilitating and supporting the training needs of care home staff in the care of those with severe memory problems.
- Recognising the needs of family carers, including being alert to possible anxiety and depression.
- Supporting the commissioning of effective and sustainable systems to deliver these objectives.

Overview of the intervention

The enhanced model of care delivered by the intervention will run for 6 months. For a detailed description of the intervention see Appendix 1. Facilitating effective clinical change in complex health and social care systems can be challenging. Compassion aims to set out a clear pathway of the actions that need to be taken, and by whom, for its effective implementation. This includes integrating change within existing systems to underpin current expertise and developing an understanding of what is needed for continued best practice. The key people involved in delivering Compassion for the pilot phase are listed below.

Interdisciplinary Care Leader (ICL)

The ICL will be a new post funded through the Compassion research project. The main responsibilities of the ICL will include:

- Developing an understanding of the health and social care professionals, pathways and services relevant to the care home residents with severe memory problems that are currently available.
- Working with the care home staff to identify and assess residents suitable for inclusion in the intervention.
- Establishing who the members of the core team involved in care will be, co-ordinating the weekly meetings and working within the core team to develop and implement personalised care plans for each resident included in the intervention.

For peer review only - http://bmjopen.bmj.com/site/about/guidelines.xhtml

- Establishing the wider clinical team, co-ordinating monthly meetings and maintaining effective communication to facilitate integrated co-ordination of care and the development of good working relationships between all health and social care professionals involved in the care of those with severe memory problems.
- Working with the care home staff to identify and support their educational and training needs, including fostering a culture of respect, dignity and quality of care for all residents and their family carers supporting someone with severe memory problems.
- Meeting with and supporting family carers to ensure their needs and wishes are understood.
- Collecting process data to support evaluation of the intervention.

The ICL will receive training in standard procedures with regard to clinical and information governance, safeguarding of vulnerable adults and the Mental Capacity Act prior to commencing in post. He/she will keep an anonymised reflective diary and will be supported by the research team at the Marie Curie Palliative Care Research Unit.

The Core Team

The core team comprises a range of existing staff who already regularly visit the homes and are responsible for overseeing the medical, nursing and social care needs of residents. During the intervention they will work with the ICL and are the key personnel required to deliver Compassion. The team will meet weekly and includes:

- Clinical Lead Professional (GP supporting the care home, Geriatrician or Old Age Psychiatrist)
- Member of care home staff (care home manager or floor/ unit manager)
- Interdisciplinary Care Leader

The Wider Team

The wider team includes local health and social care professionals and specialist services involved in the care of people with severe memory problems. The team includes staff from General Practice, Care of the Elderly, Old Age Psychiatrist, Palliative Care, Social Services and Community services such as District Nursing, Social Workers, Speech and Language Therapy, Dietetics, Tissue Viability, Physiotherapy and Occupational Therapy. However, the exact composition will depend on local working practices and the availability of key personnel. The wider care team will meet monthly with the core team; meetings may be face to face or via links such as conference calling. The organisation, communication, facilitation and recording of meetings will be the responsibility of the ICL but the team will be required to appoint a lead to chair the meetings.

For peer review only - http://bmjopen.bmj.com/site/about/guidelines.xhtml

The Compassion Programme: WS3- pilot study Version 1, 24th January 2014

The Compassion flow chart, shown below, outlines the steps of the intervention pathway, the roles and the responsibilities of those participating, and the work required within each step of the pathway.

Compassion **Intervention flow chart for pilot study**

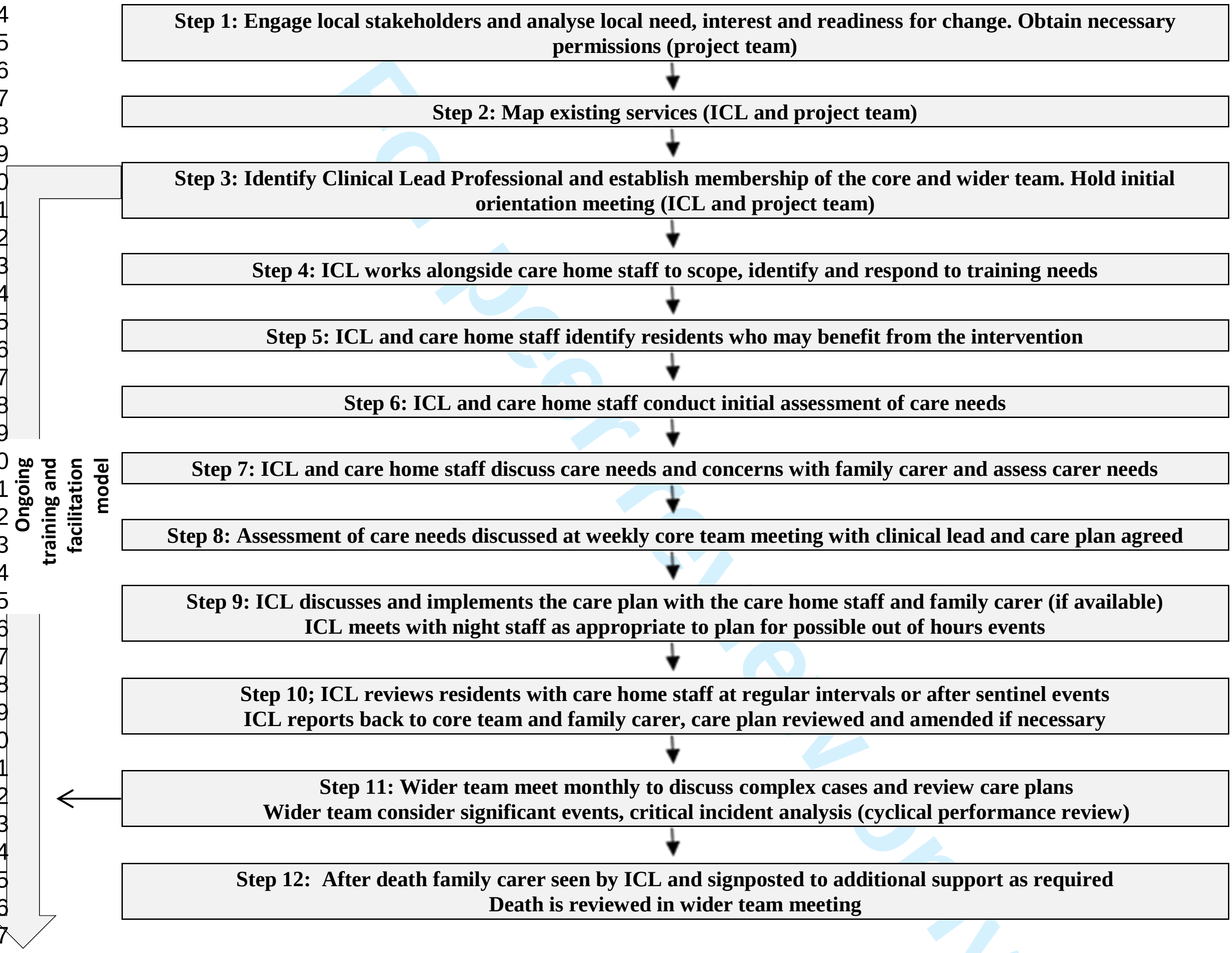

Education and Training considerations

Current education and training provision on end of life care in for people with severe memory problems within the CCG area will be scoped and mapped.

The ICL will work with the care home to help to establish and address the training and educational needs of their staff. This will be primarily by working alongside the staff but may also include one to one reflective discussions with key staff members. Learning and training needs will be addressed in a variety of ways but will include shared working and mentoring, use of online learning resources and formal topic based teaching sessions from local services

For peer review only - http://bmjopen.bmj.com/site/about/guidelines.xhtml

and experts if required. Training will be feasible within timing, staffing and financial constraints and will be agreed with the care home manager.

Education and training provided as part of the intervention will aim to enable care staff to recognise and respond effectively to the needs of people with severe memory problems and to support family carers with increased confidence and competence. Education and training will link to the core competencies outlined in the document "Developing end of life care practice: A guide to workforce development to support social care and health workers to apply the common core principles and competences for end of life care" (Skills for Care, Skills for Health, National End of Life Care Programme. 2012) and will include, communication skills, with residents suffering from severe memory problems and their family carers, assessment and care planning, advance care planning, symptom management to maintain comfort and wellbeing, knowledge and values.

DATA COLLECTION

Our enhanced care model may have an impact at a number of levels, for example on the individual resident and their family carer, on care home staff, at processes which occur at the level of care home management and on the intervention team itself. This is a feasibility study and thus we have to collect data on a range of outcome and process measures, to detect any impacts which the intervention may have on a complex care system and those who reside and work within it. Our measures map onto our key objectives which are to understand the barriers and facilitators to the implementation of the enhanced care model, to assess feasibility and acceptability of the model and to understand the impact of this model on individual residents and their family carers. Data collection is summarised in table 3 (below).

Process data: these will be collected by the ICL and the team delivering the enhanced service. It is evaluation data much of which is already routinely collected within this setting and is required for national NHS and social care end of life care targets and key commissioning performance indicators (marked with * in outcomes table). The data will give us information on the feasibility and acceptability of the intervention and barriers and facilitators to its implementation. This data will be anonymous at source and not collected at an individual level.

Data on individual outcomes: these data will be collected by the research team who will work independently of the enhanced service implementation team. We will collect data from residents with severe memory problems who receive the service, their family carers, individual care home staff and individual members of the intervention team. Thus to collect these data will require individual informed consent (or in the case of care home residents who may lack capacity, assent). For further information in our consent processes please see page 8.

For peer review only - http://bmjopen.bmj.com/site/about/guidelines.xhtml

The Compassion Programme: WS3- pilot study Version 1, 24th January 2014

Table 3: Process and outcomes measures

	Process data	Information on individual outcomes and perspectives
**Enhanced care team**	• Number of residents reviewed<br>• Contacts with family carers<br>• Attendance at team meetings<br>• Number of individual care plans made*<br>• Referral to other specialists	• Experience of participating in the enhanced care intervention<br>• Experience of participating in the enhanced care intervention<br>• ICL reflective practice diary<br>• Barriers and facilitators to the enhanced care intervention
**Care home level data**	• Use of pain tools *<br>• Number of residents with pain management plans*<br>• Recording of surrogate decision makers*<br>• Number of residents with resuscitation status recorded*<br>• Number of deaths within the care home in the last month*<br>• Recording of preferred place of death*<br>• Number of deaths in the usual/preferred place of care*<br>• Numbers of ambulance transfers to acute care*<br>• Visits by out of hours primary care*	
**Care home staff**	• Education and training needs of care home staff and how these were addressed	• Experience of participating in the enhanced care intervention
**Family carer**	• Numbers who have a needs assessment	• Satisfaction with the intervention<br>• Burden<br>• Anxiety and depression<br>• Satisfaction with general care<br>• Quality of life<br><br>If the resident dies:<br>• Satisfaction/quality of end of life care
**Care home resident**	• Number of baseline assessments<br>• Number of review assessments	• Severity of impairment<br>• Pressure sores risk and severity<br>• Pain<br>• Agitation<br>• Behavioural Symptoms<br>• Symptom management at end of

For peer review only - http://bmjopen.bmj.com/site/about/guidelines.xhtml

		life<br>• Quality of life<br>• Resource Utilisation<br>• Number of hospital admissions<br>• Sentinel events<br>• Use of parenteral feeding<br>• Use of personalised care plans<br>• Death in usual /preferred place of care<br><br>If the resident dies:<br>• Use of medication<br>• Burdensome interventions<br>• Adherence to individual care plan

*evaluation data which is already routinely collected within this setting and is required for national NHS and social care end of life care targets and key commissioning performance indicators

Enhanced care team process data

The ICL will record process data on a pro-forma to enable monitoring and evaluation of the enhanced service. These data will be collected on a monthly basis, it will be anonymised and not identifiable at the level of individual residents. These data give us information on the feasibility and acceptability of the intervention and will include:

- Number of residents reviewed by the enhanced care team
- Number of contacts with family carers (by phone and face to face)
- Attendance at team meetings
- Number of individual care plans made by the enhanced care team
- Referral to other specialists outside the care home, for example dietician, speech and language therapists, tissue viability nurses
- Education and training needs of care home staff and how these were addressed i.e., by individual training sessions, referral to online training resources

In addition the ICL will keep a reflective diary (carefully written to ensure anonymisation and confidentiality) recording their experiences of scoping for and implementing the intervention, including notes on care home dynamics, their interactions with the core and wider teams, the care being delivered by staff and any changes being observed that may not be captured by the outcome measures.

Care home level process data

This data will be collected on a monthly basis by the care home manager (to comply with the UK Data Protection Act 1998) in collaboration with the ICL. It will be anonymised and not identifiable at the level of individual residents. Much of this data should already be routinely

For peer review only - http://bmjopen.bmj.com/site/about/guidelines.xhtml

collected and is required by governance organisations and local health and social care commissioners in their assessment of whether services are meeting statutory key performance indicators. The ICL will document:

- Whether pain tools are being routinely used in the care home
- The number of residents with pain management plans
- The recording of surrogate decision makers in the care home records
- Number of residents with resuscitation status recorded
- Number of deaths within the care home in the last month
- Recording of preferred place of death
- Number of deaths in the usual/preferred place of care
- Numbers of ambulance transfers to acute care
- Visits by out of hours primary care

Acceptability of the intervention to care home and enhanced care team staff

We will explore the experience of participating in the intervention with members of the enhanced care team and care home staff. We will conduct qualitative interviews with a purposively sampled selection of staff at the end of the project. These interviews will occur at the end of the intervention period. We will explore the staff experience of the enhanced care team using a structured topic guide which maps onto key areas of current UK end of life and social care policy, for example, how they found working with the ICL, whether the ICL enhanced the way they performed their role, whether the enhanced care model changed how they recognised symptoms such as pain and how these were managed (for interview guide see Appendix 2). Interviews will be audio taped and transcribed verbatim (anonymised). They will last no longer than one hour and participants will be offered the opportunity to review transcripts to ensure accuracy.

Outcomes for care home residents receiving the intervention

These data will be collected independently by the research team only on those residents who have given informed consent to participate or whose relatives have given signed assent for their participation

Demographic information (age, marital status, previous employment) will be collected at the beginning of the evaluation. Severity of dementia will be measured using the FAST scale. At study entry information from GP notes will be obtained by the research team, including: medical co-morbidity (the Charlson Co-morbidity Index (CCI): which includes 19 diseases weighted on the basis of their association with mortality). This allows for the documentation of painful co-morbidities (29).We will document medications from GP prescriptions (e.g. antibiotics, analgesia and antipsychotics). We will document the presence of advance directives, care plans and specific requests regarding hospitalization and resuscitation.

For peer review only - http://bmjopen.bmj.com/site/about/guidelines.xhtml

Clinical assessment

Researchers will assess participants and document their symptom burden with the proforma used in our cohort study (25). It consists of a typical, detailed generalist approach to palliative care.

Functional Assessment Staging Scale (FAST): This observational scale describes a continuum of seven successive stages of functional impairment, from normality to the most severe dementia (5). (See Table 2; ***Recruitment of people with severe memory problems for evaluation of outcomes***)

Bedford Alzheimer Nursing Scale (BANS): This brief 8-item scale is used to stage the level of severe memory impairment in terms of factors such as eye-contact and speech (30). (See Appendix 2).

Pressure sores risk and severity: The Waterlow Scale will be used for the assessment of risk for developing pressure sores (See Appendix 3). It has high inter-rater reliability and sensitivity (31). The Stirling Scale measures the extent of damage from a scale of 1, Non-blanching erythema of intact skin to 4, full-thickness wound, which involving subcutaneous tissue and the deep fascia (32). (See Appendix 4).

Observational scales completed with care home staff or family carers

Pain Assessment in Advanced Dementia (PAINAD): This measures pain during care tasks and at rest. A comprehensive systematic review has identified this tool as having sensitivity and clinical utility (33). (See Appendix 5).

Cohen Mansfield Agitation Inventory (CMAI): This observational scale rates a range of behaviours many of which are relevant and challenging in dementia, for example wandering, grabbing on to people and pushing. It enables measurements over short timescales and is completed with a carer or staff member (34). (See Appendix 6).

The Neuropsychiatric Inventory (NPI): is a brief caregiver questionnaire that is used to assess behavioural and psychological symptoms commonly observed in residents with severe memory problems (BPSD) i.e. psychosis, mood disturbances, agitation, personality changes, pacing, wandering, and appetite disturbances. Its use in primary care is recommended, as it not only assesses the severity of the symptom for the patient but also the distress that the symptom causes the caregiver (35). (See Appendix 7).

Symptom Management at the End of Life in Dementia Scale (SM-EOLD): Is a tool used to assess comfort and pain during the prior 30 days (36). (See Appendix 8).

The Quality of Life in Late Stage Dementia Scale (QUALID): is a validated scale that assesses quality of life over the prior week (37). (See Appendix 9).

For peer review only - http://bmjopen.bmj.com/site/about/guidelines.xhtml

Resource Utilisation in Dementia (RUD)-lite: Resource Utilisation in Dementia (RUD)-lite: Is a short version of the RUD structured interview to assess costs of care including patient accommodation, informal care, community care and hospitalizations (38). (See Appendix 10).

Monthly follow up assessments

Participating residents will be reviewed every four weeks in the care home by the research team, for a maximum of six months, or until death. We will repeat measures: the generalist clinical assessment; Waterlow, Sterling, CMAI, NPI, BANS, PAINAD, SM-EOLD, QUALID, and the RUD-lite. We shall also record prospectively the number of acute hospital admissions, the reasons for these, "burdensome interventions" e.g. enteral feeding tubes (27) and "sentinel events", defined as "new medical conditions that have the potential to lead to a significant change in health status and a shift in the goals of care" e.g. pneumonia, hip fracture (6). Prescription medications and use will also be collected.

Data collection post death

The Comfort Assessment in Dying with Dementia Scale (CAD-EOLD) (36) (See Appendix 11) will be completed with care home staff within 14 days of the resident's death to assess their level of comfort and pain in the seven days prior to their death. Through a review of care home notes we shall record use of medication at the end of life (i.e. "just in case" prescribing, opiates, syringe drivers and artificial hydration or nutrition), sentinel events and burdensome interventions. We will examine adherence to any individual care plans which were made.

Outcomes for family carers

Data will be collected independently by the research team during face to face interviews at study entry within 14 days of the initial resident assessment and then every month, by post or over the telephone (family carers' preference). If family carers are un-contactable for more than 2 months or withdraw from the study we will document the reason and aim to continue to include the person with severe memory problems in the study, unless the carer specifically withdraws their assent.

At project start

We will collect demographic data to include age, sex, ethnicity, education, employment and occupation (present or previous), marital status, relationship to the care home resident, the number of years spent caring and any other caring responsibilities e.g. children under 18 years of age.

For peer review only - http://bmjopen.bmj.com/site/about/guidelines.xhtml

At project start and each monthly follow-up

As with the participating residents, measures (listed below) will be repeated at monthly intervals. We shall inquire about contact with the ICL and whether end of life issues have been mentioned.

Zarit Burden Interview: a 22-item self-report questionnaire, the most consistently used measure of carer burden in dementia. The questionnaire asks the carer to reflect on how they feel when they are caring for the person (39). (See Appendix 12).

Hospital Anxiety and Depression Scale (HADS): a self-report instrument for clinically significant anxiety and depression (40). (See Appendix 13).

The Satisfaction with Care at the End of Life Scale in Dementia Scale **(SWC/CAD-EOLD):** a validated tool that quantifies overall satisfaction with care in advanced dementia. This brief 10-item self-administered questionnaire assesses the caregiver's level of satisfaction with decision-making, medical and nursing care, and their understanding of the condition of the person with dementia (See Appendix 14). The CAD version is used to assess care received around the time of death (36) (see data collection in bereavement - below). (See Appendix 11).

EQ-5D-5L: this instrument is an index-based utility set for the calculation of quality-adjusted life years (QALYs) used to inform health economic evaluations of healthcare interventions (41). (See Appendix 15).

Qualitative interviews

To gain a deeper understanding of how they experience the enhanced care model and working with the ICL we shall offer qualitative interviews with the research team and to all participating family carers in a place of their choice. These will occur at the end of the feasibility study for the enhanced model of care or in bereavement if the resident dies (for interview schedule see Appendices 16 and 17).

Data collection in bereavement

To gain a deeper understanding of the circumstances surrounding the death and the views of the carer on which aspects of care were or were not satisfactory, where possible we shall ask additional questions all bereaved family carers. In this case we will ensure these interviews take place two months after bereavement, this has been found to be the optimal time for such work whereby the carer feels ready to think about their loss but still has sufficient recall of events (42;43). We found in our cohort study that these interviews are acceptable (we have completed ten so far) and family carers are keen to reflect on their experiences (25). The SWC-EOLD scale will be completed to assess family carer's level of satisfaction with care

For peer review only - http://bmjopen.bmj.com/site/about/guidelines.xhtml

and the CAD-EOLD to assess the resident's level of comfort and pain in the 7 days prior to their death from the carer's perspective.

We will items from a topic guide similar to that used successfully in our cohort study which was acceptable to family carers (44). Interviews will be audio taped and transcribed verbatim (anonymised). They will last no longer than one hour and carers will be offered the opportunity to review transcripts to ensure accuracy.

Table 4: Summary of data collection

	Project start	During project (monthly for 6 months)	After death/ in bereavement	After project ends
Enhanced care team process data	x	x		
Care home level data	x	x		
Paid carers/ enhanced care team staff qualitative interviews				x
**Residents**				
Demographic information	x			
FAST scale	x			
Charlson Co-morbidity Index	x			
Medications	x	x		
Prior advance care plans and wishes documented	x			
Symptom burden/generalist clinical assessment	x	x		
Bedford Alzheimer Nursing Scale	x	x		
Pressure sore risk and severity	x	x		
Pain Assessment in Advanced Dementia	x	x		
Cohen Mansfield Agitation Inventory	x	x		
Neuropsychiatric inventory	x	x		
Symptom Management at the End of Life in Dementia Scale	x	x		
Quality of Life in Late dementia Scale	x	x		
Resource Utilization in Dementia Scale	x	x		
Burdensome interventions		x		
Sentinel events		x		
Comfort Assessment in Dying Scale			x	
**Family carers**				
Demographic data	x			
Zarit Burden Interview	x	x		

For peer review only - http://bmjopen.bmj.com/site/about/guidelines.xhtml

Hospital Anxiety and Depression Scale	x	x	x	
Satisfaction with Care at the End of Life in Dementia Scale	x	x	x	
Comfort Assessment in Dying Scale			x	
EQ-5D-5L	x	x	x	
Qualitative interviews			x	x

DATA ANALYSIS

Data will be collected at the start of the intervention, and at monthly time points until a resident dies or until the end of the intervention period (6 months). This will ensure a detailed understanding and, because of the mortality rates expected, minimize attrition. Data will be entered into a password protected anonymised database by the research team.

Quantitative analysis

We will use simple descriptive statistics to summarise process data and the outcomes collected by the ICL at the care home level (i.e. number of deaths in the last month etc.) We will describe the demographic and clinical characteristics of residents and family carers who participate in the data collection, as well as symptoms experienced, interventions received and any sentinel events. We will describe the symptom burden and quality of care received using SWEOLCD, QUALID. We will compare the scores to the results of our previous study in order to gain inferences on whether the enhanced care project makes a difference. The results will be summarised using mean and standard deviation or alternatives in case of non-normally distrusted data. Appropriate plots will also be produced.

Qualitative analysis

The interviews will be audio-taped, transcribed verbatim and entered onto a qualitative software programme (Atlas-ti) for the coding, management and retrieval of data. Transcripts will be analysed and coded using Thematic Analysis. The data analysis process will follow the guidelines provided by Braun and Clarke (45) to develop meaningful themes and a rigorous approach to data analysis will be adopted by working to the quality framework recommended by Spencer (46). Throughout the analytic process, the researchers will engage in ongoing reflection with the use of memoing and reflective diaries to engage with the data further and refine emergent themes. Data triangulation will be achieved by interviewing both family carers and care home staff from a variety of work roles (i.e., care home manager, health care assistant, nurse) to explore the facilitators and barriers to the implementation of the enhanced model of care from different perspectives.

For peer review only - http://bmjopen.bmj.com/site/about/guidelines.xhtml

Final analyses

After full data collection ends, we will undertake definitive analyses to detail the demographic features of the cohort and assess the symptom management and their health care needs (using Stirling, Waterlow, NPI and sentinel events), taking into account repeated measures on individual subjects. We shall describe the level and nature of unmet needs and examine descriptively (using mean and standard deviations or suitable alternatives in case of non-normally distributed data and graphs) how comfort and quality of life change over time (using PAINAD, SM-EOLD, SW-EOLD and QUALID). We will describe the trajectory of carer wellbeing (HADS and Zarit Buden Inteview) during their friend/relative's final stages of life with severe memory problems and how this may change if the resident dies, using plots of the of wellbeing over time.

Sample size

This is a pilot study and as such a formal power calculation is not appropriate. Numbers are chosen on pragmatic grounds as sufficient to demonstrate feasibility in terms of recruitment and acceptance of the intervention. We will aim to recruit 30 residents with severe memory problems from two care homes from which to collect individual outcome data.

Health economics

Health economic evaluation will consider resource allocation in caring for patients with severe problems and, where relevant, in their last 6 months of life, as well as the quality of life of their family carers and associated economic impact on these family carers in this period.

Data on resource and service use for people with severe memory problems (RUD-Lite) and economic burden on family carers (Zarit Burden Interview) will be collected both at baseline and monthly after the enhanced care project has been implemented. These data will be collated with unit costs data from *Unit Costs of Health and Social Care (2012)* (47;48) to obtain costs per patient from NHS (such as averted hospital admission, costs for a typical episode), costs from personal social services (such as training and education for care home staff) and costs from societal perspectives (such as local commissioners' decisions on scarce resource allocation, additional costs to public purse where caring responsibilities had been met by the state instead of family carers).

Economic evaluation of the quality-adjusted life years (QALYs) for family carers will utilize EQ-5D-5L instrument to assess if enhanced care project has resulted in greater utility attained for this group and associated cost-effectiveness.

PROJECT MANAGEMENT

For peer review only - http://bmjopen.bmj.com/site/about/guidelines.xhtml

Registration, sponsorship and indemnity

The project will be registered with the research departments at the participating CCG. University College London will be the project sponsor and provide insurance. The research team will obtain honorary clinical contracts for each participating CCG, adhering to the Marie Curie Palliative Care Research Unit's Lone Worker Policy (2012).

Data protection

Case Report Forms (CRF) for the study will be stored in accordance with the Declaration of Helsinki. Electronic data will be anonymised and stored on a password protected database. At the end of the study anonymised files will be stored securely in a secure UCL archiving facility.

Research network support

The programme has been adopted by the DeNDRoN (Dementias and Neurodegenerative Diseases Network)

Project Staffing

The person appointed to the ICL post will have extensive experience in the care of older people and their family carers in care home settings and with expertise in severe memory problems and social care. They will deliver the intervention with the core team. They will be supervised by the PI (Dr Louise Jones) and, given the nature of the work, offered supportive clinical supervision by Dr E Sampson. The ICL will receive training to acclimatise them to the care homes in which they will be working and familiarise them with the intervention manual. They will have a monthly meeting with the project team to check adherence to the principles of the manual and to make any necessary adaptations to this. Two clinical researchers, from the Marie Curie Palliative Care Research Unit, who have extensive clinical and research experience with both palliative care and people with severe memory problems and family carers will collect the individual data for the evaluation of the enhanced care intervention. The researchers have particular skills in interviewing bereaved family carers and relatives.

Core study team

Dr Louise Jones, Head of Unit, is PI and guarantor for the programme. She leads the Marie Curie palliative care research team at UCL. She is a palliative care physician and expert in qualitative and quantitative research in end of life care in a range of long term conditions. She has a long history of collaboration with other members of the team.

Dr Elizabeth Sampson is an international expert in end of life care research in dementia. She has expertise in epidemiology and old age psychiatry and leads the dementia research

For peer review only - http://bmjopen.bmj.com/site/about/guidelines.xhtml

group within the Marie Curie research team at UCL where she is deputy Head of Unit. She will lead this research programme and manage the research team.

Professor Michael King the director of the Division of Psychiatry at UCL, in which the Marie Curie Unit resides. He is co-director of PRIMENT Clinical Trials Unit which specialises in trials in mental health and primary care. He is expert in epidemiology, development and evaluation of complex health care interventions and clinical trials. He will provide expertise in particular for the development and testing of our intervention.

Professor Irwin Nazareth is professor of Primary Care and head of department of Primary Care and Population Health at UCL. He is co-director of PRIMENT Clinical Trials Unit. He is expert in epidemiology, development and testing of complex healthcare interventions.

Professor Stephen Morris is professor of Health Economics UCL. He is expert in economic evaluations of complex healthcare interventions and NHS databases and will provide expertise on health economics for all workstreams.

Professor Rumana Omar is professor in Biostatistics UCL and expert in analysing complex datasets where, because of the nature of the cohort under study, data may be missing.

Professor Gerard Leavey is a social scientist who is expert in qualitative research particularly in complex mental health conditions. He leads the Northern Ireland centre for mental health research and policy (NIAMH) and is academic lead for the Ulster hub of the All Ireland Institute for Palliative Care Research.

Membership of our expert steering group

We have convened an expert steering group that has met every six months throughout the programme. The core members of our research team bring expertise in end of life care, care of the elderly, old age psychiatry, health services research, epidemiology, primary care, social science, health economics and statistics. To complement this skill mix we have included a further range of expertise through the external membership of our expert steering group:

Experts in dementia care research- in secondary care - Professor Gill Livingston (UCL), and in primary care-Professor Louise Robinson (Newcastle)

Experts in end of life care: Min Stacpoole (Senior Nurse, St Christopher's Hospice), Claire Henry (Lead NHS National End of Life Programme), Karen Harrison-Dening (Consultant Admiral nurse, Dementia UK and dementia policy adviser to Marie Curie Cancer Care)

Experts in social care: Sharon Blackburn, Chief Executive, English Care Homes association (ECCA), Graham Stokes, BUPA, to represent the private sector

Expert by experience: Mr John Sprange

For peer review only - http://bmjopen.bmj.com/site/about/guidelines.xhtml

Patient and Public Involvement

Mr John Sprange will participate in our steering group. His input will be essential and we will encourage and facilitate him in this work through our local Camden Services User Research Forum (SURF).

STUDY OUTPUTS

Dissemination

We shall prepare documents for dissemination by end of life and dementia care organisations such as Marie Curie Cancer Care, BUPA, Dementia UK, The Alzheimer's Society, National End of Life Care programme and the government special advisor for dementia including detailed reports, scientific presentations and papers for peer reviewed journals, and publicise our findings on the Marie Curie website. A summary will be provided to all participants who would like to receive this.

REFERENCES

(1) Knapp M, Privette A. Dementia UK. London: Alzheimer's Society; 2007.

(2) Brayne C, Gao L, Dewey M, Matthews FE. Dementia before death in ageing societies--the promise of prevention and the reality. PLoS Med 2006 October;3(10):e397.

(3) Dewey ME, Saz P. Dementia, cognitive impairment and mortality in persons aged 65 and over living in the community: a systematic review of the literature. Int J Geriatr Psychiatry 2001 August;16(8):751-61.

(4) Sampson EL, Bulpitt CJ, Fletcher AE. Survival of community-dwelling older people: the effect of cognitive impairment and social engagement. J Am Geriatr Soc 2009 June;57(6):985-91.

(5) Reisberg B. Functional assessment staging (FAST). Psychopharmacol Bull 1988;24(4):653-9.

(6) Mitchell SL, Teno JM, Kiely DK, Shaffer ML, Jones RN, Prigerson HG et al. The clinical course of advanced dementia. N Engl J Med 2009 October 15;361(16):1529-38.

(7) McCarthy M, Addington-Hall J, Altmann D. The experience of dying with dementia: a retrospective study. International Journal of Geriatric Psychiatry 1997 March;12(3):404-9.

For peer review only - http://bmjopen.bmj.com/site/about/guidelines.xhtml

(8) Sampson EL, Blanchard MR, Jones L, Tookman A, King M. Dementia in the acute hospital: prospective cohort study of prevalence and mortality. Br J Psychiatry 2009 July;195(1):61-6.

(9) Sampson EL, Gould V, Lee D, Blanchard MR. Differences in care received by patients with and without dementia who died during acute hospital admission: a retrospective case note study. Age Ageing 2006 March;35(2):187-9.

(10) Davies, E. and Higginson, I. J. Better palliative care for older people. World Health Organisation Europe; 2004.

(11) Sanders S, Swails P. Caring for individuals with end-stage dementia at the end of life. Dementia 2009;8:117-38.

(12) McCarty CE, Volicer L. Hospice access for individuals with dementia. Am J Alzheimers Dis Other Demen 2009 December 20;24(6):476-85.

(13) Department of Health. End of life care strategy: Promoting high quality care for all adults at the end of life. London: Department of Health; 2008.

(14) Bourne, J. Improving services and support for people with dementia. National Audit Office; 2007.

(15) Sampson EL, Ritchie CW, Lai R, Raven PW, Blanchard MR. A systematic review of the scientific evidence for the efficacy of a palliative care approach in advanced dementia. Int Psychogeriatr 2005 March;17(1):31-40.

(16) Goodman C, Evans C, Wilcock J, Froggatt K, Drennan V, Sampson E et al. End of life care for community dwelling older people with dementia: an integrated review. Int J Geriatr Psychiatry 2009 August 17.

(17) The coalition: our programme for government. London: Cabinet Office; 2010.

(18) Fitch, K., Bernstein, S. J., Aguilar, M., Burnand, B., LaCalle, J., Lazaro, P., van het Loo, M., McDonnell, J., Vader, J. P., and Kahan, J. P. The Rand/UCLA appropriateness method user's manual. Santa Monica: RAND; 2001.

(19) Grol RP, Bosch MC, Hulscher ME, Eccles MP, Wensing M. Planning and studying improvement in patient care: the use of theoretical perspectives. Milbank Q 2007;85(1):93-138.

(20) Ferlie EB, Shortell SM. Improving the quality of health care in the United Kingdom and the United States: a framework for change. Milbank Q 2001;79(2):281-315.

(21) Greenhalgh T, Robert G, Macfarlane F, Bate P, Kyriakidou O. Diffusion of innovations in service organizations: systematic review and recommendations. Milbank Q 2004;82(4):581-629.

(22) American Psychiatric Association. Diagnostic and Statistical Manual of Mental Disorders. 4th Edition ed. Washington DC: American Psychiatric Association; 1994.

For peer review only - http://bmjopen.bmj.com/site/about/guidelines.xhtml

(23) Gold Standards Framework. The Gold Standards Framework. NHS End of Life Care Programme 2008;Available from: URL: http://www.goldstandardsframework.nhs.uk/

(24) Thuné-Boyle ICV, Sampson EL, Jones L, King M, Lee D, Blanchard MR. Challenges to improving end of life care of people with advanced dementia in the UK. Dementia 2010;9:285-98.

(25) Sampson EL, Jones L, Thune-Boyle IC, Kukkastenvehmas R, King M, Leurent B et al. Palliative assessment and advance care planning in severe dementia: an exploratory randomized controlled trial of a complex intervention. Palliat Med 2011 April;25(3):197-209.

(26) Walsh K, Jones L, Tookman A, Mason C, McLoughlin J, Blizard R et al. Reducing emotional distress in people caring for patients receiving specialist palliative care. Randomised trial. Br J Psychiatry 2007 February;190:142-7.

(27) Low J, Perry R, Wilkinson S. A qualitative evaluation of the impact of palliative care day services: the experiences of patients, informal carers, day unit managers and volunteer staff. Palliat Med 2005 January;19(1):65-70.

(28) Chambers DA, Glasgow RE, Stange KC. The dynamic sustainability framework: addressing the paradox of sustainment amid ongoing change. Implement Sci 2013;8:117.

(29) Charlson ME. Studies of prognosis: progress and pitfalls. J Gen Intern Med 1987 September;2(5):359-61.

(30) Volicer L, Hurley AC, Lathi DC, Kowall NW. Measurement of severity in advanced Alzheimer's disease. J Gerontol 1994 September;49(5):M223-M226.

(31) Waterlow J. Pressure sores: a risk assessment card. Nursing Times 1985;81(48):49-55.

(32) Reid J, Morison M. Classification of pressure sore severity. Nurs Times 1994 May 18;90(20):46-50.

(33) Zwakhalen SM, Hamers JP, bu-Saad HH, Berger MP. Pain in elderly people with severe dementia: a systematic review of behavioural pain assessment tools. BMC Geriatr 2006;6:3.

(34) Cohen-Mansfield J, Marx MS, Rosenthal AS. A description of agitation in a nursing home. J Gerontol 1989 May;44(3):M77-M84.

(35) Cummings JL, Mega M, Gray K, Rosenberg-Thompson S, Carusi DA, Gornbein J. The Neuropsychiatric Inventory: comprehensive assessment of psychopathology in dementia. Neurology 1994 December;44(12):2308-14.

(36) Kiely DK, Volicer L, Teno J, Jones RN, Prigerson HG, Mitchell SL. The validity and reliability of scales for the evaluation of end-of-life care in advanced dementia. Alzheimer Dis Assoc Disord 2006 July;20(3):176-81.

For peer review only - http://bmjopen.bmj.com/site/about/guidelines.xhtml

(37) Weiner MF, Martin-Cook K, Svetlik DA, Saine K, Foster B, Fontaine CS. The quality of life in late-stage dementia (QUALID) scale. J Am Med Dir Assoc 2000 May;1(3):114-6.

(38) Wimo A, Winblad B. Resource utilisation in dementia: RUD Lite. Brain Aging 2003;3:48-59.

(39) Zarit SH, Reever KE, Bach-Peterson J. Relatives of the impaired elderly: correlates of feelings of burden. Gerontologist 1980 December;20(6):649-55.

(40) Zigmond AS, Snaith RP. The hospital anxiety and depression scale. Acta Psychiatr Scand 1983 June;67(6):361-70.

(41) Herdman M, Gudex C, Lloyd A, Janssen M, Kind P, Parkin D et al. Development and preliminary testing of the new five-level version of EQ-5D (EQ-5D-5L). Qual Life Res 2011 December;20(10):1727-36.

(42) Engel SE, Kiely DK, Mitchell SL. Satisfaction with end-of-life care for nursing home residents with advanced dementia. J Am Geriatr Soc 2006 October;54(10):1567-72.

(43) Mitchell SL, Kiely DK, Jones RN, Prigerson H, Volicer L, Teno JM. Advanced dementia research in the nursing home: the CASCADE study. Alzheimer Dis Assoc Disord 2006 July;20(3):166-75.

(44) Sampson EL, Harrison-Dening K, Greenish W, Mandal U, Holman A, Jones L. End of Life Care for People with Dementia. Marie Curie Cancer Care 2009;Available from: URL: http://www.mariecurie.org.uk/forhealthcareprofessionals/end-of-life-dementia.htm

(45) Braun V, Clarke V. Using thematic analysis in psychology . Qual Res Psychol 2006;3(2):77-101.

(46) Spencer, L., Ritchie, J., Lewis, J., and Dillon, L. Quality in qualitative evaluation: A framework for assessing research evidence. London: The Cabinet Office; 2003.

(47) Publications and statistics. Department of Health 2010;Available from: URL: www.dh.gov.uk/en/Publicationsandstatistics/Publications/PublicationsPolicyAndGuidance

(48) Curtis L. PSSRU 2009;Available from: URL: http://www.pssru.ac.uk/uc/uc2009contents.htm

For peer review only - http://bmjopen.bmj.com/site/about/guidelines.xhtml

APPENDICES

Appendix 1: Compassion intervention manual

The Final Compassion Intervention Manual will be published with free access on the Marie Curie website (www.mariecurie.org.uk).

Appendix 2. Bedford Alzheimer Nursing Severity (BANS) scale

Please refer to: Volicer L, Hurley AC, Lathi DC, Kowall NW. Measurement of severity in advanced Alzheimer's disease. J Gerontol 1994 September;49(5):M223-M226.

Appendix 3: Waterlow scale

Please refer to: Waterlow J. Pressure sores: a risk assessment card. Nursing Times 1985;81(48):49-55.

Appendix 4: Stirling Wound Assessment Scale

Please refer to Reid J, Morison M. Classification of pressure sore severity. Nurs Times 1994 May 18;90(20):46-50.

Appendix 5: Pain Assessment in Advanced Dementia (PAIND)

Please refer to: Zwakhalen SM, Hamers JP, bu-Saad HH, Berger MP. Pain in elderly people with severe dementia: a systematic review of behavioural pain assessment tools. BMC Geriatr 2006;6:3.

Appendix 6: Cohen Mansfield Agitation Inventory (CMAI)

Please refer to: Cohen-Mansfield J, Marx MS, Rosenthal AS. A description of agitation in a nursing home. J Gerontol 1989 May;44(3):M77-M84.

Appendix 7: Neuropsychiatric Inventory (NPI) questionnaire

Please refer to: Cummings JL, Mega M, Gray K, Rosenberg-Thompson S, Carusi DA, Gornbein J. The Neuropsychiatric Inventory: comprehensive assessment of psychopathology in dementia. Neurology 1994 December;44(12):2308-14.

Appendix 8: Symptom Management at the End Of Life in Dementia (SM-EOLD) scale

Please refer to: Kiely DK, Volicer L, Teno J, Jones RN, Prigerson HG, Mitchell SL. The validity and reliability of scales for the evaluation of end-of-life care in advanced dementia. Alzheimer Dis Assoc Disord 2006 July;20(3):176-81.

Appendix 9: Quality of Life in late-stage Dementia (QUALID)

Please refer to: Weiner MF, Martin-Cook K, Svetlik DA, Saine K, Foster B, Fontaine CS. The quality of life in late-stage dementia (QUALID) scale. J Am Med Dir Assoc 2000 May;1(3):114-6.

For peer review only - http://bmjopen.bmj.com/site/about/guidelines.xhtml

For peer review only

Appendix 10: Resource Utilisation in Dementia (RUD) - Lite

Please refer to: Wimo A, Winblad B. Resource utilisation in dementia: RUD Lite. Brain Aging 2003;3:48-59.

Appendix 11: The Comfort Assessment in Dying with Dementia scale (CAD-EOLD)

Please refer to: Kiely DK, Volicer L, Teno J, Jones RN, Prigerson HG, Mitchell SL. The validity and reliability of scales for the evaluation of end-of-life care in advanced dementia. Alzheimer Dis Assoc Disord 2006 July;20(3):176-81.

Appendix 12: The Zarit Burden Interview

Please refer to: Zarit SH, Reever KE, Bach-Peterson J. Relatives of the impaired elderly: correlates of feelings of burden. Gerontologist 1980 December;20(6):649-55.

Appendix 13: Hospital Anxiety and Depression Scale (HADS)

Please refer to: Zigmond AS, Snaith RP. The hospital anxiety and depression scale. Acta Psychiatr Scand 1983 June;67(6):361-70.

Appendix 14: The Satisfaction with Care at the End-of-Life in Dementia Questionnaire (SWC/CAD-EOLD)

Please refer to: Kiely DK, Volicer L, Teno J, Jones RN, Prigerson HG, Mitchell SL. The validity and reliability of scales for the evaluation of end-of-life care in advanced dementia. Alzheimer Dis Assoc Disord 2006 July;20(3):176-81.

Appendix 15: EQ-5D-5L

Please refer to: Herdman M, Gudex C, Lloyd A, Janssen M, Kind P, Parkin D et al. Development and preliminary testing of the new five-level version of EQ-5D (EQ-5D-5L). Qual Life Res 2011 December;20(10):1727-36.

For peer review only

Appendix 16: Health Care Professional Qualitative Interview Schedule

HCP interview schedule Compassion Study (HCP interview schedule for intervention V1 09.01.2014)

Preamble

Thank you for agreeing to this interview. As you know we have introduced an Interdisciplinary Care Leader into the care home in which you work. The reason why we have invited you today for this discussion is to understand what your thoughts are on this service and if there was anything about this service that you think can be improved. Also, please be assured that the topics that we discuss today are strictly confidential and will remain completely anonymous.

Interview

Firstly, just for the purposes of the recording can you:

1. Describe your current role here

2. The type and amount of contact you have on a day to day basis with residents with severe memory problems (how severe these are, their roles and responsibilities)

Now I would like to talk about the role of the ICL and how it may have influenced the way you perform your job:

3. Tell me about how you found working with the ICL

4. Did the ICL influence the way you performed your role? If so, how? Can you provide some examples of how the ICL did this?

5. Do you think the ICL changed the care you provided to residents?

For peer review only - http://bmjopen.bmj.com/site/about/guidelines.xhtml

The Compassion Programme: WS3- pilot study Version 1, 24th January 2014

6. Did you find that it influenced any of the following:
    a. Your knowledge of dementia
    b. How you assess patients with severe memory problems
    c. How you recognise symptoms such as pain and how you manage these symptoms?
    d. Were you given any support and guidance on initiating and implementing advance care plans? If so, can you give us an example of when this happened?
    e. The way you communicate/interact with patients who are no longer able to communicate
    f. How comfortable you are about communicating with family members, including discussions about palliative care and death/dying
    g. How you communicate with other HCPs

7. Tell me about your needs. Did the ICL influence the support that you receive in your role?
    a. E.g., such as support following patient death

For care home manager: Did you notice any changes in the way your staff provided care to patients? How do you feel the ICL was received by your staff?

8. Is there anything about this service that can be improved? Is there anything that you would do differently if you were implementing this service?

For peer review only - http://bmjopen.bmj.com/site/about/guidelines.xhtml

Appendix 17: Family Carer Qualitative Interview Schedule

Family carer interview schedule (Compassion Study - Carer interview schedule for intervention V1 09.01.2014)

Preamble

Thank you for agreeing to this interview. The reason that we have invited you along for this discussion is to get an idea of the care and support that you and your relative have received over the last few months. If you feel that you need to stop or leave the room at any time please tell me. Whatever you tell me will be made anonymous for the purposes of the study.

Interview

I'd like to begin by asking you a little bit about X memory problems and your understanding of his/her illness

1. Tell me about X's illness and symptoms over the last few months
    a. Both physiological and psychological needs

2. Tell me about the types of support or services has X received over the last few months
    a. Formal or informal (Religion/spirituality)
    b. Satisfaction

Now I'd like to ask you some questions about your needs as a carer:

3. How have you found dealing with X's illness over the last few months? What have you found particularly difficult?
    a) Both physiological and psychological needs
    b) Own mental health

4. Tell me about the support that you needed including emotional, psychological and social needs religious/spiritual needs. Were your needs assessed?

For peer review only - http://bmjopen.bmj.com/site/about/guidelines.xhtml

a. If so, tell me about the services that you were offered to meet these needs (If yes, determine who this was offered by and when this took place)

5. Did you have any discussions with HCP's (GP, Consultant, nursing home staff etc) about (if yes, determine when these took place):
    a. Course of illness
    b. Additional information
    c. Treatments – decision making – past and future
    d. Inclusion of other family members

6. Has anyone discussed your thoughts if X's condition were to deteriorate? If so, who discussed these with you and when?

    a) POA
    b) DNAR
    c) Place of death
    d) ACP – Feasibility of carrying out another person's wishes

7. Has anyone discussed what the future holds for X?
    a. i.e., religious beliefs/spirituality – Any recognition in the home?

8. We would also like to find out if the ICL has influenced the care and support that you and your relative have received over the last few months.
    a. Tell me about any changes to the care and support that both you and X have received over the last few months
    b. Tell me if these changes had a positive or a negative impact on you and X
    c. Ways in which we can improve this service? How else can the ICL help you and your relative?

Additional question if patient has passed away: Can you tell me a little about what happened when X passed away?

For peer review only - http://bmjopen.bmj.com/site/about/guidelines.xhtml

a) Did it all go smoothly?

b) Were their end of life wishes met? (such as religious/spiritual wishes)

c) Did you receive immediate and ongoing bereavement, emotional and spiritual support?

For peer review only

Evaluation of the implementation of the COMPASSION intervention to improve care towards the end of life for people with advanced dementia residing in two care homes in north London: assessment of long term effects, maintenance and sustainability.

The COMPASSION programme research team:

L Jones, E L Sampson, K Moore, M Elliott , N Kupeli, S Davis, J Harrington, B Candy, V Vickerstaff, A Gola, M King, G Leavey,S Morris, I Nazareth, R Z Omar

Background

The COMPASSION intervention (available from the authors) was developed through a 3 year NIHR portfolio research programme funded by Marie Curie Cancer Care (Jones et al 2012) and it aims to improve end of life care for people with advanced dementia. In the final year of the programme, COMPASSION was implemented, in two care homes in two different clinical commissioning groups, in north London in an exploratory study (ref Elliott 2014).

COMPASSION consists of two key components enabled by an interdisciplinary care leader (ICL) working with the multidisciplinary team within the care home and with associated primary and secondary care providers. These components are: (i) facilitation of integrated care (ii) provision of training and support for care home staff and family carers. We anticipate that there will be ripple and diffusion effects that will influence a third component which is the wider political, economic and commissioning environment within each clinical commissioning group.

The two study sites differed in their level of readiness for receipt of the intervention: service provision for care at the end of life for people with advanced dementia was thought to be more developed at the Camden care home. The exploratory study commenced between May and June 2014 and lasted for 6 months at each site.

An important part of understanding the effects of complex healthcare interventions is collecting evidence on their long term effects, both positive and negative, checking for evidence of potential harms, and what factors are affecting maintenance of any change exerted by the intervention (MRC 2008). Much thought has been given to how maintenance and sustainability might be assessed. In a recent paper, Chambers et al 2013 suggest that when an innovation team leaves a test site, it becomes difficult for the routine service providers to adhere to the new model as closely and ‘programme drift’ and ‘voltage drop’ (reduced adherence to protocols) are natural and inevitable processes. However, they argue that each site may adapt what they have learned from the innovation and continue to behave in newly adapted ways that are sympathetic to their own particular context. Thus those components of an intervention that are effective and workable will vary between sites. It is likely that, given this flexibility, such mechanisms are most likely to lead towards the aims and objectives of the intervention or innovative model of care.

Aims

We aim to assess the longer term effects of implementation of COMPASSION at two care home sites by understanding the impact of the intervention on members of the multidisciplinary team involved in the care of residents with advanced dementia.

For peer review only - http://bmjopen.bmj.com/site/about/guidelines.xhtml

Design

We shall collect qualitative data from a purposive sample of health and social care professionals in the care home and in associated primary and secondary care services. We shall seek to understand any alterations in how services are organized and resources allocated (such as changes in staffing levels, engagement of the multi-disciplinary team across primary and secondary care) that have occurred since the COMPASSION exploratory intervention team exited the site. We shall use a realist approach to analyzing the data to enable an understanding of the contexts and mechanisms that are operating that are likely to affect outcomes in the care of people with advanced dementia (Pawson and Tilley 1997). We shall consider the mechanisms at the 4 levels recommended in the study of organizational change: individual, group, organizational, and wider economic and political context (Ferlie and Shortell 2001; Grol 2007)

Study setting

2 care homes in North London, UK. BLINDED TO MEET ETHICAL REQUIREMENTS STATED BELOW.

Sample

A maximum of 10 health and social care providers at each site. We shall attempt to approach professionals who have previously been interviewed as part the piloting of our intervention in an exploratory study (Elliott 2014). Where there has been staff turnover, we shall attempt to interview the newly hired personnel. We expect our sample to include health care assistants, trained nursing staff, allied health professionals, social care professionals, care home managers, general practitioners, and members of specialist services such as community palliative care, geriatricians and mental health providers.

Procedures

Participants will be given an information sheet and at least 48 hours to consider whether they wish to take part. Those who agree will be asked to give informed consent to two in-depth qualitative interviews that will be audio-taped and transcribed verbatim. The first interview will take place 4 months after the COMPASSION exploratory team left the site; the second after a further 4 months. Interviews will last between 15-60 minutes. We shall work to a topic guide and our focus will be on understanding the experience of the intervention, whether and how it has affected practice, whether and how it has affected behaviours of individuals and teams, whether and how it has been thought to influence care. We shall explore with care home managers whether there have been changes in resource allocation, service organization and personnel, and whether there have been any effects on the behaviours of the care home owners. In speaking with any newly hired personnel we shall attempt to understand whether any of the effects of COMPASSION are thought to have diffused into their training and practice. In this way we hope to gain an understanding of whether components of COMPASSION have started to become embedded in the culture of each care home.

Data will be collected by a member of the research team who was not involved in the implementation of the COMPASSION intervention.

For peer review only - http://bmjopen.bmj.com/site/about/guidelines.xhtml

Analysis

Transcribed interviews will be read and coded for emergent themes using framework analysis (Ritchie and Spencer 1993). Coding and themes will be checked by a second member of the research team. We shall then hold meetings of the wider research team, including those involved in implementation in the exploratory study, to discuss what themes are emerging and categorise them to understand the contexts, mechanisms and outcomes that are operating. We shall use these data to develop a realist programme theory for sustainability of COMPASSION.

We shall consider data collected at four months to develop a provisional programme sustainability theory, and this will be refined in an iterative process using data collected at eight months. See Figure 1 below.

Figure 1. Realist analysis of data and development of programme theories

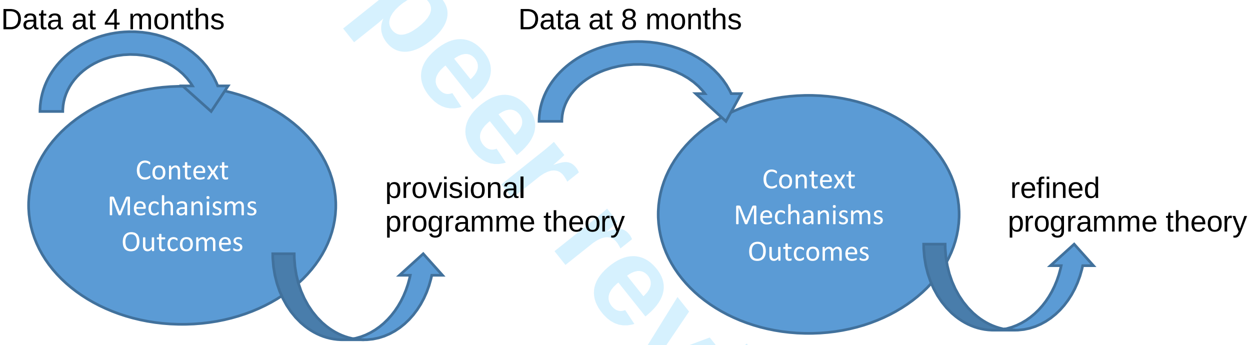

We shall merge these data with qualitative data collected from similar health and social care professionals during the exploratory study to refine an overall programme theory of how COMPASSION has operated throughout its implementation and beyond.

We shall attempt to understand which components of COMPASSION are key to its implementation and which sections of the intervention manual are followed most closely. We shall attempt to describe and understand the reasons for programme drift and voltage drop described by Chambers 2013. We shall consider how our data inform further amendments to the structure and content of COMPASSION and the role of the ICL who was the key implementation person working at each site during the exploratory study. This will allow us to adapt and tailor the intervention manual accordingly.

Economic considerations

We shall not collect any economic data directly. However, we shall use the understanding gained from the qualitative data and work with the health economist within our wider research team to explore how COMPASSION components 1 and 2 have influenced attitudes to commissioning and the wider economic and political context within each participating clinical commissioning group. We shall use refinements we make to the COMPASSION manual to consider the costs of the core components that we retain and whether resource allocation has altered since the intervention ceased. This will inform recommendations for further roll out of the intervention at other sites and for consideration by service planners and providers in the

For peer review only - http://bmjopen.bmj.com/site/about/guidelines.xhtml

clinical commissioning groups, the care home provision system and providers of end of life care and care of people with dementia in the NHS and the voluntary sector.

Ethical issues

Data collection in this work will involve health and social care professionals only who will be given information sheets in advance of giving written informed consent for participation in audio-taped interviews. All data will be anonymized and no individual or research site will be identifiable in reports or publications arising from the work.

Data will be kept in locked cabinets using usual procedures within the research department and all procedures will conform to the Data Protection Act.

Plans for dissemination

Findings from this work will be prepared for publication at national and international conferences, in scientific journals and as part of policy documents prepared by organisations involved in dementia and end of life care such as the Alzheimer's Society and Marie Curie.

Findings will be merged with other data arising from the COMPASSION programme. Learning from the programme will be used within the MARQUE programme, funded by ESRC and NIHR in workstreams led by our research team. MARQUE is one of the tranches of work arising from the UK Prime Minister's Dementia Challenge 2013.

References

Chambers DA, Glasgow RE, Stange KC. The dynamic sustainability framework: addressing the paradox of sustainment amid ongoing change. Implement Sci. 2013 Oct 2;8:117. doi: 10.1186/1748-5908-8-117.

Craig P, Dieppe P, Macintyre S, et al. Developing and evaluating complex interventions: the new Medical Research Council guidance. BMJ 2008;337:979–83

Elliot, M., Harrington, J., Moore, K., Davis, S., Kupeli, N., Vickerstaff, V. H., Gola, A., Candy, B., Sampson, E. L., Jones, L. (2014). A protocol for an exploratory phase i mixed-methods study of enhanced integrated care for care home residents with advanced dementia: The compassion intervention. *BMJ Open* doi:10.1136/bmjopen-2014-005661

Grol RPTM, Bosch MC, Hulscher MEJL, Eccles MP, Wensing M. Planning and stuying improvement in patient care: the use of theoretical perspectives. Millbank quarterly Vol 85 No1 2007 (pp93-138)

Jones L, Harrington J, Scott S, et al. CoMPASs: IOn programme (Care of Memory Problems in Advanced Stages of dementia: Improving Our Knowledge): protocol for a mixed methods study. BMJ Open 2012;2:e002265. doi:10.1136/bmjopen-2012-002265

R Pawson and N Tilley (1997) xvii + 235p *REALISTIC EVALUATION*, London: Sage.

Spencer L, Ritchie J, Lewis J, *et al. Quality in qualitative evaluation: A framework for assessing research evidence.* London: The Cabinet Office; 2003. http://www.civilservice.gov.uk/wp-content/uploads/2011/09/a_quality_framework_tcm6-38740.pdf (accessed May 2014)

For peer review only - http://bmjopen.bmj.com/site/about/guidelines.xhtml

Supplementary File 3: Context of each NH

Level	Both NHs	
**Political and economic context (health and social care system in the UK)**	• Both NHs located in north London<br>• Despite policy attempts to integrate services (e.g. Better Care Fund), funding and management of social services are separate from the National Health Service (NHS)<br>• NHs operate within the social service system<br>• The majority of NHs are privately run entities operating for profit<br>• Residents are assessed for eligibility for continuing care funding from their local authority or pay privately for social care<br>• Clinical Commissioning Groups (CCGs) manage priorities for funding of healthcare services and operate locally. The two NHs were located within different CCGs. UK residents are entitled to services through the NHS<br>• Other specialist and allied health services should be available in all NHs, however, access and availability can be uneven[1]<br>• NHs do not require a nurse to be employed, unless beds are allocated as nursing home beds<br>• Some NH beds are also allocated as dementia specific, requiring the NH to have staff with expertise in dementia care	
**Organisational context**	Both NHs privately run by larger companies operating multiple NHs.	
**CCG context**	**Camden CCG**	**Barnet CCG**
Index of Multiple Deprivation#[2]	• 3rd decile of relative deprivation	• 6th decile of relative deprivation
Number of NHs in CCG*	• 13 NHs and care homes (not 24 hour nursing support)	• 91 NHs and care homes (not 24 hour nursing support)
Relevant CCG priorities	• 'Frail and elderly' programme<br>• 'Long term conditions and Cancer' programme[3]	• 'End of life' priority[4]
**Individual NH context**	**NH1**	**NH2**
Beds and levels of care	99 nursing home beds across five units including one dementia specific unit and one younger people with disabilities (not engaged in Intervention)	77 beds with three units: residential care and two nursing care units, one was dementia specific.
Management	Manager and deputy manager. Deputy manager retired half way through implementation.	Manager and Deputy manager. Deputy manager resigned in the weeks prior to implementation.
Nursing and healthcare assistants	Each unit managed by a nurse 24 hours a day with up to five healthcare assistants. Staff involved in direct care work 12hr shifts from 8:00-20:00 or 20:00-8:00	Both nursing units managed by a nurse with up to five healthcare assistants.
Activity co-ordinator	3 part-time staff (approx. 2 full time equivalent)	1 full time
External healthcare professionals		

For peer review only - http://bmjopen.bmj.com/site/about/guidelines.xhtml

Level	Both NHs	
GP	All residents registered with one GP clinic. Regular GP visits for 2X3hr sessions per week.	Residents registered with one GP clinic. Regular GP visits for 1X3hr session per week.
Actively involved at NH	Dietetics/nutrition, Geriatrics, Nursing (palliative care; tissue viability; Mental Health), Occupational Therapy, Physiotherapy (although long waiting lists are a deterrent), Podiatry, Social Work, Speech and Language Therapy, Hospital programme facilitating safe discharge from emergency department for complex and frail older patients.	Speech and Language Therapy, Old Age Psychiatry, district nursing (for non-nursing unit)
Available if required	Old Age Psychiatry, psychology	Nursing (palliative care and mental health)
Not available	Care of the Elderly	Geriatrics
Care planning	Care plans are monitored on a monthly basis by the nurse. They are kept as paper based records in the relevant nurse's office. There are templates for different areas of care. Examples of assessments used include: Abbey Pain scale; Doloplus 2, Cornell Depression Scale and Geriatric Depression Scale, Malnutrition Universal Screening Tool, Waterlow Pressure Ulcer Risk, Bradford Dementia Group Wellbeing Profile. Residents typically have 14-20 different care plans. Sentinel events or a significant change in condition will lead to a review and potentially instigating a new care plan as indicated.	Care plans are monitored on a monthly basis by the nurse. They are kept as paper based records in the relevant nurse's office. The template includes 25 different care needs.
Communication processes	• Documentation is manually recorded. Only the manager enters data for generating report back to the NH company.<br>• Verbal handover occurs twice daily during change of shift.<br>• Offer meetings for family members; recent poor attendance was leading the manager to query continued value.<br>• Nurses communicate with other nurses on the same floor working on different shifts using a communication book.<br>• Care plans include communication pages to report when healthcare professionals or family members have had discussions/appointments with NH staff.	• Documentation is manually recorded.<br>• No central place for recording deaths, hospitalisations or other adverse events.<br>• Nurses report in resident care plan on a daily basis and review care plans on a monthly basis. Nurses keep dairies to record resident medical appointments etc.<br>• Handover occurs at staff changeover.<br>• Regular family meetings are held.
Training and professional development	• 40 care staff have National Vocational Qualifications; 20 enrolled in health and social care training.<br>• Electronic matrix shows when each staff member completed compulsory and non-compulsory training flagging those who are due. There are 11 mandated competencies reviewed regularly.<br>• Training sessions rune on a regular basis – staff are informed via flyers in each unit. Sessions are scheduled at a set hour that is the quietest in the afternoon. Expectation that up to half of the staff currently working are given the opportunity to attend.	• No formalised structure for running regular training programmes. Training no longer offered via local palliative care service.<br>• A multi-day dementia training programme was run on an annual for a small number of staff to complete.

For peer review only

For peer review only - http://bmjopen.bmj.com/site/about/guidelines.xhtml

Level	Both NHs	
	• Access to training is not available to staff who have been in the country for less than three years. This can be a barrier for upskilling staff.	
Dementia and palliative care	• An advance care plan is developed on admission.<br>• Specialist Palliative Care Nurse from the local hospital's community palliative care nursing service visits the NH regularly and manages complex symptoms at EOL and provides staff training in palliative care. Links commenced 6-7 years earlier when palliative care felt that the NH's referrals were low or inappropriate.<br>• Use local electronic register to inform emergency and out-of-hour services about residents at the EOL and documented care wishes such as 'Do Not Attempt Resuscitation'<br>• 60 nursing and care staff were enrolled (prior to Intervention) in a distant education course about dementia.<br>• The manager attends local dementia strategy meetings.<br>• Manager frustrated by lack of consensus on best care in dementia. Manager felt staff needed more understanding of biological processes in dementia to help understand why a resident is acting the way they are.<br>• Annual memorial function with religious service; family of deceased residents invited.<br>• Two nurses (prior to the Intervention) were attending Gold Standards Accreditation training. Accreditation not achieved during implementation.<br>• During implementation it became evident that there were a range of staff development needs to build skills in dementia and palliative care	• They use the Gold Standards Framework to assess whether residents are nearing EOL. Specialist palliative care specifies that they are only to be called for 'signs and symptoms' (primarily pain management).<br>• Prior to the Intervention they had introduced forms regarding Power of Attorney, the Mental Capacity Act and deprivation of liberty.<br>• Staff have completed syringe driver training; no team available to provide IV antibiotics.<br>• One nurse (prior to the Intervention) attending Gold Standards Accreditation training with the goal of achieving accreditation. Not achieved during implementation.<br>• During implementation it became evident that there were a range of staff development needs to build skills in dementia and palliative care

1st decile = most deprived

* Source: http://www.carehome.co.uk/care_search.cfm (accessed 20th October 2016)

References

1. Seymour JE, Kumar A, Froggatt K. Do nursing homes for older people have the support they need to provide end-of-life care? A mixed methods enquiry in England. *Palliative medicine* 2011;25(2):125-38 doi: doi: 10.1177/0269216310387964published Online First: Epub Date]|.
2. Department for Communities and Local Government. English Indices of Deprivation 2015. Open Government License 2015.
3. NHS Camden Clinical Commissioning Group. 2014/15 Final Annual Report and Outcomes: Working with the people of Camden to achieve the best health for all, 2015.
4. NHS Barnet Clinical Commissioning Group. Annual Report and Accounts 2014/15: Working with local people to develop seamless, accessible care for a healthier Barnet, 2015.

For peer review only - http://bmjopen.bmj.com/site/about/guidelines.xhtml

For peer review only

Supplementary File 4: ICL time spent by activity by hours

Activity	NH1 hours (%)	NH2 hours (%)	Hours not attributable to a NH (%)	Total hours (%)	Total Costs*
Assessing needs					
Assessing needs**	122.75 (44)	87.75 (40)	NA	210.5 (32)	£6,241
Meeting family	9.75 (3)	14 (6)	NA	23.75 (4)	£665
Meeting staff	21.75 (8)	16.25 (7)	NA	38 (6)	£1,064
Emails/phone calls^	24 (9)	14.25 (6)	5.75 (4)	44 (7)	£869#
Core meetings	10.25 (4)	5.75 (3)	NA	16 (3)	£448
Wider Meetings	7.5 (3)	NA	NA	7.5 (1)	£210
Staff training					
Preparing training	19 (7)	34.25 (16)	26.75 (17)	80 (12)	£1,753
Providing training	14.25 (5)	19.25 (9)	NA	33.5 (5)	£1,019
Other					
Travel	47.25 (17)	29.75 (13)	30 (19)	107 (16)	£4,053***
ICL professional development	NA	NA	67 (42)	67 (10)	£1,468
ICL clinical supervision	NA	NA	28.75 (18)	28.75 (4)	£463
Total	276.5 (100)	221.25 (100)	158.25 (100)	656 (100)	£18,255

*Source for hourly rate: Department of Health and Health Education England, includes on-costs
**Includes unproductive time in the NH such as waiting to speak to staff, trying to locate staff or records etc.
***Includes cost of train fare
#excludes cost of telephone calls
^includes time speaking with or sending emails to family members
NA = not applicable

For peer review only - http://bmjopen.bmj.com/site/about/guidelines.xhtml

TREND Statement Checklist

Paper Section/ Topic	Item No	Descriptor	Reported? ✓	Pg #
**Title and Abstract**				
Title and Abstract	1	• Information on how unit were allocated to interventions	NA	
		• Structured abstract recommended	✓	2,3
		• Information on target population or study sample	✓	2
**Introduction**				
Background	2	• Scientific background and explanation of rationale	✓	4,5
		• Theories used in designing behavioral interventions	✓	5,8
**Methods**				
Participants	3	• Eligibility criteria for participants, including criteria at different levels in recruitment/sampling plan (e.g., cities, clinics, subjects)	✓	5
		• Method of recruitment (e.g., referral, self-selection), including the sampling method if a systematic sampling plan was implemented	✓	10,11
		• Recruitment setting	✓	9, 13,14
		• Settings and locations where the data were collected	✓	9,13,14
Interventions	4	• Details of the interventions intended for each study condition and how and when they were actually administered, specifically including:		
		○ Content: what was given?	✓	5-7
		○ Delivery method: how was the content given?	✓	8,9
		○ Unit of delivery: how were the subjects grouped during delivery?	NA	
		○ Deliverer: who delivered the intervention?	✓	8,9
		○ Setting: where was the intervention delivered?	✓	9,13,14
		○ Exposure quantity and duration: how many sessions or episodes or events were intended to be delivered? How long were they intended to last?	✓	6-9
		○ Time span: how long was it intended to take to deliver the intervention to each unit?	✓	9
		○ Activities to increase compliance or adherence (e.g., incentives)	✓	14-17
Objectives	5	• Specific objectives and hypotheses	✓	7,8
Outcomes	6	• Clearly defined primary and secondary outcome measures	✓	9,10
		• Methods used to collect data and any methods used to enhance the quality of measurements	✓	9-11
		• Information on validated instruments such as psychometric and biometric properties	✓	10,22,23
Sample Size	7	• How sample size was determined and, when applicable, explanation of any interim analyses and stopping rules	✓	9
Assignment Method	8	• Unit of assignment (the unit being assigned to study condition, e.g., individual, group, community)	✓	8,9
		• Method used to assign units to study conditions, including details of any restriction (e.g., blocking, stratification, minimization)	NA	
		• Inclusion of aspects employed to help minimize potential bias induced due to non-randomization (e.g., matching)	NA	

For peer review only

TREND Statement Checklist

Blinding (masking)	9	• Whether or not participants, those administering the interventions, and those assessing the outcomes were blinded to study condition assignment; if so, statement regarding how the blinding was accomplished and how it was assessed.	✓	9
Unit of Analysis	10	• Description of the smallest unit that is being analyzed to assess intervention effects (e.g., individual, group, or community)	✓	11,12
		• If the unit of analysis differs from the unit of assignment, the analytical method used to account for this (e.g., adjusting the standard error estimates by the design effect or using multilevel analysis)	NA	
Statistical Methods	11	• Statistical methods used to compare study groups for primary methods outcome(s), including complex methods of correlated data	✓	11,12
		• Statistical methods used for additional analyses, such as a subgroup analyses and adjusted analysis	NA	
		• Methods for imputing missing data, if used	NA	
		• Statistical software or programs used	✓	12
**Results**				
Participant flow	12	• Flow of participants through each stage of the study: enrollment, assignment, allocation, and intervention exposure, follow-up, analysis (a diagram is strongly recommended)	✓	Fig 1
		○ Enrollment: the numbers of participants screened for eligibility, found to be eligible or not eligible, declined to be enrolled, and enrolled in the study	✓	Fig 1
		○ Assignment: the numbers of participants assigned to a study condition	✓	Fig 1
		○ Allocation and intervention exposure: the number of participants assigned to each study condition and the number of participants who received each intervention	✓	Fig 1
		○ Follow-up: the number of participants who completed the follow-up or did not complete the follow-up (i.e., lost to follow-up), by study condition	✓	Fig 1
		○ Analysis: the number of participants included in or excluded from the main analysis, by study condition	✓	Fig 1
		• Description of protocol deviations from study as planned, along with reasons	NA	
Recruitment	13	• Dates defining the periods of recruitment and follow-up	✓	9
Baseline Data	14	• Baseline demographic and clinical characteristics of participants in each study condition	✓	22
		• Baseline characteristics for each study condition relevant to specific disease prevention research	✓	22
		• Baseline comparisons of those lost to follow-up and those retained, overall and by study condition	NA	
		• Comparison between study population at baseline and target population of interest	NA	
Baseline equivalence	15	• Data on study group equivalence at baseline and statistical methods used to control for baseline differences	NA	

For peer review only

TREND Statement Checklist

Numbers analyzed	16	• Number of participants (denominator) included in each analysis for each study condition, particularly when the denominators change for different outcomes; statement of the results in absolute numbers when feasible	✓	Fig 1
		• Indication of whether the analysis strategy was "intention to treat" or, if not, description of how non-compliers were treated in the analyses	NA	
Outcomes and estimation	17	• For each primary and secondary outcome, a summary of results for each estimation study condition, and the estimated effect size and a confidence interval to indicate the precision	✓	22,23
		• Inclusion of null and negative findings	NA	
		• Inclusion of results from testing pre-specified causal pathways through which the intervention was intended to operate, if any	NA	
Ancillary analyses	18	• Summary of other analyses performed, including subgroup or restricted analyses, indicating which are pre-specified or exploratory	NA	
Adverse events	19	• Summary of all important adverse events or unintended effects in each study condition (including summary measures, effect size estimates, and confidence intervals)	NA	
**DISCUSSION**				
Interpretation	20	• Interpretation of the results, taking into account study hypotheses, sources of potential bias, imprecision of measures, multiplicative analyses, and other limitations or weaknesses of the study	✓	24-27
		• Discussion of results taking into account the mechanism by which the intervention was intended to work (causal pathways) or alternative mechanisms or explanations	✓	24,25
		• Discussion of the success of and barriers to implementing the intervention, fidelity of implementation	✓	24-27
		• Discussion of research, programmatic, or policy implications	✓	26-27
Generalizability	21	• Generalizability (external validity) of the trial findings, taking into account the study population, the characteristics of the intervention, length of follow-up, incentives, compliance rates, specific sites/settings involved in the study, and other contextual issues	✓	25-26
Overall Evidence	22	• General interpretation of the results in the context of current evidence and current theory	✓	24-27

From: Des Jarlais, D. C., Lyles, C., Crepaz, N., & the Trend Group (2004). Improving the reporting quality of nonrandomized evaluations of behavioral and public health interventions: The TREND statement. *American Journal of Public Health*, 94, 361-366. For more information, visit: http://www.cdc.gov/trendstatement/

For peer review only

Implementing the Compassion Intervention, a model for integrated care for people with advanced dementia towards the end of life in nursing homes: A naturalistic feasibility study

Journal:	*BMJ Open*
Manuscript ID	bmjopen-2016-015515.R1
Article Type:	Research
Date Submitted by the Author:	28-Mar-2017
Complete List of Authors:	Moore, Kirsten; University College London, Marie Curie Palliative Care Research Department<br>Candy, Bridget; University College London, Marie Curie Palliative Care Research Department<br>Davis, Sarah; University College London, Marie Curie Palliative Care Research Department<br>Gola, Anna; Marie Curie Palliative Care Research Unit, UCL Division of Psychiatry<br>Harrington, Jane; University College London, Marie Curie Palliative Care Research Department<br>Kupeli, Nuriye; Marie Curie Palliative Care Research Unit, UCL Division of Psychiatry<br>Vickerstaff, Victoria; University College London, Marie Curie Palliative Care Research Department<br>King, Michael; University College Medical School, Division of Psychiatry<br>Leavey, Gerard; University of Ulster, Psychology<br>Nazareth, Irwin; UCL, Primary Care and Population Health<br>Omar, Rumana; University College London,<br>Jones, Louise; University College London, Marie Curie Palliative Care Research Department<br>Sampson, Elizabeth; University College London, Marie Curie Palliative Care Research Department
**Primary Subject Heading**:	Palliative care
Secondary Subject Heading:	Neurology
Keywords:	Dementia < NEUROLOGY, Adult palliative care < PALLIATIVE CARE, Change management < HEALTH SERVICES ADMINISTRATION & MANAGEMENT, MEDICAL EDUCATION & TRAINING

For peer review only - http://bmjopen.bmj.com/site/about/guidelines.xhtml

IMPLEMENTING THE COMPASSION INTERVENTION, A MODEL FOR INTEGRATED CARE FOR PEOPLE WITH ADVANCED DEMENTIA TOWARDS THE END OF LIFE IN NURSING HOMES: A NATURALISTIC FEASIBILITY STUDY

Kirsten J Moore[1], Bridget Candy[1], Sarah Davis[1], Anna Gola[1], Jane Harrington[1], Nuriye Kupeli[1], Victoria Vickerstaff[1], Michael King[2], Gerard Leavey[3], Irwin Nazareth[4], Rumana Z Omar[5], Louise Jones[1], Elizabeth L Sampson[1]

[1]Marie Curie Palliative Care Research Department, Division of Psychiatry, University College London, London, United Kingdom;

[2]Division of Psychiatry, University College London, London, United Kingdom;

[3]Bamford Centre for Mental Health & Wellbeing, University of Ulster, Derry Londonderry, United Kingdom;

[4]Department of Primary Care and Population Health, University College London, United Kingdom;

[5]Department of Statistical Science, University College London, London, United Kingdom;

Corresponding author: Kirsten Moore kirsten.moore@ucl.ac.uk

6th Floor, Maple House, 149 Tottenham Court Road, London, United Kingdom, W1T 7NF

P: +44 (0)20 7679 9488

Word count (excluding title page, abstract, references, figures and tables): 6769 (6010 excluding quotes)

For peer review only - http://bmjopen.bmj.com/site/about/guidelines.xhtml

ABSTRACT

Background: Many people with dementia die in nursing homes, but quality of care may be sub-optimal. We developed the theory-driven 'Compassion Intervention' to enhance end-of-life care in advanced dementia.

Objectives: To (i) understand how the Intervention operated in nursing homes in different health economies; (ii) collect preliminary outcome data and costs of an Interdisciplinary Care Leader to facilitate the Intervention; (iii) check the Intervention caused no harm.

Design: A naturalistic feasibility study of Intervention implementation for 6 months

Settings: Two nursing homes in northern London, United Kingdom.

Participants: Thirty residents with advanced dementia were assessed of whom nine were recruited for data collection; four of these residents' family members were interviewed. Twenty-eight nursing home and external healthcare professionals participated in interviews at seven (n=19), 11 (n=19) and 15 months (n=10).

Intervention: An Interdisciplinary Care Leader led two core Intervention components: 1) integrated, interdisciplinary assessment and care; 2) education and support for paid and family carers.

Data collected: Process and outcome data were collected. Symptoms were recorded monthly for recruited residents. Semi-structured interviews were conducted at seven, eleven and 15 months with nursing home staff and external healthcare professionals and at seven months with family carers. Interdisciplinary Care Leader hours were costed using Department of Health and Health Education England tariffs.

Results: Contextual differences were identified between sites: Nursing Home 2 had lower involvement with external healthcare services. Core components were implemented at both sites but multidisciplinary meetings were only established in Nursing Home 1. The Intervention prompted improvements in advance care planning, pain management and person-centred care; we observed no harm. Six-month Interdisciplinary Care Leader costs were £18,255.

For peer review only - http://bmjopen.bmj.com/site/about/guidelines.xhtml

For peer review only

Conclusions: Implementation was feasible to differing degrees across sites, dependent on context. Our data inform future testing to identify the Intervention's effectiveness in improving end-of-life care in advanced dementia.

Trial registration: ClinicalTrials.gov:NCT02840318

STRENGTHS AND LIMITATIONS OF THIS STUDY

- This feasibility study informs future testing of the Compassion Intervention to identify its effectiveness in improving end of life care for residents with advanced dementia and their families.
- We followed principles of dynamic sustainability, recognising that implementing protocols in real-life settings requires adaptations, and that rigid adherence to guidelines tested in controlled settings may not be suitable or effective in broader contexts.
- We structured our approach using the five phases of implementation described in the literature on whole systems change in healthcare including orientation, insight, acceptance, change and maintenance.
- Recognising the importance of context on implementation, we report on four levels of nursing home context: political and economic; organisational; social; and individual professionals
- As an exploratory study the sample size was small and we did not aim to detect differences or calculate a sample size for future studies.

INTRODUCTION

Dementia is the fourth commonest cause of death in high income countries[1] where most people with dementia die in long-term care institutions including nursing homes (NHs)[2-4]. The European Association for Palliative Care (EAPC) defines good care for people with dementia approaching death as person-centred, involving shared decision-making with the

For peer review only - http://bmjopen.bmj.com/site/about/guidelines.xhtml

person with dementia and family members[5]. This may require an integrated approach [6] and a central care coordinator[5]. UK policy states that care is integrated when “people benefit from care that is person-centred and co-ordinated within healthcare settings, across mental and physical health and across health and social care. For care to be integrated, organisations and care professionals need to bring together all of the different elements of care that a person needs.”[7]

Currently, barriers to integrated care remain[8]. Many NH residents experience burdensome interventions and distressing symptoms during the last months of life[9]. Recent data show higher emergency admissions amongst older people residing in NHs[10], indicating persistent gaps in healthcare planning.

Providing good EOL dementia care is complex, prognosis is unpredictable[11] and managing symptoms is difficult when communication is compromised. The need for a complex intervention is reflected in the EAPC’s 57 recommendations for optimal EOL dementia care[5]. However, interventional research on providing EOL care in dementia is scant[12] and lacks a theoretical basis[13].

Establishing a complex intervention begins with development based on the available evidence and theories, testing its acceptability and feasibility in practice, evaluation via larger trials through to wider dissemination into practice[14]. Practice change theories highlight the challenge of incorporating interventions into practice and the need to consider the effect of context at societal, organisational and individual levels[15].

Few other interventions have been specifically developed to improve EOL care in advanced dementia. In the US, an interdisciplinary approach towards individualised care plans for residents with advanced dementia achieved this by creating new hospice units within the long term care setting rather than attempting to change NH practice [16]. A protocol for an

For peer review only - http://bmjopen.bmj.com/site/about/guidelines.xhtml

Australian trial describes a study to be conducted that aims to promote family case conferencing through training NH nurses to work as palliative care coordinators and involving family, NH staff and healthcare professionals in case conferences for residents with advanced dementia [17]. In the UK, the Gold Standards Framework for Care Homes (GSFCH) and the ABC EOL Education Programme promote a palliative approach within care homes (including NHs), although not specifically for residents with dementia [18 19]. Further studies of the GSFCH have found that most care homes fail to pass the accreditation standard and that high facilitation with additional action learning sessions increased accreditation rates from 7% to 83% [18]. This suggests that education programmes alone are unlikely to change resistant norms and practices[20].

The Compassion Intervention

Within a three-year research programme funded by Marie Curie Care (National Institute for Health Research, Primary Care Research Network Refs. 12621; 12623)[21], we used the RAND/UCLA Appropriateness Method[22] to achieve national consensus on the components of Compassion ('the Intervention'), a complex model of EOL care for people with advanced dementia. The development of the Intervention has been reported[6], is based in theories of multi-level and whole systems change[15 23], and is described in detail in a manual (available on the Marie Curie website).

The Intervention is aimed at people aged 65 years and over who have advanced dementia using criteria based on an existing model of UK best practice[24]:

a) memory problems indicating a diagnosis of dementia according to the fourth Diagnostic and Statistical Manual of Mental Disorders;
b) Functional Assessment Staging scale grade 6a (difficulty putting on clothing) through to 7f (unable to hold head up)[25];

For peer review only - http://bmjopen.bmj.com/site/about/guidelines.xhtml

c) comorbidities or unmanaged symptoms such as agitation, recurrent infections, pain and pressure ulcers.

There are two core components: facilitation of an integrated, multi-disciplinary approach to assessment, treatment and care; and education, training and support for formal and informal carers (Table 1). The Intervention is coordinated by an Interdisciplinary Care Leader (ICL) who scopes local practice and identifies key personnel to support EOL care. Scoping ensures the Intervention complements, rather than duplicates, existing local processes. The ICL establishes and co-ordinates key activities to address the two core components of the Intervention (Table 1). Activities to facilitate component 1 include: (i) person-centred assessment of residents, focussing on their physical, psychological, emotional and social needs, (ii) meetings of the core care team and the wider multidisciplinary care teams. Activities to facilitate component 2 include: (iii) staff training sessions, education and support for NH staff and family carers. The ICL role requires a broad range of skills including clinical experience in care of frail older people and those with dementia, particularly towards EOL, ability to educate staff and talk empathically with family carers, and sensitivity to identify and minimise poor care practices. Skills may be drawn from the fields of nursing, social work or a profession allied to medicine.

The Intervention has similar components to existing EOL programmes in care homes such as education provision [18 19] and multidisciplinary input [17]. The key distinguishing feature of the Intervention is the role of the independent ICL who works solely with two NHs to provide mentoring, role modelling, advice and training and who can develop relationships with NH staff, external healthcare professionals, residents and family carers and develop an in-depth understanding of the organisational culture underpinning practice and impacting on practice change initiatives.

Table 1: Key activities of the Compassion Intervention

For peer review only - http://bmjopen.bmj.com/site/about/guidelines.xhtml

Component and activity	Purpose	Who is involved	Content
1: facilitation of an integrated, multi-disciplinary approach to assessment, treatment and care:<br>a) Individual holistic resident assessment	To identify symptoms, areas of current unmet need, anticipated future needs and corresponding actions and goals.	The ICL assesses eligible residents in conjunction with NH nurses and healthcare assistants. The process involves liaison with the resident and family about their perceived needs, issues and expectations regarding EOL care. The assessment involves observations and if possible, discussions with the resident. The assessment template focuses on observational measures to identify whether the resident is showing signs of comfort, discomfort, distress and/or pain.	Assessment template:<br>• Dementia diagnosis and progression (Functional Assessment Staging scale)<br>• Significant other medical conditions<br>• Life history, interests<br>• Important goals for care & wellbeing<br>• Needs or restrictions related to faith and/or culture<br>• EOL wishes (Did the resident document preferences when they had capacity? Are family carer preferences documented? Are resuscitation status and preferred place of death documented and reviewed?)<br>• Current medication (and recent changes)<br>• Level of meaningful communication & understanding<br>• Presence of pain or discomfort (Pain Assessment in Advanced Dementia)<br>• Behavioural symptoms and sleep disturbance<br>• Psychological wellbeing, mood, anxiety or depression (Cornell Scale for Depression in Dementia)<br>• Mobility, falls risk, sitting balance and posture, contractures/tone<br>• Skin conditions, pressure sore risk (Waterlow score)<br>• Continence, constipation/bowel problems, UTIs<br>• Eating and swallowing, oral care, weight loss, nutritional status<br>• Other problems – chest infections, breathlessness, fits, blackouts<br>• Recent change in condition<br>• Summary of unmet needs and anticipated/ future needs<br>• Action plan and goals
1: facilitation of an integrated, multi-disciplinary approach to assessment, treatment and care:<br>b) Weekly core meetings	To review, agree on and enact (including referrals), the individual holistic resident assessments.	The core team includes those responsible for medical, nursing and social needs of resident and may include: the clinician responsible for resident's medical needs (GP, geriatrician or Old Age Psychiatrist), NH nursing staff responsible for resident's needs, and the ICL	Review of individual assessments including developing an action plan to address areas of unmet need, discussion of anticipated needs, an escalation plan for the most likely 'what ifs', review of medications and prescribing 'just in case' medications if appropriate and review of EOL wishes and resuscitation status to ensure these are clearly documented. A review date and whether the resident's needs require discussion with the wider team will be decided.
1: facilitation of an integrated,	To discuss (in person or via teleconference),	The wider team will consist of the core team plus any local health and social care	The core team will present for discussion residents who have complex needs requiring specialist advice or

For peer review only - http://bmjopen.bmj.com/site/about/guidelines.xhtml

Component and activity	Purpose	Who is involved	Content
multi-disciplinary approach to assessment, treatment and care: c) Monthly wider team meetings	complex cases and review care plans, consider significant events, critical incident analysis.	professionals and specialist services involved in the care of people with advanced dementia. This is likely to include General Practice, Care of the Elderly, Old Age Psychiatry, Palliative Care, Social Services and Community services such as District Nursing, Speech and Language Therapy, Dietetics, Tissue Viability, Physiotherapy and Occupational Therapy. Composition will depend on local working practices and the availability of key personnel.	those where actions agreed by the core team have not been successful at alleviating symptoms. The wider team will also consider learning or training needs that may become evident as a consequence of this shared working. The meetings will include discussion of critical incidents, deaths, hospital admissions, complaints or compliments, and significant events relating to the care of residents so that learning points can be identified.
2: Education, training and support for formal and informal carers	To establish and address the educational needs of staff members so that they can recognise and respond effectively to the needs of people with advanced dementia and to support family carers with increased confidence	ICL will work with the NH and wider team to identify and address education needs and will obtain agreement from NH manager to run formal training sessions. The ICL will be supported by the wider team to undertake training and education. The target of training could include staff and family carers.	EOL care for people with advanced dementia linking to core competencies outlined in[26] including:<br>• Communication skills with residents with advanced dementia and family carers<br>• Assessment and care planning<br>• Symptom management to maintain comfort and wellbeing<br>• Advance care planning<br>• Knowledge and values, to understand advanced dementia and EOL care and when to refer to specialist services. To be sensitive to the needs of family carers and to foster respect, dignity and quality care.

Aim

We aimed to (i) understand how the Intervention operated in two NHs in different health and social care settings; (ii) collect preliminary outcome data and estimate the cost of employing an ICL to inform further evaluative studies; (iii) check that the Intervention caused no physical or psychological harm to residents or their family carers.

METHOD

A naturalistic feasibility study of the Compassion Intervention. We followed the principles of dynamic sustainability, recognising that implementing protocols in real-life settings requires

For peer review only - http://bmjopen.bmj.com/site/about/guidelines.xhtml

adaptations, and that rigid adherence to guidelines tested in controlled settings may not be suitable or effective in broader contexts[27]. We structured our approach using the five phases of implementation described by Grol[23]:

a) Orientation (awareness of the need for a revised model of care; interest and involvement in the work)
b) Insight (understanding of the revised model of care; insight into existing routines of care)
c) Acceptance (positive attitudes to the possibilities of developing practice; a decision to explore change)
d) Change (actual adoption of a new care model; try-out and confirmation of value)
e) Maintenance (new practice integrated into routines; new practice embedded in the organisation).

Recognising the importance of context on implementation, we report on four levels of NH context: political and economic; organisational; social; and individual professionals[23].

We employed a full-time ICL (KM) with a social care background and experience of working with people with dementia in NHs. The ICL received supervision from clinicians with palliative and dementia expertise. Two NHs were invited to participate; both were involved earlier in our research programme and provided data for a longitudinal (9 months) cohort study to understand the clinical context of people with advanced dementia and their family carers[21]. NH managers identified eligible residents. We aimed to assess two residents in each NH per week (Activity 1a, Table 1).

Implementation occurred over 6 months at each site (see published protocol[28], Supplementary file 1 and Supplementary file 2). In month 1, the ICL met with NH managers and key external healthcare professionals, introduced herself to staff and displayed study posters. The Intervention was launched in Nursing Home 1 (NH1) in May 2014 and Nursing

For peer review only - http://bmjopen.bmj.com/site/about/guidelines.xhtml

Home 2 (NH2) in June 2014. Table 1 shows the activities led by the ICL and after six months the ICL ceased active engagement. To assess maintenance of activities, interviews with relevant stakeholders were conducted after the ICL withdrew at months 7, 11 and 15. Participants were recruited from May 2014 to August 2015. The nature of the intervention prevented masking but independent researchers collected individual level resident and carer data and conducted qualitative interviews.

Data collection

Scoping of existing context

The ICL interviewed each NH manager prior to launching the Intervention. Topics included: resident characteristics, staffing levels, care planning and communication processes, access to external healthcare professionals, training opportunities, dementia and palliative care and expectations about the Intervention. This was supplemented through meetings with deputy managers and other external healthcare professionals.

Qualitative and quantitative process data recorded by ICL

The ICL kept a (i) reflective diary recording observations of practice, liaison with staff, family and residents, examples of improvements in care and personal responses to the role[29]; (ii) a daily log of time spent on tasks related to implementation to enable estimation of costs. We assumed that staff time spent in meetings and training was consistent with usual working practice and so was not considered an additional cost; any opportunity costs incurred would have been offset by the training skills acquired.

Over six months at each site, the ICL collected monthly NH-wide data on the number of residents with: documented resuscitation status; a pain management plan; preferred place of death recorded; hospital admissions as possible indicators of quality of EOL care. Data on emergency phone calls and location of deaths were also collected for this purpose. Resident

For peer review only - http://bmjopen.bmj.com/site/about/guidelines.xhtml

assessments undertaken by the ICL (Activity 1a, Table 1) were part of routine care and were maintained within the NH as clinical information according to their governance polices. Findings from assessments could be reflected on in the anonymised ICL diary and used to inform other Intervention activities such as training. Formal training sessions with staff and family (Activity 2, Table 1) were formally evaluated by participants.

NH resident and carer data

Monthly individual outcome data from participant residents who had been assessed by the ICL and their family carers were collected by researchers (NK, SD). Residents were recruited during the first four months of implementation to enable at least three months of outcome data. We used measures from our earlier cohort study for simple comparisons and to check for potential harm[21]. To describe the sample at baseline we used the Functional Assessment Staging scale[25], the Charlson Comorbidity Index[30] and Bedford Alzheimer Nursing Scale[31]. To assess resident outcomes we used the Waterlow Scale (pressure ulcer risk)[32], Neuropsychiatric Inventory[33], Cohen Mansfield Agitation Inventory[34], Pain Assessment in Advanced Dementia[35], Symptom Management at EOL in Dementia[36] and Quality of Life in Late Stage Dementia Scale[37]. For carer outcomes we used the 22-item Zarit Burden Interview [38], the Hospital Anxiety and Depression Scale[39], Satisfaction with Care at EOL in Dementia[36] and the Resource Utilization in Dementia Questionnaire[40].

Qualitative interview data from staff and family carers

We conducted semi-structured interviews with a purposive representative sample of NH staff and attending professionals at three time-points (months 7, 11 and 15) after the ICL left the site. Family carers who had agreed for a resident to have monthly individual data collected were invited for interview at month 7. Interviews were audio-recorded and transcribed verbatim. We aimed to: assess participants' views of the strengths and weaknesses of the

For peer review only - http://bmjopen.bmj.com/site/about/guidelines.xhtml

Intervention; identify whether any changes in practice were implemented due to the Intervention; and explore whether these changes were maintained after the ICL left.

Analysis

Qualitative analysis

Transcripts were checked against the audio-recording. One researcher involved in interviewing and transcribing (NK) re-read and coded all transcripts using QSR International Pty Ltd NVivo V10 software (2012). Framework analysis was used[41], based on the five phases of implementation[23]. Small chunks of text were extracted and coded, summarising their content. NK categorised each piece of coded text under each of the five phases. After all coded text was categorised, codes were grouped into a smaller number of themes within each phase of implementation. Additional details about each category reported by Grol et al[23] were also used to inform the categorisation process. The revised structure was reviewed by GL to check for agreement with interpretation. This led to an additional theme being incorporated into the context section of the results. Themes were evident in both NHs, unless identified otherwise.

Quantitative analysis

Process data are reported as total number of activities (as outlined in Table 1) undertaken and total ICL hours spent on different activities. ICL hours spent on activities associated with the implementation were costed using the Department of Health and Health Education England tariffs to estimate the cost of engaging the ICL. Training evaluations and outcomes (facility wide and individual) are reported using descriptive statistics using statistical package IBM SPSS Version 22 (2013). Outcome data were used for monitoring potential harm and to examine the feasibility of collecting measures in future trials, hence a sample size calculation was not performed. For individual assessments we present outcome measures from the last available assessment using descriptive statistics. We also compare these measures with

For peer review only - http://bmjopen.bmj.com/site/about/guidelines.xhtml

For peer review only

data from our earlier cohort study but did not make statistical comparisons due to an anticipated small sample size.

Ethics approval and consent to participate

Ethical approval for roll out of Compassion and data collection was granted by the National Research Ethics Service, London—Camden and Islington Research Ethics Committee (Reference 14/LO/0370) and for assessment of maintenance and sustainability by UCL Research Ethics Committee (ID 3618/001). NH managers gave written consent for their site to participate, and permission for the ICL to carry out clinical assessments of eligible residents and have access to their files. None of the residents had capacity to make an informed decision for research participation so NH managers invited their next of kin/primary contact to give agreement. If next of kin were not available, a professional consultee provided agreement according to the Mental Capacity Act (2005). Staff and family gave written informed consent prior to each interview.

RESULTS

We begin by describing the NH context based on the experiences of the ICL, data collected during set-up and qualitative interviews. We describe how the Intervention operated in practice from experiences of the ICL and qualitative interviews. We report the extent to which the core Intervention activities (Table 1) were possible. We present findings from the qualitative interviews to understand the five phases of implementation: orientation, insight, acceptance, change and maintenance [23]. Finally we present individual and NH wide outcomes and cost data to inform future testing or commissioning of a similar intervention. Figure 1 provides a flowchart of all participants. In total 48 interviews were conducted (NH1=30; NH2=18) with 28 NH and external healthcare professionals at seven (n=19), 11 (n=19) and 15 months (n=10). Four family carers all from NH2 were interviewed at seven months.

For peer review only

Figure 1 here

Context

Supplementary file 3 describes both NHs according to contextual levels; political and economic, organisational, social, and individual professionals[23]. While both NHs were located within the same broader political and economic contexts, they also operated within different local funding systems for health and social care services (Clinical Commissioning Groups; CCGs). NH1 was located in a more socio-economically deprived area[42]. Both NHs were located in CCGs with priorities around EOL, but only the NH1 CCG also had a priority relating to care for the ‘frail and elderly’[43 44]. NH1 was located in a CCG with fewer NHs than NH2. Both NHs were part of larger private companies and both had contracts with one GP surgery with the goal of having one GP oversee the medical care of all residents within the NH. Key functional differences between NH1 and NH2 related to access and involvement with external healthcare services, level of detail in care planning processes, and procedures for training for staff, all indicating greater support and development of processes in NH1. While NH1 only contained nursing beds (99 beds with 85 for older people), NH2 had three units with only two of these providing nursing care (52 beds). The third unit (25 beds) was a residential unit with visiting nurses only; residents from here were not assessed during the Intervention.

During implementation and through in-depth qualitative interviews, we found that the context of both NHs was characterised by poor knowledge in dementia and EOL care. Training needs were identified in: pain management, clinical observation and needs assessment, communication with family and residents, advance care planning, person-centred care, psychological aspects of dementia and transition planning. For example, concerns were

For peer review only - http://bmjopen.bmj.com/site/about/guidelines.xhtml

raised by NH nurses and external healthcare professionals about the confidence of NH nurses having EOL conversations with family:

> *"…often these conversations are quite difficult to conduct and it needs time and it needs some background knowledge and I... No disrespect to the nurses here, I just don't think many of them would have the depth of knowledge and skills to actually do that" (NH1 Geriatrician, Month 11)*

Staff worried about the pressures of time and the need to complete tasks which sometimes meant basic care tasks were overlooked, lengthy discussions about EOL care were impossible and social engagement with residents was minimal.

Even the patient care, she [ICL] was able to get in and say this one their nails need to be cut, this one has been refusing to get out of bed but their hair needs to be washed, maybe we have applied some approaches but they did not work… [ICL] had all the time, she was able to … give recommendations so actually GP will do this and us [nurses], we'll do this. (NH1 Deputy Manager, Month 7)

Activities undertaken

Assessments (Activity 1a), core meetings (Activity 1b) and training (Activity 2) were undertaken in both NHs (Table 2). Weekly core meetings were scheduled, but many were cancelled due to staff leave or immediate resident care needs. At NH2, the GP experienced significant time constraints and attended only the first two meetings. The group agreed to weekly meetings with the ICL, manager and nurse with specific medical issues referred to the GP. Core meetings provided an opportunity to discuss individual assessments. These involved the ICL reviewing the resident's file, observing and talking to them and their family and seeking clarification from NH staff. NH staff had limited time and may have viewed this as duplicating existing assessments. Discussions with families sought views about current

For peer review only - http://bmjopen.bmj.com/site/about/guidelines.xhtml

care and concerns about EOL care. The ICL intended to involve NH staff in these discussions but competing staff demands usually prevented this. Common issues identified included swallowing and eating difficulties, pain, pressure area care and lack of social engagement. Advance care plan documentation was more routinely discussed in core meetings at NH1 than NH2.

Table 2: Process Measures

Component	Over 6 month period	NH1	NH2
Scoping	ICL visits to NH prior to implementation	8	2
Scoping	ICL visits to external HCPs prior to implementation	2 - palliative care nurse and GP	0
All components	ICL visits to NH during implementation	64	53
All components	ICL visits to external HCPs during implementation	1 – palliative care nurse	1 - palliative care Lead Clinical Nurse Specialist
1a) Individual holistic resident assessments	Individual assessments completed	15	15
1a) Individual holistic resident assessments	Number of discussions with family members (not number of family members)	15	24
1b) Weekly core meetings	Number of meetings	10 core meetings with GP, deputy manager and nurse from relevant floor (GP missed one meeting)	8 core meetings with manager and a nurse. GP attended first two meetings.
1b) Weekly core meetings	Individualised assessments discussed at core meeting	15	13
1b) Weekly core meetings	Individual reviews completed	15	0*
1b) Weekly core meetings	Referrals made to external HCPs	6 (2 X Community Mental Health Team; 2 X Speech and Language Therapist; 2 X Occupational Therapist)	4 (3 X Old Age Psychiatrist; 1 X Manual Handling Trainer)

For peer review only - http://bmjopen.bmj.com/site/about/guidelines.xhtml

Component	Over 6 month period	NH1	NH2
1c) Monthly wider team meetings	Number of meetings	6 meetings; usually with Geriatrician, GP, palliative care nurse, Triage and Rapidly Elderly Assessment Team, NH nursing staff and deputy manager (and/or manager)	Wider meetings not established. The ICL was able to arrange one meeting with the palliative care nurse, NH manager and deputy manager.
1c) Monthly wider team meetings	Number of residents assessed by ICL discussed	11	Not applicable
2) Education	Number of training sessions (total number of attendees)	9 (84)	5 (21)

*No formal reviews involving reassessment were completed at NH2, although there was subsequent discussion of many of the residents at subsequent meetings.

During core meetings (Activity 1b), staff training needs were discussed and sessions planned (Activity 2), including managing distress during hoist transfers (NH1), and understanding pain and behavioural symptoms (both NHs). At NH1 the manager requested a general information session on dementia and EOL care, while at NH2 the manager requested a half-day session for nurses on pain management and discussing EOL care with family. Fewer training sessions were held at NH2 and staff attendance was sub-optimal. Training was positively evaluated (Table 3).

Table 3: Staff training evaluation

	Reducing distress during personal care	Behaviour and pain management		EOL care in dementia	
NH	NH1 (n= 23)	NH1 (n=36)	NH2 (n=12)	NH1 (n=25)	NH2 (n=9*)
Duration in hours	1	1	1	1	4
Sessions	2Xday; 1Xnight	2Xday; 1Xnight	2Xday; 1X night & day	2Xday; 1Xnight	2 X nursing staff
**Evaluation: Median (IQR)**					
Was this training relevant to your	4 (3-4)	4 (4-4)	4 (3-4)	4 (3-4)	4 (3.25-4)

For peer review only - http://bmjopen.bmj.com/site/about/guidelines.xhtml

day to day work? #					
Did you learn anything new from the training? #	3 (3-4)	4 (3.25-4)	4 (3-4)	3 (3-4)	3.5 (3-4)
Do you think this training will influence your work? #	4 (3-4)	4 (4-4)	4 (3-4)	3 (3-4)	4 (3-4)
Was the training level:~	1 (0-1)	1 (1-1)	1 (1-1)	1 (1-1)	1 (1-1)
Did the training provide a useful refresher? #	3 (3-3)	4 (3-4)	3 (3-3.75)	Not asked	Not asked
Has this training improved your confidence in talking to family about EOL care? ^	Not asked	Not asked	Not asked	4 (4-4)	4 (4-4)

*evaluation sheet missing from one attendee
measured on a 5 point likert scale from 0=Strongly Disagree – 4=Strongly Agree
~ measured on a 3 point likert scale: 0=too basic; 1=about right; 2= too complex
^ measured on a 5 point likert scale from 0=Not at all – 4=Yes, a lot; higher median better

Both managers requested the ICL to run information sessions for family members on issues regarding dementia, EOL symptoms and advance care planning. Twelve family members attended at NH1 with the NH manager. At NH2 the session (6 families) generated much discussion, overran the allotted time and led to a follow-up session (3 families). Evaluations indicated that the sessions were relevant, helpful, contained new information and that the timing was appropriate.

The lower involvement with external healthcare professionals at NH2 prevented establishing wider meetings (Activity 1c). At NH1, six months prior to implementation, wider monthly meetings had been initiated. These meetings were supported by the ICL and involved both review of residents requiring palliative care and reflecting on whether EOL care processes could have been better for deceased residents.

For peer review only - http://bmjopen.bmj.com/site/about/guidelines.xhtml

Implementation phases

The staff and family interviews give information on the five implementation phases [23].

Phase 1: Orientation

NH managers highlighted their role in promoting the Intervention; "*Within two or three weeks I had gone in and prepared the staff that she [ICL] was going to be here and that she had full access to the records and the staff*" (NH1 Manager, Month 7). Staff and family engagement was attributed to the importance of the Intervention topic. "*I am happy that something like this is going on, that someone is interested and is trying to help people with dementia and end of life*" (NH1 Nurse, Month 7); and "*I think it was right for the programme to suggest and talk about end of life palliative care*" (NH2 Family Carer, Month 7). Characteristics of the ICL were attributed to engaging staff with the Intervention; *"[ICL] was very helpful… I would say she's a very good listener… she's got plenty of time, which I think is lovely"* (NH2 Deputy Manager, Month 7).

Phase 2: Insight

As reported under context, NH staff had only basic knowledge regarding dementia EOL care and it was important that they gained insight into the need for practice improvements. Many staff were receptive to receiving information. Training from the ICL improved knowledge and promoted a person-centred view of dementia care. The Intervention provided insights into existing routines critical for driving practice improvements, often highlighting existing deficits in the care being provided:

> *"… through these 6 months I realised... the paperwork was being reviewed, reviewed, reviewed but actually the patient was not being reviewed it was just being carried forward." (NH1 GP, Month 7)*

For peer review only - http://bmjopen.bmj.com/site/about/guidelines.xhtml

"She needs to give us more training about the caring, like dementia. It will also help us communicate with our colleagues because some of our colleagues don't know how to communicate with the service user; she can train them how to do it." (NH1 Healthcare Assistant, Month 11)

I think we will take on her advice that she gave on end of life and on dealing with dementia for the relatives. We deal with the residents but then it's the relatives that… need the help. Why's this happening? Why doesn't he know them? We do a lot with the residents but not with the relatives. (NH2 Activity Coordinator, Month 7)

Whilst wider meetings at NH1 had started before implementation, the ICL also provided an alternative view during these meetings:

"…her [ICL] input was useful… during the MDM [wider multidisciplinary meeting]…her feedback and some of her suggestions actually helped us to see things a little bit differently" (NH1 Geriatrician, Month 7)

Phase 3: Acceptance

Staff were energised by the Intervention as it provided an opportunity to develop new ideas and skills, and, ultimately, improve dementia care:

"… anybody new coming [in] will come up with new ideas, new experiences from other places, it's building up. You cannot say I am that clever when I am not. I am open to new ideas all the time." (NH1 Nurse, Month 7)

"I never knew what it was she {ICL] was willing or she was about to tell me. But because it was end of life management I hope it is good for every carer to know how to

For peer review only - http://bmjopen.bmj.com/site/about/guidelines.xhtml

manage… it will help me to get some ideas to prepare and how to deal with those situations". (NH1 Healthcare Assistant, Month 7)

However, initially, the NH staff were wary of change and the ICL experienced some early difficulties engaging:

"I don't know that the staff really understood for quite a while why she [ICL] was there and what she was doing. I don't think it was her problem; I think it was more what the project was all about." (NH1 Palliative Care Nurse, Month 7).

Phase 4: Change

Participants identified practices that had become part of NH protocols and routines as a result of the Intervention. Participants confirmed the value of the ICL's EOL discussions with family carers. At NH1 a modified template to support advance care planning was introduced to replace three existing care plans relating to EOL wishes, and to provide greater guidance to NH staff about how to manage possible EOL symptoms. At NH2 modifiable wall-mounted care charts (Care Charts UK ©) in residents' rooms were introduced to communicate residents' needs and preferences. Greater focus on pain assessment for residents who were unable to verbally communicate led to introducing the Pain Assessment in Advanced Dementia assessment[35] and pain management plans at NH2.

"[ICL] gave me this wonderful sheet about pain control, really and how to… so we've implemented some of the things that she has given to us." (NH2 Deputy Manager, Month 7)

However, time demands also prevented NH staff and GPs attending Intervention meetings and training:

For peer review only - http://bmjopen.bmj.com/site/about/guidelines.xhtml

"It was really good what she was saying but I haven't got the time to do it. So she would sit and discuss them and it would take them half an hour forty minutes to talk about two or three patients and if I've got to see fourteen in the morning - I just can't do it." (NH2 GP, Month 7)

"I didn't do the end of life training; not that I didn't want to do it, there was not really the chance to go in there." (NH1 Healthcare Assistant, Month 7).

Phase 5: Maintenance

Staff described the new Advance Care Plan at NH1 and pain management plans and the wall mounted care charts at NH2 as being maintained at Months 11 and 15 and becoming embedded into routine care:

"The care [nursing] home are actually using her template, developed a new advanced care plan which has incorporated the points that she [the ICL] raised and so that's what we are using now, for all new patients that come in… existing patients, we are transferring gradually. (NH1 GP, Month 11)

Do you know who loves them [care charts] best? Can I tell you, the relatives… they will tell you the detail about their loved one… So the minute somebody comes in I tell them about the work that the ICL did and then I tell them about the 'this is me' life profile… when we had our Care Quality Commission inspection they really liked the 'this is me' profiles (NH2 Manager, Month 15)

It was apparent that the need for staff development and a shift from task-driven to compassionate care would require a longer duration and further training and support from

For peer review only - http://bmjopen.bmj.com/site/about/guidelines.xhtml

the ICL. Continuing support and training from the ICL could build on this work, further enhancing staff confidence.

> *"I think that if she'd been there for a whole lot longer then what would have happened is there would be an evolving of her role in a sense that the issues that were raised would have become identified by the nurses as routine" (NH1 GP, Month 7)*

Cost of Implementation

Supplementary file 4 presents the time the ICL spent on various activities and this was used to calculate the costs of Implementation. Of the total 656 hours, 42% were spent on NH1 activities, 34% on NH2 activities and 24% on activities not attributable to one particular NH. Engagement of the ICL to implement the Intervention in two NHs for six months was costed at £18,255 including on-costs and travel fares (and excluding time the ICL spent on non-Intervention activities).

Individual resident and carer outcomes

We recruited 9/28 residents assessed by the ICL for monthly data collection (Figure 1). Recruitment was hampered by difficulties engaging with family members who had limited day-to-day involvement with their relative and did not respond to letters and phone calls. Four residents died or moved NH before agreement was obtained. One daughter declined participation due to her family's request that their relative should not be involved in research.

At NH1 the three residents had a median age of 81 years (Interquartile Range [IQR]: 76-93) and two were female. At NH2 the median age of the six residents was 80 years (IQR: 76-85) and all were female. Data were descriptively compared to those from the larger cohort (Table 4). As none of the nine participants died during the data collection period, we compared their outcomes with the 52 participants involved in the cohort study who survived

For peer review only - http://bmjopen.bmj.com/site/about/guidelines.xhtml

the nine month data collection period. Findings in Table 4 suggest that the Intervention did not cause harm to residents, but the effects on carers at NH2 may need further consideration.

Table 4: Resident and carer evaluation data compared with larger cohort

Baseline Assessment	***Cohort study (n=52)****	***NH1 (n=3)***	***NH2 (n=6)***
**Functional Assessment Staging scale**			
6b-6d (Unable to bathe independently – urinary incontinence)	0	0	1
6e-7b (doubly incontinent- loss of ability to speak > 6 words)	21	1	4
7c-7e (ambulatory ability lost-can't hold up head independently)	31	2	1
**Charlson comorbidity index** median (IQR)	6 (6-7)	6 (4-7)	5 (4-6)
**Bedford Alzheimer Nursing Scale** median (IQR)	22 (18-23)	22 (21-24)	22 (20-23)
Final Visit	***Cohort study (n=52)***	***NH1 (n=3)***	***NH2 (n=6)***
**Waterlow Scale (Pressure ulcer risk)**			
High risk (15-19)	14 (27)	1 (33)	1 (17)
Very high risk (≥20)	36 (69)	2 (67)	4 (67)
**Neuropsychiatric inventory -** Number of symptoms, median (IQR)	4 (1.5-6)	2 (2-5)	4 (2-6)
**Cohen Mansfield Agitation Inventory:** behavioural disturbances (≥39)	29 (56)	1 (33)	3 (50)
**Pain Assessment in Advanced Dementia:** (n, %)			
Rest (≥2)	10 (19)	0 (0)	2 (33)
Movement (≥2)	29 (60)	2 (67)	1 (17)
**Symptom Management at EOL in Dementia Scale** median (IQR)	26 (20-35)	30 (26-32)	33 (31-37)
**Quality of Life in Late Stage Dementia Scale** median (IQR)	24.5 (20-28.5)	23 (23-31)	25 (20-28)
Carer measures:	(n= 23)	(n=0)	(n= 4)
**Zarit Burden Interview** median (IQR)	11 (6-18)		23 (15-28)
**Hospital Anxiety and Depression Scale ≥8** n (%)			

For peer review only - http://bmjopen.bmj.com/site/about/guidelines.xhtml

Anxiety	8 (35)		2 (50)
Depression	5 (21)		2 (50)
**Satisfaction with Care at EOL in Dementia Scale** median (IQR)	30 (29-33)		34 (28-39)
**Resource Utilization in Dementia Questionnaire** median (IQR)			
Visits from doctor, physiotherapist, psychologist, other HCP in previous month	1 (1-3)	0 (0-2)	1 (1-2)
All general hospital admissions in previous month	0.5 (0-1)	0 (0-0)	0 (0-0)

*The cohort study involved 85 residents in total but this table only includes the 52 participants who survived the nine month data collection period.
Charlson Comorbidity Index (19 diseases)[30]
Bedford Alzheimer Nursing Scale: range 7-28, higher scores indicate severity[31]
Waterlow Scale: range 2-46, higher score higher pressure ulcer risk[32]
Neuropsychiatric inventory: total symptoms, maximum 12[33]
Cohen Mansfield Agitation Inventory: range 29-203, scores ≥39 indicates clinically significant agitation[34]
Pain Assessment in Advanced Dementia: range 0-10; scores ≥2 indicates pain[35]
Symptom Management at EOL in Dementia: range 0–45; higher scores indicate better symptom control[36]
Quality of Life in Late Stage Dementia Scale: range 11-55, lower scores indicate better quality of life[37]
Zarit Burden Interview: range 0-88, higher scores indicate greater burden[38]
Hospital Anxiety and Depression Scale: Anxiety and depression subscales range 0-21, scores ≥8 indicates clinically significant depression or anxiety[39]
Satisfaction with Care at EOL in Dementia: range 10–40; higher scores indicate more satisfaction with EOL care[36]
Resource Utilization in Dementia Questionnaire[40]

For peer review only

NH wide outcomes

NHs did not maintain electronic records of any of the NH-wide outcomes. Manual searches of daily logs and individual care plans were required. At NH1 resuscitation status was not documented consistently and at NH2 obtaining these data required reading of individual care plans. Due to these difficulties we reduced collection frequency to three time points (months 1, 4 and 7). What data were collected showed few of out-of-hours GP calls and visits, ambulance calls and unplanned hospitalisations. At NH1 pain management plan frequency increased slightly during implementation from 71% to 85% of residents. Preferred place of death was reported for 30% of residents at month 1 and 85% at month 4 (month 7 data were unavailable). These measures could only be collected at month 1 in NH2 where we found

For peer review only - http://bmjopen.bmj.com/site/about/guidelines.xhtml

one resident (not cognitively impaired) had a pain management plan in place, 21% had their preferred place of death recorded and 30% had a documented 'Do not attempt resuscitation' form.

Over the seven month data collection period, 17 NH1 residents died, ten in their usual NH. For the seven hospital deaths, one was the preferred place of death reported by family and another did not have a documented preference. For two residents with the NH documented as the preferred place, families requested their relative be admitted to hospital. At NH2 for the three months in which resident deaths were reported, twelve residents died and seven who had a documented preference, died in their preferred place.

DISCUSSION

Principal findings

We report on how the Compassion Intervention operated in two UK NHs in different healthcare funding systems and the feasibility of implementation. Our data inform evaluative studies to address gaps in EOL care for residents with advanced dementia. We found that implementation was dependent on several aspects of the local NH context. These included the state of readiness for accepting the intervention, in particular local funding priorities within the healthcare system and relations between multidisciplinary care providers across specialist and generalist services; organisational structures within the NH including staffing levels, confidence, knowledge and skills of staff, and existing assessment procedures for residents. The period of implementation was short but there was evidence that the Intervention achieved acceptance within both NHs. We noted changes in care processes such as advance care planning, pain management and the introduction of wall-mounted care charts; these were maintained nine months later. Despite limited NH staff availability, three of the four key activities were implemented in both NHs. No wider meetings and fewer

For peer review only - http://bmjopen.bmj.com/site/about/guidelines.xhtml

training sessions were implemented at NH2 than NH1. The NH context may explain these differences.

We were unable to assess whether changes led to better outcomes for residents or family, but there were no indications of harm to residents. Of concern was that the small number of carers recruited appeared to have poorer mental health when compared with the wider cohort, despite reporting benefits of participation and higher satisfaction with end of life care. Possibly distressed carers seeking support were more motivated to participate. Previous studies suggest that EOL discussions can improve carer satisfaction with EOL care [45]. We have analysed practice relating to EOL conversations elsewhere [29].

Strengths and weaknesses

This was an exploratory study. Whilst the sample size was small, we did not aim to detect differences or calculate a sample size for future studies. Our work is strengthened by the theory and evidence underpinning the Intervention described in earlier publications[6 21 28]. We took note of contextual factors affecting the five phases of implementation described in the literature on whole systems change in healthcare[23]. Our Intervention provides a framework that may promote EOL care in accordance with EAPC recommendations[5]. The Compassion Intervention supports many of the EAPC's domains including: 2) person-centred care, communication and shared decision making; 3) setting care goals and advance planning; 6) avoiding overly aggressive, burdensome or futile treatment; 7) optimal treatment of symptoms and providing comfort; 8) psychosocial and spiritual support; 9) family care and involvement; and 10) education of the health care team.

Our implementation phase was short. There was limited time for the ICL to gain the trust of key stakeholders and family members. The short time frame and the difficulty in scheduling weekly meetings to discuss assessments limited the number of residents who could be

For peer review only - http://bmjopen.bmj.com/site/about/guidelines.xhtml

assessed and who were therefore eligible for recruitment for collecting individual outcome data. Often the person listed as a proxy decision maker had minimal contact with the resident and felt unable to make decisions on their behalf, prohibiting recruitment of both carers and residents. Using professional consultees enabled involvement of isolated residents.

Recruitment of only four informal carers limits our understanding of the impact of the Intervention on families and this needs exploration in future work. There is evidence from other research[45] that carers do benefit from attempts to improve care for relatives with dementia who are dying.

Involvement of the ICL in both roll-out and monitoring of the Intervention (KM) creates potential for bias. This may be counter-balanced by the depth of understanding achieved which was of importance at this stage of evaluation. We engaged independent researchers in the analysis of interviews (NK, GL) and quantitative data (AG, VV, RO, ES) and all co-authors critically reviewed the findings. We have not incorporated an analysis of the ICL diary here, but auto-ethnographic findings have been published elsewhere [29].

Implications and future research

Consistent with previous studies[46], collecting NH level data proved challenging and further evaluations should allocate resources for collecting reliable data. The low frequency of deaths, unplanned hospitalisations and out-of-hours calls implies a large number of NHs would be required to give sufficient power to investigate NH wide outcomes. These measures are not very sophisticated indicators of quality of end-of-life care and individual resident measures may be more useful as they describe symptom burden. The Symptom Management at EOL in Dementia[36] and the Satisfaction with Care at EOL in Dementia[36] Scales can assess multiple EOL symptoms and family satisfaction with care.

For peer review only - http://bmjopen.bmj.com/site/about/guidelines.xhtml

For peer review only

The criteria for inclusion may appear inappropriate given that none of the recruited residents died during the intervention period. However, three had died in the period between the ICL assessment and the research team trying to recruit the participant. In addition, another participant died a few weeks after the Intervention period ceased. The other deaths in the NHs were amongst residents who did not all have dementia. Also, there were residents who were eligible for the Intervention but who the ICL had not had time to assess during the Intervention period. Also, our larger cohort study [21], using similar eligibility criteria found that only 36% of residents with advanced dementia died during a nine month observation period, reflecting the difficulty in prognosing EOL in dementia. We advocate a proactive approach to addressing advance care planning and actively managing symptoms of pain and discomfort for all NH residents, with the need for particular attention to the unique needs of residents with advanced dementia and limited capacity to verbally communicate their needs.

We have information regarding the costs, time and skills required to engage an ICL. We also highlight the benefits of an ICL who was external to the NH to drive practice change and to provide independent support for family carers [47]. For localities with good external multidisciplinary support for NHs, the Intervention might be implemented by employing a full time ICL working across 2-3 NHs. However, for contexts such as NH2, external support from a range of disciplinary areas (not costed in this study) would require greater investment from commissioners. The extent to which the context of NH1 or NH2 reflects the typical level of support for UK NHs is unknown.

Further investigation of the Intervention could examine how the ICL role might be integrated into usual practice, perhaps up-skilling an existing NH staff member, harnessing the expertise of a member of the wider multidisciplinary team or through palliative care services provided within the charitable sector such as outreach from a hospice. The benefits of

For peer review only - http://bmjopen.bmj.com/site/about/guidelines.xhtml

external facilitation from programmes such as the Gold Standards Framework have been demonstrated for supporting end of life care in NHs [48]. The ICL may be challenged by working across a large number of NHs and flexibility is needed to allow enough time within each NH for the ICL to integrate and be effective. Further work is required to determine whether the ICL role would need to remain at the same level of intensity and for how long. There is the need for someone with the skills to discuss end of life with family carers and to provide staff training, given the high turnover of direct care staff in NHs [49]. During family group sessions it was evident that carers had a poor understanding of dementia and wanted to learn about all aspects of dementia, not only about EOL. Staff in the facility lacked confidence in providing information to families and would require a considerable amount of development in EOL dementia care before a role of an ICL became redundant.

Our ICL had a social care background but individuals with a different disciplinary background, such as a palliative care nurse or dementia-specific Admiral Nurse, may have brought different skills to the role and focused on different goals and care issues. A key benefit of Compassion appeared to be the ICL offering a more holistic approach which went beyond physical and medical care needs. Professional development and clinical support for the ICL role was crucial.

Further work also needs to examine the applicability of the model to long term care settings where nursing care is not available. We focused on nursing homes in this study as residents fitting the criteria for advanced dementia would most likely require nursing home level of care. In this study we did not involve healthcare assistants in core or wider meetings although their input was sought during assessments and they received training to improve EOL knowledge [50]. The benefit of involving them in the core and wider meetings requires further investigation.

For peer review only - http://bmjopen.bmj.com/site/about/guidelines.xhtml

Our work did not lead to substantial changes to the Compassion Intervention manual. The assessment template we developed aimed to be holistic covering a broad range of issues including the person's physical, social, psychological and spiritual needs. Although observational assessments may have identified environmental factors that impacted on the resident's wellbeing, these were not explicitly included in the assessment but could be important to include [51]. Further testing of the Intervention may lead to further refinement of the assessment and identify new elements over time. In addition, the assessment required some duplication of existing assessments undertaken in each NH. To address this issue we have added a checklist to prompt NHs to examine existing assessment domains rather than requiring another assessment template. Prior to working with this Intervention, NHs should consider the feasibility of weekly core meetings and how to incorporate assessments into existing processes.

The Compassion Intervention was underpinned by organisational change theory [23]. There has been few intervention studies developed in NHs in advanced dementia, but none that have used an external role such as an ICL to facilitate practice change. External facilitators of the education focused GSFCH, report concerns about their lack of time to enable adequate support [52]. The level of facilitation in the Compassion Intervention was higher than the 'high facilitation' reported in the GSFCH programme, and training on its own is unlikely to change resistant norms and practices [20]. The study using the most similar approach but has not been completed at the date of this paper may provide useful insights into the benefits of family case conferencing in the NH setting [17] with implementation of a similar role as ICL but from a nurse within the NH. This will provide a useful comparison for the importance of an internal or external ICL.

Our implementation was flexible in responding to the unique needs of the different NH contexts and the holistic assessments undertaken by the ICL were crucial in providing insights to NH staff about gaps in existing care provision. The ICL implemented a

For peer review only - http://bmjopen.bmj.com/site/about/guidelines.xhtml

relationship-centred approach which aimed to provide information and practical and emotional support to NH staff, family and residents [53]. However, other approaches to implementing practice change are also worth considering. For example, action research used in the NH setting has been useful in transforming task-driven approaches to approaches that engage staff more meaningfully with care processes to enable practice improvements [54].

Conclusion

Implementation of the Compassion Intervention was feasible to differing degrees across two sites, dependent on context. The role of the ICL appeared the key factor for supporting practice change in this exploratory study. Our data inform future testing to identify the Intervention's effectiveness in improving end-of-life care in advanced dementia.

Figure 1: Flowchart of participants

Acknowledgements

We thank other members of the Compassion research team, past and present, for their support in completing this research, particularly Sharon Scott and Steve Morris for their earlier contributions in developing the Compassion programme, and Margaret Elliott for her work on developing the Compassion Intervention manual, providing clinical support to the ICL, and undertaking qualitative interviews. We would also like to thank Ritchard Ledgerd and Dr Karen Harrison-Dening for providing clinical support to the ICL outside the research team. Thank you to Professor Martin Marshall for advice on implementation theory. Thank you to Marie Curie for funding and supporting the programme, the two care homes who completed the Intervention and the residents and carers who participated in this study.

For peer review only - http://bmjopen.bmj.com/site/about/guidelines.xhtml

For peer review only

Competing interests

All authors have completed the ICMJE uniform disclosure form at www.icmje.org/coi_disclosure.pdf and declare: authors had financial support from Marie Curie for the submitted work; no financial relationships with any organisations that might have an interest in the submitted work in the previous three years; no other relationships or activities that could appear to have influenced the submitted work.

Funding

This work was supported by Marie Curie Cancer Care (now Marie Curie), [grant number: MCCC-FPR-11-U] through a process administered in partnership with Cancer Research UK.

Authors' contributions

The Intervention conception and the process used to develop core components of the Compassion manual design was undertaken by LJ, ES, MK, GL, IN, RO, BC and JH. ES and LJ managed the study from conception to completion. VV and AG managed all quantitative data analysis and cleaning with input from ES and RO. SD, JH and NK were involved in undertaking qualitative interviews, transcribing and analysing the qualitative data. KM undertook the role as the ICL working in the two care homes and involved in collecting facility wide data, process data and maintaining a reflective dairy. KM prepared the manuscript which was critically reviewed and the final version approved by all authors.

Data sharing statement

The Final Compassion Intervention Manual will be published with free access on the Marie Curie website (www.mariecurie.org.uk). All process data is included in the paper. We do not have ethical permission to disseminate at an individual level the resident outcome data and staff and family carer transcripts.

For peer review only

REFERENCES

1. World Health Organization. The top 10 causes of death. Fact sheet N°310, 2014.
2. Houttekier D, Cohen J, Bilsen J, et al. Place of Death of Older Persons with Dementia. A Study in Five European Countries. *Journal of the American Geriatrics Society* 2010;58(4):751-56 doi: 10.1111/j.1532-5415.2010.02771.x.
3. Reyniers T, Deliens L, Pasman HR, et al. International Variation in Place of Death of Older People Who Died From Dementia in 14 European and non-European Countries. *J Am Med Dir Assoc* 2015;16(2):165-71 doi: 10.1016/j.jamda.2014.11.003.
4. Sleeman KE, Ho YK, Verne J, et al. Reversal of English trend towards hospital death in dementia: a population-based study of place of death and associated individual and regional factors, 2001-2010. *BMC neurology* 2014;14:59 doi: 10.1186/1471-2377-14-59.
5. van der Steen JT, Radbruch L, Hertogh CM, et al. White paper defining optimal palliative care in older people with dementia: a Delphi study and recommendations from the European Association for Palliative Care. *Palliative medicine* 2014;28(3):197-209 doi: 10.1177/0269216313493685.
6. Jones L, Candy B, Davis S, et al. Development of a model for integrated care at the end of life in advanced dementia: A whole systems UK-wide approach. *Palliative medicine* 2016;30(3):279-95 doi: 10.1177/0269216315605447.
7. Monitor. Guidance: Delivering better integrated care. Secondary Guidance: Delivering better integrated care 2014 (updated 2015). https://www.gov.uk/guidance/enabling-integrated-care-in-the-nhs.
8. Kupeli N, Leavey G, Harrington J, et al. What are the barriers to care integration for those at the advanced stages of dementia living in care homes in the UK? Health care professional perspective. *Dementia (London, England)* 2016 doi: 10.1177/1471301216636302.

For peer review only - http://bmjopen.bmj.com/site/about/guidelines.xhtml

9. Mitchell SL, Teno JM, Kiely DK, et al. The Clinical Course of Advanced Dementia. *New England Journal of Medicine* 2009;361(16):1529-38 doi: 10.1056/NEJMoa0902234.

10. Smith P, Sherlaw-Johnson C, Ariti C, et al. Focus on: Hospital Admissions from Care Homes: Nuffield Trust and Health Foundation, 2015.

11. Mitchell SL, Miller SC, Teno JM, et al. Prediction of 6-month survival of nursing home residents with advanced dementia using ADEPT vs hospice eligibility guidelines. *Jama* 2010;304(17):1929-35 doi: 10.1001/jama.2010.1572.

12. Goodman C, Evans C, Wilcock J, et al. End of life care for community dwelling older people with dementia: an integrated review. *International Journal of Geriatric Psychiatry* 2010;25(4):329-37 doi: 10.1002/gps.2343.

13. van der Steen JT, Goodman C. What research we no longer need in neurodegenerative disease at the end of life: the case of research in dementia. *Palliative medicine* 2015;29(3):189-92 doi: 10.1177/0269216315569998.

14. Craig P, Dieppe P, Macintyre S, et al. Developing and evaluating complex interventions: New guidance. Secondary Developing and evaluating complex interventions: New guidance 2008. https://www.mrc.ac.uk/documents/pdf/complex-interventions-guidance/.

15. Ferlie EB, Shortell SM. Improving the quality of health care in the United Kingdom and the United States: a framework for change. *The Milbank quarterly* 2001;79(2):281-315 doi: 10.1111/1468-0009.00206.

16. Kovach C, Kovach SA, Wilson PE. The effects of hospice interventions on behaviors, discomfort, and physical complications of end stage dementia nursing home residents. *American journal of Alzheimer's disease and other dementias* 1996;11(4):7-15 doi: 10.1177/153331759601100402.

17. Agar M, Beattie E, Luckett T, et al. Pragmatic cluster randomised controlled trial of facilitated family case conferencing compared with usual care for improving end of life care and outcomes in nursing home residents with advanced dementia and their

For peer review only - http://bmjopen.bmj.com/site/about/guidelines.xhtml

families: the IDEAL study protocol. *BMC palliative care* 2015;14:63 doi: 10.1186/s12904-015-0061-8.

18. Kinley J, Stone L, Dewey M, et al. The effect of using high facilitation when implementing the Gold Standards Framework in Care Homes programme: a cluster randomised controlled trial. *Palliative medicine* 2014;28(9):1099-109 doi: 10.1177/0269216314539785.
19. Mayrhofer A, Goodman C, Smeeton N, et al. The feasibility of a train-the-trainer approach to end of life care training in care homes: an evaluation. *BMC palliative care* 2016;15(1):11 doi: 10.1186/s12904-016-0081-z.
20. Johnson M, Attree M, Jones I, et al. Diagnosis, prognosis and awareness of dying in nursing homes: towards the Gold Standard? *International journal of older people nursing* 2014;9(2):95-105 doi: 10.1111/opn.12024.
21. Jones L, Harrington J, Scott S, et al. CoMPASs: IOn programme (Care Of Memory Problems in Advanced Stages of dementia: Improving Our Knowledge): protocol for a mixed methods study. *BMJ open* 2012;2(6) doi: 0.1136/bmjopen-2012-002265.
22. Fitch K, Bernstein S, Aguilar M, et al. The RAND/UCLA Appropriateness Method User's Manual Santa Monica: RAND, 2001.
23. Grol RP, Bosch MC, Hulscher ME, et al. Planning and studying improvement in patient care: the use of theoretical perspectives. *The Milbank quarterly* 2007;85(1):93-138 doi: 10.1111/j.1468-0009.2007.00478.x.
24. Sonola L, Thiel V, Goodwin N, et al. Oxleas advanced dementia service. Supporting carers and building resilience: The Kings Fund, 2013.
25. Reisberg B. Functional assessment staging (FAST). *Psychopharmacology bulletin* 1988;24(4):653-9
26. Skills for Care, Skills for Health, National End of Life Care Programme. Developing end of life care practice: a guide to workforce development to support social care and health workers to apply the common core principles and competencies for end of life

For peer review only - http://bmjopen.bmj.com/site/about/guidelines.xhtml

care. 2012. http://www.skillsforcare.org.uk/Documents/Topics/End-of-life-care/A-guide-to-workforce-development-for-end-of-life-care.pdf.

27. Chambers DA, Glasgow RE, Stange KC. The dynamic sustainability framework: addressing the paradox of sustainment amid ongoing change. *Implementation science : IS* 2013;8:117 doi: 10.1186/1748-5908-8-117.

28. Elliott M, Harrington J, Moore K, et al. A protocol for an exploratory phase I mixed-methods study of enhanced integrated care for care home residents with advanced dementia: the Compassion Intervention. *BMJ open* 2014;4(6):e005661 doi: 10.1136/bmjopen-2014-005661.

29. Saini G, Sampson EL, Davis S, et al. An ethnographic study of strategies to support discussions with family members on end-of-life care for people with advanced dementia in nursing homes. *BMC palliative care* 2016;15(1):55 doi: 10.1186/s12904-016-0127-2.

30. Charlson ME, Pompei P, Ales KL, et al. A new method of classifying prognostic comorbidity in longitudinal studies: development and validation. *Journal of chronic diseases* 1987;40(5):373-83

31. Volicer L, Hurley AC, Lathi DC, et al. Measurement of severity in advanced Alzheimer's disease. *J Gerontol* 1994;49(5):M223-6 doi: 10.1093/geronj/49.5.M223.

32. Waterlow J. Pressure sores: a risk assessment card. *Nurs Times* 1985;81(48):49-55

33. Cummings JL, Mega M, Gray K, et al. The Neuropsychiatric Inventory - Comprehensive assessment of psychopathology in dementia. *Neurology* 1994;44(12):2308-14

34. Cohen-Mansfield J, Marx MS, Rosenthal AS. A description of agitation in a nursing-home. *Journals of Gerontology* 1989;44(3):M77-M84 doi: 10.1093/geronj/44.3.M77.

35. Warden V, Hurley AC, Volicer L. Development and psychometric evaluation of the Pain Assessment in Advanced Dementia (PAINAD) scale. *J Am Med Dir Assoc* 2003;4(1):9-15 doi: 10.1097/01.jam.0000043422.31640.f7.

For peer review only - http://bmjopen.bmj.com/site/about/guidelines.xhtml

36. Kiely DK, Volicer L, Teno J, et al. The validity and reliability of scales for the evaluation of end-of-life care in advanced dementia. *Alzheimer Dis Assoc Disord* 2006;20(3):176-81 doi: 10.1097/00002093-200607000-00009.

37. Weiner MF, Martin-Cook K, Svetlik DA, et al. The quality of life in late-stage dementia (QUALID) scale. *J Am Med Dir Assoc* 2000;1(3):114-6

38. Zarit SH, Reever KE, Bach-Peterson J. Relatives of the impaired elderly: correlates of feelings of burden. *Gerontologist* 1980;20(6):649-55 doi: 10.1093/geront/20.6.649.

39. Zigmond AS, Snaith RP. The hospital anxiety and depression scale. *Acta Psychiatr Scand* 1983;67(6):361-70 doi: 10.1111/j.1600-0447.1983.tb09716.x.

40. Wimo A, Winblad B. Resource Utilization in Dementia: RUD Lite. *Brain Aging.* 2003;3:48-59

41. Gale NK, Heath G, Cameron E, et al. Using the framework method for the analysis of qualitative data in multi-disciplinary health research. *BMC medical research methodology* 2013;13:117-17 doi: 10.1186/1471-2288-13-117.

42. Department for Communities and Local Government. English Indices of Deprivation 2015. Open Government License 2015.

43. NHS Barnet Clinical Commissioning Group. Annual Report and Accounts 2014/15: Working with local people to develop seamless, accessible care for a healthier Barnet, 2015.

44. NHS Camden Clinical Commissioning Group. 2014/15 Final Annual Report and Outcomes: Working with the people of Camden to achieve the best health for all, 2015.

45. Engel SE, Kiely DK, Mitchell SL. Satisfaction with end-of-life care for nursing home residents with advanced dementia. *J Am Geriatr Soc* 2006;54(10):1567-72 doi: 10.1111/j.1532-5415.2006.00900.x.

46. Schnelle JF, Bates-Jensen BM, Chu L, et al. Accuracy of nursing home medical record information about care-process delivery: implications for staff management and

For peer review only

For peer review only - http://bmjopen.bmj.com/site/about/guidelines.xhtml

improvement. *J Am Geriatr Soc* 2004;52(8):1378-83 doi: 10.1111/j.1532-5415.2004.52372.x.

47. Cheek J, Gilbert A, Ballantyne A, et al. Factors influencing the implementation of quality use of medicines in residential aged care. *Drugs Aging* 2004;21(12):813-24 doi 10.2165/00002512-200421120-00005.

48. Seymour JE, Kumar A, Froggatt K. Do nursing homes for older people have the support they need to provide end-of-life care? A mixed methods enquiry in England. *Palliative medicine* 2011;25(2):125-38 doi: doi: 10.1177/0269216310387964.

49. Care Quality Commission. The State of Health Care and Adult Social Care in England: 2013/14: Williams Lea Group on behalf of the Controller of Her Majesty's Stationery Office, 2014.

50. Parks SM, Haines C, Foreman D, et al. Evaluation of an educational program for long-term care nursing assistants. *J Am Med Dir Assoc* 2005;6(1):61-5 doi: 10.1016/j.jamda.2004.12.001.

51. Chaudhury H, Hung L, Badger M. The role of physical environment in supporting person-centered dining in long-term care: a review of the literature. *Am J Alzheimers Dis Other Demen* 2013;28(5):491-500 doi: 10.1177/1533317513488923.

52. Clifford C, Badger F, Plumridge G, et al. Using the Gold Standards Framework in Care Homes: An evaluation of the Phase 2 programme. Secondary Using the Gold Standards Framework in Care Homes: An evaluation of the Phase 2 programme 2007. http://www.endoflifecare-intelligence.org.uk/view?rid=96.

53. Nolan MR, Davies S, Brown J, et al. Beyond 'person-centred' care: a new vision for gerontological nursing. *Journal of Clinical Nursing* 2004;13(3A):45-53 doi: 10.1111/j.1365-2702.2004.00926.x.

54. Lea E, Andrews S, Hill K, et al. Beyond the 'tick and flick': facilitating best practice falls prevention through an action research approach. *J Clin Nurs* 2012;21(13-14):1896-905 doi: 10.1111/j.1365-2702.2012.04121.x.

For peer review only - http://bmjopen.bmj.com/site/about/guidelines.xhtml

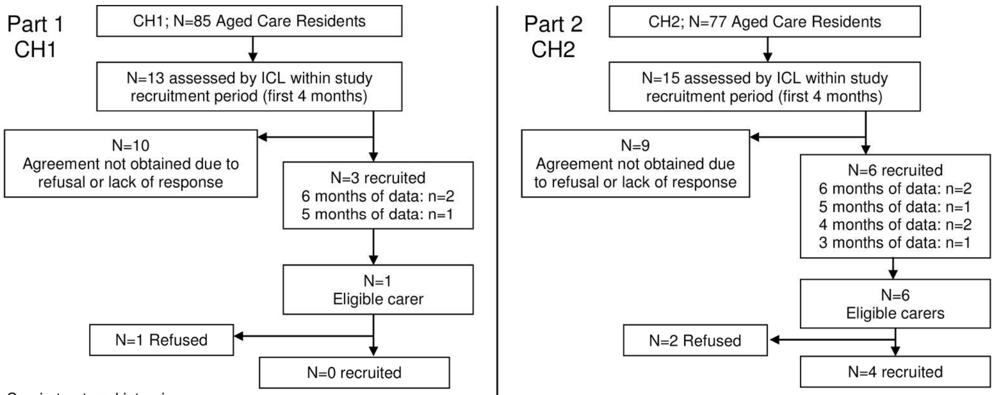

Semi-structured interviews

Role	Month 7	Month 11	Month 15
CH Manager	1	1	1
CH Deputy Manager	2	1	1
CH Nurse	2	3	2
CH Healthcare assistant	4	1	0
CH Activity Co-ordinator	1	1	0
General Practitioner	1	1	1
Geriatrician	1	1	1
Palliative Care Nurse	1	1	0
Mental Health Care Nurse	0	1	0
Family Carers	0		
Total	13	11	6

Role	Month 7	Month 11	Month 15
CH Manager	1	1	1
CH Deputy Manager	1	1	1
CH Nurse	0	2	2
CH Healthcare assistant	2	2	0
CH Activity Co-ordinator	1	1	0
General Practitioner	1	0	0
Geriatrician	NA	NA	NA
Palliative Care Nurse	0	1	0
Mental Health Care Nurse	NA	NA	NA
Family Carers	4		
Total	10	8	4

Figure 1: Flowchart of participants

190x120mm (300 x 300 DPI)

For peer review only - http://bmjopen.bmj.com/site/about/guidelines.xhtml

The Compassion Programme

(Care Of Memory Problems in Advanced Stages: Improving Our kNowledge)

Work-stream 3: Pilot study of enhanced integrated care for people with severe memory problems

Principal Investigator: Dr Louise Jones

Study Funders: Marie Curie Cancer Care

For peer review only

SYNOPSIS OF PROGRAMME 8
BACKGROUND 9
Epidemiological background 9
The clinical picture 9
Challenges 9
DEVELOPMENT OF THE COMPASSION MODEL OF ENHANCED CARE 10
Work stream 1 10
Work stream 2 10
Synthesis of findings and development of the enhanced model of care 11
AIMS AND OBJECTIVES OF PILOT STUDY 12
Objectives 12
RECRUITMENT AND CONSENT PROCEDURES 13
Location 13
Recruitment of care homes 13
Consent for implementing the enhanced model of care within the care home 13
Informing participants about the study 14
Recruitment of people with severe memory problems for evaluation of outcomes 14
Consent Procedures 16
Recruitment of family carers to give information for the evaluation 19
Recruitment of enhanced care team and care home staff to participate in qualitative interviews 20
Potential risks/strengths 21
PROJECT INTERVENTION 22
Preparing the Compassion Intervention manual for the enhanced model of care 22
Overview of the intervention 23
DATA COLLECTION 26

For peer review only - http://bmjopen.bmj.com/site/about/guidelines.xhtml

Enhanced care team process data 28

Care home level process data 28

Acceptability of the intervention to care home and enhanced care team staff 29

Outcomes for care home residents receiving the intervention 29

Outcomes for family carers 31

DATA ANALYSIS 34

Quantitative analysis 34

Qualitative analysis 34

Final analyses 35

Sample size 35

Health economics 35

PROJECT MANAGEMENT 35

Project Staffing 36

Core study team 36

Membership of our expert steering group 37

Patient and Public Involvement 38

STUDY OUTPUTS 38

REFERENCES 38

Appendix 1: Compassion intervention manual 42

Appendix 2. Bedford Alzheimer Nursing Severity (BANS) scale 42

Appendix 3: Waterlow scale 42

Appendix 4: Stirling Wound Assessment Scale 42

Appendix 5: Pain Assessment in Advanced Dementia (PAIND) 42

Appendix 6: Cohen Mansfield Agitation Inventory (CMAI) 42

Appendix 7: Neuropsychiatric Inventory (NPI) questionnaire 42

For peer review only

Appendix 8: Symptom Management at the End Of Life in Dementia (SM-EOLD) scale 42

Appendix 9: Quality of Life in late-stage Dementia (QUALID) 42

Appendix 10: Resource Utilisation in Dementia (RUD) - Lite.. 43

Appendix 11: The Comfort Assessment in Dying with Dementia scale (CAD-EOLD) 43

Appendix 12: The Zarit Burden Interview... 43

Appendix 13: Hospital Anxiety and Depression Scale (HADS) 43

Appendix 14: The Satisfaction with Care at the End-of-Life in Dementia Questionnaire (SWC/CAD-EOLD).. 43

Appendix 15: EQ-5D-5L 43

Appendix 16: Health Care Professional Qualitative Interview Schedule 44

Appendix 17: Family Carer Qualitative Interview Schedule.. 46

For peer review only - http://bmjopen.bmj.com/site/about/guidelines.xhtml

The Compassion Programme: WS3- pilot study Version 1, 24th January 2014

Table 1: Abbreviations

BANS	Bedford Alzheimer Nursing Scale
BPSD	Behavioural and Psychological Symptoms of Dementia
CAD-EOLD	The Comfort Assessment in Dying with Dementia Scale
CCG	Clinical Commissioning Group
CCI	Charlson Co-morbidity Index
CMAI	Cohen Mansfield Agitation Inventory
CRF	Case Report Form
CRM	Cluster Representation Mechanism
DSM-IV	Diagnostic and Statistical Manual
FAST	Functional Assessment Staging
GP	General Practitioner
HADS	Hospital Anxiety Scale
HCPs	Health Care Professionals
ICL	Interdisciplinary Care Leader
MRC	Medical Research Council
NPI	The Neuropsychiatric Inventory
PAINAD	Pain Assessment in Advanced Dementia
QALY's	Quality-Adjusted Life Years
QUALID	The Quality of Life in Late Stage Dementia
RAM	Rand Appropriateness Method
RUD-Lite	Resource Utilisation in Dementia
SM-EOLD	Symptom Management at the End of Life in Dementia Scale
SWC/CAD-EOLD	The Satisfaction with Care/Care at dying at the End of Life in Dementia

A note on terminology:

Two groups of carers need to be considered in people with severe memory problems: family (unpaid, informal) carers and paid (formal) carers. Here we use "family carer" as: "someone of any age providing unpaid support to family or friends" (Carers UK). No term is ideal and not all unpaid care is provided by families; "informal carer" is seen to minimise the carer role; and "unpaid carer" suggests a form of voluntary work. Thus "family carer" indicates the family member, friend or other close person acting as the primary unpaid carer for, or key decision maker/supporter of the person with severe memory problems. In addition we refer to "paid carers" in care homes and the community.

Only a third of people with dementia ever receive a formal diagnosis. Therefore in the following protocol, in earlier work streams and information sheets for family and paid carers we have used the term "*severe memory problems*". This allows us to recruit a more representative sample of all those with severe memory problems caused by dementia- many of whom may not have received a previous diagnosis.

For peer review only - http://bmjopen.bmj.com/site/about/guidelines.xhtml

SYNOPSIS OF PROGRAMME

The numbers of people living and dying with severe memory problems are increasing. Currently, people with severe memory problems often receive poor quality end of life care. The aim of our research, funded by Marie Curie Cancer Care as a three year programme grant, is to develop and pilot a complex intervention that aims to improve end of life care for people with severe memory problems. In years 1 and 2 we developed the intervention (Compassion), an enhanced model of existing care. In year 3 we now plan to pilot the Compassion Intervention and assess how it operates in practice.

Our research programme has been divided into three consecutive work streams: In work stream one we defined, in detail, the final disease trajectory of people with severe memory problems. We gained an in-depth understanding of the:

- clinical symptom burden;
- health and social care needs of people with severe memory problems,
- current pathways of care as they reach the end of life;
- needs of their family carers

In work stream two we used mixed methods (focus groups and individual interviews with people with early dementia, family carers and health and social care staff) to develop a complex intervention (Compassion) to improve end of life care. We have defined the core components of the Compassion Intervention which aims to enhance current care, and the circumstances needed to operationalize these. This protocol describes the final work stream in which we shall pilot this enhanced model of care in order to learn and understand how it might operate in practice and to obtain data to inform a future definitive trial.

For peer review only - http://bmjopen.bmj.com/site/about/guidelines.xhtml

BACKGROUND

Epidemiological background

Approximately 600,000 people in the United Kingdom (UK) have dementia (10% of those over 65 years). By 2026 it is estimated that this will approach 840,000 rising to 1.2 million by 2050 (1). One *third of people aged over 65 in the UK will die whilst suffering from dementia* (2). Systematic reviews suggest people with dementia have significantly increased mortality rates (3); even minor cognitive impairment is a strong independent predictor of mortality (4).

The clinical picture

People with severe memory problems can be identified using the Functional Assessment Staging Scale (FAST)(5). At level 6a and above the person will have difficulty putting clothing on properly without assistance, may have difficulty bathing properly, have urinary incontinence, be doubly incontinent or speak only a few words. A retrospective UK study of symptoms experienced in the last year of life by people with severe memory problems compared to cancer patients showed that the symptom burden and health care needs were comparable. In particular, 64% of those with severe memory problems experienced pain (compared to 59% with cancer), 46% breathing difficulties, 39% pressure sores and 86% difficulty with swallowing or loss of appetite (6;7). In people with severe memory problems acute physical illness may be an indicator of imminent death; 24% of those with moderate/severe dementia die after acute unplanned medical admissions compared to 7.5% of those without dementia (8).

Challenges

Essential components of good end of life care are often neglected in people with severe memory problems and referral to palliative care is rare (9) with fewer than 1% of hospice patients in Europe having a neurological diagnosis (10). In people with severe memory problems there are concerns about prognostic uncertainty and whether hospice staff can manage behavioural problems or communication difficulties (11;12);however, most symptoms experienced at the end of life such as pain or difficulties swallowing can be managed with good generalist care (13). Providing care in the usual place of residence is a major aim of the UK Government's End of Life Care Strategy; as well as benefitting patients and family carers this aims to save NHS costs by avoiding acute hospital admissions (13). A recent National Audit Office report indicated that about 50% of care home residents who died in hospital could have died within the care home setting (14). Evidence on how to improve care is limited. Based on available evidence, systematic reviews suggest the need for "care" tends to focus on specific interventions such as pain control, or the withdrawal of aspects of care e.g. *not* prescribing antibiotics (15;16). We suggest that good care requires a broader (but cost effective) palliative approach, tailored to meet the symptoms experienced by those with severe memory problems and also to meet the needs of family carers, particularly in the

terminal phase and in bereavement. Our work responds to UK government initiatives for care in dementia and at the end of life (13;17).

DEVELOPMENT OF THE COMPASSION MODEL OF ENHANCED CARE

We have used a realistic evaluation framework to develop the intervention, which incorporates information from a wide range of locations and sources. Improving end of life care is a complex undertaking. Our approach acknowledges the importance of context and social processes and allows us to find out about what mechanisms work, in what conditions, why, and how these produce particular outcomes. In brief, our findings so far have informed the enhanced model of care:

Work stream 1

In work stream 1 we conducted detailed research to define the symptom burden and needs of people with severe memory problems at the end of life, and their family carers. We have undertaken a longitudinal cohort study and have recruited 61 people with severe memory problems (FAST stage 7a and above, doubly incontinent and speaks only 5-6 words per day), 57 residing in care homes and four in their own homes. We have also recruited 26 of their family carers. Results from these studies showed how people with severe memory problems have multiple unmet needs, particularly with regards to management of pain and agitation. They are at high risk of pressure sores and have problems with eating and swallowing. There is lack of individual care planning and consideration of end of life care needs.

Work stream 2

Workshops with health and social care professionals

In work stream 2 in a first cycle of workshops we included a wide range of stakeholders and participants at all levels of responsibility. We conducted two workshops in London and one in each of Edinburgh, Solihull and Belfast. We used clinical vignettes describing people with severe memory problems and asked participants to consider how their care could be enhanced to provide solutions to the issues described.

In a second round of workshops we enhanced the content and face validity of our intervention, by using the RAND/UCLA approach (18). A key aspect of this approach is the Rand Appropriateness Method (RAM) which was used as a way to agree the key components of the intervention. To ensure that we took proper account of context further workshops were held across the four countries of the UK (sites in London, Edinburgh, Solihull, Belfast and Penarth). Before each workshop an online process managed by Survey Monkey, asked stakeholders to rank, for appropriateness, statements describing possible intervention components that were derived from the first round of workshops. Results were then analysed before each workshop and any points of disagreement were discussed further in the

For peer review only - http://bmjopen.bmj.com/site/about/guidelines.xhtml

workshop. Following this, participants were asked to rank statements describing components for necessity but independent of economic considerations. Data from all the workshops were pooled and a final bank of statements describing potential components of the enhanced model of care derived.

Interactive qualitative interviews with family carers and healthcare professionals

We conducted individual interactive interviews with 14 family carers and 14 health care professionals from a wide range of stakeholder sources including commissioners and health care assistants. Data analysis is on-going.

Workshops with family carers and people with early dementia

We conducted one workshop with five people with early dementia. We asked them to consider the type of care they would want in the future, especially towards the end of their lives. We also held a workshop with five family carers of people with severe memory problems. They were asked to suggest ways that care could be improved particularly considering end of life care planning and their own experiences of difficulties associated with the transfer of the person with severe memory problems to the acute hospital.

Policy documents

We undertook a detailed review of key documents currently operational in the four countries of the UK. We have focussed on documents that have been published since the National End of Life Care Strategy (2008) and Living Well with Dementia: a national dementia strategy (2009). Using a standardised template, we have summarised key statements arising and looked for similarities and differences in health and social care delivery across the four nations.

Synthesis of findings and development of the enhanced model of care

Findings from the cohort study workshop and interview data suggested a number of issues and ways that care could be improved, for example;

1. Importance of context: considerable regional variation in health and social care organisation and policy within the countries of the UK and Northern Ireland/ detailed repository of policy documents will be used to inform the reporting of our qualitative data and provide context for our recommendations.
2. Training for paid carers at the end of life, learning from hospice model
3. Training for paid carers on difficult conversations and care planning with family carers.
4. Improved staff skills and confidence/more trained nurses in ratio to health care assistants, a medical model like hospice care.
5. Need for enhanced bereavement support for paid and family carers including reflection on the death and care provided
6. Issues in care home culture/ prevent fear of deaths occurring

For peer review only - http://bmjopen.bmj.com/site/about/guidelines.xhtml

7. Lack of engagement of palliative care team/more education on dying with severe memory problems
8. Referrals and multi-disciplinary team working/ single point of contact, continuity of general practitioner (GP) care, rotating staff across environments to bring new learning, out of hours care from GP's who know patients.

The likelihood of successful implementation of our new enhanced care model requires that we understand the sociological theory underlying how our intervention would operate in practice (19). Following the RAM process, we scrutinised retained intervention components and mapped them to the theories described by Grol (19), categorising them according to which of the four operational levels identified by Ferlie and Shortell (20)and others such as Greenhalgh (21). We thought the components might operate on; 1) individual, 2) team, 3) group and 4) system levels. We explored both impact and process theories, operational and utilisation plans at the levels of the individual, social interaction, organisational context and economic/political context.

Details of the enhanced model of care for piloting are presented below (page 16 and Appendix 1).

AIMS AND OBJECTIVES OF PILOT STUDY

Our aim is to conduct a naturalistic pilot study to understand how the Compassion enhanced model of care operates in practice in two care homes in two different health and social care economies; one in the Camden Commissioning Group and one in the Barnet Commissioning Group.

Objectives

In the pilot study we will provide a coordinator with clinical skills- an "Interdisciplinary Care Leader (ICL)" who will coordinate and support the existing team of health and social care professionals working with participating care homes to enhance the management of people with severe memory problems. Our objectives will be met by collecting both quantitative data and qualitative data from the enhanced care team, care home staff and family caregivers

Specific objectives of the pilot study will be to:

1. Understand whether the enhanced model of care is feasible in the setting

2. Determine whether the enhanced model of care is acceptable to staff and family carers of people with severe memory problems in the care home

3. Understand facilitators and barriers to the implementation of the enhanced model of care by collecting qualitative data from paid and family carers on the experience of the intervention

For peer review only - http://bmjopen.bmj.com/site/about/guidelines.xhtml

4. Evaluate whether the enhanced model of care has an impact on a range of national key performance indicators and outcomes including those operating at a number of levels:
    a. Enhanced care team
    b. Care home environment and management
    c. Care home staff
    d. Family carers
    e. Residents with severe memory problems
5. Attempt to describe in detail the costs of delivering the intervention at our pilot sites and the costs of each of its sub-components to inform the commissioning process. These costs can be set against potential benefits and recommendations made

RECRUITMENT AND CONSENT PROCEDURES

Location

Through our previous cohort study we have worked with care homes in the Camden and Barnet Commissioning Group areas. We have chosen these as sites for our pilot intervention because we have previous experience of working with local clinicians including GPs and palliative care teams and they represent different location in terms of the socioeconomic and demographic composition of the area.

Recruitment of care homes

After gaining ethical consent for the study we will approach each care home manager by sending them a letter with brief study details. If the manager is interested, senior study staff will then visit the care home and provide further information regarding the project. We will, at the same time, also approach the proprietor or owner of the care home with similar information and seek their written consent for the home to participate in the enhanced care service and the collection of data from the home for the project outcomes.

Consent for implementing the enhanced model of care within the care home

We will be implementing our intervention of the enhanced care model at the level of the care home; our study can therefore be defined as a cluster pilot evaluation. The model of individual informed consent (or nominee assent) to receive the intervention may not be appropriate for a number of reasons. Firstly we are working with existing clinical services to offer an enhancement of usual care which is in line with the recent English Government Dementia and End of life care strategies. Secondly, we will be training and supporting the existing team to enhance and optimise practice, and thus may influence the care of all residents of the home.

For peer review only

We have consulted the UK Medical Research Council Guidance Document "Cluster randomised trials: methodological and ethical considerations." Using this framework our intervention is designated as "type A"- interventions that are received (or not) by a whole cluster together so that there is only one decision to be made for the care home. Therefore we use the appropriate Cluster Representation Mechanism (CRM), in our case, the nursing home owners who will give their consent for the intervention to be implemented in their care home. We will also obtain the permission of an ethics committee to implement the enhanced model of care so that the project undergoes appropriate ethical scrutiny. Some evaluation data will be collected at the individual level from the care home and these data will be anonymised, and therefore managers will not be providing any individually identifiable participant data.

Where we will be collecting individual level data, i.e. the qualitative evaluation, resident quality of life and measures from nursing home staff and family carers, we will obtain individual informed consent to participate. We will document how many participants who are approached do consent to us collecting individual level data as this may inform the planning of our future work.

Informing participants about the study

After gaining ethical consent to implement the enhanced care model the research team will meet with care home staff to inform them of the study and to answer or discuss their queries or concerns regarding the study.

Recruitment of people with severe memory problems for evaluation of outcomes

To collect evaluation data we will aim to recruit as many eligible residents as possible from each participating care home. Our criteria have been developed from an existing NHS and Social Care enhanced model of care from South London which has been used by the King's Fund as an example of UK best practice:

Resident Inclusion criteria

1. Aged over 65 years.
2. Severe memory problems indicating a clinical diagnosis of DSM-IV criteria for dementia (22).
3. Moderately severe or severe memory problems as classified on the Functional Assessment Staging Scale (FAST) grade 6a and above (5) see Table 2.

   Plus at least one of the following criteria:

- There are recurrent infections, significant weight loss and poor nutrition level, recurrent fevers, pains, falls, severe pressure ulcers that are not easily amenable to treatment, severe physical frailty.

For peer review only - http://bmjopen.bmj.com/site/about/guidelines.xhtml

- OR, the resident has severe, persistent distress (mental or physical) that is not easily amenable to treatment OR another condition (eg. co-morbid cancer) whose co-existence with dementia means that more intrusive treatments would be less appropriate.

Resident Exclusion criteria

- Residents who indicate either verbally or non-verbally that they do not wish to participate.

- Residents who are moribund, in a coma, or those where there are clinical concerns that may preclude them being approached.

Table 2: Functional Assessment Staging Scale (FAST)

STAGE	Description of functions lost
1	No difficulties, either subjectively or objectively
2	Complains of forgetting location of objects. Subjective word finding difficulties.
3	Decreased job functioning evident to co-workers; difficulty in traveling to new locations. Decreased organisational capacity.*
4	Decreased ability to perform complex tasks (e.g. planning dinner for guests, handling personal finances, difficulty marketing etc.)
5	Requires assistance in choosing proper clothing to wear for the day, season or occasion.
6a	Difficulty putting clothing on properly without assistance.
6b	Unable to bathe properly; e.g., difficulty adjusting bath water temperature) occasionally or more frequently over the past weeks.*
6c	Inability to handle mechanics of toileting (e.g., forgets to flush toilet, does not wipe properly or properly dispose of toilet tissue) occasionally or more frequently over the past weeks.*
6d	Urinary incontinence, occasional or more frequent.
6e	Faecal incontinence, (occasional or more frequently over the past week).
7a	Ability to speak limited to approximately a half dozen different words or fewer, in the course of an average day or in the course of an intensive interview.
7b	Speech ability limited to the use of a single intelligible word in an average day or in the course of an interview (the person may repeat the word over and over).
7c	Ambulatory ability lost (cannot walk without personal assistance).
7d	Ability to sit up without assistance lost (e.g., the individual will fall over if there are no lateral rests [arms] on the chair).
7e	Loss of the ability to smile.
7f	Loss of ability to hold up head independently.

*scored primarily on the basis of information obtained from a knowledgeable informant and/or caregiver.

For peer review only - http://bmjopen.bmj.com/site/about/guidelines.xhtml

Consent Procedures

Potential resident participants will have severe memory problems and may be physically frail. It is likely that they may not have the capacity to consent. Therefore our procedure has been developed to comply with capacity legislation governing England and Wales (Mental Capacity Act 2005, Sections 30-34) (see Figure 1).

Residents in care homes with severe memory problems

1. Although it is unlikely that any residents with severe memory problems will have capacity to give consent to participate in the study, The Mental Capacity act requires that we assume a person has this, unless shown otherwise. If the resident has capacity to consent to participate in the data collection, the care home manager will ask the resident if they are willing to see a member of the research team who will then consent them into the study. If capacity is not present the following steps will be taken.

2. On our behalf, the care home manager will attempt to identify their next of kin, family carer or someone close to the person (who does not receive remuneration for this role) who will act as a "personal consultee".

3. If the personal consultee is visiting the care home they will be approached by the care home manager and given verbal information and a written information sheet about the study. They will be encouraged to consider the person's prior wishes or thoughts regarding taking part in research. They will be asked to sign and return a reply slip indicating if they give consent for their contact details to be passed to the research team. If no reply slip is returned to the research team within 14 days, the research team will contact the care home to inform them of this. The care home will then contact the family carer only once and ask if they agree to the home giving the research team their contact details so the research team can contact them regarding the study. If the family carer does not give permission for the care home to give their details to the research team, no further contact will be made. If permission is granted a member of the research team will telephone the family carer. If the personal consultee agrees to the person taking part they will be sent an information sheet and a family carer assent form to sign or be invited to visit the care home and meet with the research team to do this in person. If no assent form is returned within 14 days then the research team will telephone the personal consultee on the maximum of two occasions to see whether they are still interested in participating.

4. If the personal consultee is not available in the care home (i.e. lives a distance from the home or is not able (or wishes) to visit) the care home manager will post the study information sheet to them. They will also be sent a reply slip to sign and return on whether they give permission for the care home to pass their contact details onto the research team. If no reply slip is returned to the research team within 14 days the research team will contact the care home to inform them of this. The care home will contact the family carer only once and ask if they agree to the home giving the research

For peer review only - http://bmjopen.bmj.com/site/about/guidelines.xhtml

team their contact details so the research team can contact them regarding the study. If the family carer does not give permission for the care home to give their details to the research team, no further contact will be made. If permission is granted a member of the research team will telephone the family carer. If the consultee agrees to the person taking part they will be sent a family carer assent form to sign or invited to visit the care home to meet with the research team to do this in person. If no assent form is returned within 14 days then the research team will telephone the personal consultee to see whether they are still interested in participating.

5. If a) no friend or next of kin that can act as a personal consultee is documented in the clinical notes, or, b) after three attempts at telephone contact over one week by the care home manager, they are unable to contact a personal consultee, then the research team will use a professional consultee. This will be defined as a senior experienced health or social care worker who is not directly involved in the research or care of the patient. Through the cohort study we have identified skilled professionals within each CCG who are not involved in the research project or in the patient's direct clinical care and are happy to act in this role. These "consultees" will be given information about the study and training on their responsibilities by the research team. They will follow a structured procedure to give assent for the person's participation in the study and sign their assent for this.

For peer review only - http://bmjopen.bmj.com/site/about/guidelines.xhtml

Figure 1. Summary of consent procedures for the collection of individual level outcome data

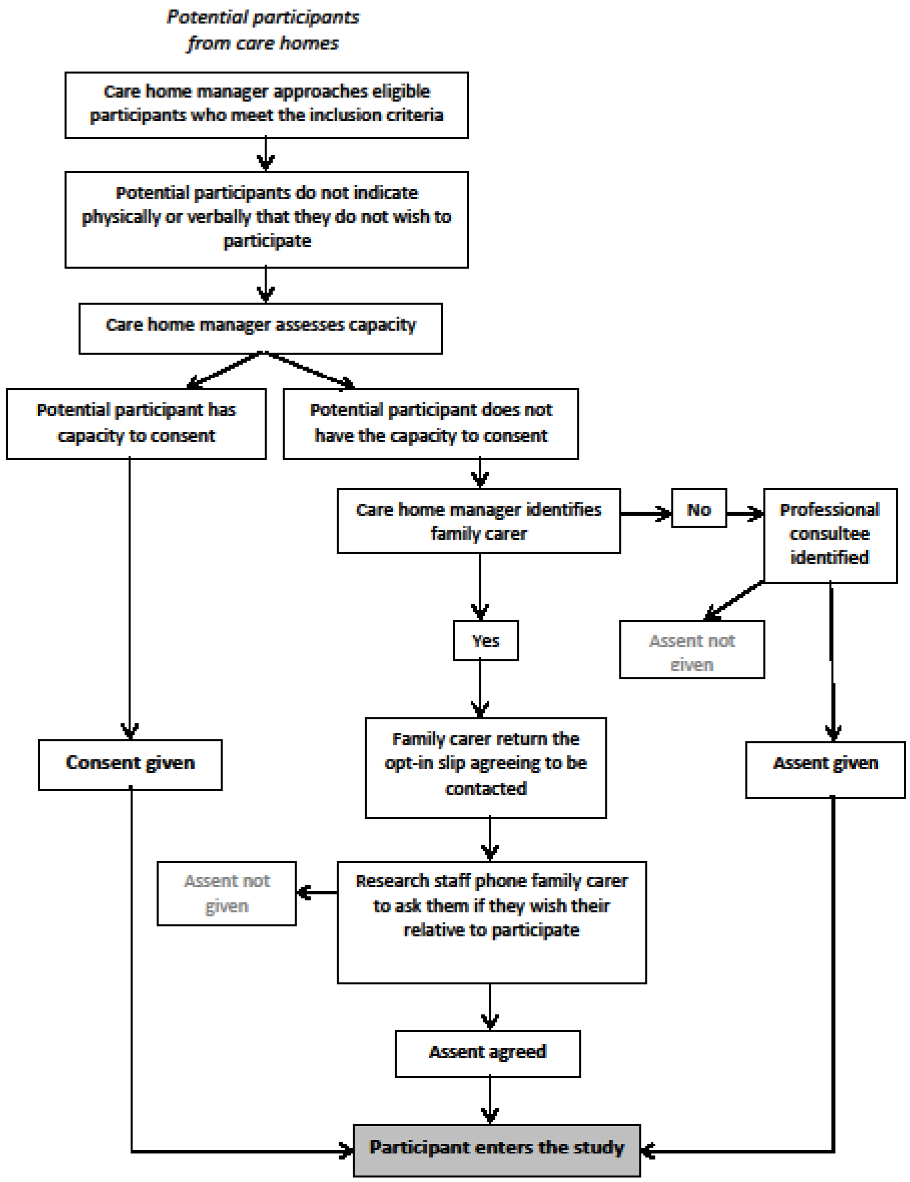

Recruitment of family carers to give information for the evaluation

We wish to evaluate the opinions of family carers of residents with severe memory problems who have received the enhanced care service. We will only recruit carers of people with severe memory problems who have already entered the study as the recruitment of dyads will enable us to link the experiences of people with severe memory problems and their family carers.

Family carer inclusion criteria

- If the resident with severe memory problems does not have capacity this will be the main family carer (e.g. family member or friend in regular contact and who is the next of kin or a 'key decision maker', identified by the care home manager). If the resident does have capacity we will ask them to nominate who they think is their family carer.
- English language sufficient to complete the study ratings.

Family carer exclusion criteria

- Family carers where there are clinical concerns that may preclude them being approached.
- Family carers aged 16 and under.
- If for any reason during the study the family carer becomes unavailable/unable to give consent we will withdraw the family carer from the study.

Consent procedure

Family carers of residents who do not have capacity to consent will be asked if they wish to participate when we recruit their relative/friend into the study. We will explain that we are interested in exploring their experiences of the enhanced care service now and, should the person die, their experiences of bereavement. They will be informed that they will have two weeks to decide whether they want to participate and can, if they wish, take time to discuss the study further, with other family members/friends, GP and/or research staff. They will be informed that if they decide not to take part that this will not adversely affect the care of their friend/relative or the support they receive as a family carer in any way. If the family carer agrees to participate then a consent form will be sent to them (or given to them when we see them face to face). If the consent form is not returned within 7 days we will contact them again to check whether they still wish to participate. There will be a maximum of two attempts to contact.

Where the resident does have capacity to consent for themselves we will need to recruit family carers independently. The care home manager will approach the family carer and given verbal information and a written information sheet about the study. They will be asked to sign and return a reply slip indicating if they give consent for their contact details to be

For peer review only - http://bmjopen.bmj.com/site/about/guidelines.xhtml

passed to the research team. If no reply slip is returned to the research team within 14 days, the research team will contact the care home to inform them of this. The care home will then contact the family carer only once and ask if they agree to the home giving the research team their contact details so the research team can contact the family carer regarding the study. If the family carer does not give permission for the care home to give their details to the research team, no further contact will be made. If permission is granted, a member of the research team will telephone the family carer. If they agree to participate they will be sent an information sheet and a consent form to sign or be invited to visit the care home and meet with the research team to do this in person. If no consent form is returned within 14 days then the research team will telephone the family carer on the maximum of two occasions to see whether they are still interested in participating.

If the family carer is not available in the care home (i.e. lives a distance from the home or is not able (or wishes) to visit) the care home manager will post the study information sheet to them. They will also be sent a reply slip to sign and return on whether they give permission for the care home to pass their contact details onto the research team. If no reply slip is returned to the research team within 14 days the research team will contact the care home to inform them of this. The care home will contact the family carer only once and ask if they agree to the home giving the research team their contact details so the research team can contact them regarding the study. If the family carer does not give permission for the care home to give their details to the research team, no further contact will be made. If permission is granted a member of the research team will telephone the family carer. If they agree to take part they will be sent a consent form to sign or invited to visit the care home to meet with the research team to do this in person. If no consent form is returned within 14 days then the research team will telephone the family carer to see whether they are still interested in participating.

Recruitment of enhanced care team and care home staff to participate in qualitative interviews

Recruitment and consent of heath care professionals and paid carers

The Interdisciplinary care leader (ICL)/care home manager will identify Healthcare Professionals (HCPs) and paid carers who have been involved in providing care and support to people with severe memory problems in the care home; this will include those from a variety of disciplines and organisations who go into the care home for example, care home staff, general practitioners, speech and language therapists, social workers etc. They will be asked whether they are interested in participating in the research and whether they are happy if the research staff can be given their contact number at work. The researcher will then contact them to discuss the study in detail.

The research team at the research site will ask if they are interested in participating, provide them with an information sheet and ask if they would be happy to participate. They will have

For peer review only - http://bmjopen.bmj.com/site/about/guidelines.xhtml

at least 48 hours to consider whether they wish to participate. They will be informed that their participation is voluntary and individuals or their organisation will not be identifiable in anyway and that all information will be anonymised and kept confidential. If the HCP/paid carer decides they do wish to take part in the study they will be asked to sign a consent form. We intend to conduct a maximum of 10 interviews per care home.

Potential risks/strengths

A strength of our approach is that we have developed our intervention using information gathered from a range of participants. These include health and social care staff, people with early dementia and their family and other unpaid carers. The intervention is also an enhancement of usual care which merely formalises recommendations made in exiting policy documents such as the English National Dementia and End of Life Care Strategies. It is being run in conjunction with established clinical services, adding to their capacity to manage and improve the care of people with severe memory problems who reside in a care home. It will not inhibit the "usual care" that they should receive and **clinical responsibility for the resident's care will, as per usual practice, remain with their GP**. The measures we use to evaluate outcomes are mostly observational with no additional burden or discomfort to the patient and should be part of good routine end of life care (23) therefore the risk of any harm is minimal. If the person with severe memory problems does become upset or uncomfortable in any way with the assessment process, the researcher will stop the assessment immediately and report this to the care home staff and/or the resident's family carer.

We do understand that this research may touch on some sensitive issues for family carers and paid staff, however, the Marie Curie Palliative Care Research Unit has extensive experience of conducting interviews with bereaved relatives of patients with malignant and non-malignant conditions, including end-stage renal disease and advanced dementia (24-27).

In the unlikely event that family carers do become upset in taking part in the study, the researcher will stop the assessment. They will with the family carer's permission ask them if they want to have a break from the assessment, continue or to stop. It is natural that family carers may at times feel emotional when talking about their role or their relative/friend. If the family carer wishes to stop then the assessment will be brought to a close. If they become upset or if their scores on the Hospital Anxiety and Depression Scale (HADS) scale suggest clinical depression or anxiety they will be given information regarding support networks/agencies to contact should they wish, for example, the Admiral Nurse DIRECT or Alzheimer's Society National Help lines, their General Practitioner or other relevant service if there is prior involvement.

The research staff collecting data will be given training and supervision on all of the study assessment tools and family carer interview schedule. The research team will review their recruitment procedures after one month. Any problems will be documented. If substantial

For peer review only - http://bmjopen.bmj.com/site/about/guidelines.xhtml

changes to the protocol are needed we will seek approval of proposed changes from the Research Ethics Committee.

If we discover issues of malpractice, maltreatment or serious neglect, to the degree that the relevant local authority's safeguarding procedures are triggered, we will in this circumstance be required to break patient confidentiality and inform the relevant authorities, following whichever standard local authority safeguarding procedures are in operation.

It is important that issues of sustainability are considered so that we do not leave the care home unsupported after the enhanced care pilot has finished. Evidence suggests that even after the research team have finished the pilot, benefits may persist and that local services maintain and further develop new interventions , so maintaining on going improvements in care; "dynamic sustainability" (28) . One aspect to sustain any benefits is that participating nursing homes will be provided with a structured training programme designed to meet any training/care needs identified during the cohort study.

PROJECT INTERVENTION

Preparing the Compassion Intervention manual for the enhanced model of care

We have produced a written document to describe Compassion in manual form as recommended by the Medical Research Council (MRC) guidance on the development of complex healthcare interventions 2008. This provides a framework by which the intervention can be sustained and becomes replicable at a number of sites. It describes for participating partners the core intervention components and the steps required to implement components. There are two core components:

1. Facilitation of integrated care for people with severe memory problems and their family carers.

2. Education, training and support for health and social care professionals at all levels and for family carers.

The manual in its development was reviewed by key stakeholders during a focus group (care home managers, representatives from palliative care, GPs and care of the elderly physicians). Necessary changes were made, and further amendments were made by the programme grant expert steering group.

The manual describes in detail processes which aim to improve end of life care for people with severe memory problems by:

- Enabling holistic individualised person centred care.

For peer review only - http://bmjopen.bmj.com/site/about/guidelines.xhtml

- Providing an interdisciplinary care leader (ICL) who will act as a central resource for health care professionals, care home staff and family carers involved in the care of people with severe memory problems.
- Developing links and joint working between all those involved in the care and management of people with severe memory problems to establish a model of integrated care.
- Improving the understanding of what is meant by an individualised personal care plan and how such a plan might be worked out and used in practice
- Providing support to front-line staff and managers in care homes to enable them to hold uncertainty and manage risk in people with severe memory problems to avoid unnecessary place of care transfers.
- Identifying, facilitating and supporting the training needs of care home staff in the care of those with severe memory problems.
- Recognising the needs of family carers, including being alert to possible anxiety and depression.
- Supporting the commissioning of effective and sustainable systems to deliver these objectives.

Overview of the intervention

The enhanced model of care delivered by the intervention will run for 6 months. For a detailed description of the intervention see Appendix 1. Facilitating effective clinical change in complex health and social care systems can be challenging. Compassion aims to set out a clear pathway of the actions that need to be taken, and by whom, for its effective implementation. This includes integrating change within existing systems to underpin current expertise and developing an understanding of what is needed for continued best practice. The key people involved in delivering Compassion for the pilot phase are listed below.

Interdisciplinary Care Leader (ICL)

The ICL will be a new post funded through the Compassion research project. The main responsibilities of the ICL will include:

- Developing an understanding of the health and social care professionals, pathways and services relevant to the care home residents with severe memory problems that are currently available.
- Working with the care home staff to identify and assess residents suitable for inclusion in the intervention.
- Establishing who the members of the core team involved in care will be, co-ordinating the weekly meetings and working within the core team to develop and implement personalised care plans for each resident included in the intervention.

For peer review only - http://bmjopen.bmj.com/site/about/guidelines.xhtml

- Establishing the wider clinical team, co-ordinating monthly meetings and maintaining effective communication to facilitate integrated co-ordination of care and the development of good working relationships between all health and social care professionals involved in the care of those with severe memory problems.
- Working with the care home staff to identify and support their educational and training needs, including fostering a culture of respect, dignity and quality of care for all residents and their family carers supporting someone with severe memory problems.
- Meeting with and supporting family carers to ensure their needs and wishes are understood.
- Collecting process data to support evaluation of the intervention.

The ICL will receive training in standard procedures with regard to clinical and information governance, safeguarding of vulnerable adults and the Mental Capacity Act prior to commencing in post. He/she will keep an anonymised reflective diary and will be supported by the research team at the Marie Curie Palliative Care Research Unit.

The Core Team

The core team comprises a range of existing staff who already regularly visit the homes and are responsible for overseeing the medical, nursing and social care needs of residents. During the intervention they will work with the ICL and are the key personnel required to deliver Compassion. The team will meet weekly and includes:

- Clinical Lead Professional (GP supporting the care home, Geriatrician or Old Age Psychiatrist)
- Member of care home staff (care home manager or floor/ unit manager)
- Interdisciplinary Care Leader

The Wider Team

The wider team includes local health and social care professionals and specialist services involved in the care of people with severe memory problems. The team includes staff from General Practice, Care of the Elderly, Old Age Psychiatrist, Palliative Care, Social Services and Community services such as District Nursing, Social Workers, Speech and Language Therapy, Dietetics, Tissue Viability, Physiotherapy and Occupational Therapy. However, the exact composition will depend on local working practices and the availability of key personnel. The wider care team will meet monthly with the core team; meetings may be face to face or via links such as conference calling. The organisation, communication, facilitation and recording of meetings will be the responsibility of the ICL but the team will be required to appoint a lead to chair the meetings.

For peer review only - http://bmjopen.bmj.com/site/about/guidelines.xhtml

The Compassion Programme: WS3- pilot study Version 1, 24th January 2014

The Compassion flow chart, shown below, outlines the steps of the intervention pathway, the roles and the responsibilities of those participating, and the work required within each step of the pathway.

Compassion **Intervention flow chart for pilot study**

Step 1: Engage local stakeholders and analyse local need, interest and readiness for change. Obtain necessary permissions (project team)

Step 2: Map existing services (ICL and project team)

Step 3: Identify Clinical Lead Professional and establish membership of the core and wider team. Hold initial orientation meeting (ICL and project team)

Step 4: ICL works alongside care home staff to scope, identify and respond to training needs

Step 5: ICL and care home staff identify residents who may benefit from the intervention

Step 6: ICL and care home staff conduct initial assessment of care needs

Step 7: ICL and care home staff discuss care needs and concerns with family carer and assess carer needs

Step 8: Assessment of care needs discussed at weekly core team meeting with clinical lead and care plan agreed

Step 9: ICL discusses and implements the care plan with the care home staff and family carer (if available)
ICL meets with night staff as appropriate to plan for possible out of hours events

Step 10; ICL reviews residents with care home staff at regular intervals or after sentinel events
ICL reports back to core team and family carer, care plan reviewed and amended if necessary

Step 11: Wider team meet monthly to discuss complex cases and review care plans
Wider team consider significant events, critical incident analysis (cyclical performance review)

Step 12: After death family carer seen by ICL and signposted to additional support as required
Death is reviewed in wider team meeting

Ongoing training and facilitation model

Education and Training considerations

Current education and training provision on end of life care in for people with severe memory problems within the CCG area will be scoped and mapped.

The ICL will work with the care home to help to establish and address the training and educational needs of their staff. This will be primarily by working alongside the staff but may also include one to one reflective discussions with key staff members. Learning and training needs will be addressed in a variety of ways but will include shared working and mentoring, use of online learning resources and formal topic based teaching sessions from local services

For peer review only - http://bmjopen.bmj.com/site/about/guidelines.xhtml

and experts if required. Training will be feasible within timing, staffing and financial constraints and will be agreed with the care home manager.

Education and training provided as part of the intervention will aim to enable care staff to recognise and respond effectively to the needs of people with severe memory problems and to support family carers with increased confidence and competence. Education and training will link to the core competencies outlined in the document "Developing end of life care practice: A guide to workforce development to support social care and health workers to apply the common core principles and competences for end of life care" (Skills for Care, Skills for Health, National End of Life Care Programme. 2012) and will include, communication skills, with residents suffering from severe memory problems and their family carers, assessment and care planning, advance care planning, symptom management to maintain comfort and wellbeing, knowledge and values.

DATA COLLECTION

Our enhanced care model may have an impact at a number of levels, for example on the individual resident and their family carer, on care home staff, at processes which occur at the level of care home management and on the intervention team itself. This is a feasibility study and thus we have to collect data on a range of outcome and process measures, to detect any impacts which the intervention may have on a complex care system and those who reside and work within it. Our measures map onto our key objectives which are to understand the barriers and facilitators to the implementation of the enhanced care model, to assess feasibility and acceptability of the model and to understand the impact of this model on individual residents and their family carers. Data collection is summarised in table 3 (below).

Process data: these will be collected by the ICL and the team delivering the enhanced service. It is evaluation data much of which is already routinely collected within this setting and is required for national NHS and social care end of life care targets and key commissioning performance indicators (marked with * in outcomes table). The data will give us information on the feasibility and acceptability of the intervention and barriers and facilitators to its implementation. This data will be anonymous at source and not collected at an individual level.

Data on individual outcomes: these data will be collected by the research team who will work independently of the enhanced service implementation team. We will collect data from residents with severe memory problems who receive the service, their family carers, individual care home staff and individual members of the intervention team. Thus to collect these data will require individual informed consent (or in the case of care home residents who may lack capacity, assent). For further information in our consent processes please see page 8.

For peer review only - http://bmjopen.bmj.com/site/about/guidelines.xhtml

The Compassion Programme: WS3- pilot study Version 1, 24th January 2014

Table 3: Process and outcomes measures

	Process data	Information on individual outcomes and perspectives
**Enhanced care team**	• Number of residents reviewed<br>• Contacts with family carers<br>• Attendance at team meetings<br>• Number of individual care plans made*<br>• Referral to other specialists	• Experience of participating in the enhanced care intervention<br>• Experience of participating in the enhanced care intervention<br>• ICL reflective practice diary<br>• Barriers and facilitators to the enhanced care intervention
**Care home level data**	• Use of pain tools *<br>• Number of residents with pain management plans*<br>• Recording of surrogate decision makers*<br>• Number of residents with resuscitation status recorded*<br>• Number of deaths within the care home in the last month*<br>• Recording of preferred place of death*<br>• Number of deaths in the usual/preferred place of care*<br>• Numbers of ambulance transfers to acute care*<br>• Visits by out of hours primary care*	
**Care home staff**	• Education and training needs of care home staff and how these were addressed	• Experience of participating in the enhanced care intervention
**Family carer**	• Numbers who have a needs assessment	• Satisfaction with the intervention<br>• Burden<br>• Anxiety and depression<br>• Satisfaction with general care<br>• Quality of life<br><br>If the resident dies:<br>• Satisfaction/quality of end of life care
**Care home resident**	• Number of baseline assessments<br>• Number of review assessments	• Severity of impairment<br>• Pressure sores risk and severity<br>• Pain<br>• Agitation<br>• Behavioural Symptoms<br>• Symptom management at end of

For peer review only - http://bmjopen.bmj.com/site/about/guidelines.xhtml

		life<br>• Quality of life<br>• Resource Utilisation<br>• Number of hospital admissions<br>• Sentinel events<br>• Use of parenteral feeding<br>• Use of personalised care plans<br>• Death in usual /preferred place of care<br><br>If the resident dies:<br>• Use of medication<br>• Burdensome interventions<br>• Adherence to individual care plan

*evaluation data which is already routinely collected within this setting and is required for national NHS and social care end of life care targets and key commissioning performance indicators

Enhanced care team process data

The ICL will record process data on a pro-forma to enable monitoring and evaluation of the enhanced service. These data will be collected on a monthly basis, it will be anonymised and not identifiable at the level of individual residents. These data give us information on the feasibility and acceptability of the intervention and will include:

- Number of residents reviewed by the enhanced care team
- Number of contacts with family carers (by phone and face to face)
- Attendance at team meetings
- Number of individual care plans made by the enhanced care team
- Referral to other specialists outside the care home, for example dietician, speech and language therapists, tissue viability nurses
- Education and training needs of care home staff and how these were addressed i.e., by individual training sessions, referral to online training resources

In addition the ICL will keep a reflective diary (carefully written to ensure anonymisation and confidentiality) recording their experiences of scoping for and implementing the intervention, including notes on care home dynamics, their interactions with the core and wider teams, the care being delivered by staff and any changes being observed that may not be captured by the outcome measures.

Care home level process data

This data will be collected on a monthly basis by the care home manager (to comply with the UK Data Protection Act 1998) in collaboration with the ICL. It will be anonymised and not identifiable at the level of individual residents. Much of this data should already be routinely

For peer review only - http://bmjopen.bmj.com/site/about/guidelines.xhtml

collected and is required by governance organisations and local health and social care commissioners in their assessment of whether services are meeting statutory key performance indicators. The ICL will document:

- Whether pain tools are being routinely used in the care home
- The number of residents with pain management plans
- The recording of surrogate decision makers in the care home records
- Number of residents with resuscitation status recorded
- Number of deaths within the care home in the last month
- Recording of preferred place of death
- Number of deaths in the usual/preferred place of care
- Numbers of ambulance transfers to acute care
- Visits by out of hours primary care

Acceptability of the intervention to care home and enhanced care team staff

We will explore the experience of participating in the intervention with members of the enhanced care team and care home staff. We will conduct qualitative interviews with a purposively sampled selection of staff at the end of the project. These interviews will occur at the end of the intervention period. We will explore the staff experience of the enhanced care team using a structured topic guide which maps onto key areas of current UK end of life and social care policy, for example, how they found working with the ICL, whether the ICL enhanced the way they performed their role, whether the enhanced care model changed how they recognised symptoms such as pain and how these were managed (for interview guide see Appendix 2). Interviews will be audio taped and transcribed verbatim (anonymised). They will last no longer than one hour and participants will be offered the opportunity to review transcripts to ensure accuracy.

Outcomes for care home residents receiving the intervention

These data will be collected independently by the research team only on those residents who have given informed consent to participate or whose relatives have given signed assent for their participation

Demographic information (age, marital status, previous employment) will be collected at the beginning of the evaluation. Severity of dementia will be measured using the FAST scale. At study entry information from GP notes will be obtained by the research team, including: medical co-morbidity (the Charlson Co-morbidity Index (CCI): which includes 19 diseases weighted on the basis of their association with mortality). This allows for the documentation of painful co-morbidities (29).We will document medications from GP prescriptions (e.g. antibiotics, analgesia and antipsychotics). We will document the presence of advance directives, care plans and specific requests regarding hospitalization and resuscitation.

For peer review only - http://bmjopen.bmj.com/site/about/guidelines.xhtml

Clinical assessment

Researchers will assess participants and document their symptom burden with the proforma used in our cohort study (25). It consists of a typical, detailed generalist approach to palliative care.

Functional Assessment Staging Scale (FAST): This observational scale describes a continuum of seven successive stages of functional impairment, from normality to the most severe dementia (5). (See Table 2; ***Recruitment of people with severe memory problems for evaluation of outcomes***)

Bedford Alzheimer Nursing Scale (BANS): This brief 8-item scale is used to stage the level of severe memory impairment in terms of factors such as eye-contact and speech (30). (See Appendix 2).

Pressure sores risk and severity: The Waterlow Scale will be used for the assessment of risk for developing pressure sores (See Appendix 3). It has high inter-rater reliability and sensitivity (31). The Stirling Scale measures the extent of damage from a scale of 1, Non-blanching erythema of intact skin to 4, full-thickness wound, which involving subcutaneous tissue and the deep fascia (32). (See Appendix 4).

Observational scales completed with care home staff or family carers

Pain Assessment in Advanced Dementia (PAINAD): This measures pain during care tasks and at rest. A comprehensive systematic review has identified this tool as having sensitivity and clinical utility (33). (See Appendix 5).

Cohen Mansfield Agitation Inventory (CMAI): This observational scale rates a range of behaviours many of which are relevant and challenging in dementia, for example wandering, grabbing on to people and pushing. It enables measurements over short timescales and is completed with a carer or staff member (34). (See Appendix 6).

The Neuropsychiatric Inventory (NPI): is a brief caregiver questionnaire that is used to assess behavioural and psychological symptoms commonly observed in residents with severe memory problems (BPSD) i.e. psychosis, mood disturbances, agitation, personality changes, pacing, wandering, and appetite disturbances. Its use in primary care is recommended, as it not only assesses the severity of the symptom for the patient but also the distress that the symptom causes the caregiver (35). (See Appendix 7).

Symptom Management at the End of Life in Dementia Scale (SM-EOLD): Is a tool used to assess comfort and pain during the prior 30 days (36). (See Appendix 8).

The Quality of Life in Late Stage Dementia Scale (QUALID): is a validated scale that assesses quality of life over the prior week (37). (See Appendix 9).

For peer review only - http://bmjopen.bmj.com/site/about/guidelines.xhtml

Resource Utilisation in Dementia (RUD)-lite: Resource Utilisation in Dementia (RUD)-lite: Is a short version of the RUD structured interview to assess costs of care including patient accommodation, informal care, community care and hospitalizations (38). (See Appendix 10).

Monthly follow up assessments

Participating residents will be reviewed every four weeks in the care home by the research team, for a maximum of six months, or until death. We will repeat measures: the generalist clinical assessment; Waterlow, Sterling, CMAI, NPI, BANS, PAINAD, SM-EOLD, QUALID, and the RUD-lite. We shall also record prospectively the number of acute hospital admissions, the reasons for these, "burdensome interventions" e.g. enteral feeding tubes (27) and "sentinel events", defined as "new medical conditions that have the potential to lead to a significant change in health status and a shift in the goals of care" e.g. pneumonia, hip fracture (6). Prescription medications and use will also be collected.

Data collection post death

The Comfort Assessment in Dying with Dementia Scale (CAD-EOLD) (36) (See Appendix 11) will be completed with care home staff within 14 days of the resident's death to assess their level of comfort and pain in the seven days prior to their death. Through a review of care home notes we shall record use of medication at the end of life (i.e. "just in case" prescribing, opiates, syringe drivers and artificial hydration or nutrition), sentinel events and burdensome interventions. We will examine adherence to any individual care plans which were made.

Outcomes for family carers

Data will be collected independently by the research team during face to face interviews at study entry within 14 days of the initial resident assessment and then every month, by post or over the telephone (family carers' preference). If family carers are un-contactable for more than 2 months or withdraw from the study we will document the reason and aim to continue to include the person with severe memory problems in the study, unless the carer specifically withdraws their assent.

At project start

We will collect demographic data to include age, sex, ethnicity, education, employment and occupation (present or previous), marital status, relationship to the care home resident, the number of years spent caring and any other caring responsibilities e.g. children under 18 years of age.

For peer review only - http://bmjopen.bmj.com/site/about/guidelines.xhtml

At project start and each monthly follow-up

As with the participating residents, measures (listed below) will be repeated at monthly intervals. We shall inquire about contact with the ICL and whether end of life issues have been mentioned.

Zarit Burden Interview: a 22-item self-report questionnaire, the most consistently used measure of carer burden in dementia. The questionnaire asks the carer to reflect on how they feel when they are caring for the person (39). (See Appendix 12).

Hospital Anxiety and Depression Scale (HADS): a self-report instrument for clinically significant anxiety and depression (40). (See Appendix 13).

The Satisfaction with Care at the End of Life Scale in Dementia Scale **(SWC/CAD-EOLD):** a validated tool that quantifies overall satisfaction with care in advanced dementia. This brief 10-item self-administered questionnaire assesses the caregiver's level of satisfaction with decision-making, medical and nursing care, and their understanding of the condition of the person with dementia (See Appendix 14). The CAD version is used to assess care received around the time of death (36) (see data collection in bereavement - below). (See Appendix 11).

EQ-5D-5L: this instrument is an index-based utility set for the calculation of quality-adjusted life years (QALYs) used to inform health economic evaluations of healthcare interventions (41). (See Appendix 15).

Qualitative interviews

To gain a deeper understanding of how they experience the enhanced care model and working with the ICL we shall offer qualitative interviews with the research team and to all participating family carers in a place of their choice. These will occur at the end of the feasibility study for the enhanced model of care or in bereavement if the resident dies (for interview schedule see Appendices 16 and 17).

Data collection in bereavement

To gain a deeper understanding of the circumstances surrounding the death and the views of the carer on which aspects of care were or were not satisfactory, where possible we shall ask additional questions all bereaved family carers. In this case we will ensure these interviews take place two months after bereavement, this has been found to be the optimal time for such work whereby the carer feels ready to think about their loss but still has sufficient recall of events (42;43). We found in our cohort study that these interviews are acceptable (we have completed ten so far) and family carers are keen to reflect on their experiences (25). The SWC-EOLD scale will be completed to assess family carer's level of satisfaction with care

For peer review only - http://bmjopen.bmj.com/site/about/guidelines.xhtml

and the CAD-EOLD to assess the resident's level of comfort and pain in the 7 days prior to their death from the carer's perspective.

We will items from a topic guide similar to that used successfully in our cohort study which was acceptable to family carers (44). Interviews will be audio taped and transcribed verbatim (anonymised). They will last no longer than one hour and carers will be offered the opportunity to review transcripts to ensure accuracy.

Table 4: Summary of data collection

	Project start	During project (monthly for 6 months)	After death/ in bereavement	After project ends
Enhanced care team process data	x	x		
Care home level data	x	x		
Paid carers/ enhanced care team staff qualitative interviews				x
**Residents**				
Demographic information	x			
FAST scale	x			
Charlson Co-morbidity Index	x			
Medications	x	x		
Prior advance care plans and wishes documented	x			
Symptom burden/generalist clinical assessment	x	x		
Bedford Alzheimer Nursing Scale	x	x		
Pressure sore risk and severity	x	x		
Pain Assessment in Advanced Dementia	x	x		
Cohen Mansfield Agitation Inventory	x	x		
Neuropsychiatric inventory	x	x		
Symptom Management at the End of Life in Dementia Scale	x	x		
Quality of Life in Late dementia Scale	x	x		
Resource Utilization in Dementia Scale	x	x		
Burdensome interventions		x		
Sentinel events		x		
Comfort Assessment in Dying Scale			x	
**Family carers**				
Demographic data	x			
Zarit Burden Interview	x	x		

For peer review only - http://bmjopen.bmj.com/site/about/guidelines.xhtml

Hospital Anxiety and Depression Scale	x	x	x	
Satisfaction with Care at the End of Life in Dementia Scale	x	x	x	
Comfort Assessment in Dying Scale			x	
EQ-5D-5L	x	x	x	
Qualitative interviews			x	x

DATA ANALYSIS

Data will be collected at the start of the intervention, and at monthly time points until a resident dies or until the end of the intervention period (6 months). This will ensure a detailed understanding and, because of the mortality rates expected, minimize attrition. Data will be entered into a password protected anonymised database by the research team.

Quantitative analysis

We will use simple descriptive statistics to summarise process data and the outcomes collected by the ICL at the care home level (i.e. number of deaths in the last month etc.) We will describe the demographic and clinical characteristics of residents and family carers who participate in the data collection, as well as symptoms experienced, interventions received and any sentinel events. We will describe the symptom burden and quality of care received using SWEOLCD, QUALID. We will compare the scores to the results of our previous study in order to gain inferences on whether the enhanced care project makes a difference. The results will be summarised using mean and standard deviation or alternatives in case of non-normally distrusted data. Appropriate plots will also be produced.

Qualitative analysis

The interviews will be audio-taped, transcribed verbatim and entered onto a qualitative software programme (Atlas-ti) for the coding, management and retrieval of data. Transcripts will be analysed and coded using Thematic Analysis. The data analysis process will follow the guidelines provided by Braun and Clarke (45) to develop meaningful themes and a rigorous approach to data analysis will be adopted by working to the quality framework recommended by Spencer (46). Throughout the analytic process, the researchers will engage in ongoing reflection with the use of memoing and reflective diaries to engage with the data further and refine emergent themes. Data triangulation will be achieved by interviewing both family carers and care home staff from a variety of work roles (i.e., care home manager, health care assistant, nurse) to explore the facilitators and barriers to the implementation of the enhanced model of care from different perspectives.

For peer review only - http://bmjopen.bmj.com/site/about/guidelines.xhtml

Final analyses

After full data collection ends, we will undertake definitive analyses to detail the demographic features of the cohort and assess the symptom management and their health care needs (using Stirling, Waterlow, NPI and sentinel events), taking into account repeated measures on individual subjects. We shall describe the level and nature of unmet needs and examine descriptively (using mean and standard deviations or suitable alternatives in case of non-normally distributed data and graphs) how comfort and quality of life change over time (using PAINAD, SM-EOLD, SW-EOLD and QUALID). We will describe the trajectory of carer wellbeing (HADS and Zarit Buden Inteview) during their friend/relative's final stages of life with severe memory problems and how this may change if the resident dies, using plots of the of wellbeing over time.

Sample size

This is a pilot study and as such a formal power calculation is not appropriate. Numbers are chosen on pragmatic grounds as sufficient to demonstrate feasibility in terms of recruitment and acceptance of the intervention. We will aim to recruit 30 residents with severe memory problems from two care homes from which to collect individual outcome data.

Health economics

Health economic evaluation will consider resource allocation in caring for patients with severe problems and, where relevant, in their last 6 months of life, as well as the quality of life of their family carers and associated economic impact on these family carers in this period.

Data on resource and service use for people with severe memory problems (RUD-Lite) and economic burden on family carers (Zarit Burden Interview) will be collected both at baseline and monthly after the enhanced care project has been implemented. These data will be collated with unit costs data from *Unit Costs of Health and Social Care (2012)* (47;48) to obtain costs per patient from NHS (such as averted hospital admission, costs for a typical episode), costs from personal social services (such as training and education for care home staff) and costs from societal perspectives (such as local commissioners' decisions on scarce resource allocation, additional costs to public purse where caring responsibilities had been met by the state instead of family carers).

Economic evaluation of the quality-adjusted life years (QALYs) for family carers will utilize EQ-5D-5L instrument to assess if enhanced care project has resulted in greater utility attained for this group and associated cost-effectiveness.

PROJECT MANAGEMENT

For peer review only - http://bmjopen.bmj.com/site/about/guidelines.xhtml

Registration, sponsorship and indemnity

The project will be registered with the research departments at the participating CCG. University College London will be the project sponsor and provide insurance. The research team will obtain honorary clinical contracts for each participating CCG, adhering to the Marie Curie Palliative Care Research Unit's Lone Worker Policy (2012).

Data protection

Case Report Forms (CRF) for the study will be stored in accordance with the Declaration of Helsinki. Electronic data will be anonymised and stored on a password protected database. At the end of the study anonymised files will be stored securely in a secure UCL archiving facility.

Research network support

The programme has been adopted by the DeNDRoN (Dementias and Neurodegenerative Diseases Network)

Project Staffing

The person appointed to the ICL post will have extensive experience in the care of older people and their family carers in care home settings and with expertise in severe memory problems and social care. They will deliver the intervention with the core team. They will be supervised by the PI (Dr Louise Jones) and, given the nature of the work, offered supportive clinical supervision by Dr E Sampson. The ICL will receive training to acclimatise them to the care homes in which they will be working and familiarise them with the intervention manual. They will have a monthly meeting with the project team to check adherence to the principles of the manual and to make any necessary adaptations to this. Two clinical researchers, from the Marie Curie Palliative Care Research Unit, who have extensive clinical and research experience with both palliative care and people with severe memory problems and family carers will collect the individual data for the evaluation of the enhanced care intervention. The researchers have particular skills in interviewing bereaved family carers and relatives.

Core study team

Dr Louise Jones, Head of Unit, is PI and guarantor for the programme. She leads the Marie Curie palliative care research team at UCL. She is a palliative care physician and expert in qualitative and quantitative research in end of life care in a range of long term conditions. She has a long history of collaboration with other members of the team.

Dr Elizabeth Sampson is an international expert in end of life care research in dementia. She has expertise in epidemiology and old age psychiatry and leads the dementia research

For peer review only - http://bmjopen.bmj.com/site/about/guidelines.xhtml

group within the Marie Curie research team at UCL where she is deputy Head of Unit. She will lead this research programme and manage the research team.

Professor Michael King the director of the Division of Psychiatry at UCL, in which the Marie Curie Unit resides. He is co-director of PRIMENT Clinical Trials Unit which specialises in trials in mental health and primary care. He is expert in epidemiology, development and evaluation of complex health care interventions and clinical trials. He will provide expertise in particular for the development and testing of our intervention.

Professor Irwin Nazareth is professor of Primary Care and head of department of Primary Care and Population Health at UCL. He is co-director of PRIMENT Clinical Trials Unit. He is expert in epidemiology, development and testing of complex healthcare interventions.

Professor Stephen Morris is professor of Health Economics UCL. He is expert in economic evaluations of complex healthcare interventions and NHS databases and will provide expertise on health economics for all workstreams.

Professor Rumana Omar is professor in Biostatistics UCL and expert in analysing complex datasets where, because of the nature of the cohort under study, data may be missing.

Professor Gerard Leavey is a social scientist who is expert in qualitative research particularly in complex mental health conditions. He leads the Northern Ireland centre for mental health research and policy (NIAMH) and is academic lead for the Ulster hub of the All Ireland Institute for Palliative Care Research.

Membership of our expert steering group

We have convened an expert steering group that has met every six months throughout the programme. The core members of our research team bring expertise in end of life care, care of the elderly, old age psychiatry, health services research, epidemiology, primary care, social science, health economics and statistics. To complement this skill mix we have included a further range of expertise through the external membership of our expert steering group:

Experts in dementia care research- in secondary care - Professor Gill Livingston (UCL), and in primary care-Professor Louise Robinson (Newcastle)

Experts in end of life care: Min Stacpoole (Senior Nurse, St Christopher's Hospice), Claire Henry (Lead NHS National End of Life Programme), Karen Harrison-Dening (Consultant Admiral nurse, Dementia UK and dementia policy adviser to Marie Curie Cancer Care)

Experts in social care: Sharon Blackburn, Chief Executive, English Care Homes association (ECCA), Graham Stokes, BUPA, to represent the private sector

Expert by experience: Mr John Sprange

For peer review only - http://bmjopen.bmj.com/site/about/guidelines.xhtml

Patient and Public Involvement

Mr John Sprange will participate in our steering group. His input will be essential and we will encourage and facilitate him in this work through our local Camden Services User Research Forum (SURF).

STUDY OUTPUTS

Dissemination

We shall prepare documents for dissemination by end of life and dementia care organisations such as Marie Curie Cancer Care, BUPA, Dementia UK, The Alzheimer's Society, National End of Life Care programme and the government special advisor for dementia including detailed reports, scientific presentations and papers for peer reviewed journals, and publicise our findings on the Marie Curie website. A summary will be provided to all participants who would like to receive this.

REFERENCES

(1) Knapp M, Privette A. Dementia UK. London: Alzheimer's Society; 2007.

(2) Brayne C, Gao L, Dewey M, Matthews FE. Dementia before death in ageing societies--the promise of prevention and the reality. PLoS Med 2006 October;3(10):e397.

(3) Dewey ME, Saz P. Dementia, cognitive impairment and mortality in persons aged 65 and over living in the community: a systematic review of the literature. Int J Geriatr Psychiatry 2001 August;16(8):751-61.

(4) Sampson EL, Bulpitt CJ, Fletcher AE. Survival of community-dwelling older people: the effect of cognitive impairment and social engagement. J Am Geriatr Soc 2009 June;57(6):985-91.

(5) Reisberg B. Functional assessment staging (FAST). Psychopharmacol Bull 1988;24(4):653-9.

(6) Mitchell SL, Teno JM, Kiely DK, Shaffer ML, Jones RN, Prigerson HG et al. The clinical course of advanced dementia. N Engl J Med 2009 October 15;361(16):1529-38.

(7) McCarthy M, Addington-Hall J, Altmann D. The experience of dying with dementia: a retrospective study. International Journal of Geriatric Psychiatry 1997 March;12(3):404-9.

For peer review only - http://bmjopen.bmj.com/site/about/guidelines.xhtml

(8) Sampson EL, Blanchard MR, Jones L, Tookman A, King M. Dementia in the acute hospital: prospective cohort study of prevalence and mortality. Br J Psychiatry 2009 July;195(1):61-6.

(9) Sampson EL, Gould V, Lee D, Blanchard MR. Differences in care received by patients with and without dementia who died during acute hospital admission: a retrospective case note study. Age Ageing 2006 March;35(2):187-9.

(10) Davies, E. and Higginson, I. J. Better palliative care for older people. World Health Organisation Europe; 2004.

(11) Sanders S, Swails P. Caring for individuals with end-stage dementia at the end of life. Dementia 2009;8:117-38.

(12) McCarty CE, Volicer L. Hospice access for individuals with dementia. Am J Alzheimers Dis Other Demen 2009 December 20;24(6):476-85.

(13) Department of Health. End of life care strategy: Promoting high quality care for all adults at the end of life. London: Department of Health; 2008.

(14) Bourne, J. Improving services and support for people with dementia. National Audit Office; 2007.

(15) Sampson EL, Ritchie CW, Lai R, Raven PW, Blanchard MR. A systematic review of the scientific evidence for the efficacy of a palliative care approach in advanced dementia. Int Psychogeriatr 2005 March;17(1):31-40.

(16) Goodman C, Evans C, Wilcock J, Froggatt K, Drennan V, Sampson E et al. End of life care for community dwelling older people with dementia: an integrated review. Int J Geriatr Psychiatry 2009 August 17.

(17) The coalition: our programme for government. London: Cabinet Office; 2010.

(18) Fitch, K., Bernstein, S. J., Aguilar, M., Burnand, B., LaCalle, J., Lazaro, P., van het Loo, M., McDonnell, J., Vader, J. P., and Kahan, J. P. The Rand/UCLA appropriateness method user's manual. Santa Monica: RAND; 2001.

(19) Grol RP, Bosch MC, Hulscher ME, Eccles MP, Wensing M. Planning and studying improvement in patient care: the use of theoretical perspectives. Milbank Q 2007;85(1):93-138.

(20) Ferlie EB, Shortell SM. Improving the quality of health care in the United Kingdom and the United States: a framework for change. Milbank Q 2001;79(2):281-315.

(21) Greenhalgh T, Robert G, Macfarlane F, Bate P, Kyriakidou O. Diffusion of innovations in service organizations: systematic review and recommendations. Milbank Q 2004;82(4):581-629.

(22) American Psychiatric Association. Diagnostic and Statistical Manual of Mental Disorders. 4th Edition ed. Washington DC: American Psychiatric Association; 1994.

For peer review only - http://bmjopen.bmj.com/site/about/guidelines.xhtml

(23) Gold Standards Framework. The Gold Standards Framework. NHS End of Life Care Programme 2008;Available from: URL: http://www.goldstandardsframework.nhs.uk/

(24) Thuné-Boyle ICV, Sampson EL, Jones L, King M, Lee D, Blanchard MR. Challenges to improving end of life care of people with advanced dementia in the UK. Dementia 2010;9:285-98.

(25) Sampson EL, Jones L, Thune-Boyle IC, Kukkastenvehmas R, King M, Leurent B et al. Palliative assessment and advance care planning in severe dementia: an exploratory randomized controlled trial of a complex intervention. Palliat Med 2011 April;25(3):197-209.

(26) Walsh K, Jones L, Tookman A, Mason C, McLoughlin J, Blizard R et al. Reducing emotional distress in people caring for patients receiving specialist palliative care. Randomised trial. Br J Psychiatry 2007 February;190:142-7.

(27) Low J, Perry R, Wilkinson S. A qualitative evaluation of the impact of palliative care day services: the experiences of patients, informal carers, day unit managers and volunteer staff. Palliat Med 2005 January;19(1):65-70.

(28) Chambers DA, Glasgow RE, Stange KC. The dynamic sustainability framework: addressing the paradox of sustainment amid ongoing change. Implement Sci 2013;8:117.

(29) Charlson ME. Studies of prognosis: progress and pitfalls. J Gen Intern Med 1987 September;2(5):359-61.

(30) Volicer L, Hurley AC, Lathi DC, Kowall NW. Measurement of severity in advanced Alzheimer's disease. J Gerontol 1994 September;49(5):M223-M226.

(31) Waterlow J. Pressure sores: a risk assessment card. Nursing Times 1985;81(48):49-55.

(32) Reid J, Morison M. Classification of pressure sore severity. Nurs Times 1994 May 18;90(20):46-50.

(33) Zwakhalen SM, Hamers JP, bu-Saad HH, Berger MP. Pain in elderly people with severe dementia: a systematic review of behavioural pain assessment tools. BMC Geriatr 2006;6:3.

(34) Cohen-Mansfield J, Marx MS, Rosenthal AS. A description of agitation in a nursing home. J Gerontol 1989 May;44(3):M77-M84.

(35) Cummings JL, Mega M, Gray K, Rosenberg-Thompson S, Carusi DA, Gornbein J. The Neuropsychiatric Inventory: comprehensive assessment of psychopathology in dementia. Neurology 1994 December;44(12):2308-14.

(36) Kiely DK, Volicer L, Teno J, Jones RN, Prigerson HG, Mitchell SL. The validity and reliability of scales for the evaluation of end-of-life care in advanced dementia. Alzheimer Dis Assoc Disord 2006 July;20(3):176-81.

For peer review only - http://bmjopen.bmj.com/site/about/guidelines.xhtml

(37) Weiner MF, Martin-Cook K, Svetlik DA, Saine K, Foster B, Fontaine CS. The quality of life in late-stage dementia (QUALID) scale. J Am Med Dir Assoc 2000 May;1(3):114-6.

(38) Wimo A, Winblad B. Resource utilisation in dementia: RUD Lite. Brain Aging 2003;3:48-59.

(39) Zarit SH, Reever KE, Bach-Peterson J. Relatives of the impaired elderly: correlates of feelings of burden. Gerontologist 1980 December;20(6):649-55.

(40) Zigmond AS, Snaith RP. The hospital anxiety and depression scale. Acta Psychiatr Scand 1983 June;67(6):361-70.

(41) Herdman M, Gudex C, Lloyd A, Janssen M, Kind P, Parkin D et al. Development and preliminary testing of the new five-level version of EQ-5D (EQ-5D-5L). Qual Life Res 2011 December;20(10):1727-36.

(42) Engel SE, Kiely DK, Mitchell SL. Satisfaction with end-of-life care for nursing home residents with advanced dementia. J Am Geriatr Soc 2006 October;54(10):1567-72.

(43) Mitchell SL, Kiely DK, Jones RN, Prigerson H, Volicer L, Teno JM. Advanced dementia research in the nursing home: the CASCADE study. Alzheimer Dis Assoc Disord 2006 July;20(3):166-75.

(44) Sampson EL, Harrison-Dening K, Greenish W, Mandal U, Holman A, Jones L. End of Life Care for People with Dementia. Marie Curie Cancer Care 2009;Available from: URL: http://www.mariecurie.org.uk/forhealthcareprofessionals/end-of-life-dementia.htm

(45) Braun V, Clarke V. Using thematic analysis in psychology . Qual Res Psychol 2006;3(2):77-101.

(46) Spencer, L., Ritchie, J., Lewis, J., and Dillon, L. Quality in qualitative evaluation: A framework for assessing research evidence. London: The Cabinet Office; 2003.

(47) Publications and statistics. Department of Health 2010;Available from: URL: www.dh.gov.uk/en/Publicationsandstatistics/Publications/PublicationsPolicyAndGuidance

(48) Curtis L. PSSRU 2009;Available from: URL: http://www.pssru.ac.uk/uc/uc2009contents.htm

For peer review only - http://bmjopen.bmj.com/site/about/guidelines.xhtml

APPENDICES

Appendix 1: Compassion intervention manual

The Final Compassion Intervention Manual will be published with free access on the Marie Curie website (www.mariecurie.org.uk).

Appendix 2. Bedford Alzheimer Nursing Severity (BANS) scale

Please refer to: Volicer L, Hurley AC, Lathi DC, Kowall NW. Measurement of severity in advanced Alzheimer's disease. J Gerontol 1994 September;49(5):M223-M226.

Appendix 3: Waterlow scale

Please refer to: Waterlow J. Pressure sores: a risk assessment card. Nursing Times 1985;81(48):49-55.

Appendix 4: Stirling Wound Assessment Scale

Please refer to Reid J, Morison M. Classification of pressure sore severity. Nurs Times 1994 May 18;90(20):46-50.

Appendix 5: Pain Assessment in Advanced Dementia (PAIND)

Please refer to: Zwakhalen SM, Hamers JP, bu-Saad HH, Berger MP. Pain in elderly people with severe dementia: a systematic review of behavioural pain assessment tools. BMC Geriatr 2006;6:3.

Appendix 6: Cohen Mansfield Agitation Inventory (CMAI)

Please refer to: Cohen-Mansfield J, Marx MS, Rosenthal AS. A description of agitation in a nursing home. J Gerontol 1989 May;44(3):M77-M84.

Appendix 7: Neuropsychiatric Inventory (NPI) questionnaire

Please refer to: Cummings JL, Mega M, Gray K, Rosenberg-Thompson S, Carusi DA, Gornbein J. The Neuropsychiatric Inventory: comprehensive assessment of psychopathology in dementia. Neurology 1994 December;44(12):2308-14.

Appendix 8: Symptom Management at the End Of Life in Dementia (SM-EOLD) scale

Please refer to: Kiely DK, Volicer L, Teno J, Jones RN, Prigerson HG, Mitchell SL. The validity and reliability of scales for the evaluation of end-of-life care in advanced dementia. Alzheimer Dis Assoc Disord 2006 July;20(3):176-81.

Appendix 9: Quality of Life in late-stage Dementia (QUALID)

Please refer to: Weiner MF, Martin-Cook K, Svetlik DA, Saine K, Foster B, Fontaine CS. The quality of life in late-stage dementia (QUALID) scale. J Am Med Dir Assoc 2000 May;1(3):114-6.

For peer review only - http://bmjopen.bmj.com/site/about/guidelines.xhtml

Appendix 10: Resource Utilisation in Dementia (RUD) - Lite

Please refer to: Wimo A, Winblad B. Resource utilisation in dementia: RUD Lite. Brain Aging 2003;3:48-59.

Appendix 11: The Comfort Assessment in Dying with Dementia scale (CAD-EOLD)

Please refer to: Kiely DK, Volicer L, Teno J, Jones RN, Prigerson HG, Mitchell SL. The validity and reliability of scales for the evaluation of end-of-life care in advanced dementia. Alzheimer Dis Assoc Disord 2006 July;20(3):176-81.

Appendix 12: The Zarit Burden Interview

Please refer to: Zarit SH, Reever KE, Bach-Peterson J. Relatives of the impaired elderly: correlates of feelings of burden. Gerontologist 1980 December;20(6):649-55.

Appendix 13: Hospital Anxiety and Depression Scale (HADS)

Please refer to: Zigmond AS, Snaith RP. The hospital anxiety and depression scale. Acta Psychiatr Scand 1983 June;67(6):361-70.

Appendix 14: The Satisfaction with Care at the End-of-Life in Dementia Questionnaire (SWC/CAD-EOLD)

Please refer to: Kiely DK, Volicer L, Teno J, Jones RN, Prigerson HG, Mitchell SL. The validity and reliability of scales for the evaluation of end-of-life care in advanced dementia. Alzheimer Dis Assoc Disord 2006 July;20(3):176-81.

Appendix 15: EQ-5D-5L

Please refer to: Herdman M, Gudex C, Lloyd A, Janssen M, Kind P, Parkin D et al. Development and preliminary testing of the new five-level version of EQ-5D (EQ-5D-5L). Qual Life Res 2011 December;20(10):1727-36.

For peer review only

Appendix 16: Health Care Professional Qualitative Interview Schedule

HCP interview schedule Compassion Study (HCP interview schedule for intervention V1 09.01.2014)

Preamble

Thank you for agreeing to this interview. As you know we have introduced an Interdisciplinary Care Leader into the care home in which you work. The reason why we have invited you today for this discussion is to understand what your thoughts are on this service and if there was anything about this service that you think can be improved. Also, please be assured that the topics that we discuss today are strictly confidential and will remain completely anonymous.

Interview

Firstly, just for the purposes of the recording can you:

1. Describe your current role here

2. The type and amount of contact you have on a day to day basis with residents with severe memory problems (how severe these are, their roles and responsibilities)

Now I would like to talk about the role of the ICL and how it may have influenced the way you perform your job:

3. Tell me about how you found working with the ICL

4. Did the ICL influence the way you performed your role? If so, how? Can you provide some examples of how the ICL did this?

5. Do you think the ICL changed the care you provided to residents?

For peer review only - http://bmjopen.bmj.com/site/about/guidelines.xhtml

The Compassion Programme: WS3- pilot study Version 1, 24th January 2014

6. Did you find that it influenced any of the following:
    a. Your knowledge of dementia
    b. How you assess patients with severe memory problems
    c. How you recognise symptoms such as pain and how you manage these symptoms?
    d. Were you given any support and guidance on initiating and implementing advance care plans? If so, can you give us an example of when this happened?
    e. The way you communicate/interact with patients who are no longer able to communicate
    f. How comfortable you are about communicating with family members, including discussions about palliative care and death/dying
    g. How you communicate with other HCPs

7. Tell me about your needs. Did the ICL influence the support that you receive in your role?
    a. E.g., such as support following patient death

For care home manager: Did you notice any changes in the way your staff provided care to patients? How do you feel the ICL was received by your staff?

8. Is there anything about this service that can be improved? Is there anything that you would do differently if you were implementing this service?

For peer review only - http://bmjopen.bmj.com/site/about/guidelines.xhtml

Appendix 17: Family Carer Qualitative Interview Schedule

Family carer interview schedule (Compassion Study - Carer interview schedule for intervention V1 09.01.2014)

Preamble

Thank you for agreeing to this interview. The reason that we have invited you along for this discussion is to get an idea of the care and support that you and your relative have received over the last few months. If you feel that you need to stop or leave the room at any time please tell me. Whatever you tell me will be made anonymous for the purposes of the study.

Interview

I'd like to begin by asking you a little bit about X memory problems and your understanding of his/her illness

1. Tell me about X's illness and symptoms over the last few months
    a. Both physiological and psychological needs

2. Tell me about the types of support or services has X received over the last few months
    a. Formal or informal (Religion/spirituality)
    b. Satisfaction

Now I'd like to ask you some questions about your needs as a carer:

3. How have you found dealing with X's illness over the last few months? What have you found particularly difficult?
    a) Both physiological and psychological needs
    b) Own mental health

4. Tell me about the support that you needed including emotional, psychological and social needs religious/spiritual needs. Were your needs assessed?

For peer review only - http://bmjopen.bmj.com/site/about/guidelines.xhtml

a. If so, tell me about the services that you were offered to meet these needs (If yes, determine who this was offered by and when this took place)

5. Did you have any discussions with HCP's (GP, Consultant, nursing home staff etc) about (if yes, determine when these took place):
    a. Course of illness
    b. Additional information
    c. Treatments – decision making – past and future
    d. Inclusion of other family members

6. Has anyone discussed your thoughts if X's condition were to deteriorate? If so, who discussed these with you and when?

    a) POA
    b) DNAR
    c) Place of death
    d) ACP – Feasibility of carrying out another person's wishes

7. Has anyone discussed what the future holds for X?
    a. i.e., religious beliefs/spirituality – Any recognition in the home?

8. We would also like to find out if the ICL has influenced the care and support that you and your relative have received over the last few months.
    a. Tell me about any changes to the care and support that both you and X have received over the last few months
    b. Tell me if these changes had a positive or a negative impact on you and X
    c. Ways in which we can improve this service? How else can the ICL help you and your relative?

Additional question if patient has passed away: Can you tell me a little about what happened when X passed away?

For peer review only - http://bmjopen.bmj.com/site/about/guidelines.xhtml

a) Did it all go smoothly?

b) Were their end of life wishes met? (such as religious/spiritual wishes)

c) Did you receive immediate and ongoing bereavement, emotional and spiritual support?

For peer review only

Evaluation of the implementation of the COMPASSION intervention to improve care towards the end of life for people with advanced dementia residing in two care homes in north London: assessment of long term effects, maintenance and sustainability.

The COMPASSION programme research team:

L Jones, E L Sampson, K Moore, M Elliott , N Kupeli, S Davis, J Harrington, B Candy, V Vickerstaff, A Gola, M King, G Leavey,S Morris, I Nazareth, R Z Omar

For peer review only

Background

The COMPASSION intervention (available from the authors) was developed through a 3 year NIHR portfolio research programme funded by Marie Curie Cancer Care (Jones et al 2012) and it aims to improve end of life care for people with advanced dementia. In the final year of the programme, COMPASSION was implemented, in two care homes in two different clinical commissioning groups, in north London in an exploratory study (ref Elliott 2014).

COMPASSION consists of two key components enabled by an interdisciplinary care leader (ICL) working with the multidisciplinary team within the care home and with associated primary and secondary care providers. These components are: (i) facilitation of integrated care (ii) provision of training and support for care home staff and family carers. We anticipate that there will be ripple and diffusion effects that will influence a third component which is the wider political, economic and commissioning environment within each clinical commissioning group.

The two study sites differed in their level of readiness for receipt of the intervention: service provision for care at the end of life for people with advanced dementia was thought to be more developed at the Camden care home. The exploratory study commenced between May and June 2014 and lasted for 6 months at each site.

An important part of understanding the effects of complex healthcare interventions is collecting evidence on their long term effects, both positive and negative, checking for evidence of potential harms, and what factors are affecting maintenance of any change exerted by the intervention (MRC 2008). Much thought has been given to how maintenance and sustainability might be assessed. In a recent paper, Chambers et al 2013 suggest that when an innovation team leaves a test site, it becomes difficult for the routine service providers to adhere to the new model as closely and 'programme drift' and 'voltage drop' (reduced adherence to protocols) are natural and inevitable processes. However, they argue that each site may adapt what they have learned from the innovation and continue to behave in newly adapted ways that are sympathetic to their own particular context. Thus those components of an intervention that are effective and workable will vary between sites. It is likely that, given this flexibility, such mechanisms are most likely to lead towards the aims and objectives of the intervention or innovative model of care.

Aims

We aim to assess the longer term effects of implementation of COMPASSION at two care home sites by understanding the impact of the intervention on members of the multidisciplinary team involved in the care of residents with advanced dementia.

For peer review only - http://bmjopen.bmj.com/site/about/guidelines.xhtml

Design

We shall collect qualitative data from a purposive sample of health and social care professionals in the care home and in associated primary and secondary care services. We shall seek to understand any alterations in how services are organized and resources allocated (such as changes in staffing levels, engagement of the multi-disciplinary team across primary and secondary care) that have occurred since the COMPASSION exploratory intervention team exited the site. We shall use a realist approach to analyzing the data to enable an understanding of the contexts and mechanisms that are operating that are likely to affect outcomes in the care of people with advanced dementia (Pawson and Tilley 1997). We shall consider the mechanisms at the 4 levels recommended in the study of organizational change: individual, group, organizational, and wider economic and political context (Ferlie and Shortell 2001; Grol 2007)

Study setting

2 care homes in North London, UK. BLINDED TO MEET ETHICAL REQUIREMENTS STATED BELOW.

Sample

A maximum of 10 health and social care providers at each site. We shall attempt to approach professionals who have previously been interviewed as part the piloting of our intervention in an exploratory study (Elliott 2014). Where there has been staff turnover, we shall attempt to interview the newly hired personnel. We expect our sample to include health care assistants, trained nursing staff, allied health professionals, social care professionals, care home managers, general practitioners, and members of specialist services such as community palliative care, geriatricians and mental health providers.

Procedures

Participants will be given an information sheet and at least 48 hours to consider whether they wish to take part. Those who agree will be asked to give informed consent to two in-depth qualitative interviews that will be audio-taped and transcribed verbatim. The first interview will take place 4 months after the COMPASSION exploratory team left the site; the second after a further 4 months. Interviews will last between 15-60 minutes. We shall work to a topic guide and our focus will be on understanding the experience of the intervention, whether and how it has affected practice, whether and how it has affected behaviours of individuals and teams, whether and how it has been thought to influence care. We shall explore with care home managers whether there have been changes in resource allocation, service organization and personnel, and whether there have been any effects on the behaviours of the care home owners. In speaking with any newly hired personnel we shall attempt to understand whether any of the effects of COMPASSION are thought to have diffused into their training and practice. In this way we hope to gain an understanding of whether components of COMPASSION have started to become embedded in the culture of each care home.

Data will be collected by a member of the research team who was not involved in the implementation of the COMPASSION intervention.

For peer review only - http://bmjopen.bmj.com/site/about/guidelines.xhtml

Analysis

Transcribed interviews will be read and coded for emergent themes using framework analysis (Ritchie and Spencer 1993). Coding and themes will be checked by a second member of the research team. We shall then hold meetings of the wider research team, including those involved in implementation in the exploratory study, to discuss what themes are emerging and categorise them to understand the contexts, mechanisms and outcomes that are operating. We shall use these data to develop a realist programme theory for sustainability of COMPASSION.

We shall consider data collected at four months to develop a provisional programme sustainability theory, and this will be refined in an iterative process using data collected at eight months. See Figure 1 below.

Figure 1. Realist analysis of data and development of programme theories

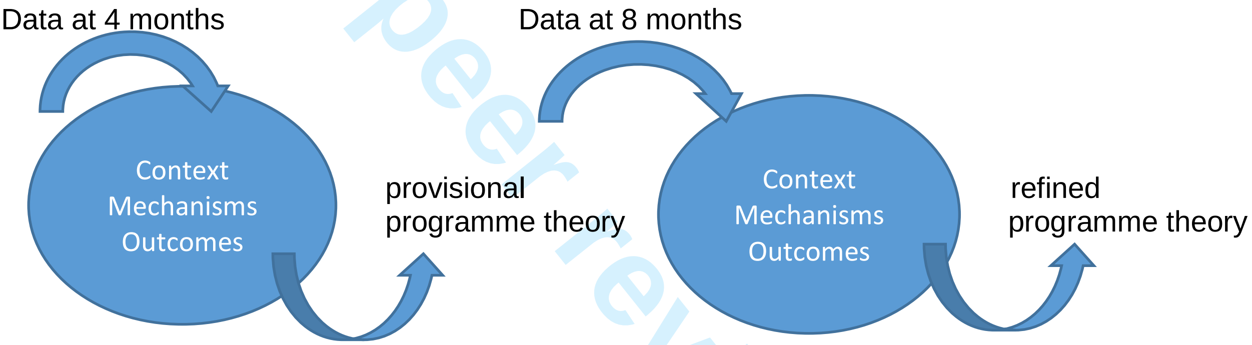

We shall merge these data with qualitative data collected from similar health and social care professionals during the exploratory study to refine an overall programme theory of how COMPASSION has operated throughout its implementation and beyond.

We shall attempt to understand which components of COMPASSION are key to its implementation and which sections of the intervention manual are followed most closely. We shall attempt to describe and understand the reasons for programme drift and voltage drop described by Chambers 2013. We shall consider how our data inform further amendments to the structure and content of COMPASSION and the role of the ICL who was the key implementation person working at each site during the exploratory study. This will allow us to adapt and tailor the intervention manual accordingly.

Economic considerations

We shall not collect any economic data directly. However, we shall use the understanding gained from the qualitative data and work with the health economist within our wider research team to explore how COMPASSION components 1 and 2 have influenced attitudes to commissioning and the wider economic and political context within each participating clinical commissioning group. We shall use refinements we make to the COMPASSION manual to consider the costs of the core components that we retain and whether resource allocation has altered since the intervention ceased. This will inform recommendations for further roll out of the intervention at other sites and for consideration by service planners and providers in the

For peer review only - http://bmjopen.bmj.com/site/about/guidelines.xhtml

clinical commissioning groups, the care home provision system and providers of end of life care and care of people with dementia in the NHS and the voluntary sector.

Ethical issues

Data collection in this work will involve health and social care professionals only who will be given information sheets in advance of giving written informed consent for participation in audio-taped interviews. All data will be anonymized and no individual or research site will be identifiable in reports or publications arising from the work.

Data will be kept in locked cabinets using usual procedures within the research department and all procedures will conform to the Data Protection Act.

Plans for dissemination

Findings from this work will be prepared for publication at national and international conferences, in scientific journals and as part of policy documents prepared by organisations involved in dementia and end of life care such as the Alzheimer's Society and Marie Curie.

Findings will be merged with other data arising from the COMPASSION programme. Learning from the programme will be used within the MARQUE programme, funded by ESRC and NIHR in workstreams led by our research team. MARQUE is one of the tranches of work arising from the UK Prime Minister's Dementia Challenge 2013.

References

Chambers DA, Glasgow RE, Stange KC. The dynamic sustainability framework: addressing the paradox of sustainment amid ongoing change. Implement Sci. 2013 Oct 2;8:117. doi: 10.1186/1748-5908-8-117.

Craig P, Dieppe P, Macintyre S, et al. Developing and evaluating complex interventions: the new Medical Research Council guidance. BMJ 2008;337:979–83

Elliot, M., Harrington, J., Moore, K., Davis, S., Kupeli, N., Vickerstaff, V. H., Gola, A., Candy, B., Sampson, E. L., Jones, L. (2014). A protocol for an exploratory phase i mixed-methods study of enhanced integrated care for care home residents with advanced dementia: The compassion intervention. *BMJ Open* doi:10.1136/bmjopen-2014-005661

Grol RPTM, Bosch MC, Hulscher MEJL, Eccles MP, Wensing M. Planning and stuying improvement in patient care: the use of theoretical perspectives. Millbank quarterly Vol 85 No1 2007 (pp93-138)

Jones L, Harrington J, Scott S, et al. CoMPASs: IOn programme (Care of Memory Problems in Advanced Stages of dementia: Improving Our Knowledge): protocol for a mixed methods study. BMJ Open 2012;2:e002265. doi:10.1136/bmjopen-2012-002265

R Pawson and N Tilley (1997) xvii + 235p *REALISTIC EVALUATION*, London: Sage.

Spencer L, Ritchie J, Lewis J, *et al. Quality in qualitative evaluation: A framework for assessing research evidence.* London: The Cabinet Office; 2003. http://www.civilservice.gov.uk/wp-content/uploads/2011/09/a_quality_framework_tcm6-38740.pdf (accessed May 2014)

For peer review only - http://bmjopen.bmj.com/site/about/guidelines.xhtml

Supplementary File 3: Context of each NH

Level	Both NHs	
**Political and economic context (health and social care system in the UK)**	• Both NHs located in north London<br>• Despite policy attempts to integrate services (e.g. Better Care Fund), funding and management of social services are separate from the National Health Service (NHS)<br>• NHs operate within the social service system<br>• The majority of NHs are privately run entities operating for profit<br>• Residents are assessed for eligibility for continuing care funding from their local authority or pay privately for social care<br>• Clinical Commissioning Groups (CCGs) manage priorities for funding of healthcare services and operate locally. The two NHs were located within different CCGs. UK residents are entitled to services through the NHS<br>• Other specialist and allied health services should be available in all NHs, however, access and availability can be uneven[1]<br>• NHs do not require a nurse to be employed, unless beds are allocated as nursing home beds<br>• Some NH beds are also allocated as dementia specific, requiring the NH to have staff with expertise in dementia care	
**Organisational context**	Both NHs privately run by larger companies operating multiple NHs.	
**CCG context**	**Camden CCG**	**Barnet CCG**
Index of Multiple Deprivation#[2]	• 3rd decile of relative deprivation	• 6th decile of relative deprivation
Number of NHs in CCG*	• 13 NHs and care homes (not 24 hour nursing support)	• 91 NHs and care homes (not 24 hour nursing support)
Relevant CCG priorities	• 'Frail and elderly' programme<br>• 'Long term conditions and Cancer' programme[3]	• 'End of life' priority[4]
**Individual NH context**	**NH1**	**NH2**
Beds and levels of care	99 nursing home beds across five units including one dementia specific unit and one younger people with disabilities (not engaged in Intervention)	77 beds with three units: residential care and two nursing care units, one was dementia specific.
Management	Manager and deputy manager. Deputy manager retired half way through implementation.	Manager and Deputy manager. Deputy manager resigned in the weeks prior to implementation.
Nursing and healthcare assistants	Each unit managed by a nurse 24 hours a day with up to five healthcare assistants. Staff involved in direct care work 12hr shifts from 8:00-20:00 or 20:00-8:00	Both nursing units managed by a nurse with up to five healthcare assistants.
Activity co-ordinator	3 part-time staff (approx. 2 full time equivalent)	1 full time
External healthcare professionals		

For peer review only - http://bmjopen.bmj.com/site/about/guidelines.xhtml

Level	Both NHs	
GP	All residents registered with one GP clinic. Regular GP visits for 2X3hr sessions per week.	Residents registered with one GP clinic. Regular GP visits for 1X3hr session per week.
Actively involved at NH	Dietetics/nutrition, Geriatrics, Nursing (palliative care; tissue viability; Mental Health), Occupational Therapy, Physiotherapy (although long waiting lists are a deterrent), Podiatry, Social Work, Speech and Language Therapy, Hospital programme facilitating safe discharge from emergency department for complex and frail older patients.	Speech and Language Therapy, Old Age Psychiatry, district nursing (for non-nursing unit)
Available if required	Old Age Psychiatry, psychology	Nursing (palliative care and mental health)
Not available	Care of the Elderly	Geriatrics
Care planning	Care plans are monitored on a monthly basis by the nurse. They are kept as paper based records in the relevant nurse's office. There are templates for different areas of care. Examples of assessments used include: Abbey Pain scale; Doloplus 2, Cornell Depression Scale and Geriatric Depression Scale, Malnutrition Universal Screening Tool, Waterlow Pressure Ulcer Risk, Bradford Dementia Group Wellbeing Profile. Residents typically have 14-20 different care plans. Sentinel events or a significant change in condition will lead to a review and potentially instigating a new care plan as indicated.	Care plans are monitored on a monthly basis by the nurse. They are kept as paper based records in the relevant nurse's office. The template includes 25 different care needs.
Communication processes	• Documentation is manually recorded. Only the manager enters data for generating report back to the NH company.<br>• Verbal handover occurs twice daily during change of shift.<br>• Offer meetings for family members; recent poor attendance was leading the manager to query continued value.<br>• Nurses communicate with other nurses on the same floor working on different shifts using a communication book.<br>• Care plans include communication pages to report when healthcare professionals or family members have had discussions/appointments with NH staff.	• Documentation is manually recorded.<br>• No central place for recording deaths, hospitalisations or other adverse events.<br>• Nurses report in resident care plan on a daily basis and review care plans on a monthly basis. Nurses keep dairies to record resident medical appointments etc.<br>• Handover occurs at staff changeover.<br>• Regular family meetings are held.
Training and professional development	• 40 care staff have National Vocational Qualifications; 20 enrolled in health and social care training.<br>• Electronic matrix shows when each staff member completed compulsory and non-compulsory training flagging those who are due. There are 11 mandated competencies reviewed regularly.<br>• Training sessions rune on a regular basis – staff are informed via flyers in each unit. Sessions are scheduled at a set hour that is the quietest in the afternoon. Expectation that up to half of the staff currently working are given the opportunity to attend.	• No formalised structure for running regular training programmes. Training no longer offered via local palliative care service.<br>• A multi-day dementia training programme was run on an annual for a small number of staff to complete.

For peer review only

For peer review only - http://bmjopen.bmj.com/site/about/guidelines.xhtml

Level	Both NHs	
	• Access to training is not available to staff who have been in the country for less than three years. This can be a barrier for upskilling staff.	
Dementia and palliative care	• An advance care plan is developed on admission.<br>• Specialist Palliative Care Nurse from the local hospital's community palliative care nursing service visits the NH regularly and manages complex symptoms at EOL and provides staff training in palliative care. Links commenced 6-7 years earlier when palliative care felt that the NH's referrals were low or inappropriate.<br>• Use local electronic register to inform emergency and out-of-hour services about residents at the EOL and documented care wishes such as 'Do Not Attempt Resuscitation'<br>• 60 nursing and care staff were enrolled (prior to Intervention) in a distant education course about dementia.<br>• The manager attends local dementia strategy meetings.<br>• Manager frustrated by lack of consensus on best care in dementia. Manager felt staff needed more understanding of biological processes in dementia to help understand why a resident is acting the way they are.<br>• Annual memorial function with religious service; family of deceased residents invited.<br>• Two nurses (prior to the Intervention) were attending Gold Standards Accreditation training. Accreditation not achieved during implementation.<br>• During implementation it became evident that there were a range of staff development needs to build skills in dementia and palliative care	• They use the Gold Standards Framework to assess whether residents are nearing EOL. Specialist palliative care specifies that they are only to be called for 'signs and symptoms' (primarily pain management).<br>• Prior to the Intervention they had introduced forms regarding Power of Attorney, the Mental Capacity Act and deprivation of liberty.<br>• Staff have completed syringe driver training; no team available to provide IV antibiotics.<br>• One nurse (prior to the Intervention) attending Gold Standards Accreditation training with the goal of achieving accreditation. Not achieved during implementation.<br>• During implementation it became evident that there were a range of staff development needs to build skills in dementia and palliative care

1st decile = most deprived
* Source: http://www.carehome.co.uk/care_search.cfm (accessed 20th October 2016)

References

1. Seymour JE, Kumar A, Froggatt K. Do nursing homes for older people have the support they need to provide end-of-life care? A mixed methods enquiry in England. *Palliative medicine* 2011;25(2):125-38 doi: doi: 10.1177/0269216310387964published Online First: Epub Date]|.
2. Department for Communities and Local Government. English Indices of Deprivation 2015. Open Government License 2015.
3. NHS Camden Clinical Commissioning Group. 2014/15 Final Annual Report and Outcomes: Working with the people of Camden to achieve the best health for all, 2015.
4. NHS Barnet Clinical Commissioning Group. Annual Report and Accounts 2014/15: Working with local people to develop seamless, accessible care for a healthier Barnet, 2015.

For peer review only - http://bmjopen.bmj.com/site/about/guidelines.xhtml

For peer review only

Supplementary File 4: ICL time spent by activity by hours

Activity	NH1 hours (%)	NH2 hours (%)	Hours not attributable to a NH (%)	Total hours (%)	Total Costs*
Assessing needs					
Assessing needs**	122.75 (44)	87.75 (40)	NA	210.5 (32)	£6,241
Meeting family	9.75 (3)	14 (6)	NA	23.75 (4)	£665
Meeting staff	21.75 (8)	16.25 (7)	NA	38 (6)	£1,064
Emails/phone calls^	24 (9)	14.25 (6)	5.75 (4)	44 (7)	£869#
Core meetings	10.25 (4)	5.75 (3)	NA	16 (3)	£448
Wider Meetings	7.5 (3)	NA	NA	7.5 (1)	£210
Staff training					
Preparing training	19 (7)	34.25 (16)	26.75 (17)	80 (12)	£1,753
Providing training	14.25 (5)	19.25 (9)	NA	33.5 (5)	£1,019
Other					
Travel	47.25 (17)	29.75 (13)	30 (19)	107 (16)	£4,053***
ICL professional development	NA	NA	67 (42)	67 (10)	£1,468
ICL clinical supervision	NA	NA	28.75 (18)	28.75 (4)	£463
Total	276.5 (100)	221.25 (100)	158.25 (100)	656 (100)	£18,255

*Source for hourly rate: Department of Health and Health Education England, includes on-costs
**Includes unproductive time in the NH such as waiting to speak to staff, trying to locate staff or records etc.
***Includes cost of train fare
#excludes cost of telephone calls
^includes time speaking with or sending emails to family members
NA = not applicable

For peer review only - http://bmjopen.bmj.com/site/about/guidelines.xhtml

TREND Statement Checklist

Paper Section/ Topic	Item No	Descriptor	Reported? ✓	Pg #
**Title and Abstract**				
Title and Abstract	1	• Information on how unit were allocated to interventions	NA	
		• Structured abstract recommended	✓	2,3
		• Information on target population or study sample	✓	2
**Introduction**				
Background	2	• Scientific background and explanation of rationale	✓	4-6
		• Theories used in designing behavioral interventions	✓	4,9
**Methods**				
Participants	3	• Eligibility criteria for participants, including criteria at different levels in recruitment/sampling plan (e.g., cities, clinics, subjects)	✓	5-6
		• Method of recruitment (e.g., referral, self-selection), including the sampling method if a systematic sampling plan was implemented	✓	11,13
		• Recruitment setting	✓	9, 14-15
		• Settings and locations where the data were collected	✓	9,14-15
Interventions	4	• Details of the interventions intended for each study condition and how and when they were actually administered, specifically including:		
		○ Content: what was given?	✓	6-8
		○ Delivery method: how was the content given?	✓	8-10
		○ Unit of delivery: how were the subjects grouped during delivery?	NA	
		○ Deliverer: who delivered the intervention?	✓	6,9
		○ Setting: where was the intervention delivered?	✓	9,14-15
		○ Exposure quantity and duration: how many sessions or episodes or events were intended to be delivered? How long were they intended to last?	✓	7-8
		○ Time span: how long was it intended to take to deliver the intervention to each unit?	✓	9
		○ Activities to increase compliance or adherence (e.g., incentives)	✓	15-18
Objectives	5	• Specific objectives and hypotheses	✓	8,9
Outcomes	6	• Clearly defined primary and secondary outcome measures	✓	10-11
		• Methods used to collect data and any methods used to enhance the quality of measurements	✓	10-12
		• Information on validated instruments such as psychometric and biometric properties	✓	11,24-5
Sample Size	7	• How sample size was determined and, when applicable, explanation of any interim analyses and stopping rules	✓	8,10
Assignment Method	8	• Unit of assignment (the unit being assigned to study condition, e.g., individual, group, community)	✓	9-10
		• Method used to assign units to study conditions, including details of any restriction (e.g., blocking, stratification, minimization)	NA	
		• Inclusion of aspects employed to help minimize potential bias induced due to non-randomization (e.g., matching)	NA	

TREND Statement Checklist

Blinding (masking)	9	• Whether or not participants, those administering the interventions, and those assessing the outcomes were blinded to study condition assignment; if so, statement regarding how the blinding was accomplished and how it was assessed.	✓	10
Unit of Analysis	10	• Description of the smallest unit that is being analyzed to assess intervention effects (e.g., individual, group, or community)	✓	12
		• If the unit of analysis differs from the unit of assignment, the analytical method used to account for this (e.g., adjusting the standard error estimates by the design effect or using multilevel analysis)	NA	
Statistical Methods	11	• Statistical methods used to compare study groups for primary methods outcome(s), including complex methods of correlated data	✓	12
		• Statistical methods used for additional analyses, such as a subgroup analyses and adjusted analysis	NA	
		• Methods for imputing missing data, if used	NA	
		• Statistical software or programs used	✓	12
**Results**				
Participant flow	12	• Flow of participants through each stage of the study: enrollment, assignment, allocation, and intervention exposure, follow-up, analysis (a diagram is strongly recommended)	✓	Fig 1
		○ Enrollment: the numbers of participants screened for eligibility, found to be eligible or not eligible, declined to be enrolled, and enrolled in the study	✓	Fig 1
		○ Assignment: the numbers of participants assigned to a study condition	✓	Fig 1
		○ Allocation and intervention exposure: the number of participants assigned to each study condition and the number of participants who received each intervention	✓	Fig 1
		○ Follow-up: the number of participants who completed the follow-up or did not complete the follow-up (i.e., lost to follow-up), by study condition	✓	Fig 1
		○ Analysis: the number of participants included in or excluded from the main analysis, by study condition	✓	Fig 1
		• Description of protocol deviations from study as planned, along with reasons	NA	
Recruitment	13	• Dates defining the periods of recruitment and follow-up	✓	10
Baseline Data	14	• Baseline demographic and clinical characteristics of participants in each study condition	✓	24-5
		• Baseline characteristics for each study condition relevant to specific disease prevention research	✓	24-5
		• Baseline comparisons of those lost to follow-up and those retained, overall and by study condition	NA	
		• Comparison between study population at baseline and target population of interest	NA	
Baseline equivalence	15	• Data on study group equivalence at baseline and statistical methods used to control for baseline differences	NA	

For peer review only

TREND Statement Checklist

Numbers analyzed	16	• Number of participants (denominator) included in each analysis for each study condition, particularly when the denominators change for different outcomes; statement of the results in absolute numbers when feasible	✓	Fig 1
		• Indication of whether the analysis strategy was "intention to treat" or, if not, description of how non-compliers were treated in the analyses	NA	
Outcomes and estimation	17	• For each primary and secondary outcome, a summary of results for each estimation study condition, and the estimated effect size and a confidence interval to indicate the precision	✓	24-5
		• Inclusion of null and negative findings	NA	
		• Inclusion of results from testing pre-specified causal pathways through which the intervention was intended to operate, if any	NA	
Ancillary analyses	18	• Summary of other analyses performed, including subgroup or restricted analyses, indicating which are pre-specified or exploratory	NA	
Adverse events	19	• Summary of all important adverse events or unintended effects in each study condition (including summary measures, effect size estimates, and confidence intervals)	NA	
**DISCUSSION**				
Interpretation	20	• Interpretation of the results, taking into account study hypotheses, sources of potential bias, imprecision of measures, multiplicative analyses, and other limitations or weaknesses of the study	✓	24-28
		• Discussion of results taking into account the mechanism by which the intervention was intended to work (causal pathways) or alternative mechanisms or explanations	✓	26-7
		• Discussion of the success of and barriers to implementing the intervention, fidelity of implementation	✓	26-32
		• Discussion of research, programmatic, or policy implications	✓	28-32
Generalizability	21	• Generalizability (external validity) of the trial findings, taking into account the study population, the characteristics of the intervention, length of follow-up, incentives, compliance rates, specific sites/settings involved in the study, and other contextual issues	✓	27-8
Overall Evidence	22	• General interpretation of the results in the context of current evidence and current theory	✓	26-32

From: Des Jarlais, D. C., Lyles, C., Crepaz, N., & the Trend Group (2004). Improving the reporting quality of nonrandomized evaluations of behavioral and public health interventions: The TREND statement. *American Journal of Public Health*, 94, 361-366. For more information, visit: http://www.cdc.gov/trendstatement/

For peer review only
